# Genome-wide CRISPR Screens in T Helper Cells Reveal Pervasive Crosstalk between Activation and Differentiation

**DOI:** 10.1016/j.cell.2018.11.044

**Published:** 2019-02-07

**Authors:** Johan Henriksson, Xi Chen, Tomás Gomes, Ubaid Ullah, Kerstin B. Meyer, Ricardo Miragaia, Graham Duddy, Jhuma Pramanik, Kosuke Yusa, Riitta Lahesmaa, Sarah A. Teichmann

**Affiliations:** 1 Wellcome Sanger Institute, Wellcome Trust Genome Campus, Hinxton, Cambridge, CB10 1SA, UK; 2 Department of Biosciences and Nutrition, Karolinska Institutet, Hälsovägen 7, Novum, SE-141 83, Huddinge, Sweden; 3 Turku Centre for Biotechnology, University of Turku and Åbo Akademi University, Tykistökatu 6 FI-20520, Turku, Finland; 4 EMBL-European Bioinformatics Institute, Wellcome Trust Genome Campus, Hinxton, Cambridge, CB10 1SD, UK; 5 Theory of Condensed Matter, Cavendish Laboratory, 19 JJ Thomson Ave, Cambridge CB3 0HE, UK

**Keywords:** CD4 T helper cell, mouse, Cas9, CRISPR, pooled screen, retrovirus, knockout, overexpression, ATAC-seq, ChIP-seq

## Abstract

T helper type 2 (Th2) cells are important regulators of mammalian adaptive immunity and have relevance for infection, autoimmunity, and tumor immunology. Using a newly developed, genome-wide retroviral CRISPR knockout (KO) library, combined with RNA-seq, ATAC-seq, and ChIP-seq, we have dissected the regulatory circuitry governing activation and differentiation of these cells. Our experiments distinguish cell activation versus differentiation in a quantitative framework. We demonstrate that these two processes are tightly coupled and are jointly controlled by many transcription factors, metabolic genes, and cytokine/receptor pairs. There are only a small number of genes regulating differentiation without any role in activation. By combining biochemical and genetic data, we provide an atlas for Th2 differentiation, validating known regulators and identifying factors, such as *Pparg* and *Bhlhe40*, as part of the core regulatory network governing Th2 helper cell fates.

## Highlights

• A retroviral CRISPR sgRNA library enables screening in primary mouse T helper cells
• Genes frequently affect both T helper cell activation and differentiation
• Genes of all functional categories impact activation and differentiation
• The transcription factor PPARG appears particularly important for Th2 gene regulation

## Graphical Abstract

**Figure:**
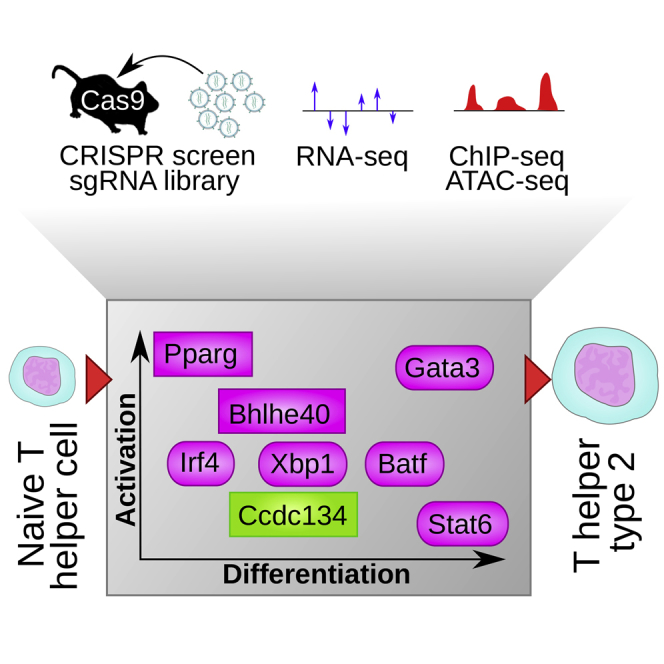

## Teaser

A CRISPR-based screen reveals gene networks central to modulating multiple aspects of Th2 cell function.

## Introduction

CD4+ T helper (Th) cells are a central part of the adaptive immune system. During immune response, Th cells transform from a naive state into different effector subtypes, including T helper type 1 cells (Th1), Th2, Th17, and regulatory T cells (Treg). Different subtypes have distinct functions and molecular characteristics (Zhu et al., 2010). Th2 cells are primarily involved in eliminating helminths and other parasites and are strongly associated with allergies.

Th2 differentiation is characterized by the production of the cytokines *Il4*, *Il5*, and *Il13*. *In vitro*, *Il4* is crucial for the activation of the signaling transducer *Stat6* (Kaplan et al., 1996, Chen et al., 2003, Elo et al., 2010), which induces the Th2 master regulator *Gata3* (Swain et al., 1990). *Gata3* activates *Il4*, forming a positive feedback loop (Zheng & Flavell 1997). Th1 cells possess an equivalent feedback mechanism for their defining transcription factor (TF), *Tbx21*, which represses *Gata3*. *Gata3* is able to inhibit *Ifng*, the main cytokine driving Th1 differentiation. Thus, the balance of the two TFs *Tbx21* and *Gata3* defines the Th1-Th2 axis (Kanhere et al., 2012). There are, however, many genes affecting this balance, and alternative Th fates are frequently affected by overlapping sets of regulatory genes. All T cell fates require activation via the T cell receptor and a co-stimulatory molecule, for example, CD28. Additional signaling via cytokines then determines the adapted T cell fate. Therefore, a delineation of activation versus differentiation is critical for our understanding of Th subtype development. Despite the importance of different T helper subtypes, so far only the Th17 subtype has been examined systematically (Ciofani et al., 2012). Here, we dissect Th2 differentiation with a special emphasis on differentiation versus activation signals.

A major challenge in performing genetic studies in primary mouse T cells is the lack of efficient genetic perturbation tools. To date, only a small-scale RNA interference screen has been performed *in vivo* on mouse T cells (Chen et al., 2014). However, recently developed CRISPR technology has the advantages of higher specificity and greater flexibility, allowing knockout, repression, and activation (Adli 2018). Currently, all existing CRISPR libraries are lentiviral-based and therefore unable to infect murine Th cells (Baumann et al., 2004). To overcome this limitation, we created a genome-wide retroviral CRISPR small guide RNA (sgRNA) library. By using this library on T cells from mice constitutively expressing *Cas9,* we obtained high knockout efficiency. In addition, we established an arrayed CRISPR screening protocol that is scalable and cost efficient.

After library transduction, we screened for and characterized genes strongly affecting Th2 differentiation and activation, with *Il4*, *Il13*, *Gata3*, *Irf4*, and *Xbp1* as our primary screen readouts. *Il4*, *Il13*, and *Gata3* are at the core of Th2 differentiation (Kanhere et al., 2012), while *Irf4* and *Xbp1* have been suggested to have supporting roles in keeping the chromatin accessible and in overcoming the stress response associated with rapid protein synthesis during T cell activation (Li et al., 2012, Kemp et al., 2013, Pramanik et al., 2018). *Gata3* is involved in both activation and differentiation, as mice deficient in *Gata3* are unable to generate single-positive CD4 T cells, which requires activation via the T cell receptor (TCR) (Pai et al., 2003). However, *Gata3* also has a well-established role in regulating the Th1 or Th2 differentiation axis. Selected genes discovered by the screen were validated in individual knockouts (KOs) and assayed by RNA sequencing (RNA-seq). To place the discovered genes into the context of Th2 differentiation, we profiled developing Th2 cells using RNA-seq for gene expression, ATAC-seq (assay for transposase-accessible chromatin using sequencing) for chromatin accessibility, and ChIP-seq (chromatin immunoprecipitation sequencing) of three key TFs: GATA3, IRF4, and BATF. We further acquired corresponding data from human donors to study the conservation of the regulatory pathways.

A genome-wide assessment of gene regulatory function was performed by combining state-of-the-art transcriptional gene regulatory network analysis, literature curation, and genome-wide screen enrichment. Selected hits were validated in individual KO and overexpression experiments. The function of key regulators of Th2 differentiation was further explored by performing additional ChIP-seq experiments. We characterize genes in terms of their impact on activation and differentiation and provide a comprehensive, multi-factor model for Th2 cell fate determination. For ease of visualization, the integrated dataset is provided online at http://www.teichlab.org/data/.

## Results and Discussion

### Genome-wide CRISPR/Cas9 Screens Reveal Genes Driving Primary Mouse Th2 Differentiation

**Figure 1:**
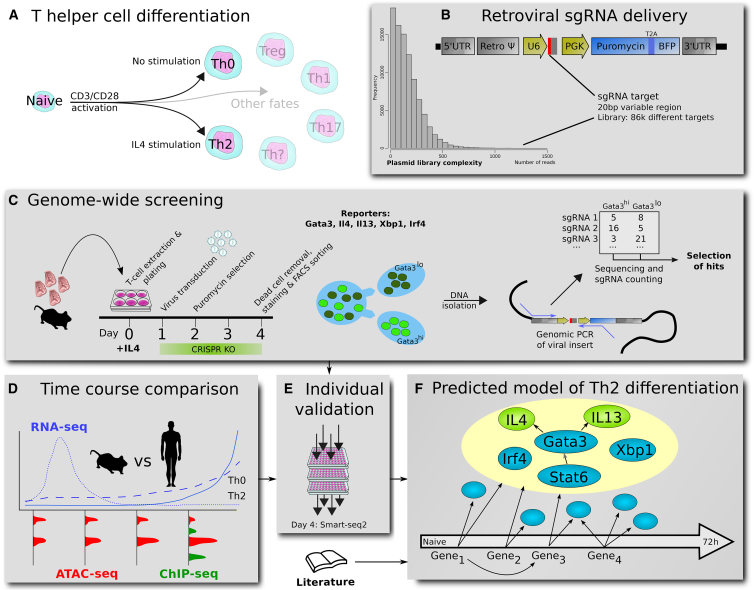
Overview of the Experimental KO Screening Strategy (A) In our culture system, naive, *ex vivo* T cells are differentiated into Th2 cells by IL4. Potential alternative T cell fates that may be open to genetically perturbed cells are indicated. *In vivo*, T cells develop into different subtypes dependent on stimuli. (B) The retrovirus is based on murine stem cell virus (MSCV), encoding one sgRNA per virus, and allows for BFP and puro selection. For the screening we have used a pool of plasmids, encoding over 86,000 sgRNAs, from all of which we produced viruses. The library is subcloned from a previous mouse sgRNA library (Tzelepis et al., 2016). (C) For genome-wide screens, we pool cells from up to 30 mice. After infection and puromycin selection, the cells are sorted based on fluorescence for the investigated gene. sgRNAs affecting gene expression are identified by genomic PCR. Differential sgRNA expression analysis then allows us to find genes affecting either viability (drop-out screen) or differentiation. (D) The top enriched and depleted genes (“hits”) were analyzed based on their dynamics measured by RNA-seq, ATAC-seq, and ChIP-seq. (E) Particularly interesting genes were further validated by individual KO and RNA-seq. (F) By using all this data and curating the literature, we provide a Th2 gene regulatory network.

Figure 1 depicts an overview of our experimental approach. First, a high-complexity retroviral sgRNA library was generated (Figure 1B). We activated naive CD4+ T cells, purified from mouse spleens, with anti-CD3 and anti-CD28 together with IL4 at day 0. On day 1, T cells were transduced with the retroviral libraries and selected with puromycin from day 3. After dead cell removal, the screens were carried out on day 4. A general protocol is supplied as Data S1.

Our screening strategy used two different approaches. For *Il4*, *Il13*, *Xbp1*, and *Gata3*, we used T cells from transgenic mice carrying a fluorescent reporter driven by the promoter of the respective genes. In this protocol, cell populations with high or low fluorescence were enriched with sgRNAs for genes inhibiting or promoting Th2 cell differentiation, respectively. In addition, we carried out screens in which T cells were stained with antibodies for IRF4, XBP1, or GATA3. Most CRISPR screens to date are “drop-out” screens where the sgRNAs from an early time point are compared to those in the final surviving cell population. In contrast, here we identify differentiation-related genes by comparing the sgRNAs in the selected target-high versus target-low fractions. We will refer to the most highly enriched or depleted genes (defined in more detail below) as “hits.”

**Figure 2:**
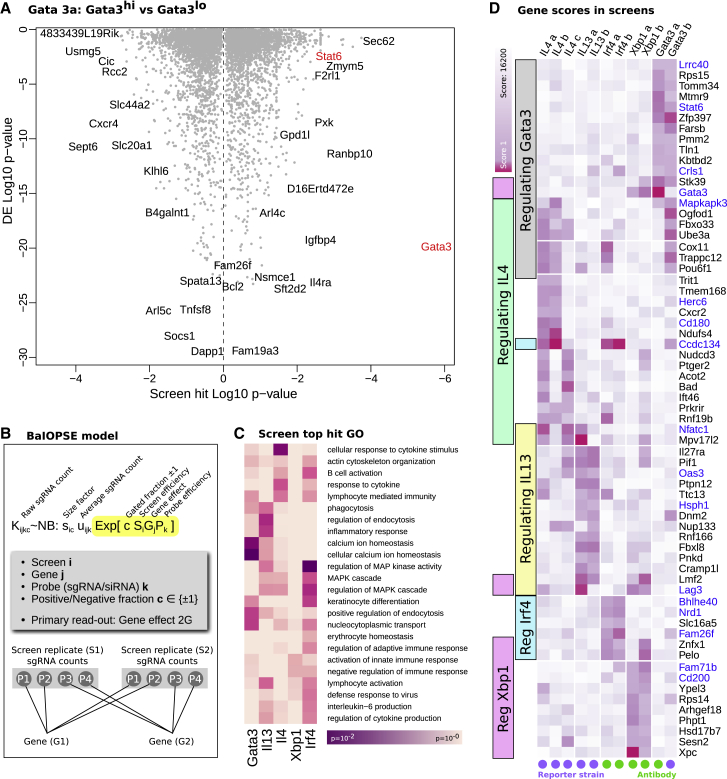
Results from Genome-wide Th2 Differentiation Screen (A) Hits from screen for *Gata3* expression measured by antibody staining. The x axis denotes the p value for differential expression obtained by MAGeCK (hits of high relevance toward both sides). The y axis shows the p value comparing Th2 and Th0 gene expression level (explained later). Highlighted in red are *Gata3* and *Stat6*, since these are known to control *Gata3* expression. (B) The alternative BaIOPSE (Bayesian inference of pooled screen enrichment) hit-calling model. This model is, in essence, an extended negative binomial differential expression model over sgRNA counts K. Each sgRNA has an efficiency term P, and each screen has an efficiency term S. The interesting readout is the gene effect 2G. (C) GO annotation of top hits for each screen as defined by BaIOPSE. The color represents log_10_ p value. (D) Summary results of all 11 screens carried out. Genes that were consistent hits in multiple screens are shown (see STAR Methods for gene selection). The purple color shows the log_10_ combined MAGeCK rank (positive and negative enrichment combined). Screens that relied on antibody staining are marked by a green circle, and those based on fluorescent gene reporters are marked by a purple circle. Genes in blue have been knocked out individually (see Figure 6).

In total, we carried out 11 genetic screens and analyzed these using the CRISPR screen hit-calling software MAGeCK (Li et al., 2014) and compared this method to an orthogonal hit-calling method BaIOPSE (Bayesian inference of pooled screen enrichment [Figure 2B], further described in Data S1). Qualitatively, we find that there is reasonable overlap between MAGeCK and BaIOPSE (BaIOPSE scores in Data S2). Figure 2A shows the hits in a screen using anti-Gata3 antibody staining (i.e., sgRNA for specific target genes), ranked by MAGeCK p value, against the fold change (Th2, 0 h versus 72 h, described later) of those sgRNA targeted genes. As expected, *Gata3* is recovered as a top hit in its own screen. Another top hit is a known signal transducer from the IL4 receptor to *Gata3*, the TF *Stat6*. Previous work has shown *Stat6* to be required for the majority of Th2 response genes in mouse and human (Chen et al., 2003, Elo et al., 2010). This gives us confidence that relevant genes are recovered. In a gene ontology (GO) analysis of top hits from all screens (Figure 2C), the categories for calcium and MAPK signaling have the lowest p values. While BaIOPSE allows a more consistent integration of multiple screen replicates than MAGeCK, we use MAGeCK for the remainder of this paper because of its pre-existing community acceptance and because BaIOPSE relies on informative priors.

In all subsequent descriptions of hits, we will refer to the expression of the targeted gene, rather than the level of sgRNA enrichment or depletion. For the sake of brevity, in this paper we will use the nomenclature X^→y^, when gene X is in the top 5% of hits in the screen Y, either positively or negatively enriched. If gene X falls within the top 1% of ranked hits, we denote this as X^→y!^. A comprehensive list of all gene hits is included in Data S2, and results are summarized in Figure 2D.

Next, we identified hits that were consistent between screens (see STAR Methods for details). Some genes appear to have a particularly strong impact on Th2 development as they are seen in multiple screens. Some affect both activation regulators (*Irf4*, *Xbp1*, *Gata3*) and differentiation regulators (*Gata3*, *Il4*, *Il13*). This includes the known genes *Il27ra*^→Il4,Il13!^ and *Lag3*^→Il4,Il13,Xbp1!^ but also genes not previously connected to T cells, e.g., *Trappc12*^→Il4!,Irf4,Gata3!^, *Mpv17l2*^→Il4!,Il13!,Xbp1^, and the TF *Pou6f1*^→Il4!,Gata3^. The cytokine-like gene *Ccdc134*^→Il4!,Irf4!,Gata3^ is also a major hit. It has so far received little attention in the literature but has been linked to arthritis (Xia et al., 2017) and shown to promote CD8+ T cell effector functions (Huang et al., 2014). In short, we have discovered many genes with a broad effect on Th2 differentiation and activation that deserve further investigation.

### Time Course Analysis of Gene Expression and Human-Mouse Comparison Highlight Metabolic Genes

**Figure 3:**
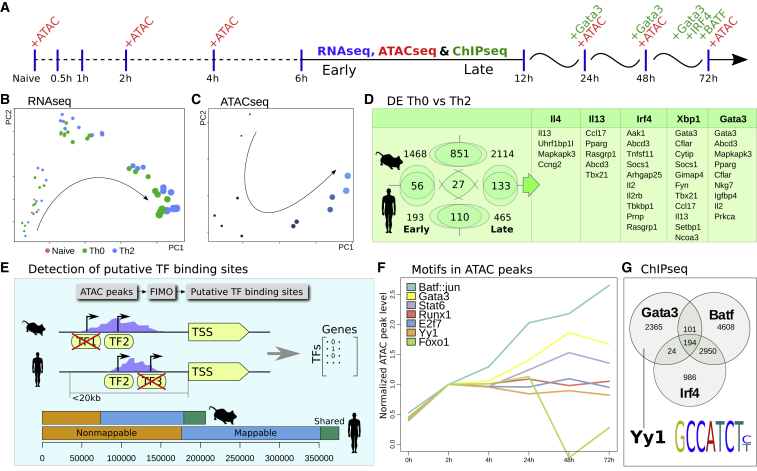
Molecular Characterization and Assessment of Hits over the Time Course of Th2 Differentiation (A) The chosen time points for RNA-seq, ATAC-seq, and ChIP-seq. (B and C) PCA projection of bulk RNA-seq (B) and ATAC-seq (C) samples. The size of the circle represents time. The naive samples separate in the third principal component not shown. (D) Number of differentially expressed genes in the early and late response, in human and mouse (p = 10^−4^). DE genes in both human and mouse that are also hits in the genetic screens sorted by rank in their respective screen. (E) Workflow for finding conserved putative TF-binding sites in human and mouse. The green region represents conserved (overlapping) peaks. The blue region represents peaks in regions with a corresponding sequence in the other species but without peak conservation. The orange region depicts peaks lying in non-syntenic (unmappable) regions. (F) Examples of ATAC-seq peak dynamics associated with different TFs. (G) Overlap of peaks in different ChIP-seq experiments at 72 h. We note the presence of the YY1 motif within the GATA3 peaks.

To place our hits into the context of Th2 development, we generated *in vitro* time course data during mouse and human Th cell activation and differentiation (Figure 3A). Mouse and human primary Th cells were isolated from spleen and cord blood, respectively, and activated with anti-CD3 and anti-CD28. Upon addition of IL4, these cells matured into Th2 cells, while absence of IL4 resulted in activated “Th0” cells, which proliferate but do not differentiate into a Th subtype. We performed time course bulk RNA-seq profiling on Th2 and Th0 and ATAC-seq at several time points during Th2 differentiation. The large number of data points allowed us to reconstruct the trajectory of Th2 differentiation by principal component analysis (PCA), using RNA-seq data or ATAC-seq data alone (Figures 3B and 3C).

When carrying out differential gene expression (DE) analysis between the murine Th0 and Th2 populations, we split the time course into the early or fine-grained (0–6 h) period and a late or coarse-grained period (0 h + 6–72 h), as shown in Figure 3A. The number of DE genes is shown in Figure 3D. Importantly, a sizeable fraction of these (21%) were also identified in at least one of our genetic screens, providing orthogonal evidence for their importance (DE scores are in Data S2).

We carried out an equivalent RNA-seq analysis across ten time points in cultured human primary T cells. Fewer DE genes were identified, possibly because genetic diversity between individuals may obscure some gene expression changes, but more than one-fifth of the human DE genes had direct orthologs in the mouse response (Figure 3E). We will refer to any gene being DE in either human or mouse, at any time, as simply DE.

A total of 216 genes were DE in both mouse and human, either early or late (p = 10^−4^). DE genes that also are top hits in our CRISPR screens are shown in Figure 3D. We note the presence of the well-known cytokines *Ccl17*^→Il4,Il13,Xbp1^, *Il13*^→Il4,Xbp1^, and *Il2*^→Irf4,Gata3^ and its receptor *Il2rb*^→Irf4^ and the TFs *Gata3*^→Xbp1!,Gata3!^, *Tbx21*^→Il13,Xbp1^, and *Pparg*^→Il13,Gata3^. Several of these are canonical Th2 genes, but many other genes were also noted. Several of these are related to metabolism, such as *Pparg*, which is thought to signal through mTOR (mechanistic target of rapamycin) and control fatty acid uptake (Angela et al., 2016). Another metabolic gene, related to fatty acid transport (Dean et al., 2001), with a strong phenotype in our screen is *Abcd3*^→Il13,Ir4,Gata3!^, which has not yet been studied in T cells. The Th1 repressor *Mapkapk3*^→Il4,Gata3!^ is also a metabolic gene (Köther et al., 2014).

Other hits have more diverse functions in T cell development. Hits include the known T cell regulator *Stat*-inhibitor *Socs1*^→Irf4,Xbp1^. The *Il13* hit *Rasgrp1*^→Il13,Irf4^ is known to be involved in T cell maturation (Priatel et al., 2002) and links guanyl to the RAS pathway. Interestingly the guanylate-binding protein, *Gbp4*^→Il13^, is also an Il13 hit (but with higher DE p value). The *Il4* candidate regulator *Uhrf1bp1l*^→Il4^ has been connected to hypomyelination but could act through the chromatin regulator *Uhrf1*, which is required for Treg maturation (Obata et al., 2014).

In conclusion, a human-mouse comparison of DE genes highlights cytokines and TFs known to be important for both Th2 activation and differentiation and suggests additional hits in our screens that are likely to be of functional importance, in particular genes that act as metabolic regulators (e.g., *Abcd3*).

### Analysis of Chromatin Dynamics Reveals Different TF Binding Patterns during Activation and Differentiation

To gain further insight into the regulation of gene expression, we examined chromatin accessibility using ATAC-seq. We performed ATAC-seq of developing Th2 cells at 0, 2, 4 24, 48, and 72 h time points in both human and mouse (Figure 3A). The chromatin of naive T cells is condensed until activation. It has previously been shown that some TFs, for example, *Stat5,* can only access the promoters of its target genes after T cell activation (Rawlings et al., 2011). Th2 differentiation is classically thought to be driven by *Stat6*, which in turn upregulates *Gata3*. We examined these dynamics over the time course of the Th2 response.

**Figure S1:**
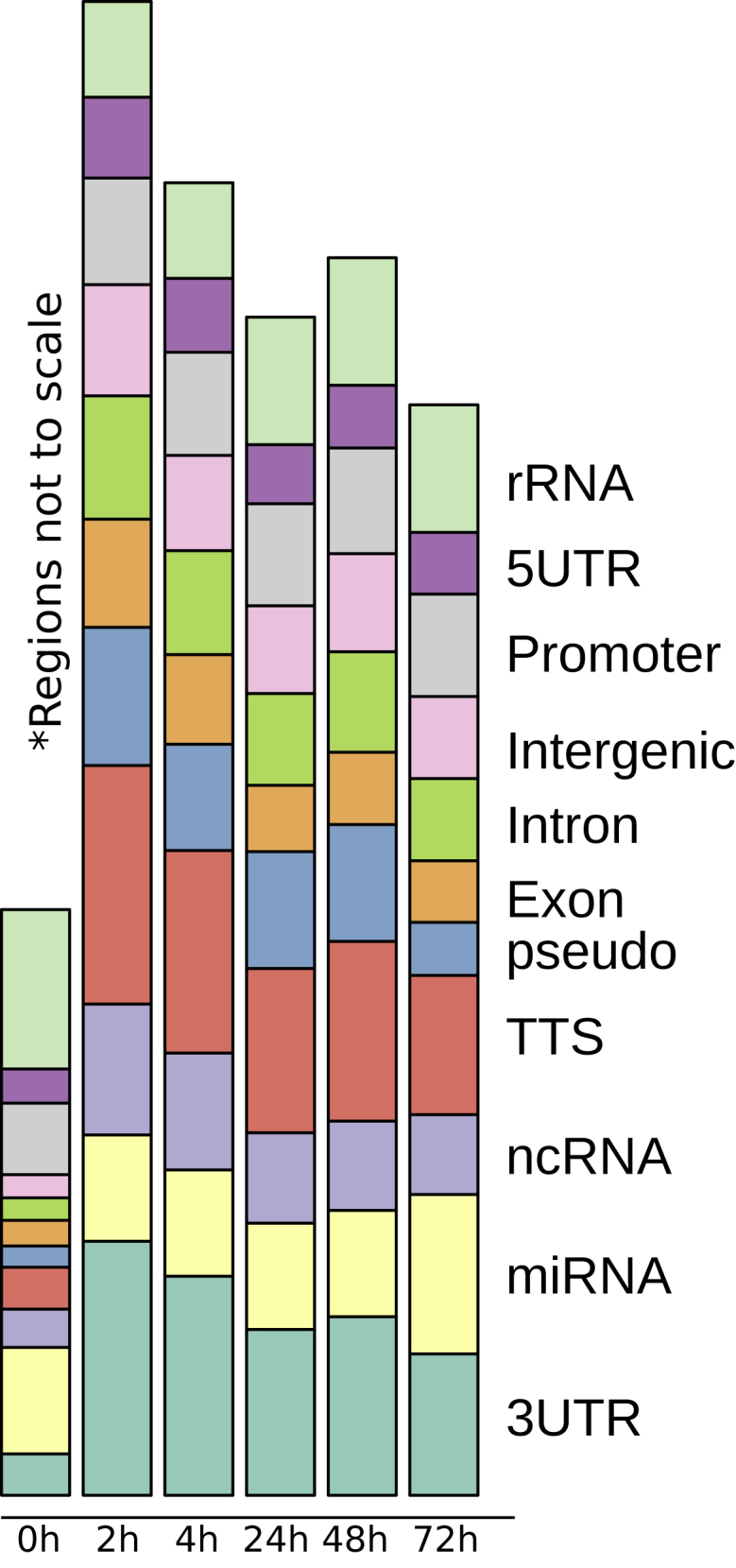
Alternative Analysis of Chromatin Opening, Related to Figure 3G Number of detected ATAC peaks at different time points (union over replicates), broken down into categories as reported by MACS2. Smaller categories have been amplified in size to highlight their dynamics over time.

The ATAC peaks were first called using MACS2. Overall, there is a massive gain of chromatin accessibility from 0 to 2 h (Figure S1). After this initial opening, the chromatin appears to recondense continuously, as indicated by the reduced total number of ATAC-seq peaks at each successive time point. We speculate that the regulatory network shifts from a general T cell network to subtype-specific network and that cell identity becomes less plastic and less responsive to external perturbation over time.

We next compared TF binding predictions between human and mouse. Using FIMO (find individual motif occurrences), we predicted TF-binding sites within ATAC-seq peaks. To reduce the number of potential false-positive peaks, we concentrated on ATAC peaks that are conserved between mouse and human by calculating the percentage of overlapping peaks between species (10%–15%) (Figure 3E) and used these conserved binding sites for the rest of the analysis.

**Figure S2:**
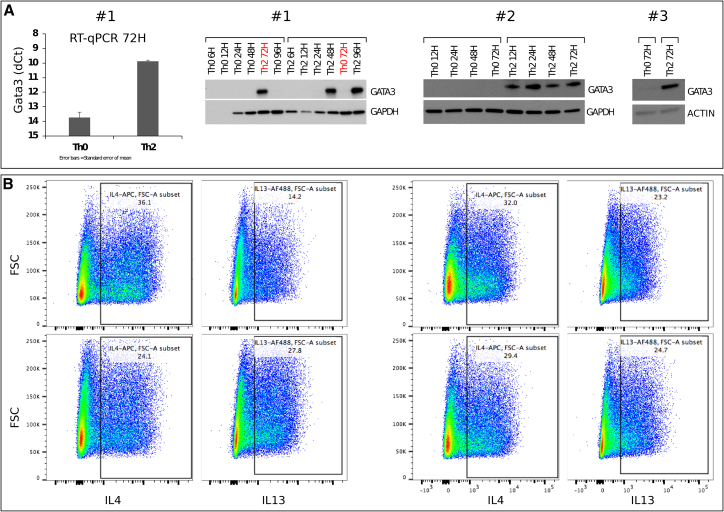
Validation of Differentiation Efficiency, Related to STAR Methods (A) Western blot and RT-qPCR to check for presence of GATA3 in human T cells stimulated by IL4, in 3 replicates. (B) Staining with anti-IL4 and anti-IL13 and anti-GATA3 at day 8 in mouse T cells, in 4 replicates, following a previous method (Pramanik et al., 2018).

For different TFs, we examined how ATAC peaks, in which the relevant TF motif is found, are changing over time (Figure 3F). As expected for Th2, chromatin accessibility over GATA3 motifs increases strongly with time, correlating with the increase in GATA3 abundance (confirmed by western blot [Figure S2] and RNA-seq [Data S2]). However, the (composite) motif that is most associated with relative peak size increase is BATF::JUN. This is consistent with the suggestion that BATF can act as a pioneer factor to open chromatin (Ciofani et al., 2012). The functional importance of *Baft* and/or *Jun* is supported by our genetic screens: *Jun*^→Il13^, *Fos*^→Irf4,Xbp1^, and *Fosl2*^→Gata3!^ are all associated with increasing peak height. Since Jun and/or Fos and Fosl2 all recognize the same AP-1 motif, the exact TF composition at these peaks is likely to depend on their expression level. Notably, *Fosl2* expression is highest at the time points of 1 and 2 h in Th0 or Th2, with largely similar levels across Th1, Th2, Th17, or Treg subtypes (Stubbington et al., 2015). Overexpression of *Fosl2* has been shown to block IL17A production in Th17 by competing for AP-1 sites (Ciofani et al., 2012), but overall *Fosl2* expression is low in lymphoid cells (Uhlén et al., 2015). *Fos* and *Jun* are transiently expressed during the first 6 h. *Jund,* another classical AP-1 factor, displays slowly increasing expression over time. As most AP-1 factors are expressed at low levels, *Batf*, whose expression increases continuously, is the most likely driver behind these peaks.

At the other extreme, some TF motifs are overrepresented in peaks that decrease over time, such as *Hoxd9*^→Il4^, *Atf3*^→Gata3^, *Atf4*^→Il4!^, *Foxj2*^→Gata3^, *Dmbx1*^→Irf4^, *Foxa2*^→Il4!^, *Foxo3*^→Il4^, and *Foxc2*^→Il13!^. Several of these TFs also have low or decreasing expression levels. We have previously shown that *Atf3*^→Gata3^ positively regulates *Ifng* (Filén et al., 2010) and promotes Th1 differentiation in humans. *Atf4*^→Il4!^ has been shown to be important for Th1 function as stress regulator (Xia et al., 2015), but its impact on *Il4* extends this claim to Th2. *Foxo1*^→Il13!,Xbp1!^ is a highly expressed TF, but peaks containing this motif are also decaying. *Foxo1* has recently been shown to inhibit H3K27me3 deposition at pro-memory T cell genes (Gray et al., 2017). *Foxj2* has similar behavior to *Foxo1* but has not been studied in T cells.

Inferred STAT6-binding sites were also compared with previous mouse and human data (Elo et al., 2010, Chen et al., 2003), and we found that the vast majority of the previous target genes are also DE in our time course analysis. A list of all TFs and the average height of peaks containing their cognate motif is provided in Data S2.

**Figure S3:**
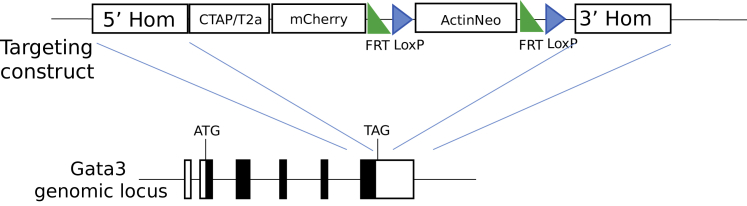
Design of the GATA3-3xFLAG-mCherry Mouse Strain, Related to STAR Methods A sequence of 3xFLAG-T2A-GFP is inserted after the last exon of the endogenous *Gata3* as described in methods.

To further characterize the dynamics of the Th2 response, we generated ChIP-seq data at several time points (Figure 3A) for the Th2 master regulator GATA3, as well as BATF and IRF4. We created a mouse strain with a 3xFLAG-mCherry GATA3 construct (T2A fusion; Figure S3) for this purpose (see STAR Methods for details). The ChIP-seq peaks for *Batf* and *Irf4* have a large overlap as previously reported (Ciofani et al., 2012) (Figure 3G) (Jaccard index = 0.35). However, we saw no significant overlap of these two factors with GATA3 (Jaccard index = 0.028 and 0.032), suggesting that any synergistic function between GATA3 and BATF and/or IRF4 is not due to direct protein-protein contact. MEME (multiple EM for motif elicitation) was applied to the sequences in the GATA3 peaks to find other potential binding partners, and we found enrichment of YY1^→Il4,Il13,Xbp1,Gata3^ (p = 2.5 × 10^−58^) binding motifs. This is consistent with previous reports that *Yy1* is required but not sufficient for Th2 cytokine expression (Hwang et al., 2013). Indeed, ATAC-seq peaks containing the YY1 motif are stable or decrease slightly (Figure 3f). This finding, together with the identification of *Yy1* as a strong hit in our screen, reiterates *Yy1* as a key supporter, but not driver, of Th2 differentiation.

Focusing on GATA3 with its 10,203 peaks, a GO term analysis of its nearby genes yielded “natural killer cell activation” (p = 6 × 10^−3^) but included few other immune-related terms. This is likely due to the fact that *Gata3* has distinct roles in other cell types (Wei et al., 2011, Van de Walle et al., 2016) (a survey has shown that its expression is highest in breast cancer cell lines [Uhlén et al., 2015]). Since we performed time course ChIP-seq, we were able to selectively investigate peaks based on their dynamics. We calculated the ChIP peak height ratio at 72 h versus 24 h and defined the most increasing or decreasing GATA3 peaks as the top and bottom 1,000 peaks ordered by ratio. Genes near peaks decreasing over time were not linked to any particular immune-related GO terms, but a GO term enrichment for genes near increasing peaks revealed “defense to bacterium” or ”viral life cycle” (p = 5 × 10^−3^) as the top term and included other terms such as “myeloid leukocyte activation” (p = 2 × 10^−2^). A ranking of peaks and nearby genes, as well as GO terms, are provided in Data S2.

Overall, the early change in ATAC-seq peak size reflects a rapid increase in accessibility for all TFs, that is further increased for specific Th2-related TFs (e.g., *Batf/Jun*, *Gata3*), followed by a progressive loss of peaks as the cells differentiate. Interestingly, our screen hits were found in both categories of TFs and may be functionally important as either activators or repressors for the specific T helper type.

### Motif Activity Analysis Quantifies Transcription Factors Controlling Activation versus Differentiation

**Figure 4:**
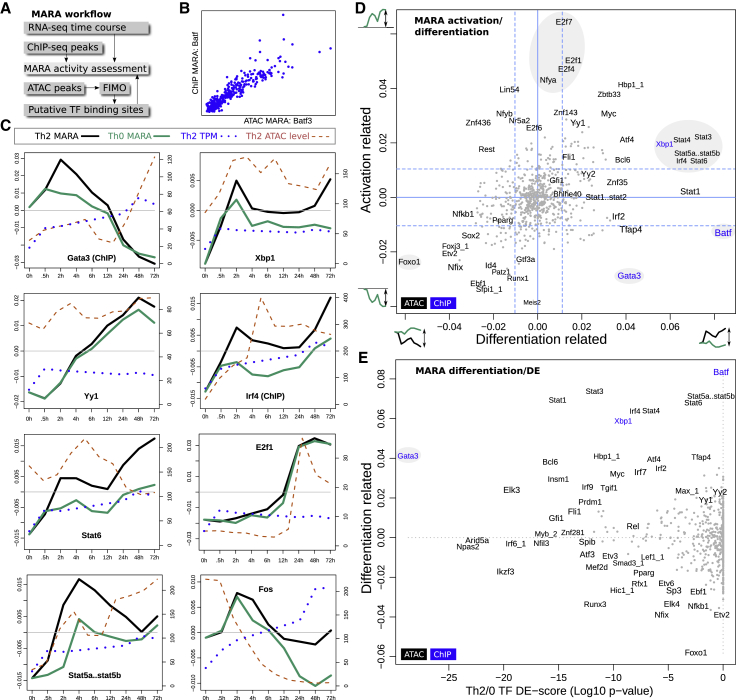
Analysis of TF Activity Using MARA (A) Workflow for combining putative binding sites with time course RNA-seq. (B) Comparison of BATF activity predictions for individual genes by ATAC-seq predicted binding sites and ChIP-seq peaks. (C) Dynamics of selected TFs, comparing their expression level, activity in Th2 (black line) and Th0 (green line), and chromatin accessibility. (D) MARA activation versus differentiation scores (as defined in text) of all TFs. (E) Comparison of differentiation score and DE p value Th2 versus Th0.

To gain a broader perspective on how genes affect differentiation and activation, we chose to perform a network analysis to compare their downstream effects. For this purpose, we used the ISMARA (Balwierz et al., 2014) algorithm, which builds a network by linking TFs to potential target genes based on the presence of the relevant motif in an ATAC-seq peak within the vicinity of the transcription start site (TSS) of that target gene (Figure 4A). In short, a TF has a high motif activity response analysis (MARA) activity score if the TF consistently explains the upregulation of all its putative downstream genes (or negative score, if it is a suppressor). Interestingly, we found a very high correlation in the predicted networks using ATAC-seq and ChIP-seq data (Figure 4B), suggesting that the algorithm performs well on ATAC-seq input data, allowing us to analyze many TFs besides those with ChIP-seq data. It should be noted that this method struggles to separate TFs with highly similar binding motifs (such as most STAT proteins) and may underestimate the activity of TFs with degenerate motifs. In our interpretation, we associate motifs with the most likely target gene based on the literature, hit score, and expression level in our RNA-seq data.

To obtain an overview of the role of all TFs, we categorized TFs according to their activity over time within the Th2 differentiation pathway and whether their activity differs between Th2 and Th0 cells. In other words, two distinct comparisons are made: first t = 0 h versus t = 72 h within the Th0 compartment, which we term “activation,” and second, Th0 versus Th2 cells at t = 72 h, which we term “differentiation.” Figure 4C illustrates this analysis by showing MARA activity scores independently calculated for Th2 and Th0 cells for a number of selected TFs. An example of a TF strongly associated with differentiation (i.e., large difference between black and green lines) is *Fos*^→Irf4,Xbp1^, while an activation phenotype, reflected in a large difference between t = 0 h and t = 72 h, is observed for *E2f1*^→Irf4^.

The majority of TFs display a behavior reflecting both activation and differentiation (Figure 4D). We note that for many TFs the activity score does not reflect an increase in expression. Indeed, this is a key strength of the MARA analysis, which calculates a score based on the activity of downstream target genes and can therefore reflect post-transcriptional regulation or protein-protein interactions affecting TF activity (individual genes in Figure 4C, comparison to differential expression in Figure 4E). An example of this is the Th2-defining TF *Gata3*^→Gata3!,Xbp1!^, which shows a transient increase in activity, yet its expression levels continually increase with time. *Gata3* is one of the strongest mediators of both activation and differentiation, although its differentiation activity appears to be exerted early. *Stat6*^→Gata3!^ is also thought to act early in differentiation, after its activation via the Il4 receptor *Il4ra*^→Gata3^. We previously showed that, during Th2 differentiation, signals from IL4R are predominantly transduced through STAT6 (Elo et al., 2010). Consistent with those findings, our data suggest that *Stat6* activity continues to increase throughout differentiation. Interestingly all the STAT proteins map closely together in Figure Dd (gray circle), affecting primarily differentiation but also activation, possibly all contributing to different extents depending on their expression, phosphorylation status, and interactions with other proteins and regulatory elements. *Irf4* is also in this cluster (Figure 4D). *Foxo1*^→Il13,Xbp1^ and *Xbp1*^→Il4^ are also strongly connected to activation and differentiation but with *Foxo1* and *Xbp1* having effects in the opposite direction. Previous work suggested that the primary role of *Batf* is to open the chromatin together with *Irf4* (Ciofani et al., 2012), and this is consistent with our analysis in Figure 3F. Here, *Batf* is one of the strongest differentiators, suggesting that chromatin opening is restricted to sites required for differentiation.

The roles of other genes are less clear. TFs that were identified as hits include *Atf4*^→Il4!^ (Xia et al., 2015) and *Yy2*^→Gata3^, *Id4*^→Il13,(Il4),Xbp1^, *Ebf1*^→Irf4^, *Foxp2*^→Gata3^, *Yy1*^→Il4,Il13,Xbp1,Gata3^, and *Fli1*^→Il4!^ affecting both activation and differentiation but with weaker effects. The identification of a cluster of E2F-proteins as strongly and purely activation-related is consistent with their role in cell-cycle control.

**Figure S4:**
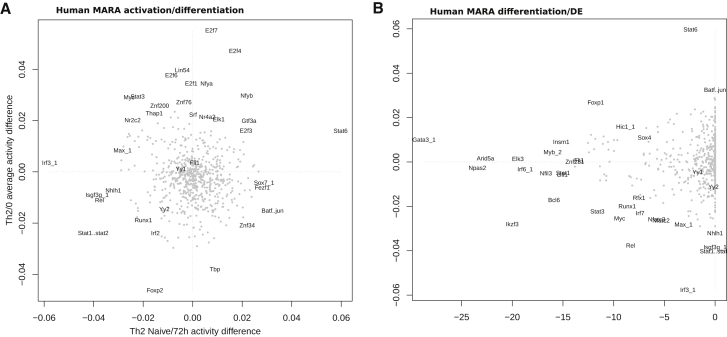
MARA Analysis of TF Activity in Human, Related to Figures 4D and 4E (A) MARA activation versus differentiation scores (as defined in text) of all TFs. (B) Comparison of differentiation score and DE *p*-value Th2 versus Th0.

The MARA approach allowed us to extract canonical Th2 TFs, such as *Stat6*, *Gata3*, and *Batf*, and in addition highlighted TF hits (*E2f1*, *Foxo1*) that are also likely to be relevant for Th2 development. Similar results hold also when applied to the human time course data (Figure S4). Since MARA is not directly dependent on TF target gene co-variation, the output is complementary to the previous DE approach. This analysis reinforces the notion that many TFs are involved in both activation and differentiation, with *Gata3* being a notable example consistent with published literature.

### Validation of Hits by Individual CRISPR KO to Assess Activation versus Differentiation

Next, we used the results described so far, related these to the existing literature, and chose a panel of 45 genes (40 by scores across all the screens and 5 controls), which were then validated by individual CRISPR KO. Several of the chosen genes have been studied before though not specifically in T cells. Our selection of interesting genes for further characterization is by no means comprehensive, and additional genes can be found by browsing our online resource.

**Figure 5:**
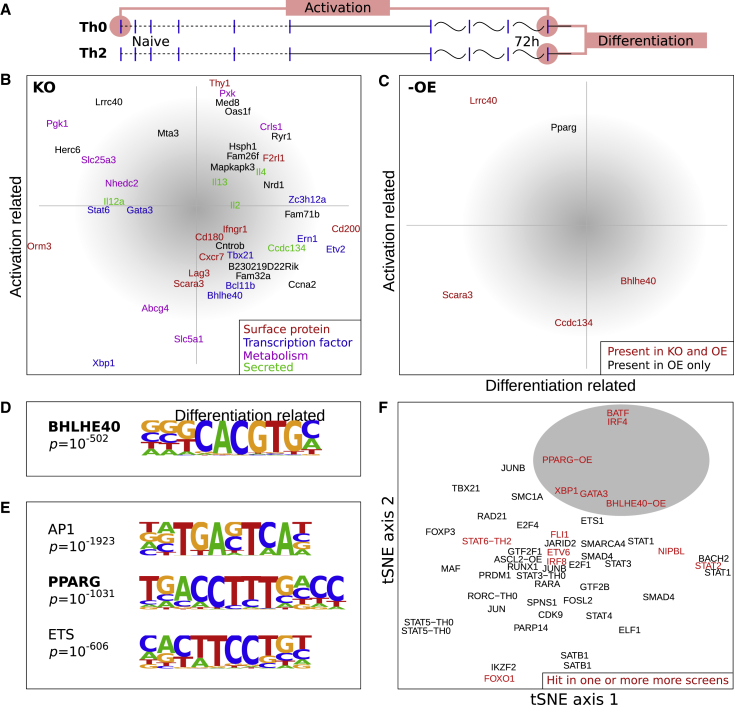
KO and Overexpression Effect on Activation and Differentiation for Key Hits (A) Axes representing activation and differentiation were defined in an unbiased way as the fold changes of DE genes from the RNA-seq time course, with activation as Th0 (t = 0) versus Th0 (t = 72 h) and differentiation as Th2 versus Th0 (t = 72 h). (B) The effect of gene KOs were quantified as the DE genes between KO and WT. These DE genes were then projected onto the axes representing activation and differentiation. Thus genes/KOs toward the middle of the plot have the least effect. (C) Verification of the KO effect by overexpression. The same projection onto the activation and differentiation axes. To facilitate comparison, the axes have been flipped, thus genes should appear in the same position as in the KO analysis. There is a qualitative agreement between KO and OE. (D) The motif found under peaks after overexpression and ChIP of BHLHE40. (E) Motifs found under peaks after overexpression and ChIP of PPARG. The most significant motifs are listed here. Further motifs are shown in Figure S5. (F) t-SNE (t-distributed stochastic neighbor embedding) clustering of TFs based on their nearest genes from the ChIP-seq peaks. A cluster of TFs (gray) contains the screened genes and hits that we have validated.

For each KO, cells were grown under Th2 differentiation conditions, and RNA-seq was carried out on day 4. For each gene, a DE list of KO versus non-targeting control was derived and compared to the activation and differentiation axes. As before, we defined the activation axis as the DE genes from 72 h versus 0 h under Th0 culture condition and the differentiation axis as the DE genes from Th0 versus Th2 at the 72 h time point (Figure 3A). It should be noted that some genes might not be consistently higher or lower in Th2 versus Th0 cells over time. To identify whether a KO aligns with one of these axes, we determined the projection of the DE genes of the particular KO to the aforementioned axes (Figure 5A; see STAR Methods for further details). Figure 5B shows that all genes tested map away from the neutral center of the plot (shaded in gray), indicating that the hits are weighted to contribute slightly more strongly to either differentiation or activation. In the KO analysis, *Il4* shows little effect, which we believe is due to IL4 being supplemented in the media.

Consistent with the MARA analysis, *Stat6* is primarily driving differentiation. By basing this analysis just on expression, *Gata3* now appears to be primarily driving activation, while in MARA it is also controlling differentiation. For TFs, the MARA analysis uses only the expression values of genes that are bound by the relevant TF and is therefore likely to be more accurate whenever the two analyses diverge.

The majority of KO genes affect both differentiation and activation to some degree. Examples of interesting genes that have not been studied extensively before in T cells are *Pgk1*^→Il4^, *Lrrc40*^→Gata3^, *Slc25a3*^→Irf4^, and *Ccdc134*^→Il4!,Irf4!^.

### Important Transcription Factors Revealed through Overexpression and ChIP-Seq Validation

To further validate the function and gain mechanistic insights for some of the genes identified in the screen, we performed overexpression by cloning the coding sequence of *Bhlhe40*, *Pparg*, *Ccdc134*, *Gata3*, *Lrrc40*, and *Scara3* into the MSCV-gene-IRES-BFP (murine stem cell virus-gene-internal ribosome entry site-blue fluorescent protein) vector. We performed individual transduction and RNA-seq; individual DE genes are listed in the Data S2. To summarize the data and allow comparison to the knockout experiments, we repeated the activation-differentiation analysis (Figure 5C). Since overexpression is approximately the opposite of KO, the sign of the axis in this panel has been reversed for easy comparability. Qualitatively, we find agreement between KO and overexpression.

The previously unpublished *Lrrc40*^→Gata3^ is in particularly good agreement with the KO analysis. We found that it upregulates *Il4* (p = 2 × 10^−17^) and *Il5* (p = 2 × 10^−11^), supporting its role in differentiation. It also regulates *Igfbp4*^→Gata3^ (p = 7 × 10^−17^). Overexpression of *Igfbp4* has been shown to inhibit the growth of the thymus (Zhou et al., 2004), which is the same phenotype as observed in *Gata3* KO mice, suggesting a link *Lrrc40→Igfbp4→Gata3*. The molecular function of *Lrrc40* is unknown. It is present in all cell types and is expressed at the same level across CD4 T cell types. The presence of leucine-rich repeats (LRR), shared with the Toll-like receptor, points to a function in the innate immune system^→Irf4^ (Sun et al., 2018). Based on literature, *Lrrc40* may regulate cell volume (Kasuya et al., 2018) or Ca^2+^ channels (Yang et al., 2017).

**Figure S5:**
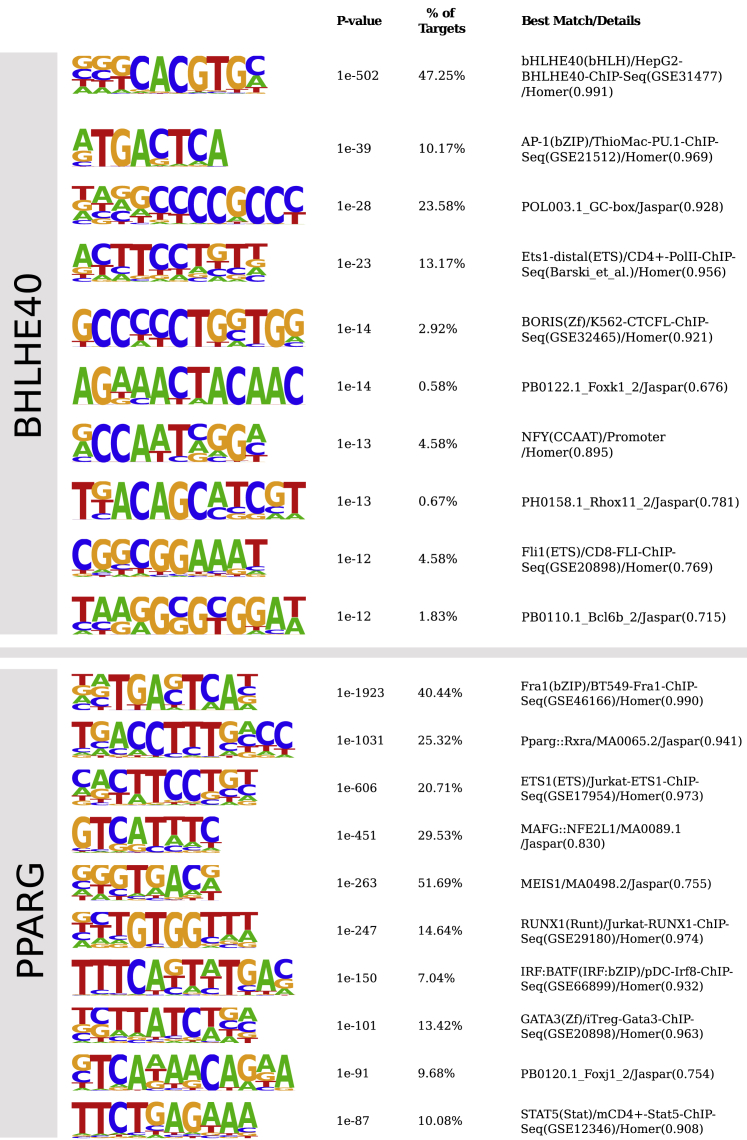
HOMER *De Novo* Motif Discovery in 3xFLAG Overexpression ChIP-Seq, Related to Figure 5 Top: motifs found under peaks after BHLHE40 ChIP: BHLHE40 is the only highly significant motif. Bottom: Motifs found under peaks after PPARG ChIP: Several highly significant motifs exist for PPARG, with the top motif being of the AP-1 family (*Fra1* is also known as *Fosl1*). As expected from comparison with other ChIP-seq datasets, the BATF:IRF4 and GATA3 motifs are also present.

While overexpression experiments validated our hits, we wished to gain further insight into the mechanisms by which some of the validated genes function. To this end, we added 3xFLAG tag at the 5′ end of the two TFs, *Bhlhe40* and *Pparg*, to allow us to find their direct targets by ChIP-seq using a FLAG antibody. Using this method, we analyzed the genome-wide binding events of *Bhlhe40* as well as *Pparg*^→Il13,Gata3^. For both *Bhlhe40* and *Pparg*, we found the expected motifs to be highly enriched (Figures 5D and 5E). Under the PPARG peaks, we found a strong enrichment of several other motifs (listed in Figure 5E; full list in Figure S5), including AP1, ETS1, RUNX1, IRF:BATF, GATA3, and STAT5.

The identification of motifs for known T cell program-related genes prompted us to extend our analysis. We compared the 3xFLAG ChIP-seq to our endogenous GATA3/IRF4/BATF ChIP-seq, and all the previously published relevant T cell ChIP-seq datasets (TFs and other DNA-binding proteins, see STAR Methods). When clustering the TFs based on their ChIP target genes (Figure 5F), GATA3, PPARG, BARF, and IRF4 appear in one cluster, and this holds true for a range of comparison methods (e.g., Pearson correlation, Jaccard index, among others, with and without normalizing for the number of peaks, data not shown). This may explain why *Pparg* is one of the most significant hits in our CRISPR screen: After *Stat6* and *Gata3*, it has the highest *Gata3* upstream screen score of all TFs. *Xbp1* and *Bhlhe40* cluster near *Gata3*. The close relationship of *Bhlhe40* and *Pparg* with canonical Th2 regulators identified these two TFs as members of the core Th2 regulatory network.

**Figure 6:**
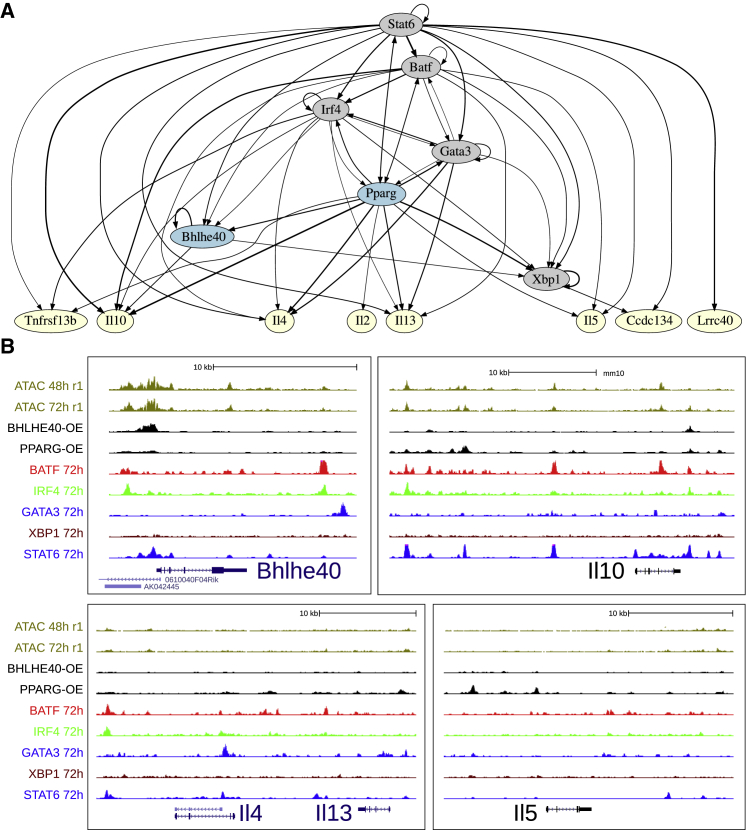
Validation of the Th2 Differentiation TF Network by ChIP-Seq (A) Network of Th2 TFs based on ChIP-seq peaks. From our validation data, the characterized TFs *Bhlhe40* and *Pparg* are highlighted. *Batf*, *Irf4*, *Pparg*, and *Gata3* cluster together as in the Figure 5 t-SNE. A network of genes especially representing the activation axis is shown in Figure S6. (B) University of California, Santa Cruz genome browser screenshots of ChIP-seq and ATAC-seq, highlighting the validated TFs for key Th2 genes.

**Figure S6:**
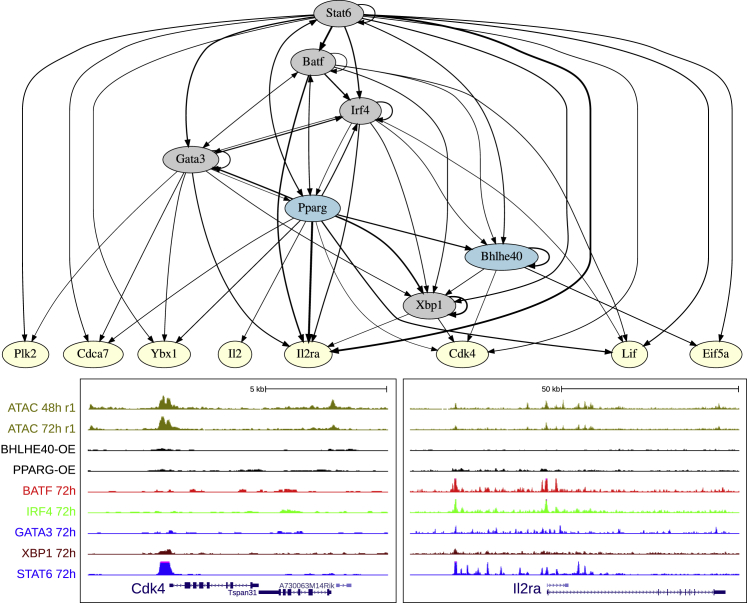
TF Network of Th2 Differentiation Based on ChIP-Seq Data, Related to Figure 6 (A) Network of Th2 transcription factors based on ChIP-seq peaks, focused on manually curated genes that are DE in activation. (B) UCSC genome browser screenshots of ChIP-seq and ATAC-seq, focusing on two examples of activation related genes.

Interestingly, STAT6 is separated from the GATA3 cluster. To understand why, we looked closer at how the main Th2 TFs connect together. A graph of TF connectivity is shown in Figure 6A, where ChIP peaks were associated with their closest genes whenever their distance to the TSS was less than 20 kb. Other metrics gave a similar result (data not shown). As in Figure 5F, PPARG-IRF4-BATF-GATA3 form a very tight cluster of TFs that regulate each other and share target genes. STAT6, which controls the Th2 program after input from IL4R, feeds into this program but also directly regulates the downstream cytokines. BHLHE40 and XBP1 are connected but mainly reside downstream of the other core TFs. A complementary network, focusing on some of the most differentially expressed activation marker genes, is provided in Figure S6.

We next focused on genes with consistent overexpression data and KO data (i.e., opposite direction in fold change), which were also identified as ChIP-seq direct targets (annotated ChIP-seq peaks are in Data S2). *Bhlhe40*^→Irf4^ has been shown to suppress inflammation through *Il10* (Huynh et al., 2018), which our data supports (direct target, Figure 6B, DE p = 0.015). In another model, ChIP-PCR in iNKT of *Bhlhe40* has shown that BHLHE40 binds near the *Ifng* locus and that binding is facilitated by *Tbx21* (Kanda et al., 2016). However, we do not see any peak near *Ifng* in Th2, despite overexpression, nor does DE analysis suggest any effect. Our data suggest alternative mechanisms. For example, *Tnfrsf13b*^→Il13,Irf4^ and *Tnfsf13b* are both DE, in opposite directions, with *Tnfrsf13b* a direct target. A peak and weak downregulation (p = 0.015), however, supports *Bhlhe40* as a negative regulator of inflammation through *Il10* (Yu et al., 2018).

*Pparg*^→Il13,Gata3^ has recently been shown to be essential for Th2 development (Chen et al., 2017), which our screen confirms. *Il5* (p = 10^−14^), *Il4* (p = 2 × 10^−6^) are highly DE direct targets (Figure 6b). This fits with previous experiments as PPAR family members have been noted to influence T cell activation and differentiation (Choi and Bothwell 2012).

To conclude, we have investigated several genes individually by overexpression and mapped their impact on activation and differentiation. We show that two upstream TFs, *Bhlhe40* and *Pparg*, functionally overlap with the central Th2 genes GATA3, BATF, and IRF4.

### Conclusions

In this study, we demonstrated, for the first time, the applicability of CRISPR to primary murine T cells. By carrying out *in vitro* genome-wide screens, we have created a resource of genes important for Th2 helper cell differentiation. We provide optimized protocols for performing additional screens as well as individual KOs. In our analysis, we chose five different readouts (*Gata3*, *Il4*, *Il13*, *Xbp1*, and *Irf4*), which represent Th2 differentiation and/or activation.

**Figure 7:**
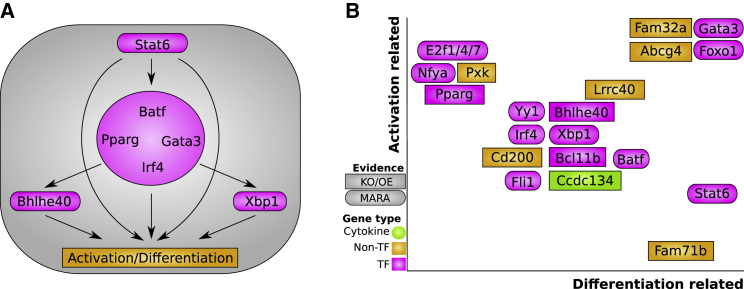
A Conceptual View of Th2 Differentiation (A) A broad overview of the core TFs. *Pparg* is closely integrated in the core program, while *Stat6* is the Th2 entry point that both goes through the core program and also connects directly to the cytokines. Several TFs work downstream. (B) While the genes controlling Th2 fate are from a wide range of programs, their mechanisms can be categorized into the modes of activation and differentiation. Here, we display a qualitative summary of key genes according to these categories based on our MARA, CRISPR knockout, ChIP-seq, and overexpression analyses.

Our unbiased approach to discover Th2 regulators show that the identified hits belong to many different classes of proteins, including cytokines, TFs, proteins involved in calcium signaling, and metabolic genes. We performed regulatory network analysis (MARA) to obtain deeper insights into the upstream genes and observed that many regulatory genes are involved in both differentiation and activation. A summary of this analysis and our validation experiments is shown in Figure 7.

Our analysis of Th2 differentiation has allowed us to add to the list of known regulators, such as *Gata3* or *Stat6*, a number of poorly or not previously examined genes. Among TFs, examples include *Foxo1*^→Il13,Xbp1^, *Bcl11b*^→Il13!^, *Pparg*^→Il13,Gata3^, and *Bhlhe40*^→Irf4^ (Figure 6B). Non-TFs have also been highlighted, including the cytokine *Ccdc134*^→Il4!,Irf4!,Gata3^.

For 45 genes, we also generated specific knockouts and analyzed overexpression and validated their impact on differentiation and activation through RNA-seq. *Bhlhe40*^→Irf4^ and *Pparg*^→Il13,Gata3^ were studied further through ChIP-seq, indicating that *Pparg* is particularly central to the Th2 program.

Our results yield further insights into genes involved in T helper cell differentiation that deserve further analysis. We also provide an efficient protocol for CRISPR-mediated KO. Both of these resources are key tools that will enable a more complete understanding of T helper cell biology. By combining our CRISPR KO screen with time course data, ChIP-seq, and overexpression, we have been able to provide a comprehensive map of the most important genes for Th2 differentiation and activation. These genes, along with their expression dynamics and chromatin accessibility, can be browsed on our supplemental website, http://www.teichlab.org/data/.

## STAR★Methods

### Key Resources Table

**Table:** 

REAGENT or RESOURCE	SOURCE	IDENTIFIER
**Antibodies**		

Anti-Cas9	BioLegend	Cat#844301, Clone #7A9
Anti-FLAG	Sigma M2	Cat#F3165
Anti-Human GATA3	BD Pharmingen	Cat#558686
Anti-Human GAPDH	Hytest	Cat#5G4MAB6C5
Anti-Human ACTIN	Sigma	Cat#A5441
Anti-Human CD3	Beckman Coulter	Cat#IM1304
Anti-Human CD28	Beckman Coulter	Cat#IM1376
Anti-Mouse CD3e	eBioscience	Cat#16-0031-81
Anti-Mouse CD28	eBioscience	Cat#16-0281-86
Anti-Mouse XBP-1S PE	BD Biosciences	Cat#562642
Anti-Mouse IRF4 FITC	BD Biosciences	Cat#11-9858-80
Anti-Mouse GATA3 Alexa Fluor 488	BD Biosciences	Cat#560077
Anti-Mouse IL4	eBioscience	Clone #11B11
Anti-Mouse IL13	eBioscience	Clone #eBio13A
Anti-Mouse CD4	eBioscience	Clone #GK1.5
Anti-Mouse CD4	eBioscience	Clone #RM4-5
Anti-Mouse IRF4	Santa Cruz Biotechnology	Cat#sc-6059
Anti-Mouse BATF	Santa Cruz Biotechnology	Cat#sc-100974

**Bacterial and Virus Strains**		

pKLV-U6sgRNA(BbsI)-PGKpuro2ABFP	Tzelepis et al., 2016	N/A
pMSCV-U6sgRNA(BbsI)-PGKpuro2ABFP	This paper	Addgene #102796
Mouse genome-wide lentiviral CRISPR gRNA library version 2	Tzelepis et al., 2016	Addgene: #67988
pMSCV-U6gRNA(lib)-PGKpuroT2ABFP	This paper	Addgene: #104861
pKLV2(W-)U6gRNA5(BbsI)-PGKpuroBFP	Tzelepis et al., 2016	N/A
pKLV2(gfp)U6gRNA5(BbsI)-PGKpuroBFP	Tzelepis et al., 2016	N/A
pMSCV(W-)U6sgRNA5(BbsI)-PGKpuroBFP	This paper	Addgene #102797
pMSCV(gfp)U6sgRNA5(BbsI)-PGKpuroBFP	This paper	Addgene #102798
MSCV-Bhlhe40-3xFLAG-IRES-BFP	This paper	Addgene #117263
MSCV-Pparg-3xFLAG-IRES-BFP	This paper	Addgene #117264
MSCV-Scara3-IRES-BFP	This paper	Addgene #117265
MSCV-Ccdc134-IRES-BFP	This paper	Addgene #117266
MSCV-Lrrc40-IRES-BFP	This paper	Addgene #117267
MSCV-Lrrc40-trunc-IRES-BFP	This paper	Addgene #117268
pcl-Eco plasmid	Naviaux et al., 1996	Addgene #12371
pMSCV-IRES-Blue FP	Gift from Dario Vignali	Addgene #52115
pMSCV-IRES-mCherry FP	Gift from Dario Vignali	Addgene #52114

**Chemicals, Peptides, and Recombinant Proteins**		

2-Mercaptoethanol	GIBCO	Cat#31350010
IL4	R&D Systems	Cat#404-ML-050
IL2	R&D Systems	Cat#402-ML-500
IL4	R&D Systems	Cat#204-IL-050
IL2	R&D Systems	Cat#202-IL-500
Proteinase K	ThermoFisher	Cat#100005393
T4 ligase	NEB	Cat#M0202T
Gibson assembly master mix	NEB	Cat#E2611S
Phusion polymerase	NEB	Cat#M0531L
Q5 Hot Start High-Fidelity 2 × Master Mix	NEB	Cat#M0494L
KAPA HiFi HotStart ReadyMix	KAPA Biosystems	Cat#KK2602
DNase I	Invitrogen	Cat#18068-015
SuperScript II Reverse Transcriptase	Invitrogen	Cat#18064014
Geneticin	GIBCO	Cat#10131035

**Critical Commercial Assays**		

DC Protein assay	Biorad	Cat#500-0111
KAPA Probe Fast Rox Low master mix	KAPA Biosystems	Cat#kk4718
KAPA Stranded mRNA-seq Kit	KAPA Biosystems	Cat#07962193001
Nextera DNA Library Preparation Kit	Illumina	Cat#FC-121-1030
Nextera index kit	Illumina	Cat#FC-121-1012
Nextera XT	Illumina	Cat#FC-131-1096
AMPureXP beads	Beckman Coulter	Cat#A63882
Protein A Dynabeads	Thermo Fisher	Cat#10001D
Protein G Dynabeads	Thermo Fisher	Cat#10003D
Foxp3 Transcription Factor Staining Buffer Kit	eBioscience	Cat#00-5523-00
Heat-inactivated FBS	Sigma	Cat#F9665-500ML
IMDM	GIBCO	Cat#12440053
Advanced DMEM/F12	GIBCO	Cat#12491015
OPTI-MEM	GIBCO	Cat#31985062
DPBS	GIBCO	Cat#14200075
EasySep Mouse Naive CD4+ T Cell Isolation Kit	Stem Cell Technologies	Cat#19765
Mouse CD4+ CD62L+ T Cell Isolation Kit II	Miltenyi	Cat#130-093-227
Mouse Naive CD4+ T Cell Isolation Kit	Miltenyi	Cat#130-104-453
Ficoll-Paque PLUS	GE Healthcare	Cat#17-1440-02
Dynal CD4+ isolation kit	Invitrogen	Cat#11331D
Amaxa Human Stem Cell Kit 2	Lonza	Cat#VPH-5022
Lipofectamine LTX with PLUS	Thermofisher	Cat#15338030
Blood & Cell Culture DNA Midi Kit	QIAGEN	Cat#13343
MinElute PCR Purification Kit	QIAGEN	Cat#28006
RNeasy Mini Kit	QIAGEN	Cat#74104
QIAquick Gel Extraction Kit	QIAGEN	Cat#28704
QIAquick PCR purification kit	QIAGEN	Cat#28104

**Deposited Data**		

RNA-seq time course analysis of human and mouse T helper type 2 and type 0 cells	This paper	ArrayExpress: E-MTAB-6300
RNA sequencing of T helper cell type 2 with CRISPR-mediated gene knockouts	This paper	ArrayExpress: E-MTAB-6285
ChIP-seq analysis of mouse T helper type 2 cells stimulated with CD3/28 and IL-4	This paper	ArrayExpress: E-MTAB-6276
ATAC-seq time-course of human and mouse T helper type 2 cells	This paper	ArrayExpress: E-MTAB-6292
ChIP-seq of T helper cell type 2, with retroviral overexpression	This paper	ArrayExpress: E-MTAB-7258
RNA sequencing of T helper cell type 2, with retroviral overexpression	This paper	ArrayExpress: E-MTAB-7260
Custom code in R & Java for the analysis	This paper	https://github.com/mahogny/th2crispr

**Experimental Models: Cell Lines**		

Stbl3, made competent in the lab	N/A	N/A
Dam-/dcm- competent *E. coli*	NEB	Cat#C2925I
5-alpha Electrocompetent *E. coli*	NEB	Cat#C2989K
293FT for virus production	N/A	N/A
JM8 F6 C57BL/6 ES cells	N/A	N/A

**Experimental Models: Organisms/Strains**		

C57BL/6JAX mouse strain	N/A	N/A
CBLA mouse strain	N/A	N/A
Rosa26^Cas9/+^ mouse strain	Tzelepis et al., 2016	N/A
Il13^+/Tom^ mouse strain	Barlow et al., 2012	N/A
Il4^tm1.1Wep^ mouse strain	Hu-Li et al., 2001	N/A
Gata3^GFP^ mouse strain	Grote et al., 2006	N/A
GATA3-3xFLAG-mCherry mouse strain	This paper	N/A

**Oligonucleotides**		

iPCRtag sequencing adapters	Quail et al., 2014	N/A
All other primers	This paper	Data S2

**Software and Algorithms**		

FACSanadu	Bürglin and Henriksson, 2017	http://www.facsanadu.net/
Collagene	N/A	http://www.collagene.org
BaIOPSE	This paper	Part of the deposited code, https://github.com/mahogny/th2crispr
FACSDiva	BD Biosciences	N/A
FlowJo	FlowJo	https://www.flowjo.com/
MAGeCK	Li et al., 2014	http://liulab.dfci.harvard.edu/Mageck/
STAN	Carpenter et al., 2017	https://mc-stan.org/
Cutadapt	Martin 2011	https://pypi.org/project/cutadapt/
Trimmomatic 0.36	Bolger et al., 2014	http://www.usadellab.org/cms/index.php?page=trimmomatic
HOMER	Heinz et al., 2010	http://homer.ucsd.edu/homer/
Bedtools	Quinlan and Hall, 2010	https://bedtools.readthedocs.io/en/latest/
Bowtie 2	Langmead and Salzberg, 2012	http://bowtie-bio.sourceforge.net/bowtie2/
Salmon v0.6.0	Patro et al., 2017	https://combine-lab.github.io/salmon/
MEME	Bailey et al., 2009	http://meme-suite.org
MACS2	Zhang et al., 2008	https://github.com/taoliu/MACS
FIMO	Grant et al., 2011	http://meme-suite.org
ISMARA	Balwierz et al., 2014	https://ismara.unibas.ch
GSNAP	Wu et al., 2016	http://research-pub.gene.com/gmap/
featureCounts	Liao et al., 2014b	http://bioinf.wehi.edu.au/featureCounts/
R package EdgeR	Robinson et al., 2010	https://www.bioconductor.org/
R package Rtsne	Krijthe, 2015	CRAN
R package GenomicRanges	Lawrence et al., 2013	https://www.bioconductor.org/
R package Sleuth + wasabi		https://github.com/COMBINE-lab/wasabi
R package GO.db	Carlson, 2016	CRAN
JASPAR 2016 database	Bryne et al., 2008	http://jaspar.genereg.net/
Database of C2H2 motifs	Schmitges et al., 2016	N/A
Mouse genome GRCm38	N/A	ftp://ftp.ensembl.org/pub/release-94/fasta/mus_musculus/
Human genome GRCh38	N/A	ftp://ftp.ensembl.org/pub/release-94/fasta/homo_sapiens/
Graphviz	N/A	https://www.graphviz.org
RStudio	RStudio	https://www.rstudio.com/
Inkscape	N/A	https://inkscape.org/
Google docs	Google	https://docs.google.com
Paperpile	Paperpile	https://paperpile.com

**Other**		

Resource website for the paper	This paper	http://www.teichlab.org/data/

### Contact for Reagent and Resource Sharing

Further information and requests for resources and reagents should be directed to and will be fulfilled by the Lead Contact, Sarah Teichmann (st9@sanger.ac.uk).

### Experimental Model and Subject Details

#### Generation of mouse strains

Rosa26^Cas9/+^ mice (Tzelepis et al., 2016) were crossed with other mice carrying fluorescent reporters. These strains were Gata3^GFP^ (Grote et al., 2006), Il13^+/Tom^ (Barlow et al., 2012) and Il4^tm1.1Wep^ (Hu-Li et al., 2001). For the screens we pooled mice, both heterozygous and homozygous for *Cas9* expression, male and female, of 8-12 weeks age.

The GATA3-3xFLAG-mCherry mouse strain was produced briefly as follows. The targeting construct was generated by BAC liquid recombineering (Skarnes et al., 2011) such that a CTAP TAG element was linked via a Picornavirus “self-cleaving” T2a peptide (Parks et al., 1986) to mCherry red fluorescent protein and placed upstream of a LoxP/Frt flanked promoter driven Neomycin cassette (CTAP-T2a-mCherry-Neomycin). The cassette was flanked by arms of homology and designed to fuse the tagged fluorescent cassette to the terminal *Gata3* coding exon, replacing the stop coding and a portion of the endogenous 3′UTR (Figure S3). Two sgRNA oligos, gata3_flag_^∗^ (See Supplemental data S8 for sequences), were designed to generate double-strand breaks 3′ to the terminal stop codon. The respective complementary oligos (Sigma Genosys) were annealed and cloned into a U6 expression vector. The targeting construct (2ug), along each U6 guide (1.5^∗^2ug) and wild-type Cas9 (3ug, kind gift from George Church) were nucleofected into 3^∗^10^7^ JM8 F6 C57BL/6 ES cells using Amaxa Human Stem Cell Kit 2 (Lonza #VPH-5022) and the Amaxa nucleofector B. Subsequent ES cell injections and animal husbandry were carried out by the Sanger Animal facility.

#### Use of mice in experiments

The mice were maintained under specific pathogen-free conditions at the Wellcome Trust Genome Campus Research Support Facility (Cambridge, UK). These animal facilities are approved by and registered with the UK Home Office. All procedures were in accordance with the Animals (Scientific Procedures) Act 1986. The protocols were approved by the Animal Welfare and Ethical Review Body of the Wellcome Trust Genome Campus.

For the screens we used different Cas9-expressing strains as denoted throughout the text. We pooled mice of mixed gender, heterozygous and homozygous expression of Cas9. The mice were 8-12 weeks old, healthy, and had not been used for other experiments. Between 15 and 30 mice were pooled in each replicate. Of these, some of the mice had shared cage, and mouse selection was guided by convenience and availability.

For the the time course ATAC-seq and RNA-seq experiments we used wild-type C57BL/6Jax mice, mixed gender 8-12 weeks old. Similar cohorts of homo/hetero Cas9 mice were used for the follow up individual CRISPR KO experiments. For the ChIP-seq and overexpression we used CBLA mice.

#### Human model

Umbilical cord blood was obtained from healthy neonates, mixed genders, at Turku University Central Hospital. The usage of the cord blood of unknown donors was approved by the Ethics Committee of Hospital District of Southwest Finland (24.11.1998 article 323) and informed consent was obtained from all subjects.

#### Cell lines for virus production

293T-cells (ATCC) were maintained in Advanced DMEM/F12 (GIBCO #12491015) supplemented with geneticin (500ug/mL, GIBCO #10131035) at 37C. The cells were split between every 3 to 5 days, 80%+ confluency except during transfections. The cell lines were not authenticated.

### Method Details

#### Cloning

The software Collagene (http://www.collagene.org/) was used to design and support the cloning. Phusion polymerase (NEB #M0531L) was used for all cloning PCR reactions.

The entire BFP/puromycin and sgRNA system was PCR-amplified from pKLV-U6sgRNA(BbsI)-PGKpuro2ABFP (primers: kosuke_mfei_fwd/kosuke_clai_rev). The plasmid pMSCV-IRES-mCherry FP (Addgene #52114) grown in dam-/dcm- competent *E. coli* (NEB #C2925I), was digested with NEB ClaI/MfeI and the backbone was gel purified using the QIAquick Gel Extraction Kit (QIAGEN #28704). Ligation was done with T4 ligase (NEB #M0202T). The resulting plasmid that can be used to target individual genes was named pMSCV-U6sgRNA(BbsI)-PGKpuro2ABFP (Addgene #102796).

To produce the pooled library pMSCV-U6gRNA(lib)-PGKpuroT2ABFP (Addgene: #104861) the sgRNA part of a previous mouse KO sgRNA pooled library (Tzelepis et al., 2016) (Addgene: #67988) was PCR-amplified using the primers gib_sgRNAlib_fwd/rev. Up to 1ug was loaded in a reaction and run for 10 cycles. The insert was gel purified, and then repurified/concentrated using the MinElute PCR Purification Kit (QIAGEN #28006). The backbone from pMSCV-U6sgRNA(BbsI)-PGKpuro2ABFP was obtained by BamHI-HF (NEB) digestion. The final product was produced by Gibson assembly (NEB #E2611S) and combining the output of 10 reactions. 6 tubes of 5-alpha Electrocompetent *E. coli* (NEB #C2989K) were transformed using electroporation and the final library obtained by combining 4 maxipreps. The library complexity was confirmed by streaking diluted bacteria onto plates and counting colonies. The total number of colonies was > 100x the size of the library which according to simulations in R is far beyond the requirement for faithful replication of a library (data not shown).

Two *Cas9* control viruses were also derived from pKLV2(W-)U6sgRNA5(BbsI)-PGKpuroBFP and pKLV2(gfp)U6sgRNA5(BbsI)-PGKpuroBFP. The resulting plasmids are correspondingly named pMSCV(W-)U6sgRNA5(BbsI)-PGKpuroBFP and pMSCV(gfp)U6sgRNA5(BbsI)-PGKpuroBFP (Addgene #102797, #102798). The cloning was performed in the same manner as for pMSCV-U6sgRNA(BbsI)-PGKpuro2ABFP.

#### Virus production

At least one day before transfection, 293T cells were kept in media without geneticin. When at roughly 80% confluency (day 1), the cells were transfected using Lipofectamine LTX. To a 10cm dish with 5ml advanced DMEM, we added 3ml OPTI-MEM (GIBCO #31985062) containing 36ul LTX, 15ul PLUS (Thermofisher #15338030), and a total of 7.5ug library plasmid and 7.5ug pcl-Eco plasmid (Naviaux et al., 1996) (Addgene #12371). The OPTI-MEM was incubated for 30 min prior to addition. The media was replaced with 5ml fresh Advanced DMEM/F12 the day after transfection (day 2), and virus harvested on day 3. Cells were removed by filtering through a 0.45um syringe filter. Virus was either snap frozen or stored in 4°C (never longer than day 5 before being used).

#### Validation of *Cas9* cutting in mouse

Expression of *Cas9* was confirmed by western blot (anti-Cas9, BioLegend 7A9, #844301) as well as by RT-PCR (primers: cas9_qpcr1/2/r/f). Qualitatively, *Cas9* expression appears to increase during activation of cells (data not included). The function of *Cas9* was also validated using the two control viruses and cytometric analysis. The resulting viruses express both GFP and BFP but only one of them contains a sgRNA targeting its own GFP sequence FACS analysis confirmed a reduction in GFP signal in T cells infected with the self-targeting virus, as compared to T cells infected with the control virus (data not included).

#### T cell extraction for CRISPR screening

6-well plates were first prepared at least 2 hours before by adding anti-CD3e (1ul/mL, eBioscience #16-0031-81) in PBS, at least 1.2ml/well, and then kept at 37°C.

Cells were extracted from spleens by the following procedure: Spleens were massaged through a 70um strainer into cold IMDM media (strainer slanted to avoid crushing the cells). Cells were spun down at 5min/400 g and then resuspended in 5ml red blood cell lysis media (3-4 spleens per 50ml falcon tube). After 4 min PBS was added up to 50ml and cells spun again. Cells were then resuspended in cold PBS and taken through a 70um strainer. The cells were counted and spun down again. Finally, the cells were negatively selected using EasySep Mouse Naive CD4+ T Cell Isolation Kit (Stem Cell Technologies, #19765) except for the following modifications: The volume and amount of antibodies were scaled down to 20% of that specified by the manufacturer. Up to the equivalent of the cells of 6-7 spleens can be loaded on one “The Big Easy” EasySep Magnet (Stem Cell Technologies, #18001). Overloading it will cause a severe drop in output cells.

On day 0, the cells were then resuspended in warm IMDM supplemented with 2-Mercaptoethanol “BME” (50 uM GIBCO #31350010), IL4 (10ng/mL, R&D Systems 404-ML), IL2 (6ng/mL) and anti-CD28 (3ug/mL, eBioscience #16-0281-86) and Pen/Strep, before being seeded onto the 6-well plates (30-40M cells per plate).

#### T cell culturing for CRISPR screening

On day 1, the cells were infected by the following procedure. To each well, 1.2ml media was added. This media consisted of 80% virus, 20% IMDM, supplemented with BME/IL2/IL4/anti-CD28 at concentrations as before. In addition, the media contained 8ug/mL polybrene. The plate was put in a zip-lock lag and spun at 1100 g for about 2 hours at 32°C. The plate was then put in an incubator overnight (never more than 24h in total). The cells in the media were spun down (the cells attached kept in place) and resuspended with media as after the T cell extraction except with the addition of 2ug/mL puromycin. Each well required 3-4ml media. For the 7 day culturing the media had to be replenished after half the day. We estimate that the MOI was about 0.2. The use of puromycin is essential to keep the FACS time down to reasonable levels (commonly 2ng/mL).

#### Sorting and genomic DNA extraction

On the day of sorting, cells were extracted and spun down. To eliminate dead cells we performed a “low-g spin,” 5 min at 200 g. This brought the viability up to roughly 50%. We have in addition tried other methods such as Ficoll (works slightly better but takes 30 min and is harder to reproduce) and Miltenyi Dead Cell Removal Kit. In our experience, the Miltenyi kit works great on uninfected cells but effectively removed almost every infected cell when attempted on the real sample. This is most likely because the kit does negative selection against Annexins which might be promoted by the virus or the puromycin.

In the cases when we used antibody reporters, we first fixed and permeabilized using the Foxp3 Transcription Factor Staining Buffer Kit (eBioscience, #00-5523-00). We then used the following antibodies: PE Mouse anti-XBP-1S (BD Biosciences, #562642), FITC anti-IRF4 (BD Biosciences, #11-9858-80) and Alexa Fluor 488 Mouse anti-GATA3 (BD Biosciences, #560077).

For sorting, cells were resuspended at 40M/mL in IMDM with BME and 3mM EDTA (PBS for the stained cells). The use of EDTA is essential to ensure singlet events at this high cell concentrations. The cells were then sorted into IMDM using either a Beckman MoFlo or MoFlo XDP, or BD Influx. For non-stained screens we could use BFP to ensure that the cells passed were infected. For the stained screens the BFP signal was disrupted by the staining and we performed it blindly. The subsequent steps are not affected by the addition of uninfected cells. During protocol development, the FACS data was analyzed using the software FACSanadu (Bürglin and Henriksson 2017) (http://www.facsanadu.net).

After sorting the cells, we performed DNA extraction in two different ways. When using fluorescent reporter strains we used the Blood & Cell Culture DNA Midi Kit (QIAGEN #13343). For the fixed cells, due to lack of suitable commercial kits (The FFPE kits we have seen are for low amounts of DNA only), we instead performed DNA extraction as follows. Sorted cells were pellet using a table-top centrifuge at 2000 g, 5 min. Cell pellet was resuspended in 500 ul Lysis Buffer I (50 mM HEPES.KOH, pH 7.5, 140 mM NaCl, 1 mM EDTA, 10% Glycerol, 0.5% NP-40, 0.25% Triton X-100) and rotate at 4°C for 10 min. Cells were spun down at 2000 g, 5 min, resuspended in 500 ul Lysis Buffer II (10 mM Tris.Cl, pH 8.0, 200 mM NaCl, 1 mM EDTA, 0.5 mM EGTA) and rotate at 4°C for 10 min. Then the cells were pelleted again at 2000 g for 5 min, and the cell pellet was resuspended in 25 ul Lysis Buffer III (Tris.Cl, pH 8.0, 100 mM NaCl, 1 mM EDTA, 0.5 mM EGTA, 0.1% Na-Deoxycholate, 0.5% N-lauroylsarcosine). Then 75 ul TES Buffer (50 mM Tris.Cl pH 8.0, 10 mM EDTA, 1% SDS) was added to the cell suspension. This 100 ul reaction was put on an Eppendorf ThermoMixer C to reverse crosslinking at 65°C, overnight. Then 1 ul proteinase K (20 mg/mL, ThermoFisher #100005393) was added, and protein was digested at 55°C for 1 hour. DNA was purified using MinElute PCR purification kit (QIAGEN, #28006) according to the manufacturer’s instruction. DNA concentration was measured by a Nanodrop.

#### Sequencing of CRISPR virus insert

The genomic DNA was first PCR-amplified (primers: gLibrary-HiSeq_50bp-SE_u1/l1 (Tzelepis et al., 2016)) in a reaction with Q5 Hot Start High-Fidelity 2 × Master Mix (NEB #M0494L). In each 50ul reaction, we loaded up to 3ug DNA. From each reaction we pipetted and pooled 5ul, before purifying it using the QIAquick PCR purification kit (QIAGEN #28104). The purified product was then further PCR-amplified using KAPA HiFi HotStart ReadyMix (KAPA Biosystems #KK2602) and iPCRtag sequencing adapters (Quail et al., 2014)þ. After Ampure XP bead purification (beads made up 70% of the solution) and Bioanalyzer QC, the libraries were pooled and sequenced with a HiSeq 2500 (Illumina #SY-401-2501, 19bp SE). The custom primers U6-Illumina-seq2 (R1) and iPCRtagseq (index sequencing) were used for this purpose. The original sgRNA library contained 86,035 distinct sgRNAs. In a representative sequencing run (*Gata3*, using antibody selection) the sgRNAs with fewer than 500 reads encompassing 91% of the total complexity.

#### Mouse time-course RNA-seq

CD4^+^CD62L^+^ naive T cells were purified from spleens of wild-type C57BL/6JAX adult (6 - 8 weeks) mice using the CD4+CD62L+ T Cell Isolation Kit II (Miltenyi #130-093-227). Cell culture plates were coated with anti-CD3e antibody (1 ug/mL, eBioscience #16-0031-81) in 1X DPBS (GIBCO) at 4°C overnight. Purified naive T cells were seeded at a concentration of 1 M cells/mL on the coated plates in IMDM (GIBCO) with 10% heat-inactivated FBS (Sigma #F9665-500ML), supplied with 5 ug/mL anti-CD28 (eBioscience #16-0281-86) with (Th2) or without (Th0) 10 ng/mL mouse recombinant IL-4 (R&D Systems #404-ML-050). Cells were cultured in plates for up to 72 hours.

Total RNA was purified from all the cultured cells by QIAGEN RNeasy Mini Kit according to manufacturer’s instruction, and concentration was determined by a Nanodrop. A total of 500 ng RNA was used to prepare sequencing libraries using KAPA Stranded mRNA-seq Kit (KAPA #07962193001) according to manufacturer’s instructions. Sequencing was performed on an Illumina HiSeq 2000 (125bp PE, v4 chemistry).

The efficiency of the Th2 differentiation was confirmed by antibody staining and FACS (Figure S5). *In vitro* differentiated Th2 cells were fixed, permeabilized and stained with fluorescent dye conjugated antibodies to detect intracellular cytokine expression following eBioscience intracellular staining protocol as previously described (Pramanik et al., 2018) (also described in http://tools.thermofisher.com/content/sfs/manuals/staining-intracellular-antigens-for-flow-cytometry.pdf). Fluorescent dye-conjugated primary antibodies used: IL4 (eBioscience clone #11B11), IL13 (eBioscience clone #eBio13A) and CD4 (eBioscience clone #GK1.5 or #RM4-5). Stained cells were analyzed by flow cytometry on a Fortessa (BD Biosciences) using FACSDiva and FlowJo software. CompBeads (BD Biosciences) were used for compensation where distinct positively stained populations were unavailable.

#### Human time-course RNA-seq

Mononuclear cells were isolated from the cord blood of healthy neonates at Turku University Central Hospital using Ficoll-Paque PLUS (GE Healthcare, #17-1440-02). CD4+ T cells were then isolated using the Dynal CD4+ isolation kit (Invitrogen, #11331D). CD4+ cells from three individual donors were activated directly in 24w plates with plate-bound anti-CD3 (500ng/well, Beckman Coulter, #IM1304) and soluble anti-CD28 (500 ng/mL, #Beckman Coulter, #IM1376) at a density of 2 × 10^6^ cells/mL of Yssel’s medium (Yssel et al., 1984) containing 1% human AB serum (PAA). Th2 cell polarization was initiated with IL-4 (10 ng/mL, R&D Systems, #204-IL-050). Cells activated without IL-4 were also cultured (Th0). At 48 hr, IL-2 was added to the cultures (17 ng/mL, R&D Systems, #202-IL-500). All the cells were harvested at respective time points and RNA was isolated using RNeasy Mini Kit (QIAGEN #74106) for library preparation. The efficiency of the Th2 differentiation was confirmed by measuring GATA3 levels using western blot (WB) and RT-qPCR.

For WB, cells were lysed in Triton X-100 lysis buffer (TXLB) (50 mM Tris-HCl (pH 7.5), 150 mM NaCl, 0.5% Triton X-100, 5% Glycerol, 1% SDS) and sonicated for 5 min using a Bioruptor sonicator. Protein concentration was then estimated using DC Protein assay (Biorad #500-0111). Equal protein amounts were loaded onto acrylamide gel (Bio-Rad Mini or Midi PROTEAN TGX precast gels). For protein transfer to PVDF membranes, mini or a midi transfer packs from Bio-Rad were used, depending on the gel size. Primary and secondary antibody incubations were performed in 5% Non-Fat milk or BSA in TBST buffer (0.1%Tween 20 in Tris-buffered saline). The following antibodies were used: GATA3 (BD PharMingen #558686); GAPDH (Hytest #5G4MAB6C5); Actin (Sigma, #A5441).

For RT-qPCR, RNA was isolated (RNeasy Mini Kit, #74106, QIAGEN) and treated in-column with DNase (RNase-Free Dnase Set, #79254, QIAGEN) for 15 min. The removal of genomic DNA was ascertained by treating the samples with DNase I (Invitrogen, #18068-015) before cDNA synthesis with SuperScript II Reverse Transcriptase (Invitrogen, #18064014). RT-qPCR was performed (primers: rtqpcr_hGata3_^∗^). KAPA Probe Fast Rox Low master mix (KAPA Biosystems, #kk4718) was used and amplification was monitored with QuantStudio 12K Flex Real-Time PCR System (ThermoFisher Scientific).The C_t_ values were normalized against the signal acquired with *EF1α* (primers: rtqpcr_hEF1a_^∗^).

ATAC-seq was performed from same cultures for better comparability. ATAC-seq libraries were prepared as described below.

### Time-course ATAC-seq data generation

Experiments were done according to the published protocol (Buenrostro et al., 2013)þ with some modification. Briefly, 50,000 cells were washed with ice cold 1X DPBS twice, and resuspended in a sucrose swelling buffer (0.32 M sucrose, 10 mM Tris.Cl, pH 7.5, 3 mM CaCl_2_, 2 mM MgCl_2_, 10% glycerol). The cell suspension was left on ice for 10 min. Then, a final concentration of 0.5% NP-40 was added, and the cells suspension was vortexed for 10 s and left on ice for 10 min. Nuclei was pelleted at 500 g at 4°C for 10 min. Nuclei were washed once with 1X TD buffer (from Nextera DNA Library Preparation Kit, Illumina, #FC-121-1030), and resuspended in 50 ul tagmentation mix containing:

• 25 ul 2X TD buffer (Nextera DNA Library Preparation Kit, Illumina #FC-121-1030)
• 22.5 ul H_2_O
• 2.5 ul TDE1 (Nextera DNA Library Preparation Kit, Illumina #FC-121-1030)

The tagmentation reaction was carried out on an Eppendorf ThermoMixer C at 37°C, 800 rpm, for 30 min. The reaction was stopped by the addition of 250 ul (5 volumes) Buffer PB (from QIAGEN MinElute PCR Purification Kit), The tagmented DNA was purified by QIAGEN PCR Purification Kit according to manufacturer’s instructions and eluted in 12.5 ul Buffer EB from the kit, which yielded ∼10 ul purified DNA.

The library amplification was done in a 25 ul reaction include:

• 10 ul purified DNA (from above)
• 2.5 ul PCR Primer Cocktail (Nextera DNA Library Preparation Kit, Illumina #FC-121-1030)
• 2.5 ul N5xx (Nextera index kit, Illumina #FC-121-1012)
• 2.5 ul N7xx (Nextera index kit, Illumina #FC-121-1012)
• 7.5 ul NPM PCR master mix (Nextera DNA Library Preparation Kit, Illumina #FC-121-1030)

PCR was performed as follows:

• 72°C 5 min
• 98°C 2 min
• [98°C 10 s, 63°C 30 s, 72°C 60 s] x 12
• 10°C hold

Amplified libraries were purified by double Agencourt AMPureXP beads purifications (Beckman Coulter, #A63882). 0.4X beads:DNA ratio for the first time, flow through was kept (removing large fragments); 1.4X beads:DNA ratio for the second time, beads were kept. Libraries were eluted from the beads by elution in 20 ul Buffer EB (from QIAGEN PCR Purification Kit).

1 ul library was run on a Agilent Bioanalyzer to check size distribution and quality of the libraries.

Sequencing was done with an Illumina Hiseq 2500 (75 bp PE).

#### ChIP-seq data generation

ChIPmentation (Schmidl et al., 2015)þ was used to investigate the TF binding sites. 1 million cells from each sample were crosslinked in 1% HCHO (prepared in 1X DPBS) at room temperature for 10 min, and HCHO was quenched by the addition of glycine at a final concentration of 0.125 M. Cells were pelleted at 4°C at 2000 x g, washed with ice-cold 1X DPBS twice, and snapped frozen in liquid nitrogen. The cell pellets were stored in −80°C until the experiments were performed. ChIPmentation was performed according to the version 1.0 of the published protocol (http://www.medical-epigenomics.org/papers/schmidl2015/) with some modifications at the ChIP stage. The antibody used were IRF4 (Santa Cruz Biotechnology, #sc-6059), BATF (Santa Cruz Biotechnology, #sc-100974) and FLAG (Sigma M2, #F3165).

Briefly, cell pellets were thawed on ice, and lysed in 300 ul ChIP Lysis Buffer I (50 mM HEPES.KOH, pH 7.5, 140 mM NaCl, 1 mM EDTA, pH 8.0, 10% Glycerol, 0.5% NP-40, 0.25% Triton X-100) on ice for 10 min. Then cells were pelleted at 4°C at 2000 x g for 5 min, and washed by 300 ul ChIP Lysis Buffer II (10 mM Tris.Cl, pH 8.0, 200 mM NaCl, 1 mM EDTA, pH 8.0, 0.5 mM EGTA, pH 8.0), and pelleted again at 4°C at 2000 x g for 5 min. Nuclei were resuspended in 300 ul ChIP Lysis Buffer III (10 mM Tris.Cl, pH 8.0, 100 mM NaCl, 1 mM EDTA, 0.5 mM EGTA, 0.1% Sodium Deoxycholate, 0.5% N-Lauroylsarcosine). Chromatin was sonicated using Bioruptor Pico (Diagenode) with 30 s ON/30 s OFF for 10 cycles. 30 ul 10% Triton X-100 was added into each sonicated chromatin, and insoluble chromatin was pelleted at 16,100 x g at 4°C for 10 min. 1 ul supernatant was taken as input control. The rest of the supernatant was incubated with 10 ul Protein A or G Dynabeads (Thermo Fisher #10001D, 10003D) pre-bound with 1 ug anti-FLAG in a rotating platform in a cold room overnight. Each immunoprecipitation (IP) was washed with 500 ul RIPA Buffer (50 mM HEPES.KOH, pH 7.5, 500 mM LiCl, 1 mM EDTA, 1% NP-40, 0.7% Sodium Deoxycholate, check components) for 3 times. Then, each IP was washed with 500 ul 10 mM Tris, pH 8.0 twice, and resuspended in 30 ul tagmentation reaction mix (10 mM Tris.Cl, pH 8.0, 5 mM Mg2Cl, 1 ul TDE1 (Nextera). Then, the tagmentation reaction was put on an Eppendorf ThermoMixer C at 37°C for 10 min at 800 rpm shaking. After the tagmentation reaction, each IP was washed sequentially with 500 ul RIPA Buffer twice, and 1X TE NaCl (10 mM Tris.Cl, pH 8.0, 1 mM EDTA, pH 8.0, 50 mM NaCl) once. Elution and reverse-crosslinking were done by resuspending the beads with 100 ul ChIP Elution Buffer (50 mM Tris.Cl, pH 8.0, 10 mM EDTA, pH 8.0, 1% SDS) on an Eppendorf ThermoMixer C at 65°C overnight, 1,400 rpm. DNA was purified by MinElute PCR Purification Kit (QIAGEN, #28004) and eluted in 12.5 ul Buffer EB (QIAGEN kit, #28004), which yielded ∼10 ul ChIPed DNA.

The library preparation reactions contained the following:

• 10 ul purified DNA (from above)
• 2.5 ul PCR Primer Cocktails (Nextera DNA Library Preparation Kit, Illumina #FC-121-1030)
• 2.5 ul N5xx (Nextera Index Kit, Illumina #FC-121-1012)
• 2.5 ul N7xx (Nextera index kit, Illumina #FC-121-1012)
• 7.5 ul NPM PCR Master Mix (Nextera DNA Library Preparation Kit, Illumina #FC-121-1030)

PCR was set up as follows:

• 72°C, 5 min
• 98°C, 2 min
• [98°C, 10 s, 63°C, 30 s, 72°C, 20 s] x 12
• 10°C hold

The amplified libraries were purified by double AmpureXP beads purification: first with 0.5X bead ratio, keep supernatant, second with 1.4X bead ratio, keep bound DNA. Elution was done in 20 ul Buffer EB (QIAGEN).

1 ul of library was run on an Agilent Bioanalyzer to see the size distribution. Sequencing was done on an Illumina Hiseq 2000 platform (75 bp PE, v4 chemistry).

#### Follow-up knock-out RNA-seq data generation

The backbone pMSCV-U6sgRNA(BbsI)-PGKpuro2ABFP was digested using BbsI and purified on a gel. 96^∗^2 desalted oligos for the sgRNA inserts were obtained from Sigma in premixed and diluted format. They were diluted to 10uM in T4 ligation buffer (NEB, #M0202T) and annealed (cooling from 98°C to 4°C during 1 hour on a PCR block). Ligations were performed in 10ul volume, in a 96w PCR on ice. Transformed *E. coli* (Stbl3, made competent in lab) were streaked onto 10cm ampicillin agar plates using an 8 channel pipette.

To avoid validating individual colonies, a mixture of at minimum 10+ colonies were picked and mixed for each clone. Digest by BbsI of a few representative shows at the minimum presence of clones without original bbsI spacer. Bacteria were grown overnight in a 96w deep-well plate having an air-permeable seal. Minipreps were made using a homemade gravity manifold holding miniprep tubes (blueprint for laser cutting available on request). The virus was subsequently made in 293T-cells, in 24w format. The virus was then harvested into a 96w deep-well plate and any 293T removed by centrifugation.

Naive T cells were extracted from 3 mice independently, this time with the Naive CD4+ T Cell Isolation Kit (Miltenyi #130-104-453) according to manufacturer’s instruction. Cells were seeded at 200k/well density in 96w format. Infection and puromycin selection was then performed as before. On day 5, cells were washed and dead cells removed by low-G spin. This typically raised the viability from roughly 10%–20% to 60% according to Trypan blue. Cells were spun down and as much of the media removed as possible. Up to 100ul of buffer RLT+ was then added to each well and plates frozen. Later, plates were thawed and RNA extracted by adding 100ul of Ampure XP beads. Purification was done by a robot, with 2x200ul EtOH wash and final suspension in RNase-free water. RNA was then diluted to 500ng/ul and 2ul was taken as input into non-capping DOG-seq (manuscript in preparation). This protocol is for this application roughly equivalent to Smartseq-2 (Picelli et al., 2014). Libraries were made using Nextera XT (Illumina, #FC-131-1096) and all 96^∗^3 libraries sequenced with a HiSeq 2500 (150bp PE).

#### Follow-up overexpression RNA-seq and ChIP-seq data generation

The plasmid pMSCV-IRES-Blue FP (Addgene #52115, gift from Dario Vignali) was digested with NEB MfeI-HF and BamHI-HF and purified with the QIAquick PCR Purification Kit.

The cDNA for cloning was generated by RLT lysis of *in vitro* Th2 cells (naive and day 5), Ampure XP bead purification, and SmartSeq2 first strand synthesis without the addition of the TSO.

For each gene, PCR primers were generated using an R script. The CDS of each gene was downloaded from Ensembl, and the most DE transcript as given by our RNA-seq time course was selected. Genes that later proved hard to clone from cDNA were ordered as gBlocks, with optional codon optimization using the IDTDNA web interface.

The first (genefwd) and last 30bp (generev) were used as gene-specific PCR primer part. We created the primers by concatenating sequences as follows, where RC denotes reverse complement: primer_fwd = (overlapFWD, seqkozak, genefwd), primer_3xflag_fwd = (seq3xflag, flagspacer, genefwd) and primer_rev = (overlapREV, rc(seqStop), generev). Further, to genes not starting with the codon G after ATG, the sequence GAG was added. All primers mentioned here were ordered PAGE purified from IDTDNA. The sequences were fwd_3x = TCTTACGTAGCTAGCGGATCttaaccatggactacaaagaccatgacggtgattataaagatcatgacatcgattacaaggatg, seq3xflag = cggtgattataaagatcatgacatcgattacaaggatgacgatgacaag, rev_3x = AATTGATCCCGCTCGAGCCTACTTGTCATCGTCATCCTTGTAATCGATGTCATGATCTTTATAATCACCG, seqkozak = ttaaccatg, seqStop = tag. The gene specific forward sequences are in the Supplemental Datas. The specific gene product was first obtained by PCR of the cDNA with Phusion master mix (NEB #M0531L), gene_fwd and gene_rev primers (25 cycles, 2 min extension, 72C annealing). The result was run on an 1% agarose gel, the band cut, and purified with Qiaquick gel extraction kit (QIAGEN #28704) and Qiaquick PCR purification kit (QIAGEN #28104).

To obtain 3xFLAG versions of the insert, a phusion PCR reactions were set up (8 cycles, 65C annealing, 2min extension) over the previously amplified non-3xFLAG fragments, primers fwd_3x, primer_3xflag_fwd and primer_rev. The gene specific inner primer was used at 0.5uM concentration as opposed to 10uM for the outer primers. The products were purified by Ampure XP and eluted into 10ul NFW.

The inserts and the MSCV backbone were joined with Gibson assembly master mix (NEB #E2611S) in 10ul reactions. 1ul of the ligated product was transformed in 25ul NEB DH5a competent cells, according to manufacturer’s specification.

Colonies were picked, amplified in 5ml LB (50ml Falcon tube) and plasmid purified by miniprep. Validation was done by two rounds of Sanger sequencing, forward (mscv_seq2,CTTGAACCTCCTCGTTCGAC) and reverse (mscv_seq3,TAACATATAGACAAACGCACACCG). A custom Java program was written to find the best matching expected sequence (generated by previous R script) and output a FASTA file with the reference and reads (reverse read reverse-complemented). The sequences for each clone were aligned by CLUSTALW (Chenna et al., 2003). The result was visualized with CLUSTALX and/or a plain text editor. The sanger sequencing reads of all clones are available as-is on GitHub. The finally selected clones are available on AddGene with IDs #117263 - 117267.

Naive T cells from CBLA mice were purified, cultured *in vitro* with IL4, and transduced as described before. The original pMSCV backbone was used as a negative control. On day 5, 20k BFP+ cells were FACS sorted, spun down and lysed in RLT. RNA-seq libraries were generated as described before. The remaining cells were used for ChIPmentation, with the IP performed against 3xFLAG (SIGMA, clone F1804). RNA-seq read and ChIPmentaton reads were processed as before. RNA-seq and ChIP-seq libraries were sequenced on a HiSeq 2500, 50BP SE.

### Quantification and Statistical Analysis

#### Analysis and QC of CRISPR hits

Sequencing BAM-files were transformed into FASTQ using samtools and bamToFastq. A custom Java program was then used extract per-sgRNA read counts. From these, per-gene p values were calculated using MAGeCK (Li et al., 2014)þ using the positive and negative cell fraction from each screen. The hit rankings were then compared using R. To obtain a total per-gene score, we first calculate the total rank from one screen as r = min(r_pos_,r_neg_), using the ranks from the positive and negative enrichments respectively. Then, to calculate the composite score of two or more screens, we used the geometric mean (r_1_r_2_r_3_...r_n_)^1/n^. Follow-up hits were manually picked as those scoring high between the replicates, with genes of low expression level qualitatively filtered out using ImmGen (Heng et al., 2008)þ.

The BaIOPSE model was implemented in STAN (Carpenter et al., 2017) using the RStan interface. For the full model implementation and parameters, with variances rather defined by the exponentials over the priors, we refer to the source code. 12 Markov chains were run 800 steps and convergence was checked by the r-value. The top 300 hits were used to calculate GO term *p*-values. GO terms were obtained in R by GO.db (Carlson 2016) and assessed individually using a Fisher exact test.

#### ChIP-seq peak analysis

The reads were first trimmed using Trimmomatic 0.36 (Bolger et al., 2014) with settings ILLUMINACLIP:NexteraPE-PE.fa:2:30:10 LEADING:3 TRAILING:3 SLIDINGWINDOW:4:15 MINLEN:30. Reads were mapped to the mouse reference genome mm10 by bowtie2 (Langmead & Salzberg 2012)þ. Peaks were then called using MACS2 (Zhang et al., 2008), merged over time, and annotated using HOMER (Heinz et al., 2010).

The quality of the peaks was assessed using the two available replicates for each time point. While the trend over time agreed, the number in each time point did not. For this reason we decided to consider the union of the peaks rather than the common peaks.

The sequences of the detected ChIP-seq peaks were extracted using “bedtools getfasta” (Quinlan & Hall 2010), for 200, 300, 400, 500bp regions around the peaks. These were fed into MEME (Bailey et al., 2009) for additional motif discovery.

To compare genes associated to increasing and non-increasing GATA3 peaks, we calculated the relative peak height at 72h versus 24h. We define the most/least increasing as the peaks with top/bottom 1000 ratios, and then included the genes the peaks are closest to. GO analysis were performed to compare these groups.

#### Time-course RNA-seq differential expression

Gene expression from RNA-seq data was quantified in TPM using Salmon v0.6.0 (Patro et al., 2017)þ, with the parameters–fldMax 150000000–fldMean 350–fldSD 250–numBootstraps 100–biasCorrect–allowOrphans–useVBOpt. The cDNA sequences supplied contain genes from GRCm38 (mouse), GRCh38 (human) and sequences from RepBase, as well as ERCC sequences and an eGFP sequence.

Differentially expressed (DE) genes were found using the Sleuth R package (Pimentel et al., 2017), using the wasabi R package (https://github.com/COMBINE-lab/wasabi) to allow it to accept Salmon input data. To strengthen the test of differential dynamics between Th2 and Th0 culture conditions, instead of testing each time point individually (with few replicates), we separated time into early (≤6h) and late (> 6h). The DE test consisted of a likelihood-ratio test using the sleuth_lrt function, where the full model contained terms accounting for the culture condition, for the temporal effect (modeled as a spline with 5 degrees of freedom) and for an interaction of both terms. To capture the Th0/Th2 difference, the reduced model only contained a term accounting for the time variation, modeled as before. A gene is considered differentially expressed for p value < 0.01.

#### Human/Mouse *Stat6* comparison

Targets of Stat6 and Il4 as defined by time-course microarray and ChIP-seq data were downloaded from a previous study (Elo et al., 2010).

#### ATAC-seq motif extraction

ATAC-seq reads were aligned using Bowtie 2 (Langmead & Salzberg 2012)þ with the parameter –X 2000 and the mouse genome mm10. This was followed by peak calling on each replicate individually using MACS2 (Zhang et al., 2008)þ with the function “callpeak” and the parameters -B–SPMR–call-summits. The peaks obtained were kept if they overlapped a peak from the other replicate of the same time point by at least 50%. In these cases, the new peak would equal the combined coordinates of all the overlapping peaks considering all replicates and time points.

Peaks were classified (annotatePeaks.pl–annStats) as intronic, exonic, upstream or intergenic, according to the gene feature they intersected. Intersection is scored first considering the number of bases overlapped, and then the closeness in size between the peak and the feature.

Known motif detection was performed on the peaks’ sequences using FIMO (Grant et al., 2011)þ, and motifs from the JASPAR 2016 database (Bryne et al., 2008)þ considering only those starting in MA or PB. In addition, we supplemented with a more recent list of C2H2 motifs (Schmitges et al., 2016). To make the analysis more targeted, only motifs from TFs DE between Th2 and Th0 were considered, and for each of them a single motif was selected, prioritizing the longest ones with the lowest mean entropy.

The overlap between human and mouse was calculated using liftOver -minMatch = 0.03 -multiple. Roughly 100 peaks mapped to multiple sites and were thus ignored. LiftOver was also performed on individual TF sites from FIMO. The overlap between organisms was calculated using R GenomicRanges (Lawrence et al., 2013). The overlap procedure was done at the peak and detected motif levels.

We found that the analyses throughout the paper appear to give similar results when using all mouse peaks as opposed to only using the conserved (overlapping) peaks. However, the ChIP-seq peaks of GATA3, IRF4 and BATF appear more comparable to ATAC-seq predicted sites if only the conserved sites are used are used in the MARA.

#### ATAC-seq chromatin dynamics analysis

The height of the peaks, as well as any reads outside the peaks, were quantified using bedtools (Quinlan & Hall 2010). The peak levels were divided by the background signal for normalization. Further, to make the contributions from different peaks comparable, they were normalized to the level of the second time point. The contribution of motifs over time is defined as the average peak signal in which they are present.

#### UCSC visualization of ChIP-seq and ATAC-seq

The MACS2-generated BedGraph files were prepared for UCSC visualization using bedSort and bedGraphToBigWig.

#### MARA analysis

The MARA analysis was performed as follows. Early and late times were analyzed independently. For each of the two durations, the connectivity matrix was constructed based on if a motif peak was present for a gene at any time. The number of such peaks, ignoring time fluctuations, were entered as the connectivity value. The full RNA-seq time-course data for either Th0 or Th2 was used as the signal. These two files were uploaded to ISMARA (Balwierz et al., 2014) using expert mode.

In the MARA comparison over time, Th0 and Th2 difference is calculated as the average MARA activity difference over time. The activity increase is taken as the difference in activity at the first and last time points for Th0.

#### Follow-up knock-out RNA-seq analysis

Reads were filtered using Cutadapt (Martin 2011) for the Smart-seq2 TSO and mapped using GSNAP (Wu et al., 2016)þ. The software featureCounts was then used to produce a final count table (Liao et al., 2014b)þ. The effects of the KO was studied using an EdgeR (Robinson et al., 2010) linear regression model using the KO with scrambled sgRNA as reference point. We studied the impact of the virus infection level, measured as a function of BFP, and found it to be confounding. To obtain stronger DE effect for future KO experiments we recommend that non-infected cells are removed by FACS sorting rather than puromycin selection. Individual replicates were compared in terms of p value and correlation of DE genes when one sgRNA was used versus when several sgRNAs targeting the same gene were pooled. Libraries with low replicability or low virus infection were manually removed.

We define a differentiation axis as the DE genes (using DEseq2) from the RNA-seq time-course, Th0 versus Th2 at t = 72h, with p < 10^−10^. The activation axis is similarly defined as the DE genes Th0 at t = 0h versus Th0 at t = 72h, with p < 10^−10^.

#### Follow-up overexpression RNA-seq analysis

Presence of the overexpressed gene was verified by manual inspection of RNA-seq reads as well as *p*-value in DE gene list. Constructs failing this test were excluded. DESeq2 was used for the analysis and we tested 3 linear models: ∼treatment, ∼treatment + mouse, and treatment versus all_samples. They all give consistent results. Here we report using the model ∼treatment + mouse, and do not report genes which were less consistent between the models. Interestingly we obtained two clones of *Lrrc40*, where one was truncated on the 3′, but they qualitatively yield the same DE genes during overexpression, and cluster together using tSNE (data not shown). We have deposited both versions to Addgene, but used the full-length *Lrrc40* DE gene list for validation. For sequencing data, see Addgene entry.

#### Clustering and analysis of ChIP-seq datasets

Additional BED-files of ChIP-seq datasets were downloaded from Cistrome (Liu et al., 2011). The following external datasets were included, in addition to the 3xFLAG BHLHE40 & PPARG, and our endogenous BATF, IRF4 and GATA3 ChIP-seq: ASCL2 Th0 overexpression (Liu et al., 2014) (GSM1276938), BACH2 Th2 (Kuwahara et al., 2016) (GSM1547779), CDK9 Th2 (Hertweck et al., 2016) (GSM1527704), E2F4 DC cells 120 min post LPS-stimulation (Garber et al., 2012) (GSM881061), E2F1 DC cells 120 min post LPS-stimulation (Garber et al., 2012) (GSM881057), ELF1 Th0 FOXP3-(Samstein et al., 2012) (GSM999185), ETS1 Th2 (Wei et al., 2011) (GSM654875), ETV6 Th2 (Humblin et al., 2017) (GSM2634697), FLI1 Th2 (Wei et al., 2011) (GSM654872), FOLS2 Th0 (Liao et al., 2014a) (GSM1004808), FOXO1 Foxp3+ CD4 (Liao et al., 2014a) (GSM1480611), FOXP3 Treg (Hayatsu et al., 2017) (GSM2387501), GTF2B CD4/CD8 (Koch et al., 2011) (GSM727002), GTF2F1 CD4/CD8 (Koch et al., 2011) (GSM727004), IKZF2 Treg (Kim et al., 2015) (GSM1876372), IRF8 Th2 (Humblin et al., 2017) (GSM2634696), JARID2 Th17 (Escobar et al., 2014) (GSM1151625), JUN CD4 (Li et al., 2012) (GSM978754), JUNB CD4 resting and activated (Bevington et al., 2016) (GSM1646847 and GSM1646848), MAF Th17 (Escobar et al., 2014) (GSM1151623), NIPBL CD4/CD8 (Seitan et al., 2013) (GSM1184315), PARP14 Th2 (Riley et al., 2013) (GSM1242997), PRDM1 Treg (Jain et al., 2016) (GSM1964752), RAD21 CD4/CD8 (Seitan et al., 2013) (GSM1184316), RARA Th1 (Brown et al., 2015) (GSM1474186), RORC Th0 (Ciofani et al., 2012) (GSM1004853), RUNX1 CD4 (Bevington et al., 2016) (GSM1646844), RUNX1 CD4 + PMA (Bevington et al., 2016) (GSM1646846), SATB1 peripheral CD4 (Kakugawa et al., 2017) (GSM2409720), SATB1 thymus CD4 (Kakugawa et al., 2017) (GSM2409719), SMAD4 Th17 (Zhang et al., 2017) (GSM2706519 and GSM2706520), SMARCA4 Th2 resting and stimulated (De et al., 2011) (GSM585295 and GSM585297), SMC1A CD4/CD8 (Ing-Simmons et al., 2015) (GSM1504389, GSM1504390), SPNS1 Th2 (Wei et al., 2010) (GSM550319), STAT1 (Vahedi et al., 2012) (GSM994528), STAT1 Th0 (Hirahara et al., 2015) (GSM1601720), STAT3 Th0 (Hirahara et al., 2015) (GSM1601721), STAT2 Th0 (Iwata et al., 2017) (GSM2538951), STAT3 Th0 (Ciofani et al., 2012) (GSM1004857), STAT4 Th1 (Wei et al., 2010) (GSM550303), STAT5 Th0 (Villarino et al., 2016) (GSM2055717 and GSM2055711), STAT6 Th2 (Wei et al., 2010) (SRR054675), TBX21 Th1 (Nakayamada et al., 2011) (GSM836124), XBP1 Th2 (E-MTAB-6327) (Pramanik et al., 2018).

The Rtsne package (Krijthe 2015) was used on a matrix consisting of 1 wherever a gene had a close ChIP peak according to annotatePeaks.pl (Heinz et al., 2010), otherwise 0. To generate the network diagrams, the output of annotatePeaks.pl was processed with R to select peaks near the TSS of chosen genes. The network was written to an output file and rendered using Graphviz (https://www.graphviz.org).

### Data and Software Availability

#### Plasmid resources

All the plasmids including the plasmid library are available from Addgene (see Online Methods for accession numbers).

#### Data resources

The sequencing data has been deposited at ArrayExpress: E-MTAB-6276, E-MTAB-6285, E-MTAB-6292, E-MTAB-6300, E-MTAB-7258 and E-MTAB-7260. Selected parts of the data are also available for online visualization at http://www.teichlab.org/data/.

#### Software resources

The R code used for the analysis is available on Github (https://github.com/mahogny/th2crispr). This code also covers the BaIOPSE algorithm.

## Web Resources

Teichmann Lab, http://www.teichlab.org/data/

## Supplemental Information

Data S1. Current Protocol for Producing Virus and Transducing T Cells, Related to Figure 1

Data S2. Processed Data from All the Steps of the Analysis, Related to Figure 1
