# Supplementary material for: Genome-wide CRISPR Screens in T Helper Cells Reveal Pervasive Crosstalk between Activation and Differentiation
Source: Cell. 2019 Feb 7;176(4):882–896.e18. doi: 10.1016/j.cell.2018.11.044 (PMC6370901; doi:10.1016/j.cell.2018.11.044)
Supplement: Data S1. Current Protocol for Producing Virus and Transducing T Cells, Related to Figure 1 [file mmc1.pdf]

Cell, Volume *176*

Supplemental Information

Genome-wide CRISPR Screens in T Helper Cells Reveal Pervasive Crosstalk between Activation and Differentiation

Johan Henriksson, Xi Chen, Tomás Gomes, Ubaid Ullah, Kerstin B. Meyer, Ricardo Miragaia, Graham Duddy, Jhuma Pramanik, Kosuke Yusa, Riitta Lahesmaa, and Sarah A. Teichmann

Manual for CRISPR KO screening in primary mouse T helper cells

These instructions are similar but not exactly those we use in the paper "Genome-wide CRISPR screens in T helper cells reveal pervasive cross-talk between activation and differentiation". Several steps can be improved further; in particular, higher virus titres can be achieved using caffeine (https://www.ncbi.nlm.nih.gov/pubmed/20626321). There are mixed reports of the ability to freeze mouse-specific retrovirus, which would simplify the protocol execution greatly. Concentration by ultracentrifugation has not worked well for us, but overnight spins at lower g might work better. This protocol does not have a freezing step and need careful logistical planning. The final sequencing of the CRISPR library is the same as for Kosuke Yusas mouse v.2 KO library, from which our library is derived (https://www.addgene.org/pooled-library/yusa-crispr-knockout-mouse-v2/).

Maintenance of 293T cells for virus production

We use 293T for virus production normally, but it is possible to also use a producer cell line. Even in this case it is worth supplementing the with pCL-ECO, the envelope plasmid. Do not under any circumstance let the cells go overconfluent as this reduces the proliferation rate. Subsequent virus production/transfection is severely impaired by this. Avoid running too many passages (<20-30?).

Splitting: Expect 2x growth per day

If you want to put 293T cells onto gelatin coated plates then first take 10cm dishes and add 4ml 0.1% gelatin. Incubate them at 37°C for at least 30 min. Then just remove the gelatin-containing media before adding the T cells.

To split:

1. Remove media from plate.

2. Retrieve 0.25% trypsin straight from fridge, dilute 10x in room temperature PBS.

3. Add 3ml trypsin to a 10cm dish with 293T. Wait 15 seconds. Remove media.

4. Put plate into incubator for 2 minutes (or 2.5). Do not stack the plates - if you do then reheating will take longer, and possibly be uneven, affecting the trypsinization.

5. Quick method: Flush off the cells with any amount of media (e.g. 5ml). Pipette up and down to resuspend single cells. Slow method: Flush of the cells with any media, centrifuge 450g 5min. Remove media, add 1ml, resuspend by pipetting.

6. Dilute to the amount of media needed. Add geneticin unless you are making virus the day after (50mg/µl is 100x but can be used as 500x or 1000x). Geneticin is optional but supposed to maintain and select for the cassette with the SV40 large T antigen. This may boost virus production. We frequently omit this step.

Thawing

Heat media first. Then heat cells in water bath (fast heating is good). First add 1ml media to cells. Add a bit first reduces cell death due to osmotic pressure. Then add cells to the amount of media you want. Optionally do a wash to remove DMSO here, but not sure if it is needed

Freezing

Pellet cells from 1 10cm dish. Resuspend in 1ml media with 10% DMSO. Freeze in -80°C using a Mr. Frosty. Work quickly as DMSO is toxic to the cells. Keep the cells in liquid nitrogen for long-term storage. The cells will deteriorate in -70°C.

96 well-cloning of targeted constructs into backbone

Order oligos, 2 per well, normalized in media. If ordering in 96 well format, avoid getting deep-well plates as they are hard to pipette from. Note that you can also do the cloning using a single oligo if doing Gibson assembly, though this is more expensive than ligation.

0. Ready a water bath, 42°C

1. Anneal oligos in NEB T4 buffer. 10µl oligos + 80µl NFW + 10µl T4 buffer(10x). Annealing program (1.5h) exists in PCR machine

2. Digest MSCV-grnav2 with NEB Bbsi, buffer 2. 10µl buffer + 1µg plasmid, total in 50µl NFW. In the end, you need 25ng plasmid per well (but count on 50ng). Might as well digest 8-10µg (one qiagen miniprep column has 10µg capacity)

3a. If you want to play safe, then gel purify the digest. If you do then also run a PCR cleanup after gel purification, as an extra wash step. Minelute PCR columns allow you to increase concentration but for this amount of plasmid, Qiagen works too. It is possible to elute into 50µl for a large amount of plasmid

3b. If you instead prefer to rely on the ccdB1 insert to select against unwanted ligations then just perform a PCR cleanup reaction.

4. Dilute oligos. Have done 2µl oligos + 98µl EB, then 5µl of that + 75µl EB (about 0.75ng/µl). Would in the future change the first step to reduce dilution variability there. It is up to 30-40% according to nanodrop

5. <u>All the following on ice+water all the time</u>. Note that if you make a master mix of backbone + T4 ligase then if it goes warm there is a chance it will self-circularize before you add the insert. Keeping it cold prevents this. Use a large flat ice-water filled tray to allow easy contact between ice and 96 well plates, or use dedicated chilled multiwell blocks.

6. For one well: Total 10µl with NFW. 0.5µl T4 and 1µl T4 buffer. 25ng plasmid and 0.75ng insert. Probably better to increase first dilution so you can add 2µl here instead of 1µl. Add the enzyme last.

7. Spin down plate. Incubate 22°C, 60 min (30 min might be ok)

8. Prepare bacteria in 96 well prechilled PCR plates. Add 15µl bacteria/well

9. Add 1µl of ligation mix per well. Do not pipette up and down too much, but rather swirl the pipette a bit. Maybe good to tap the entire plate as well

10. Incubate 30 min on ice (check competent cell manual for time)

11. Plunge into water bath, incubate 30s @ 42°C (check competent cell manual for time)

12. Incubate on ice, 2min

13. Add 150µl LB (or SOC)

14. Incubate in shaking incubator, 30min

15. Meanwhile, preheat LB-amp-plates for 30min - 1h. Do not heat them longer or they risk running too dry

16. Streak with an 8-channel pipette on LB-amp plates. You can easily pipette up to 40µl/tip if you leave the plates with this side upwards (100µl if careful). It is key that the plates have the right amount of dryness, or the media will either be sucked in, or the streaks will mix. Do up to 3 plates/8 channels to ensure you have enough colonies (2 plates is good). Notation: Write the letter of the column (before streaking) on the side you will streak from. Streak from left to right, with (1) on the top. Move to the incubator with the streaks still turned up.

Validation of sgRNA plasmid insert

The final validation is best done by Sanger sequencing, using primer mathU6seq: CGATACAAGGCTGTTAGAG (thanks to Mathias Friedrich).

A quick first check can be done by digestion and running the product on a gel. Digest by NEB EcoRI in buffer 2. Two fragments (8400+630) will appear if the original insert is left, otherwise just linear band (~8400). Do not do overnight digest. I have noticed that the entire plasmid can get digested (at least with this restriction enzyme). This may be due to remaining exonucleases if you miniprepped the plasmid.

This is a master mix for 500ng/µl plasmid, 20µl/reaction and 20 reactions:

40µl	NEB buffer 2
8µl	NEB EcoRI
352µl	NFW
**400µl**	**Total**

Mix 19µl + 1µl plasmid. Digest for 1h and run the product on a gel.

Virus production with lipofectamine LTX

The production also works with LT2000, but then instead use Kosuke Yusas protocol that only uses optiMEM during transfection. The most crucial parameter is confluence. If the cells ever hit the limit and becomes retarded then discard them and restart. There is no saving them from there. Lipofectamine can vary 35 - 45µl without major change in efficiency, and plasmid 12-15µg with ease.

Grow 293T in Advanced dmem/f12 with FBS and P/S. Maintain on 1-10µg/ml geneticin (recommendation from Sebastian Łukasiak who uses 10µg; we prefer 1µg). Be sure to remove geneticin the day before transfection or the titre will be low. Aim for 80% confluence on the day of transfection.

On the day of transfection:

1. Replace media with 5ml advanced DMEM (only applies to LTX, not LT2000). Kosuke Yusas version: Use 5ml optiMEM instead

    * For one 6w: 1.5ml DMEM

2. For one 10cm plate, mix 3ml optiMEM with 7.5µg pcl-eco and 7.5µg MSCV library/target vector, and 15µl PLUS reagent. Possible to go down to 12µg total plasmid and 12µl PLUS and it does not have a major effect on titre, but you want to do everything to keep it high for the library virus

    * For one 6w: 0.5ml optiMEM. 1+1µg plasmid. 2µl PLUS

3. Vortex, incubate at room temperature for 5min.

4. Add lipofectamine LTX, 36µl. Mix gently, incubate at room temperature for at least 30min. The manufacturer's protocol seems to suggest 30min-4h is a good interval but not too early for certain.

    * For one 6w: 6µl LTX

5. Add the mix to plate with 293T. Some protocols suggest adding it dropwise but this does not seem to be too important.

6. With Advanced DMEM: you can here leave it overnight. Kosuke Yusas protocol, with optiMEM, suggests to replace with 10ml advanced DMEM 5-7h later.

7. The day after, replace with 5ml advanced DMEM in the morning. CHECK: you will see that at least 50% of the cells are shining blue under a microscope in this afternoon. It should be extremely obvious or something is wrong

    * For one 6w: 1.5ml

8. The afternoon the next day, harvest virus. Media will have changed color. Sebastian Łukasiak harvests a bit earlier. There are plots from Clontech suggesting you get another 10-20% titre by waiting an extra day. Some harvest on two different days but this is only really of use if you can freeze the virus.

9. Take medium through a 20µm syringe filter. For target virus, 500-800µl aliquots is suitable. For library, keep a mix of 10ml, 5ml and 2ml aliquots in 10ml falcon tubes.

Virus production with PEI

This much cheaper methods is suitable for targeted KO but not for library making as the titre is 50% of LTX. Concentration of MSCV does not seem possible or at least not easy, but has not been tested enough.

This method, like calcium-phosphate transfection, seems based to creating cell-piercing crystals with DNA. Getting the crystal size right is crucial. Calcium-phosphate appears to give comparable results to LTX in the right hands, but PEI is much easier and the buffer lasts forever once made.

Transfection:

1. Treat the 293T as with LTX transfection (maintenance etc).

2. For 10cm plates: Change media to 9ml media on day of transfection.

3. In a sterile tube dilute total plasmid DNA (µg) in 1m, optiMEM (volume of media is 10% of final volume in culture vessel). Total plasmid (pCL-eco + MSCV, 50/50%) does not seem to be a major effector. 8-12µg total is a good number.

For 6 well plate: Add 3µl DNA in 200µl optiMEM

For 10cm plate. Add 8µg DNA in 1ml optiMEM

4. Add PEI mix (1ug/µl) to the diluted DNA. Mix immediately by vortexing or pipetting. The ratio (PEI:DNA) can be 3:1 but in my hands the ratio does not seem to be too important. Anything from 2:1 to even 4:1 seems to work.

For 6 well plate: Add 9µl PEI

For 10cm plate: Add 21µl PEI

5. Incubate 15 minutes at RT. I have tried 6-15min and it all works. Claimed to be the most critical parameter.

6. Add DNA/PEI mixture to cells, now 10ml total (for 10cm).

7. The day after, change media to 5ml. In my trial I changed to 4ml but I had less cells than were optimal.

7. Harvest transfected cells and/or viral supernatant at 48 hours post-transfection. See LTX protocol.

FUTURE MODIFICATION: To make it possible to use this protocol for many different viruses, I think the timing has to be controlled better. The crystal formation likely stops after dilution. So if one adds only 3ml media to the cells, and then dilutes the 1ml mix with 6ml media, then one has more time to perform all the steps.

Making the PEI stock solution (1µg/µl) – PEI is Polyethylenimine 25kD linear, order from for example Polysciences (cat# 23966-2).

1. Dissolve PEI in endotoxin-free dH2O that has been heated to ~80°C. You can use a microwave for this.

2. Let cool to room temperature.

3. Drop the pH to at least 4.0 (Important! Not written in any other protocol; thanks to Laura Wood for pointing this out). Neutralize to pH 7.0, filter sterilize (0.22µm), aliquot and store at -20°C; a working stock can be kept at 4°C.

Estimation of required number of cells

The number of required cells is easiest to estimate by computer simulation. While we have not validated the results, this may still provide a back-of-an-envelope estimate of the required amount. There are several bottlenecks through-out the protocol and it makes sense to try and balance these:

- Many cells die during activation and will thus restrict the effective number of available cells
- Any skew in the complexity of the library will propagate. We assumed the library to be uniform
- The number of infected cells is a key parameter

- The sequencing depth is another key parameter
- The expected fold-change of the KO that you wish to detect
- The number of sgRNAs expected to work (unknown) and the number of sgRNAs per gene

For discussion, we talk about the rate at which viruses enters cells, MOI (λ), and the probability of a cell to be seen as infected (c). The relationship is, assuming a poissonian distribution, $\lambda = -\log(1-c)$. It is generally easier to talk about MOI than infected cells. A higher MOI (λ) means more cells to measure, but also more cells with more than one sgRNA inserted. The ideal tradeoff depends on your scored phenotype. Commonly MOI=0.3 is used. The following R code shows the probability of an observed transduced cell being a single transduction:

```
lambdas <- (0:150)/100
prob_0     <- exp(-lambdas)
prob_1     <- lambdas*exp(-lambdas)
prob_2plus <- 1 - prob_0 - prob_1  #Not 0 and not 1
plot(lambdas,prob_1)
plot(lambdas,prob_1/(prob_1+prob_2plus),xlab="MOI",
      ylab="P[Observed cell is single-transduced]",type="l")
```

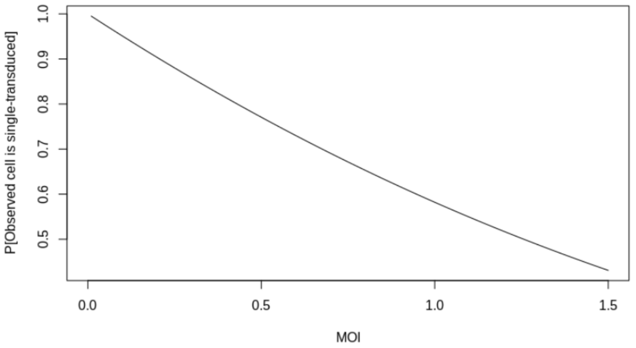

To get an idea of the number of required cells, we chose to look at the expected number of false positives. It is difficult to reason about how sgRNAs are merged together as a per-gene score (indeed, we came up with a whole Bayesian framework in our paper to calculate this). Thus we only look at the scores for one sgRNA. How many sgRNAs will by chance be reported to have a >2x fold change? The following code calculates this using the EAT model (https://www.ncbi.nlm.nih.gov/pubmed/27176874), as a function of number of plated cells N, for 88k sgRNAs:

```
makelev<-function(d,lev){
  if(length(d)==0)
    return(data.frame(id=c(),stage=c()))
  else
    return(data.frame(id=d,stage=lev))
}

dodiv <- function(cells){

  p <- runif(n=nrow(cells),min=0,max=1)
  keep1 <- cells[which(cells$stage==1 & p>=0.24 & p<0.94),]
  div1 <- makelev(cells$id[which(cells$stage==1 & p>=0.94)],2)

  p <- runif(n=nrow(cells),min=0,max=1)
  keep2 <- cells[which(cells$stage==2 & p<0.67),]
  div2 <- makelev(cells$id[which(cells$stage==2 & p>=0.67 & p<0.98)],2)
  cont2 <- makelev(cells$id[which(cells$stage==2 & p>=0.98)],3)

  p <- runif(n=nrow(cells),min=0,max=1)
  keep3 <- cells[which(cells$stage==2 & p<0.22),]
  div3 <- makelev(cells$id[which(cells$stage==3 & p>=0.22)],3)

  cells <- rbind(keep1,div1,div1,
                 keep2,div2,div2,cont2,
                 keep3,div3,div3)
  return(cells)
}
```

```
cells <- data.frame(id=round(runif(1e5,min=0,max=88000)),stage=1)

numdiv <- round(5*24/14) ## Number of divisions in 5 days
numdiv <- 8

## Simulate all the divisions
for(i in 1:numdiv){
  cells <- dodiv(cells)
  print(length(which(cells$stage!=1))/nrow(cells))
  print(nrow(cells))
}

## Check fold-change by random chance
n <- as.numeric(table(cells$id))
hist(n)
N=100000
pmorethan2 <- length(which(abs(log2(n[runif(N,min=1,max=length(n))] /
n[runif(N,min=1,max=length(n))]))>1))/N
pmorethan2
```

Example output numbers are 0.1% for 30M, and 5% for 10M. The sequencing bottleneck is to be added on top of these numbers. So even with 30M cells, ahout 80 sgRNAs will be mis-estimated.

Propagation of the pooled library

See instructions for electroporation of a similar library:
https://www.addgene.org/pooled-library/yusa-crispr-knockout-mouse-v2/

General notes on methods for T cell extraction

We have compared Stem Cell Technologies, Miltenyi and a home-brew negative T cell selection protocol. Generally the home-brew protocol worked but found that screening is already sufficiently complicated to not involve additional steps. SCT has given more varying purity results than Miltenyi, possibly due to the extraction being at room temperature. You also obtain fewer T cells for the same purity level with SCT double-negative selection as opposed to Miltenyi negative-positive selection. However, the Miltenyi protocol does not scale well to the number of cells required for screening, and takes more time to execute even for small numbers of mice. Nowadays we use Miltenyi only for individual KO, if ever. Both Miltenyi and SCT recommend against using red blood cell lysis because the debris may clog cells in subsequent steps. However, we find that with proper care, especially the filter step afterwards to remove lipids, this need not be a problem. With the much reduced number of input cells, a smaller amount of antibodies must be used, or the negative selection will be too harsh.

CRISPR screening & KO on mouse T cells

This assumes that you get T cells from mice expressing *Cas9*. Avoid mice over 12w as the number of T-cells are reduced quite a bit, but these will also work. We use mainly 8-10w. Expect ~7M cells per spleen with the following protocol.

Plan to use 20-25 mice per screening, if 100% of the cells express the gene of interest. If fewer, scale up accordingly or perform a rescreening. Assuming an MOI of 20-30%, for 30M cells output cells, this means 100M-150M cells are needed on a bare minimum.

Day 0 or before – making of plates

Use 6w plates. Coat with 1µl/ml CD3 with 1.2ml per well. Put in 37°C for at least 2 hours or

overnight 4°C (and 3µl/ml CD28 if using the old protocol with both of these plate bound, see later remark)

Day 0 – extraction of T-cells

Idea: Use cold PBS in the beginning of the protocol to keep cells happy. Then use room-temp PBS when at SCT stage. Simple solution is take it out of fridge and just keep it on the bench, and let it warm up as you perform the protocol.

1. Take a 10cm plate and add up to 4-6 sets of 70µm sieves. In each sieve add 3 spleens. More spleens risk loss of T cells due to clogging. Add 10ml cold PBS in total over the spleens. Use the back of a syringe to macerate the spleens. I believe more spleens/sieve will reduce the viability of the cells. Unconfirmed rumors also has it that the back of a syringe is bad and that the front is better, but this is much slower in my hands. I use the hard side of the syringe to first cut the spleen in pieces, then the rubber side to macerate.

2. Add as much PBS as needed to wash off the cells and pass through a 70µm into a 50ml falcon. Reusing the 10cm plate might help in retaining cells.

3. Spin down 400g/5min and resuspend 3 spleens per 50ml falcon. Up to 4 spleens can be processed in only falcon, but cell loss may be higher.

4. Perform RBC lysis: vortex cells to resuspend. add 5ml 1x RBC lysis buffer (eBioscience, use and predilute the 10x as it is much cheaper than the 1x). Wait 4 min, then fill with cold PBS to 50ml. Spin down and remove supernatant.

5. Resuspend in any amount of PBS. Run all content through a 70µm filter. This removes lipids and other things that may reduce efficiency of antibody selection later.

6. Use the SCT kit: http://www.stemcell.com/en/Products/All-Products/EasySep-Mouse-CD4-T-Cell-Isolation-Kit.aspx . Note that THE MEDIA HAS TO BE ROOM TEMPERATURE or negative selection will fail. Changes from protocol:

a. Use 20% of the antibodies and 20% of the volume (if you use 50% you end up with purity similar to Miltenyi). Use MACS buffer supplemented with the rat serum from the kit.

b. CRUCIAL: do not put more than 6 spleens into one large magnet. That is, you need to dilute it more than the protocol says. For 20-25 spleens, use all 4 magnets. If you lose the cells in the magnet then resuspend the cells on the walls of the magnet in PBS and use the magnet again. Loss is lower with 15ml polypropylene tubes than with falcon tubes.

Day 0 – Plating T cells

Resuspend cells into IMDM with 1ul/ml BME, 1ul/ml CD28 and any IL* of choice. If using only IL-2 then 5ul/ml. It is possible that adding IL-2 might boost differentiation here but we don't do it anymore. Keeping the CD28 in the media might help budding cells which later will not have direct contact with plate surface, so it makes it more even.

For a 6w plate, aim for 40M cells per plate (ready next day). Do not by any means go below 20M. 30M can be a compromise. This is a step we think deserves further optimization as other groups have reported higher infection efficiency with lower plating density.

Day 1 – Transduction

If cells are floating, remove media, spin them down and add them in new media without stirring the cells already attached to the plate too much. I use 6ml media/plate. Note that this is pushing the

limit for the amount of media/well for 6w, and you will see some cells detach. The problem seems particularly bad the more plates you stack. I resuspend the cells lightly after centrifugation to make up for this

The 1x media consists of

Polybrene	8µg/ml
Virus	80%
IMDM with FBS	20%
IL-2	5µg/ml
Other cytokines to induce your T helper type of choice, such as IL-4	

If you are doing targeted infections for 96 well-plates then it is better to make a stock solution of medium that is 5x, and add 160µl virus + 40µl medium per well. The 5x medium (IMDM+FBS) would contain:

Polybrene	40µg/ml
IMDM with FBS	20%
IL-2	25µg/ml
Other cytokines * 5	

Put plates into suitable centrifuge preheated to 32°C. Having it at 32°C keeps the cells happy while the virus is degraded slower (this has been published before, and is called “cold infection”, but we have not verified it to be better). Several 6/96 well plates can normally be stack in a centrifuge. However, if too many plates are stacked, we noted that the angle is off. This can be a problem if you centrifuge with a small amount of virus media.

Spin for 2h at 1000-1100g. Put the plates in zip-lock bags or too much liquid will evaporate. It might be beneficial to work with 12 well-plates here to also reduce use of virus but we have in practice gotten stuck on using 6 well-plates out of habit. Put the plates for up to 16h in 32°C incubation. One paper says that “extended centrifugation does not improve efficiency” but we have not tested the influence of this. It is possible to get infected cells even if you skip the incubation after the spinning, but this is not recommended.

Resuspend cells in IMDM with differentiating cytokine of choice, BME 1µg/ml and 1µg/ml CD28. Keep cells in at least 2ml/well, possibly 3ml.

Day 2 - Optionally transduct again

Redo infection if you wish. Approximately doubles the efficiency according to others. We have not verified this.

Day 2 – Puromycin selection

This is to be done on the day after transduction. 24h after spinning, change the media of the cells (IL*, BME, CD28) but now in addition 2µg/ml puromycin. For each well, use at least 3ml media as the cells are depleting it very fast.

We have estimated kill rates and think anywhere 2-3µg/ml is suitable. We once did a quick experiment and got the following kill rates: With 2.5µg/ml puromycin, 38% of the cells were transduced on day 5. With 5µg/ml, 53% of the cells were transduced.

Day 3 – Media change

It is likely that another media change is needed here too, same composition as the day before. But so many cells will already have died that the media change need will be much reduced from here on

Day 4, 5 or 6 – Dead cell removal and FACS

The choice of day depends on our your read-out. See other protocols where you may instead choose to let the cells rest off CD3/CD28, for a later restimulation. This will provide you with more cells which may help get enough material. To be able to FACS sort enough cells, you need to increase the number of viable cells. We have experimented with several methods. Do not under any circumstance use Miltenyi dead cell removal kit. In our only trial experiment removed almost all the transduced cells. We speculate that transduced T cells express Annexin, which is what the dead cell removal kit detects.

Dead cell removal with low-g spin

This is simplest and recommended methods, although many viable cells will also be lost. Simply centrifuge the cells at 120g for 5 minutes. Remove (but save!) the supernatant. Check the viability of the pellet. If you lost too many cells you can respin the supernatant. Further optimization may be needed to get your optimal ratio. We get about 60% viable cells after this step, but lose up to 50%. Avoid spinning or pipetting too many times as this may in itself reduce the viability of the cells.

Dead cell removal with Ficoll

This method also works, but is difficult to perform with large number of cells. It is very easy to accidentally mix the phase during the pipetting. Either way, you can do 200M cells per tube at least. It is possible to repurify the dead fraction to get more cells although not a great number. It is possible that washing the dead fraction better, to really get rid of the Ficoll, will improve the yield the second time

1. In a 10ml falcon, add up to 7ml media with cells.
2. Use a repeat dispenser to add 5ml ficoll at the bottom of the tube. Pipette boys are too fast and cause mixing of the phases.
3. Spin for 400g, 20C, acceleration 6 (or full), no brake, for possible 20min. Manual suggests 40min. Expect 50% of the live cells to be lost but since they have undergone clonal expansion this might not be a huge problem

Fix & stain, depending on your protocol, or FACS-sort directly. Your FACS sorter may handle up to 40M cells/ml. To reduce clogging of the cells we recommend adding 3mM EDTA. Note that while you ideally should sort only BFP+ (transduced) cells, this is not technically required. Thus if your fixation protocol kills the BFP signal, you can still sort “blindly”.

Ideally same day as FACS: Genomic PCR extraction

Use the blood & cell culture DNA midi-kit instead of the maxi-kit. The midi kit is enough for 20M cells.

If possible, start protocol on the same day right after FACS. The blood & cell culture kit claims if you do step “1-5” before freezing the sample, the extraction will be more efficient.

For fixed cells, see our paper methods for details. Qiagen support recommends using their FFPE kit instead for fixed cells, but it doesn't scale for our large number of cells. We have not investigated this but there is a chance that any fixed protein can get in the way of the polymerases during PCR. Thus, even if you see an amplified product, you might have lost complexity. We thus take care to undo the crosslinking carefully.

Amplification and sequencing of sgRNA inserts

To perform this step you will need in particular Q5 Hot Start High-Fidelity 2× Master Mix (NEB,

#M0494L), KAPA HiFi HotStart ReadyMix (Kapa, #KK2602) and Ampure XP beads (Beckman Coulter, #A63882). A Bioanalyzer is recommended for QC. Further order the following oligos as PAGE-purified, 1 µmol scale synthesis:

- Insert forward primer, gLibrary-HiSeq_50bp-SE-U1:
ACACTCTTTCCCTACACGACGCTCTTCCGATCTCTTGTGGAAAGGACGAAACA

- Insert reverse primer, gLibrary-HiSeq_50bp-SE-L1:
TCGGCATTCCTGCTGAACCGCTCTTCCGATCTCTAAAGCGCATGCTCCAGAC

- U6-Illumina-seq2 (19bp-SE):
TCTTCCGATCTCTTGTGGAAAGGACGAAACACCG

Further you will need index primers PE1.0 and any number of iPCRtag*. In principle only one iPCRtag is needed, depending on your multiplexing requirements. See http://europepmc.org/articles/PMC3849550 for a list of primers.

1. **Insert amplification:** Set up the following PCR reaction: Q5 25µl, 5µM insert forward and reverse primers 1µl + 1µl, 5µg genomic DNA, then nuclease free water up to a total of 50µl. Set up as many reactions as required to use the isolated genomic DNA. Then run PCR as follows:

- Cycle 1: 30s @ 98°C
- Cycle 2-26: 10s @ 98°C. 15s @ 61°C. 20s @ 72°C
- Cycle 27: 2min @ 72°C

An even better way is to run 15 cycles of this program. Take out 5µl (or pool 1µl from multiple wells), and put the remainder on ice. Run qPCR on this small aliquot for 15 more cycles (we use EvaGreen). Pick a suitable number of cycles before saturation is reached, take out the original PCR reaction on ice, and run to completion.

2. From each well, extract 5µl and pool. Easiest is to pipette into a reservoir with PB buffer. Aim for 10 times more PB buffer than PCR product. Then use a Qiagen PCR cleanup column and elute in 50µl NFW (nuclease free water). The genomic DNA will not pass through the column filter. Verify product on a bioanalyzer, high sensitivity DNA kit. You can use a nanodrop to avoid saturating the chip (dilute an aliquot as needed)

3. **Library prep:** Dilute the product to 200 pg/µl and set up PCR mix: 2xKAPA 25µl, 5µM PE1.0 1ul, 5µM iPCRtag* 1µl, insert PCR product 5µl, NFW 18µl. Run the following program:

- Cycle 1: 30s @ 98°C
- Cycle 2-9: 10s @ 98°C. 15s @ 66°C. 20s @ 72°C
- Cycle 10: 5min @ 72°C

4. Perform an Ampure bead SPRI cleanup with ratio 0.7, according to the manual. Dilute to concentration suited for your Illumina sequencer. We use the average of 2-3 bioanalyzer measurements to calculate the concentration and find this to be precise.

5. Sequence with the custom sequencing primer, U6-Illumina-seq2 (100µM), 19 bp single-ended. Expect to need 37.5µl (2 lanes HiSeq Rapid Run) or 4µl (1 lane MiSeq). You will need to supply the chosen iPCRtag* indices for the demultiplexing of the reads.
