# Supplementary material for: Genome-wide CRISPR Screens in T Helper Cells Reveal Pervasive Crosstalk between Activation and Differentiation
Source: Cell. 2019 Feb 7;176(4):882–896.e18. doi: 10.1016/j.cell.2018.11.044 (PMC6370901; doi:10.1016/j.cell.2018.11.044)
Supplement: Data S2. Processed Data from All the Steps of the Analysis, Related to Figure 1 [file mmc2.zip › supplemental data/motif analysis/Stat6_SRX021632_homer/homerResults.html]

stat6\_motifs/ - Homer de novo Motif Results


# Homer *de novo* Motif Results (stat6\_motifs/)

Known Motif Enrichment Results  
Gene Ontology Enrichment Results  
If Homer is having trouble matching a motif to a known motif, try copy/pasting the matrix file into
STAMP  
More information on motif finding results: HOMER
| Description of Results
| Tips
  
Total target sequences = 25277  
Total background sequences = 24161  
\* - possible false positive  

|  |  |  |  |  |  |  |  |  |
| --- | --- | --- | --- | --- | --- | --- | --- | --- |
| Rank | Motif | P-value | log P-pvalue | % of Targets | % of Background | STD(Bg STD) | Best Match/Details | Motif File |
| 1 | C A T G T C G A G C A T A C T G G C T A T A G C C G A T T G A C G C T A A G C T | 1e-2611 | -6.014e+03 | 25.05% | 4.73% | 43.5bp (64.0bp) | BATF(bZIP)/Th17-BATF-ChIP-Seq(GSE39756)/Homer(0.997) More Information | Similar Motifs Found | motif file (matrix) |
| 2 | C A T G G T A C A G T C G T A C A G T C A G T C G C A T C T A G A T C G G C A T A C T G C A T G | 1e-1585 | -3.650e+03 | 8.60% | 0.66% | 34.0bp (55.1bp) | BORIS(Zf)/K562-CTCFL-ChIP-Seq(GSE32465)/Homer(0.926) More Information | Similar Motifs Found | motif file (matrix) |
| 3 | C T G A T A G C T G A C T C A G T C A G T G C A C G T A T C A G G A C T A T C G | 1e-1477 | -3.403e+03 | 33.11% | 12.96% | 48.4bp (63.3bp) | Fli1(ETS)/CD8-FLI-ChIP-Seq(GSE20898)/Homer(0.979) More Information | Similar Motifs Found | motif file (matrix) |
| 4 | G C A T G A C T T G A C G A C T G A T C C T A G A C T G C T G A C T G A T G C A | 1e-936 | -2.157e+03 | 17.23% | 5.59% | 42.7bp (60.3bp) | Stat5a::Stat5b/MA0519.1/Jaspar(0.942) More Information | Similar Motifs Found | motif file (matrix) |
| 5 | C A T G G A C T C G A T A C T G G C A T C T A G A C T G A G C T G A C T C G A T | 1e-654 | -1.506e+03 | 14.94% | 5.52% | 49.8bp (61.8bp) | RUNX1(Runt)/Jurkat-RUNX1-ChIP-Seq(GSE29180)/Homer(0.985) More Information | Similar Motifs Found | motif file (matrix) |
| 6 | A T C G T C A G A G C T T A G C G T C A G A T C T C A G A C G T T A C G T C G A | 1e-576 | -1.328e+03 | 8.40% | 2.21% | 28.9bp (69.3bp) | USF1(bHLH)/GM12878-Usf1-ChIP-Seq(GSE32465)/Homer(0.974) More Information | Similar Motifs Found | motif file (matrix) |
| 7 | C T G A C T A G C A T G C T A G A C T G A C T G G A T C C T A G A C T G C T A G T C A G G T A C | 1e-458 | -1.056e+03 | 17.10% | 8.16% | 54.0bp (58.8bp) | Sp1(Zf)/Promoter/Homer(0.966) More Information | Similar Motifs Found | motif file (matrix) |
| 8 | T G C A A G T C C T G A A T C G G A T C C T A G T A G C T A G C G T A C G A T C G T A C C G A T | 1e-306 | -7.063e+02 | 2.27% | 0.28% | 38.3bp (67.7bp) | BORIS(Zf)/K562-CTCFL-ChIP-Seq(GSE32465)/Homer(0.744) More Information | Similar Motifs Found | motif file (matrix) |
| 9 | C A T G C T A G C T A G C G T A C T G A C G A T A G C T A T C G A C G T C T G A C T A G A G C T | 1e-264 | -6.081e+02 | 1.71% | 0.18% | 47.4bp (57.3bp) | GFY(?)/Promoter/Homer(0.969) More Information | Similar Motifs Found | motif file (matrix) |
| 10 | T A G C T C G A G C A T A C T G C T G A A G T C C T A G A G C T G T A C C G T A | 1e-220 | -5.072e+02 | 7.10% | 3.09% | 49.2bp (67.9bp) | Atf7(bZIP)/3T3L1-Atf7-ChIP-Seq(GSE56872)/Homer(0.976) More Information | Similar Motifs Found | motif file (matrix) |
| 11 | G T C A A G T C A C G T A G T C A G T C A G T C C G T A A G T C | 1e-202 | -4.661e+02 | 11.71% | 6.50% | 54.0bp (61.4bp) | Egr2(Zf)/Thymocytes-Egr2-ChIP-Seq(GSE34254)/Homer(0.826) More Information | Similar Motifs Found | motif file (matrix) |
| 12 | G T C A A C T G C G T A A C G T C G T A C G T A T C A G T G C A | 1e-191 | -4.400e+02 | 13.41% | 7.94% | 54.1bp (60.6bp) | GATA3/MA0037.2/Jaspar(0.997) More Information | Similar Motifs Found | motif file (matrix) |
| 13 | T C A G A T C G G A C T A C G T G T A C G C A T C A T G G A T C C G T A A T C G G T C A C T G A | 1e-125 | -2.890e+02 | 3.87% | 1.63% | 44.5bp (62.7bp) | STAT6(Stat)/CD4-Stat6-ChIP-Seq(GSE22104)/Homer(0.780) More Information | Similar Motifs Found | motif file (matrix) |
| 14 | C T A G A G T C A G T C C G T A C G T A A C G T T A G C T C G A A T C G T G C A | 1e-124 | -2.872e+02 | 6.78% | 3.65% | 55.1bp (63.5bp) | NFY(CCAAT)/Promoter/Homer(0.953) More Information | Similar Motifs Found | motif file (matrix) |
| 15 | T C A G C T G A C T G A G C T A T A C G G C A T T C A G C G T A C T G A G C T A T A C G G A C T | 1e-107 | -2.483e+02 | 2.85% | 1.11% | 52.6bp (66.9bp) | IRF1(IRF)/PBMC-IRF1-ChIP-Seq(GSE43036)/Homer(0.981) More Information | Similar Motifs Found | motif file (matrix) |
| 16 | A C T G A T C G C T G A C G T A G C T A C G A T G A T C G T A C A G T C A T G C | 1e-86 | -1.998e+02 | 2.23% | 0.86% | 52.2bp (56.7bp) | MF0003.1\_REL\_class/Jaspar(0.982) More Information | Similar Motifs Found | motif file (matrix) |
| 17 | C T A G G T A C C T A G A G T C G T C A A C G T A C T G A G T C C A T G A G T C T G C A C T G A | 1e-85 | -1.979e+02 | 6.02% | 3.52% | 53.9bp (72.3bp) | NRF(NRF)/Promoter/Homer(0.962) More Information | Similar Motifs Found | motif file (matrix) |
| 18 | C G T A A T C G C G T A A C G T A T C G A G T C C G A T C G T A A C G T A G T C | 1e-49 | -1.145e+02 | 0.34% | 0.04% | 46.9bp (49.9bp) | GATA(Zf),IR3/iTreg-Gata3-ChIP-Seq(GSE20898)/Homer(0.816) More Information | Similar Motifs Found | motif file (matrix) |
| 19 | T G C A G C T A A C G T T G A C G C A T A T G C A T C G G A T C T C A G T G C A | 1e-38 | -8.929e+01 | 0.93% | 0.34% | 52.5bp (64.5bp) | GFX(?)/Promoter/Homer(0.840) More Information | Similar Motifs Found | motif file (matrix) |
| 20 | A G T C T C G A A G C T C G T A A G C T A C G T A G T C G A T C G C T A A G T C T A C G C A G T | 1e-35 | -8.112e+01 | 0.13% | 0.01% | 52.4bp (18.8bp) | NFAT(RHD)/Jurkat-NFATC1-ChIP-Seq(Jolma\_et\_al.)/Homer(0.666) More Information | Similar Motifs Found | motif file (matrix) |
