# Supplementary material for: Genome-wide CRISPR Screens in T Helper Cells Reveal Pervasive Crosstalk between Activation and Differentiation
Source: Cell. 2019 Feb 7;176(4):882–896.e18. doi: 10.1016/j.cell.2018.11.044 (PMC6370901; doi:10.1016/j.cell.2018.11.044)
Supplement: Data S2. Processed Data from All the Steps of the Analysis, Related to Figure 1 [file mmc2.zip › supplemental data/motif analysis/Stat6_SRX021632_homer/knownResults.html]

stat6\_motifs - Homer Known Motif Enrichment Results


# Homer Known Motif Enrichment Results (stat6\_motifs)

Homer *de novo* Motif Results  
Gene Ontology Enrichment Results  
Known Motif Enrichment Results (txt file)  
Total Target Sequences = 25281, Total Background Sequences = 24165

|  |  |  |  |  |  |  |  |  |  |  |  |
| --- | --- | --- | --- | --- | --- | --- | --- | --- | --- | --- | --- |
| Rank | Motif | Name | P-value | log P-pvalue | q-value (Benjamini) | # Target Sequences with Motif | % of Targets Sequences with Motif | # Background Sequences with Motif | % of Background Sequences with Motif | Motif File | SVG |
| 1 | C A T G C T A G T C G A A C G T A C T G C G T A T A G C C G A T T G A C C G T A A G C T G A T C | Fra2(bZIP)/Striatum-Fra2-ChIP-Seq(GSE43429)/Homer | 1e-2598 | -5.983e+03 | 0.0000 | 5711.0 | 22.59% | 908.6 | 3.76% | motif file (matrix) | svg |
| 2 | A C T G C T A G T C G A C G A T C A T G G C T A A T C G C G A T G T A C G C T A A G C T G T A C | Fra1(bZIP)/BT549-Fra1-ChIP-Seq(GSE46166)/Homer | 1e-2581 | -5.945e+03 | 0.0000 | 6052.0 | 23.94% | 1045.3 | 4.33% | motif file (matrix) | svg |
| 3 | C A G T T G C A A C G T A C T G C G T A A T C G C G A T T G A C C G T A A C G T | BATF(bZIP)/Th17-BATF-ChIP-Seq(GSE39756)/Homer | 1e-2564 | -5.905e+03 | 0.0000 | 6574.0 | 26.00% | 1266.4 | 5.25% | motif file (matrix) | svg |
| 4 | C T A G T C G A A C G T A C T G C G T A A T G C A C G T G T A C C G T A A G C T G A T C G T A C | Atf3(bZIP)/GBM-ATF3-ChIP-Seq(GSE33912)/Homer | 1e-2560 | -5.897e+03 | 0.0000 | 6640.0 | 26.26% | 1296.2 | 5.37% | motif file (matrix) | svg |
| 5 | C T A G T C G A G C A T C A T G G C T A T A G C C G A T G T A C C T G A A G C T | JunB(bZIP)/DendriticCells-Junb-ChIP-Seq(GSE36099)/Homer | 1e-2526 | -5.819e+03 | 0.0000 | 6110.0 | 24.17% | 1094.1 | 4.53% | motif file (matrix) | svg |
| 6 | T C G A A C G T C A T G G C T A T A G C C G A T G T A C G C T A A C G T A T G C | AP-1(bZIP)/ThioMac-PU.1-ChIP-Seq(GSE21512)/Homer | 1e-2415 | -5.562e+03 | 0.0000 | 6822.0 | 26.98% | 1458.9 | 6.04% | motif file (matrix) | svg |
| 7 | C T A G T C G A C G A T A C T G C G T A T A C G A G C T T G A C G C T A A C G T G A T C T A G C | Fosl2(bZIP)/3T3L1-Fosl2-ChIP-Seq(GSE56872)/Homer | 1e-2373 | -5.466e+03 | 0.0000 | 4742.0 | 18.76% | 656.4 | 2.72% | motif file (matrix) | svg |
| 8 | C T A G T C G A A C G T A C T G C G T A T A G C C G A T G T A C C G T A A G C T G A T C G T A C | Jun-AP1(bZIP)/K562-cJun-ChIP-Seq(GSE31477)/Homer | 1e-2141 | -4.932e+03 | 0.0000 | 3884.0 | 15.36% | 463.3 | 1.92% | motif file (matrix) | svg |
| 9 | T G C A A G C T C T G A A T C G G A C T C T A G G T A C G A T C G T C A A G T C G T A C G A C T C T A G A T C G G C A T C A T G C A T G G A T C G T A C C T G A | CTCF(Zf)/CD4+-CTCF-ChIP-Seq(Barski\_et\_al.)/Homer | 1e-2109 | -4.858e+03 | 0.0000 | 3367.0 | 13.32% | 328.0 | 1.36% | motif file (matrix) | svg |
| 10 | T A G C A G T C T G A C A G T C C T A G A T C G A G T C C A T G T G A C A G T C G T A C A G T C A G T C G C A T C T A G A T C G G C A T A C T G A T C G G A T C | BORIS(Zf)/K562-CTCFL-ChIP-Seq(GSE32465)/Homer | 1e-1577 | -3.632e+03 | 0.0000 | 3684.0 | 14.57% | 606.3 | 2.51% | motif file (matrix) | svg |
| 11 | A G T C C T G A A G T C C G A T C A G T G A T C A T G C A C T G A T C G G A C T | Fli1(ETS)/CD8-FLI-ChIP-Seq(GSE20898)/Homer | 1e-1506 | -3.468e+03 | 0.0000 | 8471.0 | 33.51% | 3159.3 | 13.09% | motif file (matrix) | svg |
| 12 | T C G A T A G C T G C A A C T G A C T G C G T A C G T A C T A G G A C T T A C G | ETS1(ETS)/Jurkat-ETS1-ChIP-Seq(GSE17954)/Homer | 1e-1382 | -3.182e+03 | 0.0000 | 7601.0 | 30.07% | 2758.5 | 11.43% | motif file (matrix) | svg |
| 13 | T C G A T A G C G T C A A C T G A C T G C G T A C G T A C T A G A G C T T C A G | ERG(ETS)/VCaP-ERG-ChIP-Seq(GSE14097)/Homer | 1e-1310 | -3.018e+03 | 0.0000 | 9366.0 | 37.05% | 4027.3 | 16.68% | motif file (matrix) | svg |
| 14 | G A C T C A G T G A T C G A T C A C G T G A T C C T G A T A C G C G T A G T C A | STAT6(Stat)/Macrophage-Stat6-ChIP-Seq(GSE38377)/Homer | 1e-1285 | -2.960e+03 | 0.0000 | 4316.0 | 17.07% | 1036.7 | 4.29% | motif file (matrix) | svg |
| 15 | A T G C A G T C C T G A A G T C C G A T A C G T A G T C A G T C A C G T A T C G G A C T A C G T | Etv2(ETS)/ES-ER71-ChIP-Seq(GSE59402)/Homer(0.967) | 1e-1280 | -2.948e+03 | 0.0000 | 6694.0 | 26.48% | 2321.1 | 9.61% | motif file (matrix) | svg |
| 16 | T C G A C T G A T A G C T G A C T C A G T C A G C G T A C G T A T C A G A G C T | ETV1(ETS)/GIST48-ETV1-ChIP-Seq(GSE22441)/Homer | 1e-1215 | -2.798e+03 | 0.0000 | 8826.0 | 34.91% | 3793.7 | 15.71% | motif file (matrix) | svg |
| 17 | C T G A A G C T A C G T A C G T A G T C G A C T G A C T C T G A C T G A C T A G C G T A C G T A | STAT6(Stat)/CD4-Stat6-ChIP-Seq(GSE22104)/Homer | 1e-1206 | -2.777e+03 | 0.0000 | 4061.0 | 16.06% | 973.6 | 4.03% | motif file (matrix) | svg |
| 18 | T C G A T C G A T A G C G T A C T C A G T A C G C G T A C G T A T C A G A G C T | GABPA(ETS)/Jurkat-GABPa-ChIP-Seq(GSE17954)/Homer | 1e-1171 | -2.698e+03 | 0.0000 | 6810.0 | 26.94% | 2521.4 | 10.44% | motif file (matrix) | svg |
| 19 | C G A T T A C G T G A C G A C T C A T G C G T A T A C G A C G T G T A C C T G A | Bach2(bZIP)/OCILy7-Bach2-ChIP-Seq(GSE44420)/Homer | 1e-1026 | -2.364e+03 | 0.0000 | 2365.0 | 9.35% | 374.9 | 1.55% | motif file (matrix) | svg |
| 20 | G A T C T C G A A G T C C G A T C G A T A G T C A T G C A C T G A T C G G A C T | Elk1(ETS)/Hela-Elk1-ChIP-Seq(GSE31477)/Homer | 1e-899 | -2.071e+03 | 0.0000 | 5185.0 | 20.51% | 1865.3 | 7.73% | motif file (matrix) | svg |
| 21 | T G C A C T G A A G T C G T C A A C T G A C T G C G T A C G T A C T G A A G C T | EWS:FLI1-fusion(ETS)/SK\_N\_MC-EWS:FLI1-ChIP-Seq(SRA014231)/Homer | 1e-885 | -2.040e+03 | 0.0000 | 4591.0 | 18.16% | 1533.9 | 6.35% | motif file (matrix) | svg |
| 22 | G A T C C T G A A G T C C G A T C G A T G A T C A G T C A C T G A T C G A G C T | Elk4(ETS)/Hela-Elk4-ChIP-Seq(GSE31477)/Homer | 1e-878 | -2.022e+03 | 0.0000 | 5142.0 | 20.34% | 1865.8 | 7.73% | motif file (matrix) | svg |
| 23 | T C G A A G C T A C G T A C G T A G T C A G T C A C G T A T C G G A C T A T C G | EWS:ERG-fusion(ETS)/CADO\_ES1-EWS:ERG-ChIP-Seq(SRA014231)/Homer | 1e-856 | -1.972e+03 | 0.0000 | 4398.0 | 17.40% | 1456.4 | 6.03% | motif file (matrix) | svg |
| 24 | C T G A T G C A T A G C T G A C T A C G T C A G C T G A G C T A T C A G G A C T | ELF1(ETS)/Jurkat-ELF1-ChIP-Seq(SRA014231)/Homer | 1e-803 | -1.850e+03 | 0.0000 | 4653.0 | 18.41% | 1665.2 | 6.90% | motif file (matrix) | svg |
| 25 | T G C A C T G A A T G C G T C A A C T G A C T G C G T A C G T A C T A G A G C T | Ets1-distal(ETS)/CD4+-PolII-ChIP-Seq(Barski\_et\_al.)/Homer | 1e-759 | -1.750e+03 | 0.0000 | 2745.0 | 10.86% | 684.7 | 2.84% | motif file (matrix) | svg |
| 26 | T G C A T C G A T A G C G T A C T C A G C T A G G T C A G C T A T C A G G A C T | ETS(ETS)/Promoter/Homer | 1e-722 | -1.663e+03 | 0.0000 | 3517.0 | 13.91% | 1110.4 | 4.60% | motif file (matrix) | svg |
| 27 | C G T A T A G C T A G C T G C A A C T G C T A G C G T A C G T A T C A G G A C T | EHF(ETS)/LoVo-EHF-ChIP-Seq(GSE49402)/Homer | 1e-708 | -1.632e+03 | 0.0000 | 6711.0 | 26.55% | 3155.1 | 13.07% | motif file (matrix) | svg |
| 28 | C T G A A C G T A C G T A C G T A G T C G A C T C G A T C T G A A C T G C G T A C G T A T C G A | STAT5(Stat)/mCD4+-Stat5-ChIP-Seq(GSE12346)/Homer | 1e-561 | -1.294e+03 | 0.0000 | 2315.0 | 9.16% | 637.3 | 2.64% | motif file (matrix) | svg |
| 29 | C G T A T G A C T A G C T G C A A C T G A C T G C G T A C G T A T C A G G A C T | ELF3(ETS)/PDAC-ELF3-ChIP-Seq(GSE64557)/Homer | 1e-543 | -1.252e+03 | 0.0000 | 4092.0 | 16.19% | 1676.8 | 6.94% | motif file (matrix) | svg |
| 30 | A T G C C T G A G A C T A C G T A C G T G T A C G A T C C G A T C T A G C A T G C G T A C G T A C T G A G A C T | STAT1(Stat)/HelaS3-STAT1-ChIP-Seq(GSE12782)/Homer | 1e-532 | -1.225e+03 | 0.0000 | 1999.0 | 7.91% | 509.2 | 2.11% | motif file (matrix) | svg |
| 31 | G C T A C T G A T C G A A G T C A G T C C T G A A G T C G T C A C T G A T G C A | RUNX1(Runt)/Jurkat-RUNX1-ChIP-Seq(GSE29180)/Homer | 1e-531 | -1.224e+03 | 0.0000 | 5112.0 | 20.22% | 2372.6 | 9.83% | motif file (matrix) | svg |
| 32 | T C A G A C G T A G T C T C G A A G T C T C A G G C A T C T A G C T A G A G C T | Usf2(bHLH)/C2C12-Usf2-ChIP-Seq(GSE36030)/Homer | 1e-525 | -1.211e+03 | 0.0000 | 2471.0 | 9.77% | 750.8 | 3.11% | motif file (matrix) | svg |
| 33 | C G T A T A C G T C G A A C T G A C T G C G T A C G T A T A C G A G C T T A C G | PU.1(ETS)/ThioMac-PU.1-ChIP-Seq(GSE21512)/Homer | 1e-506 | -1.165e+03 | 0.0000 | 3228.0 | 12.77% | 1198.2 | 4.96% | motif file (matrix) | svg |
| 34 | T A G C G C T A T C G A C T G A A G T C A G T C C T G A A G T C C G T A C T A G | RUNX(Runt)/HPC7-Runx1-ChIP-Seq(GSE22178)/Homer | 1e-467 | -1.076e+03 | 0.0000 | 3926.0 | 15.53% | 1692.4 | 7.01% | motif file (matrix) | svg |
| 35 | T A C G T C A G A G C T A T G C C G T A A G T C T C A G A C G T A C T G T C G A | USF1(bHLH)/GM12878-Usf1-ChIP-Seq(GSE32465)/Homer | 1e-456 | -1.051e+03 | 0.0000 | 3092.0 | 12.23% | 1184.2 | 4.90% | motif file (matrix) | svg |
| 36 | A C T G G A T C G A C T A C T G A C G T C A T G A C T G A C G T A G C T C G A T | RUNX-AML(Runt)/CD4+-PolII-ChIP-Seq(Barski\_et\_al.)/Homer | 1e-454 | -1.046e+03 | 0.0000 | 3736.0 | 14.78% | 1590.1 | 6.59% | motif file (matrix) | svg |
| 37 | C T G A A T G C C G T A A C G T A G T C A G T C A C G T A C T G A T C G G C A T | SPDEF(ETS)/VCaP-SPDEF-ChIP-Seq(SRA014231)/Homer | 1e-436 | -1.004e+03 | 0.0000 | 5173.0 | 20.46% | 2608.4 | 10.80% | motif file (matrix) | svg |
| 38 | G C T A A G T C T A C G T G C A A T C G T C A G G C T A T C G A T C A G A G C T | ELF5(ETS)/T47D-ELF5-ChIP-Seq(GSE30407)/Homer | 1e-424 | -9.783e+02 | 0.0000 | 3971.0 | 15.71% | 1796.3 | 7.44% | motif file (matrix) | svg |
| 39 | T C A G A G C T A T G C C G T A A G T C T C A G A C G T A T C G T C G A A G T C G A T C T G A C | TFE3(bHLH)/MEF-TFE3-ChIP-Seq(GSE75757)/Homer | 1e-420 | -9.684e+02 | 0.0000 | 1119.0 | 4.43% | 204.8 | 0.85% | motif file (matrix) | svg |
| 40 | C T G A T C A G C A G T C T A G A C T G C T A G G A T C A T C G A C T G C T G A T C A G G A T C | Sp5(Zf)/mES-Sp5.Flag-ChIP-Seq(GSE72989)/Homer | 1e-419 | -9.670e+02 | 0.0000 | 6265.0 | 24.78% | 3454.8 | 14.31% | motif file (matrix) | svg |
| 41 | A T G C A T G C A T C G T A C G A G C T A G T C G C T A A G T C T C A G G A C T A C T G T C G A | E-box(bHLH)/Promoter/Homer | 1e-410 | -9.444e+02 | 0.0000 | 1110.0 | 4.39% | 206.6 | 0.86% | motif file (matrix) | svg |
| 42 | T C A G A G C T A C G T A C G T G T A C G A T C C G T A C T A G C A T G G T C A C G T A T C G A | STAT4(Stat)/CD4-Stat4-ChIP-Seq(GSE22104)/Homer | 1e-403 | -9.300e+02 | 0.0000 | 3959.0 | 15.66% | 1827.4 | 7.57% | motif file (matrix) | svg |
| 43 | T G A C G C T A T C G A T G C A A G T C A G T C C G T A A G T C C G T A C T G A G C T A G T A C | RUNX2(Runt)/PCa-RUNX2-ChIP-Seq(GSE33889)/Homer | 1e-403 | -9.292e+02 | 0.0000 | 4200.0 | 16.61% | 1992.2 | 8.25% | motif file (matrix) | svg |
| 44 | T C G A T A G C G T C A A C T G C T A G C G T A C G A T A C T G A C G T A C T G A C T G A C G T | ETS:RUNX(ETS,Runt)/Jurkat-RUNX1-ChIP-Seq(GSE17954)/Homer | 1e-396 | -9.137e+02 | 0.0000 | 1160.0 | 4.59% | 233.1 | 0.97% | motif file (matrix) | svg |
| 45 | T C A G A G C T A T G C C G T A A G C T T C A G C A G T A C T G C T G A A G T C | MITF(bHLH)/MastCells-MITF-ChIP-Seq(GSE48085)/Homer | 1e-396 | -9.133e+02 | 0.0000 | 4426.0 | 17.51% | 2163.3 | 8.96% | motif file (matrix) | svg |
| 46 | A T G C T C G A A G T C A G C T A C G T G T A C A G T C G C T A C T A G C A T G G T C A C T G A T C A G A G T C | Stat3+il21(Stat)/CD4-Stat3-ChIP-Seq(GSE19198)/Homer | 1e-392 | -9.036e+02 | 0.0000 | 3440.0 | 13.61% | 1503.5 | 6.23% | motif file (matrix) | svg |
| 47 | C T A G T C A G C A G T T C A G A C T G A C T G G A T C C T A G A C T G C T A G T C A G A T G C | KLF14(Zf)/HEK293-KLF14.GFP-ChIP-Seq(GSE58341)/Homer | 1e-375 | -8.638e+02 | 0.0000 | 8781.0 | 34.73% | 5600.8 | 23.20% | motif file (matrix) | svg |
| 48 | C T A G G C A T G A T C C G T A A G T C T C A G G A C T C T A G | CLOCK(bHLH)/Liver-Clock-ChIP-Seq(GSE39860)/Homer | 1e-358 | -8.245e+02 | 0.0000 | 3318.0 | 13.12% | 1484.4 | 6.15% | motif file (matrix) | svg |
| 49 | T A C G C T A G A T G C G A T C G T A C A G T C C T A G A G T C A G T C A G T C G T A C A G T C | Sp1(Zf)/Promoter/Homer | 1e-347 | -8.007e+02 | 0.0000 | 2907.0 | 11.50% | 1235.3 | 5.12% | motif file (matrix) | svg |
| 50 | G A C T T C A G C T A G A G T C A G T C G T A C A G T C C T G A A G T C A G T C A G T C G A C T A G T C A C T G A T G C | KLF3(Zf)/MEF-Klf3-ChIP-Seq(GSE44748)/Homer | 1e-328 | -7.568e+02 | 0.0000 | 3691.0 | 14.60% | 1794.6 | 7.43% | motif file (matrix) | svg |
| 51 | A G T C G A C T C A G T G T A C A G T C A T C G T C A G A C T G G T C A C G T A | Stat3(Stat)/mES-Stat3-ChIP-Seq(GSE11431)/Homer | 1e-315 | -7.267e+02 | 0.0000 | 2669.0 | 10.56% | 1137.3 | 4.71% | motif file (matrix) | svg |
| 52 | C A T G G T A C C G T A A G T C C T A G A C G T A C T G G T A C A G T C A G C T | bHLHE40(bHLH)/HepG2-BHLHE40-ChIP-Seq(GSE31477)/Homer | 1e-303 | -6.987e+02 | 0.0000 | 2185.0 | 8.64% | 855.3 | 3.54% | motif file (matrix) | svg |
| 53 | C G T A C G T A C G T A G C A T G C A T T A C G G T A C G A C T A C T G C G T A A T C G A C G T G T A C C G T A A G C T | Bach1(bZIP)/K562-Bach1-ChIP-Seq(GSE31477)/Homer | 1e-297 | -6.856e+02 | 0.0000 | 698.0 | 2.76% | 110.4 | 0.46% | motif file (matrix) | svg |
| 54 | C G T A C T A G A C T G A C T G G A C T C T A G C A G T C T A G C A T G G A T C | KLF5(Zf)/LoVo-KLF5-ChIP-Seq(GSE49402)/Homer | 1e-294 | -6.779e+02 | 0.0000 | 6885.0 | 27.23% | 4315.4 | 17.87% | motif file (matrix) | svg |
| 55 | A T G C A T C G T A C G A G C T A T C G C T G A A G T C C T A G A G C T A T G C C T G A A T G C | CRE(bZIP)/Promoter/Homer | 1e-282 | -6.497e+02 | 0.0000 | 1787.0 | 7.07% | 649.4 | 2.69% | motif file (matrix) | svg |
| 56 | T A C G T C G A C A G T A C T G G C T A A T G C C G A T G T A C C G T A A C T G T A G C C G T A | NF-E2(bZIP)/K562-NFE2-ChIP-Seq(GSE31477)/Homer | 1e-275 | -6.335e+02 | 0.0000 | 735.0 | 2.91% | 134.9 | 0.56% | motif file (matrix) | svg |
| 57 | T C A G C G T A A G T C A G C T C G T A A G T C C T G A C G T A A G T C G C A T A G T C A G T C A G T C C T G A A C T G T G C A T C G A C A T G A T C G G A T C | Ronin(THAP)/ES-Thap11-ChIP-Seq(GSE51522)/Homer | 1e-260 | -6.009e+02 | 0.0000 | 593.0 | 2.35% | 90.3 | 0.37% | motif file (matrix) | svg |
| 58 | G T A C C A G T A C T G A C T G A C T G G A T C A C T G A C G T A C T G A C T G A G T C G A T C | KLF6(Zf)/PDAC-KLF6-ChIP-Seq(GSE64557)/Homer | 1e-259 | -5.969e+02 | 0.0000 | 5525.0 | 21.85% | 3345.8 | 13.86% | motif file (matrix) | svg |
| 59 | G T C A G C A T A C T G G T A C G A C T A C T G G C T A A T C G C A G T G T A C C G T A A G C T | Nrf2(bZIP)/Lymphoblast-Nrf2-ChIP-Seq(GSE37589)/Homer | 1e-257 | -5.918e+02 | 0.0000 | 662.0 | 2.62% | 116.6 | 0.48% | motif file (matrix) | svg |
| 60 | T C G A T A G C T G A C C T G A A G T C A C T G G A C T C A T G | c-Myc(bHLH)/LNCAP-cMyc-ChIP-Seq(Unpublished)/Homer | 1e-254 | -5.865e+02 | 0.0000 | 3005.0 | 11.89% | 1478.1 | 6.12% | motif file (matrix) | svg |
| 61 | T C A G T A G C G A C T C A T G C T G A A T C G G C A T G T A C C G T A A C T G T A G C T G C A | MafK(bZIP)/C2C12-MafK-ChIP-Seq(GSE36030)/Homer | 1e-231 | -5.320e+02 | 0.0000 | 1509.0 | 5.97% | 556.1 | 2.30% | motif file (matrix) | svg |
| 62 | T A C G C T G A T C G A C G A T C T A G C T A G T C G A C T G A T C G A T C G A C G T A T C G A G C A T C A T G C G T A T A C G G C A T T G A C C G T A A G C T | NFAT:AP1(RHD,bZIP)/Jurkat-NFATC1-ChIP-Seq(Jolma\_et\_al.)/Homer | 1e-216 | -4.985e+02 | 0.0000 | 941.0 | 3.72% | 265.3 | 1.10% | motif file (matrix) | svg |
| 63 | A T G C T C A G T C G A G C A T A C T G C G T A A G T C T C A G G A C T T G A C C G T A A G C T | Atf2(bZIP)/3T3L1-Atf2-ChIP-Seq(GSE56872)/Homer | 1e-206 | -4.764e+02 | 0.0000 | 1619.0 | 6.40% | 657.5 | 2.72% | motif file (matrix) | svg |
| 64 | T G A C C T A G T C A G G T C A C G T A T C A G C G A T T C A G T C G A T G C A C T G A T A G C | PU.1-IRF(ETS:IRF)/Bcell-PU.1-ChIP-Seq(GSE21512)/Homer | 1e-206 | -4.756e+02 | 0.0000 | 4955.0 | 19.60% | 3075.0 | 12.74% | motif file (matrix) | svg |
| 65 | T C A G T G A C G T A C T G C A G T A C C T A G G T A C A T G C A G T C G T C A A G T C G A C T | Klf9(Zf)/GBM-Klf9-ChIP-Seq(GSE62211)/Homer | 1e-206 | -4.748e+02 | 0.0000 | 2856.0 | 11.30% | 1486.8 | 6.16% | motif file (matrix) | svg |
| 66 | A G C T A T G C G A C T G C A T C G T A A G C T G T A C C G A T A T C G A G T C | Gata6(Zf)/HUG1N-GATA6-ChIP-Seq(GSE51936)/Homer | 1e-203 | -4.695e+02 | 0.0000 | 2803.0 | 11.09% | 1454.8 | 6.03% | motif file (matrix) | svg |
| 67 | T A C G T C G A G A C T A C T G C T G A A G T C T C A G G A C T T G A C C T G A | Atf1(bZIP)/K562-ATF1-ChIP-Seq(GSE31477)/Homer | 1e-203 | -4.691e+02 | 0.0000 | 2658.0 | 10.51% | 1352.2 | 5.60% | motif file (matrix) | svg |
| 68 | T A G C C T A G T C G A G A C T A C T G C T G A A G T C T C A G G C A T T G A C C T G A A G C T | Atf7(bZIP)/3T3L1-Atf7-ChIP-Seq(GSE56872)/Homer | 1e-198 | -4.574e+02 | 0.0000 | 2093.0 | 8.28% | 976.5 | 4.04% | motif file (matrix) | svg |
| 69 | T A G C G C T A A C T G C G T A A C G T C G T A C G T A T A C G T C A G T C G A | Gata1(Zf)/K562-GATA1-ChIP-Seq(GSE18829)/Homer | 1e-196 | -4.521e+02 | 0.0000 | 1985.0 | 7.85% | 909.6 | 3.77% | motif file (matrix) | svg |
| 70 | T C G A C G T A A G T C A G C T C G T A A G T C T C G A G C T A G A C T C G A T A G T C A G T C A G T C C T G A T C A G T G C A T C G A C A G T A T C G A G T C | GFY-Staf(?,Zf)/Promoter/Homer | 1e-195 | -4.497e+02 | 0.0000 | 678.0 | 2.68% | 159.0 | 0.66% | motif file (matrix) | svg |
| 71 | T C G A T G A C A G T C C G T A A G T C C T A G A C G T A C T G A C T G A G C T A G T C G C A T | Max(bHLH)/K562-Max-ChIP-Seq(GSE31477)/Homer | 1e-192 | -4.425e+02 | 0.0000 | 3271.0 | 12.94% | 1825.3 | 7.56% | motif file (matrix) | svg |
| 72 | T A C G T C G A T A G C A G T C C G T A A G T C C T A G G C A T A C T G A T C G | n-Myc(bHLH)/mES-nMyc-ChIP-Seq(GSE11431)/Homer | 1e-190 | -4.389e+02 | 0.0000 | 3430.0 | 13.57% | 1948.2 | 8.07% | motif file (matrix) | svg |
| 73 | A G C T A G T C A T G C A G C T A C G T C G T A A C G T A G T C C G A T A T G C | Gata2(Zf)/K562-GATA2-ChIP-Seq(GSE18829)/Homer | 1e-188 | -4.340e+02 | 0.0000 | 2173.0 | 8.60% | 1051.8 | 4.36% | motif file (matrix) | svg |
| 74 | C T A G G T A C A G T C T G C A A G T C C T G A A G T C A G T C A G T C G C T A | Klf4(Zf)/mES-Klf4-ChIP-Seq(GSE11431)/Homer | 1e-187 | -4.321e+02 | 0.0000 | 2414.0 | 9.55% | 1219.8 | 5.05% | motif file (matrix) | svg |
| 75 | T G C A T A G C G A C T T G C A T G A C T G C A C G T A A G C T A G C T A G T C A G T C G T A C | GFY(?)/Promoter/Homer | 1e-185 | -4.260e+02 | 0.0000 | 650.0 | 2.57% | 154.3 | 0.64% | motif file (matrix) | svg |
| 76 | T A C G A T G C G C T A A C T G C G T A A C G T C G T A C T G A T A C G T C G A | Gata4(Zf)/Heart-Gata4-ChIP-Seq(GSE35151)/Homer | 1e-182 | -4.206e+02 | 0.0000 | 3011.0 | 11.91% | 1660.1 | 6.88% | motif file (matrix) | svg |
| 77 | G C T A A T C G G C T A G A C T G C T A T C G A T A G C T C G A | GATA3(Zf)/iTreg-Gata3-ChIP-Seq(GSE20898)/Homer | 1e-178 | -4.102e+02 | 0.0000 | 4114.0 | 16.27% | 2512.3 | 10.41% | motif file (matrix) | svg |
| 78 | C G T A C T G A C G T A C T A G T C G A C T A G A C T G C G T A C G T A T A C G A G C T A T C G | SpiB(ETS)/OCILY3-SPIB-ChIP-Seq(GSE56857)/Homer | 1e-172 | -3.971e+02 | 0.0000 | 1248.0 | 4.94% | 485.8 | 2.01% | motif file (matrix) | svg |
| 79 | T C G A A C G T A C T G C T G A A G T C T C A G A G C T G T A C C G T A A G C T G A T C T C G A | JunD(bZIP)/K562-JunD-ChIP-Seq/Homer | 1e-171 | -3.944e+02 | 0.0000 | 713.0 | 2.82% | 194.2 | 0.80% | motif file (matrix) | svg |
| 80 | T C G A G C A T A C T G C T G A A G T C T C A G G A C T G T A C C G T A A G C T A G T C G A T C | c-Jun-CRE(bZIP)/K562-cJun-ChIP-Seq(GSE31477)/Homer | 1e-163 | -3.776e+02 | 0.0000 | 1420.0 | 5.62% | 604.4 | 2.50% | motif file (matrix) | svg |
| 81 | G A C T C G A T T C A G G A T C G A C T A G C T A G C T A G T C G A T C C G T A C T A G C T A G T C G A T C G A C T G A | Bcl6(Zf)/Liver-Bcl6-ChIP-Seq(GSE31578)/Homer | 1e-141 | -3.262e+02 | 0.0000 | 4486.0 | 17.74% | 2949.5 | 12.22% | motif file (matrix) | svg |
| 82 | C T A G C A G T T G A C C G T A G A T C T C A G G A C T C A T G | BMAL1(bHLH)/Liver-Bmal1-ChIP-Seq(GSE39860)/Homer | 1e-136 | -3.139e+02 | 0.0000 | 6635.0 | 26.25% | 4775.7 | 19.78% | motif file (matrix) | svg |
| 83 | T C A G T C A G G C T A C G T A T A C G G A C T T C A G T C G A C T G A C G T A T A C G G A C T | IRF8(IRF)/BMDM-IRF8-ChIP-Seq(GSE77884)/Homer | 1e-133 | -3.073e+02 | 0.0000 | 1432.0 | 5.66% | 669.0 | 2.77% | motif file (matrix) | svg |
| 84 | C G A T T A C G G A T C G C T A T C A G G A C T C G A T G T A C G A T C G T C A T C G A T G A C C G T A C T A G G A C T C T A G C T A G G T A C A G T C C G T A | CTCF-SatelliteElement(Zf?)/CD4+-CTCF-ChIP-Seq(Barski\_et\_al.)/Homer | 1e-117 | -2.704e+02 | 0.0000 | 240.0 | 0.95% | 32.0 | 0.13% | motif file (matrix) | svg |
| 85 | A C G T T A C G G A T C A C T G A C G T C T A G A C T G A C T G G A T C C T A G C A T G C T A G | Egr2(Zf)/Thymocytes-Egr2-ChIP-Seq(GSE34254)/Homer | 1e-114 | -2.637e+02 | 0.0000 | 1308.0 | 5.17% | 626.8 | 2.60% | motif file (matrix) | svg |
| 86 | A T G C G A C T A G C T A G C T A G T C G C T A C A G T C G A T G C T A A C G T A C T G G C T A T A G C G C A T T G A C | IRF:BATF(IRF:bZIP)/pDC-Irf8-ChIP-Seq(GSE66899)/Homer | 1e-113 | -2.612e+02 | 0.0000 | 532.0 | 2.10% | 158.8 | 0.66% | motif file (matrix) | svg |
| 87 | C T A G A C T G C T A G T C A G T C A G T A C G C T A G A C T G | Maz(Zf)/HepG2-Maz-ChIP-Seq(GSE31477)/Homer | 1e-113 | -2.606e+02 | 0.0000 | 5767.0 | 22.81% | 4158.4 | 17.22% | motif file (matrix) | svg |
| 88 | A T G C C G T A A C T G C G T A A C G T G C T A T C G A A G C T C G A T C G T A A C G T A G T C C G A T A C T G G A T C | GATA(Zf),IR4/iTreg-Gata3-ChIP-Seq(GSE20898)/Homer | 1e-107 | -2.469e+02 | 0.0000 | 376.0 | 1.49% | 89.4 | 0.37% | motif file (matrix) | svg |
| 89 | A C T G T G A C G T A C C G T A A G T C T A C G A C G T A C T G G T C A A G T C | NPAS2(bHLH)/Liver-NPAS2-ChIP-Seq(GSE39860)/Homer | 1e-107 | -2.466e+02 | 0.0000 | 4258.0 | 16.84% | 2920.2 | 12.09% | motif file (matrix) | svg |
| 90 | C A G T T C A G G A T C A C T G A C G T C T A G A C T G A C T G G A C T C T A G | Egr1(Zf)/K562-Egr1-ChIP-Seq(GSE32465)/Homer | 1e-105 | -2.421e+02 | 0.0000 | 3476.0 | 13.75% | 2291.0 | 9.49% | motif file (matrix) | svg |
| 91 | C G A T T G C A G T A C C G T A A G T C C T A G G A C T C A T G | NPAS(bHLH)/Liver-NPAS-ChIP-Seq(GSE39860)/Homer | 1e-105 | -2.419e+02 | 0.0000 | 5791.0 | 22.91% | 4225.2 | 17.50% | motif file (matrix) | svg |
| 92 | C T A G T C G A C T G A C G T A T A C G G A C T T C A G T C G A G T C A T G C A T A C G A G C T | IRF2(IRF)/Erythroblas-IRF2-ChIP-Seq(GSE36985)/Homer | 1e-102 | -2.367e+02 | 0.0000 | 606.0 | 2.40% | 209.1 | 0.87% | motif file (matrix) | svg |
| 93 | C T G A C T G A C T A G T C G A C G T A A T G C C G T A A C T G C G T A A C G T C T G A C G A T A G C T C G T A A C G T A G T C C G A T T A C G G T C A G C A T | GATA(Zf),IR3/iTreg-Gata3-ChIP-Seq(GSE20898)/Homer | 1e-102 | -2.359e+02 | 0.0000 | 587.0 | 2.32% | 199.7 | 0.83% | motif file (matrix) | svg |
| 94 | G A C T C T A G G A T C C A G T A C T G C T G A A T G C G C A T A T G C C T G A | MafA(bZIP)/Islet-MafA-ChIP-Seq(GSE30298)/Homer | 1e-98 | -2.279e+02 | 0.0000 | 3115.0 | 12.32% | 2027.4 | 8.40% | motif file (matrix) | svg |
| 95 | A G C T C A T G G C A T G A T C T G C A C T A G G A T C A C G T | Tgif2(Homeobox)/mES-Tgif2-ChIP-Seq(GSE55404)/Homer | 1e-94 | -2.173e+02 | 0.0000 | 9543.0 | 37.75% | 7632.3 | 31.61% | motif file (matrix) | svg |
| 96 | C G T A G A C T C G A T A T C G G T A C G C A T C A T G C G T A T A C G G C A T G T A C C G T A C A T G A T G C G C T A C T A G G C A T G C A T G C A T G A C T | MafB(bZIP)/BMM-Mafb-ChIP-Seq(GSE75722)/Homer | 1e-88 | -2.043e+02 | 0.0000 | 1649.0 | 6.52% | 933.3 | 3.87% | motif file (matrix) | svg |
| 97 | C T A G C T A G C G T A C G T A T A C G C G A T C T A G C T G A C T G A C G T A T A C G G A C T | PU.1:IRF8(ETS:IRF)/pDC-Irf8-ChIP-Seq(GSE66899)/Homer | 1e-87 | -2.017e+02 | 0.0000 | 812.0 | 3.21% | 355.7 | 1.47% | motif file (matrix) | svg |
| 98 | A G T C C T G A A T C G A G C T A G C T G A C T A G T C G C T A A C G T C G A T G C A T C G A T A T C G C G T A T A G C G C A T A T G C C G T A | bZIP:IRF(bZIP,IRF)/Th17-BatF-ChIP-Seq(GSE39756)/Homer | 1e-75 | -1.748e+02 | 0.0000 | 1496.0 | 5.92% | 860.7 | 3.56% | motif file (matrix) | svg |
| 99 | C T G A T A C G G C A T A G C T A G C T A G T C T C G A A C T G C A G T A G C T A G C T G A T C | IRF3(IRF)/BMDM-Irf3-ChIP-Seq(GSE67343)/Homer | 1e-74 | -1.723e+02 | 0.0000 | 1174.0 | 4.64% | 629.6 | 2.61% | motif file (matrix) | svg |
| 100 | T C G A C T A G A G T C A G T C C G T A C G T A A C G T T A G C T C A G T A C G | NFY(CCAAT)/Promoter/Homer | 1e-73 | -1.699e+02 | 0.0000 | 2773.0 | 10.97% | 1866.3 | 7.73% | motif file (matrix) | svg |
| 101 | A T G C A G C T T C A G T G A C T C A G A T G C T G C A A C G T A T C G G A T C A C T G A G T C | NRF1(NRF)/MCF7-NRF1-ChIP-Seq(Unpublished)/Homer | 1e-68 | -1.575e+02 | 0.0000 | 1338.0 | 5.29% | 767.4 | 3.18% | motif file (matrix) | svg |
| 102 | T C A G T C A G T A G C A G T C C T G A A G T C C T A G A C G T A C T G A T C G | c-Myc(bHLH)/mES-cMyc-ChIP-Seq(GSE11431)/Homer | 1e-59 | -1.372e+02 | 0.0000 | 2075.0 | 8.21% | 1371.4 | 5.68% | motif file (matrix) | svg |
| 103 | T G A C C G A T A C T G A C T G A C T G G A C T A C T G A C G T A C T G A C T G G A T C G A T C | EKLF(Zf)/Erythrocyte-Klf1-ChIP-Seq(GSE20478)/Homer | 1e-59 | -1.364e+02 | 0.0000 | 1029.0 | 4.07% | 569.5 | 2.36% | motif file (matrix) | svg |
| 104 | T C A G C T G A C G T A C G T A T A C G G C A T C T A G C T G A C G T A C G T A T A C G G A C T | IRF1(IRF)/PBMC-IRF1-ChIP-Seq(GSE43036)/Homer | 1e-57 | -1.332e+02 | 0.0000 | 596.0 | 2.36% | 274.0 | 1.13% | motif file (matrix) | svg |
| 105 | C T G A A T C G A G C T A G C T A C G T T A G C C T G A T A C G C G A T A C G T G A C T A G T C | ISRE(IRF)/ThioMac-LPS-Expression(GSE23622)/Homer | 1e-52 | -1.203e+02 | 0.0000 | 323.0 | 1.28% | 115.8 | 0.48% | motif file (matrix) | svg |
| 106 | G C T A T A G C A G C T A T C G G T C A C G T A G C T A A T G C G A T C C T G A | IRF4(IRF)/GM12878-IRF4-ChIP-Seq(GSE32465)/Homer | 1e-51 | -1.191e+02 | 0.0000 | 1441.0 | 5.70% | 905.6 | 3.75% | motif file (matrix) | svg |
| 107 | A T C G A G C T A C T G A G T C A C T G A G T C C G T A A C G T A C T G A G T C A C T G A G T C | NRF(NRF)/Promoter/Homer | 1e-49 | -1.141e+02 | 0.0000 | 1432.0 | 5.66% | 907.5 | 3.76% | motif file (matrix) | svg |
| 108 | A G T C A C G T A C T G A G C T A C G T A C G T G T C A A G T C | Foxo1(Forkhead)/RAW-Foxo1-ChIP-Seq(Fan\_et\_al.)/Homer | 1e-48 | -1.124e+02 | 0.0000 | 5229.0 | 20.68% | 4128.1 | 17.10% | motif file (matrix) | svg |
| 109 | A C T G C A T G G C T A T C G A G C T A A G C T A G C T G T A C A G T C T G A C | NFkB-p65-Rel(RHD)/ThioMac-LPS-Expression(GSE23622)/Homer | 1e-47 | -1.097e+02 | 0.0000 | 273.0 | 1.08% | 93.1 | 0.39% | motif file (matrix) | svg |
| 110 | T A C G T A C G G T A C A T C G T A C G T A C G G T C A C T G A C G T A G A C T | E2F4(E2F)/K562-E2F4-ChIP-Seq(GSE31477)/Homer | 1e-43 | -9.915e+01 | 0.0000 | 2011.0 | 7.95% | 1403.8 | 5.81% | motif file (matrix) | svg |
| 111 | C G T A C A T G C A T G A C T G C T A G T C G A G C A T C G A T A G C T A G T C G A T C G T A C | NFkB-p65(RHD)/GM12787-p65-ChIP-Seq(GSE19485)/Homer | 1e-41 | -9.582e+01 | 0.0000 | 1373.0 | 5.43% | 895.5 | 3.71% | motif file (matrix) | svg |
| 112 | C G T A A C G T A C G T A C G T A C G T A G T C A G T C C T G A A G C T A G C T | NFAT(RHD)/Jurkat-NFATC1-ChIP-Seq(Jolma\_et\_al.)/Homer | 1e-41 | -9.574e+01 | 0.0000 | 2223.0 | 8.79% | 1587.9 | 6.58% | motif file (matrix) | svg |
| 113 | C A G T T C A G A G C T G A C T A C G T A G T C G A T C G A C T C T G A A C T G G A T C C G T A C T G A A G T C G T A C | Rfx6(HTH)/Min6b1-Rfx6.HA-ChIP-Seq(GSE62844)/Homer | 1e-38 | -8.846e+01 | 0.0000 | 3570.0 | 14.12% | 2759.0 | 11.43% | motif file (matrix) | svg |
| 114 | T A G C C G T A C T G A T A C G C G T A A C G T A C T G A C T G A G T C T A C G C T A G G T A C | YY1(Zf)/Promoter/Homer | 1e-38 | -8.797e+01 | 0.0000 | 535.0 | 2.12% | 276.2 | 1.14% | motif file (matrix) | svg |
| 115 | G A C T G C A T C T A G C G A T G A T C T C G A C A T G G A T C | Tgif1(Homeobox)/mES-Tgif1-ChIP-Seq(GSE55404)/Homer | 1e-37 | -8.673e+01 | 0.0000 | 8410.0 | 33.27% | 7127.2 | 29.52% | motif file (matrix) | svg |
| 116 | T G A C G C T A T G A C C G T A T C A G G A T C C G T A C A T G C A T G C T A G C T A G C T A G | Unknown-ESC-element(?)/mES-Nanog-ChIP-Seq(GSE11724)/Homer | 1e-37 | -8.544e+01 | 0.0000 | 1910.0 | 7.56% | 1354.1 | 5.61% | motif file (matrix) | svg |
| 117 | T C A G C A T G C A T G A C T G A C T G A G C T A C T G A C G T A C T G C A G T A T G C A G T C | KLF10(Zf)/HEK293-KLF10.GFP-ChIP-Seq(GSE58341)/Homer | 1e-35 | -8.284e+01 | 0.0000 | 3272.0 | 12.94% | 2520.2 | 10.44% | motif file (matrix) | svg |
| 118 | T G C A G C A T A G C T G C A T A G T C A G T C A G T C C T G A A C T G T C G A T C G A C A G T A T C G A G T C G A T C | ZNF143|STAF(Zf)/CUTLL-ZNF143-ChIP-Seq(GSE29600)/Homer | 1e-35 | -8.223e+01 | 0.0000 | 1348.0 | 5.33% | 903.1 | 3.74% | motif file (matrix) | svg |
| 119 | C T A G A G C T G A C T C A T G A G T C A G T C G T C A C A G T C T A G T C A G G T A C C T G A T C G A G A T C T G A C | Rfx2(HTH)/LoVo-RFX2-ChIP-Seq(GSE49402)/Homer | 1e-28 | -6.664e+01 | 0.0000 | 467.0 | 1.85% | 253.9 | 1.05% | motif file (matrix) | svg |
| 120 | T C A G T A C G T A G C A C G T A C T G C G A T A G T C C G T A T A C G A G T C | Meis1(Homeobox)/MastCells-Meis1-ChIP-Seq(GSE48085)/Homer | 1e-28 | -6.488e+01 | 0.0000 | 5255.0 | 20.79% | 4358.5 | 18.05% | motif file (matrix) | svg |
| 121 | C G T A C T A G C A T G A G C T A C T G C G A T A T C G C G T A G T C A G T C A | Tbet(T-box)/CD8-Tbet-ChIP-Seq(GSE33802)/Homer | 1e-27 | -6.418e+01 | 0.0000 | 2825.0 | 11.17% | 2200.1 | 9.11% | motif file (matrix) | svg |
| 122 | A G T C A T C G C T A G A G C T G A C T C T A G A G T C A G T C G C T A C A G T T C A G T C A G G A T C C T G A T C G A G A T C | RFX(HTH)/K562-RFX3-ChIP-Seq(SRA012198)/Homer | 1e-27 | -6.360e+01 | 0.0000 | 428.0 | 1.69% | 229.2 | 0.95% | motif file (matrix) | svg |
| 123 | T G C A A G C T C A T G C G T A A G C T A C T G G A T C G T C A C G T A A G C T | Atf4(bZIP)/MEF-Atf4-ChIP-Seq(GSE35681)/Homer | 1e-27 | -6.265e+01 | 0.0000 | 652.0 | 2.58% | 395.1 | 1.64% | motif file (matrix) | svg |
| 124 | T C G A G C A T A C G T C T A G G T A C T C G A G C A T T G A C T C G A A C G T | Chop(bZIP)/MEF-Chop-ChIP-Seq(GSE35681)/Homer | 1e-26 | -6.142e+01 | 0.0000 | 521.0 | 2.06% | 299.2 | 1.24% | motif file (matrix) | svg |
| 125 | T C A G C T A G C T A G C T A G T C A G T C G A C T G A C G A T A G T C G A T C A G T C T G A C | NFkB-p50,p52(RHD)/Monocyte-p50-ChIP-Chip(Schreiber\_et\_al.)/Homer | 1e-25 | -5.960e+01 | 0.0000 | 383.0 | 1.51% | 202.8 | 0.84% | motif file (matrix) | svg |
| 126 | C G T A A C T G G T C A A C G T A T C G C A G T C T A G T C A G C G T A A C T G C G T A A C G T C G T A C T G A T A C G | GATA3(Zf),DR4/iTreg-Gata3-ChIP-Seq(GSE20898)/Homer | 1e-25 | -5.940e+01 | 0.0000 | 254.0 | 1.00% | 115.2 | 0.48% | motif file (matrix) | svg |
| 127 | C T G A A T G C G C T A C G A T A T G C C G T A C G T A C G T A C T A G T A C G | Tcf3(HMG)/mES-Tcf3-ChIP-Seq(GSE11724)/Homer | 1e-25 | -5.813e+01 | 0.0000 | 806.0 | 3.19% | 522.2 | 2.16% | motif file (matrix) | svg |
| 128 | T C G A G A C T A G C T T G A C A G C T G T A C T C A G G A T C A T C G T G C A A C T G C T G A | GFX(?)/Promoter/Homer | 1e-24 | -5.582e+01 | 0.0000 | 179.0 | 0.71% | 71.6 | 0.30% | motif file (matrix) | svg |
| 129 | C T G A C T A G A T C G A G C T A C T G G A C T A G T C C T G A | Tbx5(T-box)/HL1-Tbx5.biotin-ChIP-Seq(GSE21529)/Homer | 1e-24 | -5.537e+01 | 0.0000 | 9283.0 | 36.72% | 8128.0 | 33.66% | motif file (matrix) | svg |
| 130 | C T A G C T A G A G T C T C A G A C T G A C G T A C G T C T G A | MYB(HTH)/ERMYB-Myb-ChIPSeq(GSE22095)/Homer | 1e-23 | -5.398e+01 | 0.0000 | 5132.0 | 20.30% | 4305.3 | 17.83% | motif file (matrix) | svg |
| 131 | T A G C G T A C A G T C G T A C C G A T A G T C A G T C A G T C A G T C A G T C C G T A G A T C | Zfp281(Zf)/ES-Zfp281-ChIP-Seq(GSE81042)/Homer | 1e-23 | -5.382e+01 | 0.0000 | 980.0 | 3.88% | 670.2 | 2.78% | motif file (matrix) | svg |
| 132 | C T G A A T G C G C T A G C A T A T G C C G T A T C G A C T G A C T A G T C A G T A C G G T C A | Tcf4(HMG)/Hct116-Tcf4-ChIP-Seq(SRA012054)/Homer | 1e-22 | -5.194e+01 | 0.0000 | 1340.0 | 5.30% | 971.8 | 4.02% | motif file (matrix) | svg |
| 133 | G A C T G T A C T G C A A C G T G A T C G C T A T C G A A C G T A G T C C G T A | Pdx1(Homeobox)/Islet-Pdx1-ChIP-Seq(SRA008281)/Homer | 1e-21 | -5.046e+01 | 0.0000 | 1946.0 | 7.70% | 1490.7 | 6.17% | motif file (matrix) | svg |
| 134 | A C G T C T G A G A T C A T C G G A C T T C A G G T A C T A G C | HIF-1a(bHLH)/MCF7-HIF1a-ChIP-Seq(GSE28352)/Homer | 1e-21 | -5.045e+01 | 0.0000 | 932.0 | 3.69% | 639.3 | 2.65% | motif file (matrix) | svg |
| 135 | C A T G C T A G A G C T G A C T C A T G A G T C G A T C G C T A C G A T C T A G T C A G G T A C C T G A T C G A | X-box(HTH)/NPC-H3K4me1-ChIP-Seq(GSE16256)/Homer | 1e-21 | -4.947e+01 | 0.0000 | 411.0 | 1.63% | 235.2 | 0.97% | motif file (matrix) | svg |
| 136 | C G T A A C T G C G T A A C G T A T C G C A G T T A G C C G T A T C G A G T A C C T G A T A G C C G T A A C T G C G T A A C G T C G T A C T G A A T C G G C T A | GATA3(Zf),DR8/iTreg-Gata3-ChIP-Seq(GSE20898)/Homer | 1e-21 | -4.854e+01 | 0.0000 | 276.0 | 1.09% | 140.2 | 0.58% | motif file (matrix) | svg |
| 137 | C T A G T C G A C G A T C T A G G C A T C A G T C T A G G A T C C G T A G T C A | CEBP:AP1(bZIP)/ThioMac-CEBPb-ChIP-Seq(GSE21512)/Homer | 1e-19 | -4.582e+01 | 0.0000 | 1660.0 | 6.57% | 1261.8 | 5.23% | motif file (matrix) | svg |
| 138 | A C G T C T A G C G T A A G T C G T A C A C G T A C G T A C G T G T C A G T A C T G A C G A C T | Nur77(NR)/K562-NR4A1-ChIP-Seq(GSE31363)/Homer | 1e-19 | -4.431e+01 | 0.0000 | 441.0 | 1.74% | 265.6 | 1.10% | motif file (matrix) | svg |
| 139 | A G T C G C A T C G T A C G T A G T A C A C G T A C T G G A T C G A T C T C G A | BMYB(HTH)/Hela-BMYB-ChIP-Seq(GSE27030)/Homer | 1e-18 | -4.280e+01 | 0.0000 | 4213.0 | 16.66% | 3536.1 | 14.65% | motif file (matrix) | svg |
| 140 | A C T G T C A G A G C T G A C T C A T G A G T C A G T C G C T A C G A T C T A G T C A G G T A C C T G A T C G A | Rfx1(HTH)/NPC-H3K4me1-ChIP-Seq(GSE16256)/Homer | 1e-18 | -4.246e+01 | 0.0000 | 677.0 | 2.68% | 452.4 | 1.87% | motif file (matrix) | svg |
| 141 | C A T G T A C G T A G C G A T C G A T C A T G C G T A C G A C T T C A G A T G C C G A T A T C G C A G T A C T G G T A C | Zic3(Zf)/mES-Zic3-ChIP-Seq(GSE37889)/Homer | 1e-17 | -4.084e+01 | 0.0000 | 1936.0 | 7.66% | 1518.7 | 6.29% | motif file (matrix) | svg |
| 142 | C G T A A T G C C G A T A C G T A G T C C G T A C G T A C G T A C T A G A T C G | TCFL2(HMG)/K562-TCF7L2-ChIP-Seq(GSE29196)/Homer | 1e-15 | -3.633e+01 | 0.0000 | 300.0 | 1.19% | 172.0 | 0.71% | motif file (matrix) | svg |
| 143 | C T A G C G T A G T C A C G T A A G T C G A T C A G C T C T A G C G T A A C G T G T C A G A T C | Six2(Homeobox)/NephronProgenitor-Six2-ChIP-Seq(GSE39837)/Homer | 1e-15 | -3.584e+01 | 0.0000 | 2409.0 | 9.53% | 1956.8 | 8.10% | motif file (matrix) | svg |
| 144 | T C G A A C T G A C T G C G T A C G T A T C G A A G T C C T G A A T C G G T A C G C A T C A T G | ETS:E-box(ETS,bHLH)/HPC7-Scl-ChIP-Seq(GSE22178)/Homer | 1e-15 | -3.547e+01 | 0.0000 | 324.0 | 1.28% | 191.1 | 0.79% | motif file (matrix) | svg |
| 145 | A C T G A C G T C A T G A T C G A T C G T G A C A C T G A T C G A T C G T G C A C T G A C G T A | E2F3(E2F)/MEF-E2F3-ChIP-Seq(GSE71376)/Homer | 1e-13 | -3.199e+01 | 0.0000 | 2314.0 | 9.15% | 1891.5 | 7.83% | motif file (matrix) | svg |
| 146 | T G A C C T A G A C T G T A G C C G A T A C T G A T G C C A T G A T C G A T C G A T C G T A G C C T G A T A G C G C T A A C T G C G T A A G C T C G T A C T G A | GATA:SCL(Zf,bHLH)/Ter119-SCL-ChIP-Seq(GSE18720)/Homer | 1e-13 | -3.129e+01 | 0.0000 | 306.0 | 1.21% | 184.8 | 0.77% | motif file (matrix) | svg |
| 147 | C T G A C A G T C T G A A G T C C T A G G A C T A T C G G T A C | HIF-1b(HLH)/T47D-HIF1b-ChIP-Seq(GSE59937)/Homer | 1e-13 | -3.085e+01 | 0.0000 | 3976.0 | 15.73% | 3396.3 | 14.07% | motif file (matrix) | svg |
| 148 | G A C T C A G T A G C T C G A T A G T C G A T C A G T C C G T A A T G C T C A G | Rbpj1(?)/Panc1-Rbpj1-ChIP-Seq(GSE47459)/Homer | 1e-13 | -3.023e+01 | 0.0000 | 3889.0 | 15.38% | 3321.8 | 13.76% | motif file (matrix) | svg |
| 149 | G A C T C T A G C T A G A G T C T G C A A C T G A C G T A C G T C T A G T C A G | AMYB(HTH)/Testes-AMYB-ChIP-Seq(GSE44588)/Homer | 1e-12 | -2.958e+01 | 0.0000 | 4252.0 | 16.82% | 3657.8 | 15.15% | motif file (matrix) | svg |
| 150 | T A C G T C A G T G C A A G C T T G A C A G C T A G T C A C T G G A T C A C T G T C G A A C T G C T G A C T G A A T G C | ZBTB33(Zf)/GM12878-ZBTB33-ChIP-Seq(GSE32465)/Homer | 1e-12 | -2.809e+01 | 0.0000 | 278.0 | 1.10% | 168.3 | 0.70% | motif file (matrix) | svg |
| 151 | T A G C C G A T A C G T A G C T A G C T A G T C A T G C A G T C A C T G A T G C A T G C G C T A | E2F7(E2F)/Hela-E2F7-ChIP-Seq(GSE32673)/Homer | 1e-11 | -2.726e+01 | 0.0000 | 501.0 | 1.98% | 345.9 | 1.43% | motif file (matrix) | svg |
| 152 | C T A G T A C G G A T C G T A C G C T A A G C T A G C T G T C A T C G A T A G C | Nanog(Homeobox)/mES-Nanog-ChIP-Seq(GSE11724)/Homer | 1e-10 | -2.308e+01 | 0.0000 | 9715.0 | 38.43% | 8810.6 | 36.49% | motif file (matrix) | svg |
| 153 | C T A G C A T G T G C A A C G T A G T C C G T A C A T G T C A G A C G T A C G T G C T A A G T C | Six1(Homeobox)/Myoblast-Six1-ChIP-Chip(GSE20150)/Homer | 1e-9 | -2.286e+01 | 0.0000 | 657.0 | 2.60% | 486.5 | 2.01% | motif file (matrix) | svg |
| 154 | T C A G T G A C G T A C C G T A A C G T T G A C A C G T T C A G A G C T G A C T | NeuroD1(bHLH)/Islet-NeuroD1-ChIP-Seq(GSE30298)/Homer | 1e-9 | -2.140e+01 | 0.0000 | 2171.0 | 8.59% | 1823.5 | 7.55% | motif file (matrix) | svg |
| 155 | T G C A A T G C A C G T A C G T A C G T A T G C C T A G A C G T A C G T A G C T G A T C A G C T | T1ISRE(IRF)/ThioMac-Ifnb-Expression/Homer | 1e-9 | -2.108e+01 | 0.0000 | 58.0 | 0.23% | 22.6 | 0.09% | motif file (matrix) | svg |
| 156 | C G T A T A G C A G T C C T A G C A G T C T A G C T G A G T A C G C A T T C G A C G T A G C A T A G C T C T A G T C G A | PAX3:FKHR-fusion(Paired,Homeobox)/Rh4-PAX3:FKHR-ChIP-Seq(GSE19063)/Homer | 1e-8 | -1.964e+01 | 0.0000 | 449.0 | 1.78% | 322.1 | 1.33% | motif file (matrix) | svg |
| 157 | A C T G G A C T A G T C C T G A G A T C T C A G A T G C G A C T A G T C A T G C T A G C A G C T A T C G T G C A | PAX5(Paired,Homeobox),condensed/GM12878-PAX5-ChIP-Seq(GSE32465)/Homer | 1e-8 | -1.853e+01 | 0.0000 | 381.0 | 1.51% | 269.4 | 1.12% | motif file (matrix) | svg |
| 158 | T C A G G A C T C A G T C T G A A G C T C T A G G A C T T G C A C T G A A G T C | HLF(bZIP)/HSC-HLF.Flag-ChIP-Seq(GSE69817)/Homer | 1e-7 | -1.746e+01 | 0.0000 | 1455.0 | 5.76% | 1205.4 | 4.99% | motif file (matrix) | svg |
| 159 | G T A C C G T A C G T A T A C G G C A T G T A C C G T A C A T G A G T C C G T A C G T A C G A T G C A T G C A T G A C T | MafF(bZIP)/HepG2-MafF-ChIP-Seq(GSE31477)/Homer | 1e-7 | -1.680e+01 | 0.0000 | 527.0 | 2.08% | 396.5 | 1.64% | motif file (matrix) | svg |
| 160 | T A C G T A C G G T A C A T C G A C T G T A C G T C G A C T G A T C G A A T C G | E2F6(E2F)/Hela-E2F6-ChIP-Seq(GSE31477)/Homer | 1e-6 | -1.579e+01 | 0.0000 | 1854.0 | 7.33% | 1574.4 | 6.52% | motif file (matrix) | svg |
| 161 | C T G A T C A G G T A C G C T A A C T G T G A C G C A T C A T G | SCL(bHLH)/HPC7-Scl-ChIP-Seq(GSE13511)/Homer | 1e-6 | -1.551e+01 | 0.0000 | 12543.0 | 49.61% | 11592.3 | 48.01% | motif file (matrix) | svg |
| 162 | T G C A A G C T A C G T C T A G G A T C C T A G G A T C G T C A C T G A A G T C | CEBP(bZIP)/ThioMac-CEBPb-ChIP-Seq(GSE21512)/Homer | 1e-6 | -1.463e+01 | 0.0000 | 1199.0 | 4.74% | 993.4 | 4.11% | motif file (matrix) | svg |
| 163 | T A C G A T G C G A C T A C T G A G C T A G T C G T C A T G C A A C G T A G T C G C T A T G C A | Pknox1(Homeobox)/ES-Prep1-ChIP-Seq(GSE63282)/Homer | 1e-5 | -1.349e+01 | 0.0000 | 764.0 | 3.02% | 614.0 | 2.54% | motif file (matrix) | svg |
| 164 | C A T G A G T C A G C T C G T A C G A T C G A T G C A T G C A T C G A T C T G A C A T G T G A C | Mef2d(MADS)/Retina-Mef2d-ChIP-Seq(GSE61391)/Homer | 1e-5 | -1.290e+01 | 0.0000 | 303.0 | 1.20% | 220.7 | 0.91% | motif file (matrix) | svg |
| 165 | C T A G A C G T A G T C C G T A A C T G A G T C G C A T A C T G G C A T A G T C G A C T G A T C G C A T A G T C A G C T | ZNF317(Zf)/HEK293-ZNF317.GFP-ChIP-Seq(GSE58341)/Homer | 1e-5 | -1.230e+01 | 0.0000 | 249.0 | 0.98% | 177.3 | 0.73% | motif file (matrix) | svg |
| 166 | G T A C C T A G T C A G A G C T T A G C C G T A A T G C T A C G A G T C G T A C G T C A A G T C | Srebp2(bHLH)/HepG2-Srebp2-ChIP-Seq(GSE31477)/Homer | 1e-5 | -1.210e+01 | 0.0000 | 382.0 | 1.51% | 289.6 | 1.20% | motif file (matrix) | svg |
| 167 | C G T A A C G T A G C T C G A T C T A G G T A C C G T A A G C T C G T A G C T A | Oct4(POU,Homeobox)/mES-Oct4-ChIP-Seq(GSE11431)/Homer | 1e-5 | -1.210e+01 | 0.0000 | 771.0 | 3.05% | 627.2 | 2.60% | motif file (matrix) | svg |
| 168 | G A C T C T A G C T A G C T A G A C T G T C G A C T G A C T A G C T A G C T A G G T A C G T C A | ZNF467(Zf)/HEK293-ZNF467.GFP-ChIP-Seq(GSE58341)/Homer | 1e-5 | -1.197e+01 | 0.0000 | 2564.0 | 10.14% | 2253.6 | 9.33% | motif file (matrix) | svg |
| 169 | T A G C T A G C G A C T C T A G A G C T A G T C G T C A T G C A A C G T A T G C G C T A T G C A | Pbx3(Homeobox)/GM12878-PBX3-ChIP-Seq(GSE32465)/Homer | 1e-5 | -1.170e+01 | 0.0000 | 763.0 | 3.02% | 622.2 | 2.58% | motif file (matrix) | svg |
| 170 | A G T C G A C T C A G T A C T G C T A G T G A C G C T A A T G C G C A T A T C G C G A T A C T G G A T C G T A C G T C A C T G A | NF1(CTF)/LNCAP-NF1-ChIP-Seq(Unpublished)/Homer | 1e-5 | -1.155e+01 | 0.0000 | 949.0 | 3.75% | 788.6 | 3.27% | motif file (matrix) | svg |
| 171 | T G C A C G T A G T C A A G C T A G T C G C T A T A G C C G A T C T A G G A T C | Gfi1b(Zf)/HPC7-Gfi1b-ChIP-Seq(GSE22178)/Homer | 1e-4 | -1.126e+01 | 0.0000 | 1502.0 | 5.94% | 1288.6 | 5.34% | motif file (matrix) | svg |
| 172 | C G A T C T A G T C G A A G C T C G A T C T G A C G T A A G C T A C T G C T A G A T G C G A T C | Hoxb4(Homeobox)/ES-Hoxb4-ChIP-Seq(GSE34014)/Homer | 1e-4 | -1.043e+01 | 0.0001 | 411.0 | 1.63% | 321.0 | 1.33% | motif file (matrix) | svg |
| 173 | T C G A A G T C C G T A A T C G A T G C C G A T A C T G A G T C A G C T A C T G | Tcf12(bHLH)/GM12878-Tcf12-ChIP-Seq(GSE32465)/Homer | 1e-4 | -1.013e+01 | 0.0001 | 2726.0 | 10.78% | 2421.3 | 10.03% | motif file (matrix) | svg |
| 174 | A T G C A G T C G T A C A G C T T C G A C T A G G A T C C T G A G T C A A G T C G C T A T C A G | Rfx5(HTH)/GM12878-Rfx5-ChIP-Seq(GSE31477)/Homer | 1e-4 | -9.817e+00 | 0.0001 | 1086.0 | 4.30% | 922.1 | 3.82% | motif file (matrix) | svg |
| 175 | G T A C G A C T C G T A C T G A T C G A C G T A G C T A C A G T C T G A T A C G | Mef2a(MADS)/HL1-Mef2a.biotin-ChIP-Seq(GSE21529)/Homer | 1e-4 | -9.616e+00 | 0.0001 | 650.0 | 2.57% | 533.2 | 2.21% | motif file (matrix) | svg |
| 176 | A G T C C G A T A C T G A T C G T G A C G C T A C A T G A T C G T G A C C G A T A C T G T A G C G T A C G T C A | Tlx?(NR)/NPC-H3K4me1-ChIP-Seq(GSE16256)/Homer | 1e-4 | -9.410e+00 | 0.0002 | 1064.0 | 4.21% | 905.6 | 3.75% | motif file (matrix) | svg |
| 177 | C T G A C G A T C T A G T C A G G A T C C T G A T C A G G A T C C T G A A C T G A G T C G C T A A C G T A G T C G C A T | PRDM9(Zf)/Testis-DMC1-ChIP-Seq(GSE35498)/Homer | 1e-4 | -9.403e+00 | 0.0002 | 1106.0 | 4.37% | 943.4 | 3.91% | motif file (matrix) | svg |
| 178 | T C A G A T C G G A C T A C T G G A C T C A G T C T A G C G T A G T A C C G T A C T A G A T C G | Tbx20(T-box)/Heart-Tbx20-ChIP-Seq(GSE29636)/Homer | 1e-4 | -9.365e+00 | 0.0002 | 618.0 | 2.44% | 506.7 | 2.10% | motif file (matrix) | svg |
| 179 | T G A C G T A C C G T A A C T G T G A C C G A T A C T G A T C G A G C T T A C G T C G A T A G C G T A C C G T A A T C G T G A C G C A T A C T G A C T G A T G C | Twist(bHLH)/HMLE-TWIST1-ChIP-Seq(Chang\_et\_al)/Homer | 1e-3 | -9.040e+00 | 0.0002 | 349.0 | 1.38% | 272.4 | 1.13% | motif file (matrix) | svg |
| 180 | T A G C C G A T T A C G A C T G A G T C A C T G A T C G A T C G C G T A C T G A | E2F1(E2F)/Hela-E2F1-ChIP-Seq(GSE22478)/Homer | 1e-3 | -8.973e+00 | 0.0003 | 872.0 | 3.45% | 735.5 | 3.05% | motif file (matrix) | svg |
| 181 | A G C T G C A T A C T G A C G T A G T C A C G T C T A G T A C G | Smad3(MAD)/NPC-Smad3-ChIP-Seq(GSE36673)/Homer | 1e-3 | -8.773e+00 | 0.0003 | 7913.0 | 31.30% | 7304.7 | 30.25% | motif file (matrix) | svg |
| 182 | A T C G T G A C A T G C C T G A T C A G G A C T A G T C C G A T T C A G T C G A C A T G C T A G C T A G C G T A C T A G C T A G C T G A C T A G C T A G A T G C | ZSCAN22(Zf)/HEK293-ZSCAN22.GFP-ChIP-Seq(GSE58341)/Homer | 1e-3 | -8.555e+00 | 0.0004 | 199.0 | 0.79% | 146.0 | 0.60% | motif file (matrix) | svg |
| 183 | G T C A A C G T C T A G G T C A C G T A G C A T C G T A C G T A G C A T C A G T A G T C C G T A C A G T C T A G C T G A | OCT:OCT(POU,Homeobox,IR1)/NPC-Brn2-ChIP-Seq(GSE35496)/Homer | 1e-3 | -8.383e+00 | 0.0005 | 19.0 | 0.08% | 7.2 | 0.03% | motif file (matrix) | svg |
| 184 | T C G A T G A C G C A T A G C T C A G T G A T C G C T A G A T C G A C T A C G T G C A T A G T C | PRDM1(Zf)/Hela-PRDM1-ChIP-Seq(GSE31477)/Homer | 1e-3 | -8.094e+00 | 0.0006 | 1353.0 | 5.35% | 1178.7 | 4.88% | motif file (matrix) | svg |
| 185 | T G C A G C A T C G A T C G T A C A G T A C T G G T A C C G T A C T G A A G C T G T C A A C T G C T A G G T C A C G A T A C T G G T A C T G C A C G T A A G C T | CEBP:CEBP(bZIP)/MEF-Chop-ChIP-Seq(GSE35681)/Homer | 1e-3 | -7.899e+00 | 0.0007 | 245.0 | 0.97% | 187.6 | 0.78% | motif file (matrix) | svg |
| 186 | T A C G T A G C G C T A C G A T C T A G A C G T C A G T C A G T G C T A A G T C G T C A G C A T | FOXK2(Forkhead)/U2OS-FOXK2-ChIP-Seq(E-MTAB-2204)/Homer | 1e-3 | -7.881e+00 | 0.0007 | 1305.0 | 5.16% | 1136.2 | 4.71% | motif file (matrix) | svg |
| 187 | A G C T A G C T T A G C A T C G A G T C A C T G A T G C A T C G T C G A C T G A T C G A C T G A | E2F(E2F)/Hela-CellCycle-Expression/Homer | 1e-3 | -7.612e+00 | 0.0010 | 218.0 | 0.86% | 165.7 | 0.69% | motif file (matrix) | svg |
| 188 | G T C A G C A T G C T A C A G T C T A G G A T C C G T A C T G A C G T A C G A T | Oct2(POU,Homeobox)/Bcell-Oct2-ChIP-Seq(GSE21512)/Homer | 1e-3 | -7.433e+00 | 0.0011 | 446.0 | 1.76% | 364.9 | 1.51% | motif file (matrix) | svg |
| 189 | C T A G A T C G G T C A C A T G A G T C G A C T T A C G C A G T A G T C A G T C C T G A C G A T C T A G A T C G G A C T A T C G A G T C G A C T C T A G T C G A | REST-NRSF(Zf)/Jurkat-NRSF-ChIP-Seq/Homer | 1e-3 | -7.407e+00 | 0.0012 | 29.0 | 0.11% | 14.8 | 0.06% | motif file (matrix) | svg |
| 190 | A T G C A G T C G C A T A G C T A C G T T C A G C G A T A G C T G A T C A T C G | Sox10(HMG)/SciaticNerve-Sox3-ChIP-Seq(GSE35132)/Homer | 1e-3 | -7.052e+00 | 0.0017 | 3734.0 | 14.77% | 3399.0 | 14.08% | motif file (matrix) | svg |
| 191 | G A C T C G T A A C G T A C T G A G T C C G T A C T G A C G T A C A G T A C G T G T C A T C A G | Brn1(POU,Homeobox)/NPC-Brn1-ChIP-Seq(GSE35496)/Homer | 1e-2 | -6.416e+00 | 0.0031 | 506.0 | 2.00% | 423.4 | 1.75% | motif file (matrix) | svg |
| 192 | T G C A C T A G C T A G C T G A C A T G A C T G T G C A G A T C G T C A T G C A G T C A G T C A A G C T C T A G G C A T | ZNF675(Zf)/HEK293-ZNF675.GFP-ChIP-Seq(GSE58341)/Homer | 1e-2 | -6.386e+00 | 0.0032 | 258.0 | 1.02% | 204.6 | 0.85% | motif file (matrix) | svg |
| 193 | C G A T C T A G A C G T G T C A C G T A C G T A A G T C C G T A | Foxo3(Forkhead)/U2OS-Foxo3-ChIP-Seq(E-MTAB-2701)/Homer | 1e-2 | -5.800e+00 | 0.0057 | 1419.0 | 5.61% | 1261.8 | 5.23% | motif file (matrix) | svg |
| 194 | C G T A A C G T A C T G G T A C C G T A A C G T C G T A C G T A A C G T A C T G A G T C C G T A A C G T C T G A G C A T | OCT:OCT-short(POU,Homeobox)/NPC-OCT6-ChIP-Seq(GSE43916)/Homer | 1e-2 | -5.761e+00 | 0.0059 | 988.0 | 3.91% | 865.8 | 3.59% | motif file (matrix) | svg |
| 195 | C T G A C T A G T G C A C T G A T C G A A G C T C A T G T C G A A G T C G A C T A C G T A G T C G A T C G A T C G A C T | ZNF528(Zf)/HEK293-ZNF528.GFP-ChIP-Seq(GSE58341)/Homer | 1e-2 | -5.594e+00 | 0.0069 | 16.0 | 0.06% | 7.8 | 0.03% | motif file (matrix) | svg |
| 196 | G C A T A T C G C A T G G T A C G C T A A G T C T C A G T G A C G T C A T G C A | Arnt:Ahr(bHLH)/MCF7-Arnt-ChIP-Seq(Lo\_et\_al.)/Homer | 1e-2 | -5.507e+00 | 0.0075 | 2101.0 | 8.31% | 1897.4 | 7.86% | motif file (matrix) | svg |
| 197 | T C G A G C A T A T G C C T G A A T G C T A G C A G T C G T A C T C G A A G C T | Srebp1a(bHLH)/HepG2-Srebp1a-ChIP-Seq(GSE31477)/Homer | 1e-2 | -5.295e+00 | 0.0093 | 518.0 | 2.05% | 442.0 | 1.83% | motif file (matrix) | svg |
| 198 | A T G C G A C T A C G T C T A G A C G T A C G T A C G T C T G A G A T C G C T A A G C T C G T A | Foxa2(Forkhead)/Liver-Foxa2-ChIP-Seq(GSE25694)/Homer | 1e-2 | -5.153e+00 | 0.0106 | 1587.0 | 6.28% | 1424.9 | 5.90% | motif file (matrix) | svg |
| 199 | C T A G A T G C A T G C C G A T A C T G G A C T A T G C G C T A T G A C A G C T T A G C G C T A | PBX1(Homeobox)/MCF7-PBX1-ChIP-Seq(GSE28007)/Homer | 1e-2 | -5.128e+00 | 0.0108 | 298.0 | 1.18% | 245.3 | 1.02% | motif file (matrix) | svg |
| 200 | C A T G A G T C C T G A T G A C A T C G G A C T G T C A A G T C T A G C G A T C | HIF2a(bHLH)/785\_O-HIF2a-ChIP-Seq(GSE34871)/Homer | 1e-2 | -4.680e+00 | 0.0169 | 1085.0 | 4.29% | 965.8 | 4.00% | motif file (matrix) | svg |
