# Supplementary material for: Genome-wide CRISPR Screens in T Helper Cells Reveal Pervasive Crosstalk between Activation and Differentiation
Source: Cell. 2019 Feb 7;176(4):882–896.e18. doi: 10.1016/j.cell.2018.11.044 (PMC6370901; doi:10.1016/j.cell.2018.11.044)
Supplement: Data S2. Processed Data from All the Steps of the Analysis, Related to Figure 1 [file mmc2.zip › supplemental data/motif analysis/Pparg_homer/homerResults.html]

Pparg\_motifs/ - Homer de novo Motif Results


# Homer *de novo* Motif Results (Pparg\_motifs/)

Known Motif Enrichment Results  
Gene Ontology Enrichment Results  
If Homer is having trouble matching a motif to a known motif, try copy/pasting the matrix file into
STAMP  
More information on motif finding results: HOMER
| Description of Results
| Tips
  
Total target sequences = 12520  
Total background sequences = 35819  
\* - possible false positive  

|  |  |  |  |  |  |  |  |  |
| --- | --- | --- | --- | --- | --- | --- | --- | --- |
| Rank | Motif | P-value | log P-pvalue | % of Targets | % of Background | STD(Bg STD) | Best Match/Details | Motif File |
| 1 | C A G T T C G A A C G T A C T G C G T A A T C G C G A T T G A C C G T A A G C T | 1e-1923 | -4.430e+03 | 40.44% | 9.05% | 51.7bp (68.5bp) | Fra1(bZIP)/BT549-Fra1-ChIP-Seq(GSE46166)/Homer(0.990) More Information | Similar Motifs Found | motif file (matrix) |
| 2 | G A C T T A C G T G C A G T A C G A T C G A C T G A C T G A C T T A C G G T C A G A T C G A T C | 1e-1031 | -2.375e+03 | 25.32% | 6.10% | 48.1bp (62.0bp) | Pparg::Rxra/MA0065.2/Jaspar(0.941) More Information | Similar Motifs Found | motif file (matrix) |
| 3 | G T A C C T G A A G T C C G A T C A G T A T G C T A G C A C G T A T C G G A C T | 1e-606 | -1.397e+03 | 20.71% | 6.42% | 52.1bp (62.6bp) | ETS1(ETS)/Jurkat-ETS1-ChIP-Seq(GSE17954)/Homer(0.973) More Information | Similar Motifs Found | motif file (matrix) |
| 4 | A T C G G A C T T A G C T C G A A G C T G C A T G C A T G A T C | 1e-451 | -1.039e+03 | 29.53% | 13.80% | 53.4bp (66.5bp) | MAFG::NFE2L1/MA0089.1/Jaspar(0.830) More Information | Similar Motifs Found | motif file (matrix) |
| 5 | T A C G C A T G A T C G G C A T C T A G T C G A T G A C C T A G | 1e-263 | -6.061e+02 | 51.69% | 36.45% | 55.5bp (68.2bp) | MEIS1/MA0498.2/Jaspar(0.755) More Information | Similar Motifs Found | motif file (matrix) |
| 6 | A C T G G A T C G A C T A C T G A G C T C T A G A C T G A G C T C G A T C G A T | 1e-247 | -5.696e+02 | 14.64% | 6.21% | 52.5bp (64.4bp) | RUNX1(Runt)/Jurkat-RUNX1-ChIP-Seq(GSE29180)/Homer(0.974) More Information | Similar Motifs Found | motif file (matrix) |
| 7 | A C G T G A C T A G C T T G A C G C T A A C T G C G A T G C T A A C G T A T C G C G T A T A G C | 1e-150 | -3.455e+02 | 7.04% | 2.57% | 54.1bp (65.8bp) | IRF:BATF(IRF:bZIP)/pDC-Irf8-ChIP-Seq(GSE66899)/Homer(0.932) More Information | Similar Motifs Found | motif file (matrix) |
| 8 | A C G T A T G C G C A T C G A T G C T A A G C T A T G C G C A T A T C G T G C A | 1e-101 | -2.327e+02 | 13.42% | 7.81% | 54.6bp (60.1bp) | GATA3(Zf)/iTreg-Gata3-ChIP-Seq(GSE20898)/Homer(0.963) More Information | Similar Motifs Found | motif file (matrix) |
| 9 | A T C G G C A T T G A C C G T A C G T A G C T A C T G A A G T C C G T A C T A G C T G A C T G A | 1e-91 | -2.101e+02 | 9.68% | 5.20% | 54.5bp (64.6bp) | PB0120.1\_Foxj1\_2/Jaspar(0.754) More Information | Similar Motifs Found | motif file (matrix) |
| 10 | C G A T G C A T G T A C G A C T A T C G G C T A T C A G C G T A G C T A T G C A | 1e-87 | -2.003e+02 | 10.08% | 5.58% | 55.3bp (63.3bp) | STAT5(Stat)/mCD4+-Stat5-ChIP-Seq(GSE12346)/Homer(0.908) More Information | Similar Motifs Found | motif file (matrix) |
| 11 | A C G T A G C T G T C A G A C T A C T G T G C A T A G C C G A T T A C G G C A T C T G A G T A C | 1e-75 | -1.734e+02 | 2.02% | 0.49% | 51.9bp (70.3bp) | PB0178.1\_Sox8\_2/Jaspar(0.673) More Information | Similar Motifs Found | motif file (matrix) |
| 12 | C A T G A C T G C T A G T C G A T G C A C G T A A C G T A G T C G T A C G A T C A G T C G C T A | 1e-60 | -1.403e+02 | 3.26% | 1.28% | 55.0bp (57.0bp) | NFkB-p65(RHD)/GM12787-p65-ChIP-Seq(GSE19485)/Homer(0.969) More Information | Similar Motifs Found | motif file (matrix) |
| 13 | C T G A A G T C A C G T A C G T C T G A A G T C G T A C A C T G A C T G C G T A | 1e-46 | -1.076e+02 | 0.50% | 0.04% | 55.2bp (53.9bp) | SD0001.1\_at\_AC\_acceptor/Jaspar(0.733) More Information | Similar Motifs Found | motif file (matrix) |
| 14 | C T A G C T A G A C G T C G A T C G T A A G T C C G A T A C G T C T G A A G T C C G T A A C T G | 1e-44 | -1.032e+02 | 0.39% | 0.02% | 50.4bp (27.9bp) | FOXA1(Forkhead)/MCF7-FOXA1-ChIP-Seq(GSE26831)/Homer(0.582) More Information | Similar Motifs Found | motif file (matrix) |
| 15 | A G T C G T A C A G T C G A T C A G T C G T A C G T A C G T A C G T A C G T A C | 1e-40 | -9.356e+01 | 13.27% | 9.55% | 58.1bp (55.5bp) | PB0097.1\_Zfp281\_1/Jaspar(0.905) More Information | Similar Motifs Found | motif file (matrix) |
| 16 | A G T C A C G T A C G T A T C G A T C G A C G T C A T G C G T A C G T A A G T C A G T C C G T A | 1e-29 | -6.761e+01 | 0.22% | 0.01% | 40.0bp (20.6bp) | PB0081.1\_Tcf1\_1/Jaspar(0.653) More Information | Similar Motifs Found | motif file (matrix) |
| 17 | C G A T A C T G A C T G A T C G A T C G A G T C A G T C G T A C | 1e-25 | -5.904e+01 | 3.48% | 2.02% | 55.5bp (58.5bp) | ZNF692(Zf)/HEK293-ZNF692.GFP-ChIP-Seq(GSE58341)/Homer(0.884) More Information | Similar Motifs Found | motif file (matrix) |
| 18 \* | C G A T A C T G C T G A T A C G A C G T A C T G C T G A C A T G C G A T A C T G C T G A T C A G | 1e-10 | -2.466e+01 | 0.61% | 0.26% | 50.2bp (69.4bp) | Egr1(Zf)/K562-Egr1-ChIP-Seq(GSE32465)/Homer(0.681) More Information | Similar Motifs Found | motif file (matrix) |
| 19 \* | A C T G A G T C A C G T A G T C A C G T A C T G C G T A A G T C A C G T C G T A | 1e-9 | -2.252e+01 | 0.18% | 0.03% | 50.4bp (28.8bp) | ZNF519(Zf)/HEK293-ZNF519.GFP-ChIP-Seq(GSE58341)/Homer(0.650) More Information | Similar Motifs Found | motif file (matrix) |
| 20 \* | A C T G A C G T C G T A A C G T C G T A A C G T C G T A C G T A A G T C A C G T | 1e-5 | -1.319e+01 | 0.10% | 0.02% | 59.9bp (58.0bp) | PB0080.1\_Tbp\_1/Jaspar(0.667) More Information | Similar Motifs Found | motif file (matrix) |
| 21 \* | A T G C G T A C G A T C A G C T A G T C A G T C C G A T T A G C T G A C G A T C G A T C T G A C | 1e-5 | -1.203e+01 | 0.80% | 0.50% | 58.9bp (48.4bp) | ZNF263/MA0528.1/Jaspar(0.680) More Information | Similar Motifs Found | motif file (matrix) |
