# Supplementary material for: Genome-wide CRISPR Screens in T Helper Cells Reveal Pervasive Crosstalk between Activation and Differentiation
Source: Cell. 2019 Feb 7;176(4):882–896.e18. doi: 10.1016/j.cell.2018.11.044 (PMC6370901; doi:10.1016/j.cell.2018.11.044)
Supplement: Data S2. Processed Data from All the Steps of the Analysis, Related to Figure 1 [file mmc2.zip › supplemental data/motif analysis/Pparg_homer/knownResults.html]

Pparg\_motifs - Homer Known Motif Enrichment Results


# Homer Known Motif Enrichment Results (Pparg\_motifs)

Homer *de novo* Motif Results  
Gene Ontology Enrichment Results  
Known Motif Enrichment Results (txt file)  
Total Target Sequences = 12519, Total Background Sequences = 35839

|  |  |  |  |  |  |  |  |  |  |  |  |
| --- | --- | --- | --- | --- | --- | --- | --- | --- | --- | --- | --- |
| Rank | Motif | Name | P-value | log P-pvalue | q-value (Benjamini) | # Target Sequences with Motif | % of Targets Sequences with Motif | # Background Sequences with Motif | % of Background Sequences with Motif | Motif File | SVG |
| 1 | C A G T T G C A A C G T A C T G C G T A A T C G C G A T T G A C C G T A A C G T | BATF(bZIP)/Th17-BATF-ChIP-Seq(GSE39756)/Homer | 1e-1774 | -4.087e+03 | 0.0000 | 4359.0 | 34.82% | 2515.9 | 7.02% | motif file (matrix) | svg |
| 2 | C T A G T C G A A C G T A C T G C G T A A T G C A C G T G T A C C G T A A G C T G A T C G T A C | Atf3(bZIP)/GBM-ATF3-ChIP-Seq(GSE33912)/Homer | 1e-1723 | -3.968e+03 | 0.0000 | 4277.0 | 34.16% | 2487.4 | 6.94% | motif file (matrix) | svg |
| 3 | C T A G T C G A G C A T C A T G G C T A T A G C C G A T G T A C C T G A A G C T | JunB(bZIP)/DendriticCells-Junb-ChIP-Seq(GSE36099)/Homer | 1e-1638 | -3.772e+03 | 0.0000 | 3842.0 | 30.69% | 2061.0 | 5.75% | motif file (matrix) | svg |
| 4 | A C T G C T A G T C G A C G A T C A T G G C T A A T C G C G A T G T A C G C T A A G C T G T A C | Fra1(bZIP)/BT549-Fra1-ChIP-Seq(GSE46166)/Homer | 1e-1618 | -3.727e+03 | 0.0000 | 3786.0 | 30.24% | 2019.9 | 5.64% | motif file (matrix) | svg |
| 5 | T C G A A C G T C A T G G C T A T A G C C G A T G T A C G C T A A C G T A T G C | AP-1(bZIP)/ThioMac-PU.1-ChIP-Seq(GSE21512)/Homer | 1e-1608 | -3.704e+03 | 0.0000 | 4406.0 | 35.19% | 2855.3 | 7.97% | motif file (matrix) | svg |
| 6 | C A T G C T A G T C G A A C G T A C T G C G T A T A G C C G A T T G A C C G T A A G C T G A T C | Fra2(bZIP)/Striatum-Fra2-ChIP-Seq(GSE43429)/Homer | 1e-1449 | -3.338e+03 | 0.0000 | 3378.0 | 26.98% | 1764.6 | 4.93% | motif file (matrix) | svg |
| 7 | C T A G T C G A C G A T A C T G C G T A T A C G A G C T T G A C G C T A A C G T G A T C T A G C | Fosl2(bZIP)/3T3L1-Fosl2-ChIP-Seq(GSE56872)/Homer | 1e-1184 | -2.726e+03 | 0.0000 | 2605.0 | 20.81% | 1233.9 | 3.44% | motif file (matrix) | svg |
| 8 | C G A T T A C G T G C A G T A C G A T C G A C T A G C T A C G T A T C G G T A C G A T C G T A C G A T C G T C A | PPARE(NR),DR1/3T3L1-Pparg-ChIP-Seq(GSE13511)/Homer | 1e-1026 | -2.364e+03 | 0.0000 | 3791.0 | 30.28% | 3133.8 | 8.75% | motif file (matrix) | svg |
| 9 | C T A G T C G A A C G T A C T G C G T A T A G C C G A T G T A C C G T A A G C T G A T C G T A C | Jun-AP1(bZIP)/K562-cJun-ChIP-Seq(GSE31477)/Homer | 1e-864 | -1.990e+03 | 0.0000 | 1933.0 | 15.44% | 910.5 | 2.54% | motif file (matrix) | svg |
| 10 | A G C T C T G A C T A G C T A G A C T G T A G C T G C A T C G A C T G A C T A G C A T G A C G T A T G C T C G A | RXR(NR),DR1/3T3L1-RXR-ChIP-Seq(GSE13511)/Homer | 1e-859 | -1.980e+03 | 0.0000 | 3840.0 | 30.67% | 3663.6 | 10.23% | motif file (matrix) | svg |
| 11 | T C G A T A G C T G C A A C T G A C T G C G T A C G T A C T A G G A C T T A C G | ETS1(ETS)/Jurkat-ETS1-ChIP-Seq(GSE17954)/Homer | 1e-560 | -1.291e+03 | 0.0000 | 3151.0 | 25.17% | 3408.3 | 9.51% | motif file (matrix) | svg |
| 12 | A T G C A G T C C T G A A G T C C G A T A C G T A G T C A G T C A C G T A T C G G A C T A C G T | Etv2(ETS)/ES-ER71-ChIP-Seq(GSE59402)/Homer(0.967) | 1e-518 | -1.194e+03 | 0.0000 | 2889.0 | 23.08% | 3083.0 | 8.61% | motif file (matrix) | svg |
| 13 | A G T C C T G A A G T C C G A T C A G T G A T C A T G C A C T G A T C G G A C T | Fli1(ETS)/CD8-FLI-ChIP-Seq(GSE20898)/Homer | 1e-513 | -1.181e+03 | 0.0000 | 3043.0 | 24.31% | 3385.4 | 9.45% | motif file (matrix) | svg |
| 14 | T C G A T A G C G T C A A C T G A C T G C G T A C G T A C T A G A G C T T C A G | ERG(ETS)/VCaP-ERG-ChIP-Seq(GSE14097)/Homer | 1e-488 | -1.125e+03 | 0.0000 | 4005.0 | 31.99% | 5405.5 | 15.09% | motif file (matrix) | svg |
| 15 | T C G A C T G A T A G C T G A C T C A G T C A G C G T A C G T A T C A G A G C T | ETV1(ETS)/GIST48-ETV1-ChIP-Seq(GSE22441)/Homer | 1e-452 | -1.042e+03 | 0.0000 | 3541.0 | 28.29% | 4616.5 | 12.89% | motif file (matrix) | svg |
| 16 | C G A T T A C G T G A C G A C T C A T G C G T A T A C G A C G T G T A C C T G A | Bach2(bZIP)/OCILy7-Bach2-ChIP-Seq(GSE44420)/Homer | 1e-449 | -1.035e+03 | 0.0000 | 1243.0 | 9.93% | 723.4 | 2.02% | motif file (matrix) | svg |
| 17 | T G C A C T G A A T G C G T C A A C T G A C T G C G T A C G T A C T A G A G C T | Ets1-distal(ETS)/CD4+-PolII-ChIP-Seq(Barski\_et\_al.)/Homer | 1e-434 | -1.001e+03 | 0.0000 | 1503.0 | 12.01% | 1088.5 | 3.04% | motif file (matrix) | svg |
| 18 | T C A G C T G A C T A G C A T G A C G T A T G C C T G A C T G A C T G A C T A G C A T G A C G T A T G C C T G A | TR4(NR),DR1/Hela-TR4-ChIP-Seq(GSE24685)/Homer | 1e-421 | -9.701e+02 | 0.0000 | 957.0 | 7.64% | 445.0 | 1.24% | motif file (matrix) | svg |
| 19 | T C G A T C G A T A G C G T A C T C A G T A C G C G T A C G T A T C A G A G C T | GABPA(ETS)/Jurkat-GABPa-ChIP-Seq(GSE17954)/Homer | 1e-420 | -9.679e+02 | 0.0000 | 2584.0 | 20.64% | 2895.3 | 8.08% | motif file (matrix) | svg |
| 20 | T G C A C T G A A G T C G T C A A C T G A C T G C G T A C G T A C T G A A G C T | EWS:FLI1-fusion(ETS)/SK\_N\_MC-EWS:FLI1-ChIP-Seq(SRA014231)/Homer | 1e-358 | -8.264e+02 | 0.0000 | 1878.0 | 15.00% | 1872.3 | 5.23% | motif file (matrix) | svg |
| 21 | T C G A A G C T A C G T A C G T A G T C A G T C A C G T A T C G G A C T A T C G | EWS:ERG-fusion(ETS)/CADO\_ES1-EWS:ERG-ChIP-Seq(SRA014231)/Homer | 1e-357 | -8.227e+02 | 0.0000 | 2226.0 | 17.78% | 2488.1 | 6.94% | motif file (matrix) | svg |
| 22 | G A T C T C G A A G T C C G A T C G A T A G T C A T G C A C T G A T C G G A C T | Elk1(ETS)/Hela-Elk1-ChIP-Seq(GSE31477)/Homer | 1e-284 | -6.559e+02 | 0.0000 | 1465.0 | 11.70% | 1428.6 | 3.99% | motif file (matrix) | svg |
| 23 | A C G T C T A G C G T A A G T C G T A C A C G T A C G T A C G T G T C A G T A C T G A C G A C T | Nur77(NR)/K562-NR4A1-ChIP-Seq(GSE31363)/Homer | 1e-265 | -6.118e+02 | 0.0000 | 912.0 | 7.28% | 645.3 | 1.80% | motif file (matrix) | svg |
| 24 | G A T C C T G A A G T C C G A T C G A T G A T C A G T C A C T G A T C G A G C T | Elk4(ETS)/Hela-Elk4-ChIP-Seq(GSE31477)/Homer | 1e-238 | -5.492e+02 | 0.0000 | 1366.0 | 10.91% | 1421.1 | 3.97% | motif file (matrix) | svg |
| 25 | C G T A T A G C T A G C T G C A A C T G C T A G C G T A C G T A T C A G G A C T | EHF(ETS)/LoVo-EHF-ChIP-Seq(GSE49402)/Homer | 1e-211 | -4.878e+02 | 0.0000 | 2785.0 | 22.25% | 4391.2 | 12.26% | motif file (matrix) | svg |
| 26 | T G C A T C G A T A G C G T A C T C A G C T A G G T C A G C T A T C A G G A C T | ETS(ETS)/Promoter/Homer | 1e-211 | -4.868e+02 | 0.0000 | 983.0 | 7.85% | 884.4 | 2.47% | motif file (matrix) | svg |
| 27 | C G T A T G A C T A G C T G C A A C T G A C T G C G T A C G T A T C A G G A C T | ELF3(ETS)/PDAC-ELF3-ChIP-Seq(GSE64557)/Homer | 1e-210 | -4.855e+02 | 0.0000 | 1877.0 | 14.99% | 2490.0 | 6.95% | motif file (matrix) | svg |
| 28 | C G T A T A C G T C G A A C T G A C T G C G T A C G T A T A C G A G C T T A C G | PU.1(ETS)/ThioMac-PU.1-ChIP-Seq(GSE21512)/Homer | 1e-210 | -4.845e+02 | 0.0000 | 1531.0 | 12.23% | 1828.6 | 5.10% | motif file (matrix) | svg |
| 29 | A G T C C T G A A T C G A G C T A G C T G A C T A G T C G C T A A C G T C G A T G C A T C G A T A T C G C G T A T A G C G C A T A T G C C G T A | bZIP:IRF(bZIP,IRF)/Th17-BatF-ChIP-Seq(GSE39756)/Homer | 1e-193 | -4.452e+02 | 0.0000 | 1541.0 | 12.31% | 1926.3 | 5.38% | motif file (matrix) | svg |
| 30 | C T G A T G C A T A G C T G A C T A C G T C A G C T G A G C T A T C A G G A C T | ELF1(ETS)/Jurkat-ELF1-ChIP-Seq(SRA014231)/Homer | 1e-191 | -4.411e+02 | 0.0000 | 1232.0 | 9.84% | 1367.0 | 3.82% | motif file (matrix) | svg |
| 31 | T C G A T A G C G T C A A C T G C T A G C G T A C G A T A C T G A C G T A C T G A C T G A C G T | ETS:RUNX(ETS,Runt)/Jurkat-RUNX1-ChIP-Seq(GSE17954)/Homer | 1e-188 | -4.335e+02 | 0.0000 | 468.0 | 3.74% | 238.4 | 0.67% | motif file (matrix) | svg |
| 32 | G T A C G C T A T C A G C T G A C T A G C A T G A G C T G A T C T G C A T C G A C T G A A C T G C A G T A G T C G A T C G C T A | HNF4a(NR),DR1/HepG2-HNF4a-ChIP-Seq(GSE25021)/Homer | 1e-185 | -4.267e+02 | 0.0000 | 1399.0 | 11.18% | 1697.8 | 4.74% | motif file (matrix) | svg |
| 33 | C T G A A T G C C G T A A C G T A G T C A G T C A C G T A C T G A T C G G C A T | SPDEF(ETS)/VCaP-SPDEF-ChIP-Seq(SRA014231)/Homer | 1e-184 | -4.241e+02 | 0.0000 | 2302.0 | 18.39% | 3530.0 | 9.85% | motif file (matrix) | svg |
| 34 | A T G C G A C T A G C T A G C T A G T C G C T A C A G T C G A T G C T A A C G T A C T G G C T A T A G C G C A T T G A C | IRF:BATF(IRF:bZIP)/pDC-Irf8-ChIP-Seq(GSE66899)/Homer | 1e-182 | -4.194e+02 | 0.0000 | 616.0 | 4.92% | 427.3 | 1.19% | motif file (matrix) | svg |
| 35 | A C T G G A T C G A C T A C T G A C G T C A T G A C T G A C G T A G C T C G A T | RUNX-AML(Runt)/CD4+-PolII-ChIP-Seq(Barski\_et\_al.)/Homer | 1e-175 | -4.030e+02 | 0.0000 | 1960.0 | 15.66% | 2866.0 | 8.00% | motif file (matrix) | svg |
| 36 | G C T A C T G A T C G A A G T C A G T C C T G A A G T C G T C A C T G A T G C A | RUNX1(Runt)/Jurkat-RUNX1-ChIP-Seq(GSE29180)/Homer | 1e-173 | -3.998e+02 | 0.0000 | 2581.0 | 20.62% | 4220.1 | 11.78% | motif file (matrix) | svg |
| 37 | T G A C G C T A T C G A T G C A A G T C A G T C C G T A A G T C C G T A C T G A G C T A G T A C | RUNX2(Runt)/PCa-RUNX2-ChIP-Seq(GSE33889)/Homer | 1e-162 | -3.733e+02 | 0.0000 | 2301.0 | 18.38% | 3687.0 | 10.29% | motif file (matrix) | svg |
| 38 | T A G C G C T A T C G A C T G A A G T C A G T C C T G A A G T C C G T A C T A G | RUNX(Runt)/HPC7-Runx1-ChIP-Seq(GSE22178)/Homer | 1e-154 | -3.546e+02 | 0.0000 | 1937.0 | 15.47% | 2955.4 | 8.25% | motif file (matrix) | svg |
| 39 | G C T A A G T C T A C G T G C A A T C G T C A G G C T A T C G A T C A G A G C T | ELF5(ETS)/T47D-ELF5-ChIP-Seq(GSE30407)/Homer | 1e-137 | -3.171e+02 | 0.0000 | 1649.0 | 13.17% | 2457.8 | 6.86% | motif file (matrix) | svg |
| 40 | T A C G T C G A C A G T A C T G G C T A A T G C C G A T G T A C C G T A A C T G T A G C C G T A | NF-E2(bZIP)/K562-NFE2-ChIP-Seq(GSE31477)/Homer | 1e-121 | -2.787e+02 | 0.0000 | 366.0 | 2.92% | 228.8 | 0.64% | motif file (matrix) | svg |
| 41 | T C A G T A G C G A C T C A T G C T G A A T C G G C A T G T A C C G T A A C T G T A G C T G C A | MafK(bZIP)/C2C12-MafK-ChIP-Seq(GSE36030)/Homer | 1e-114 | -2.632e+02 | 0.0000 | 789.0 | 6.30% | 902.7 | 2.52% | motif file (matrix) | svg |
| 42 | C G T A C G T A C G T A G C A T G C A T T A C G G T A C G A C T A C T G C G T A A T C G A C G T G T A C C G T A A G C T | Bach1(bZIP)/K562-Bach1-ChIP-Seq(GSE31477)/Homer | 1e-105 | -2.427e+02 | 0.0000 | 332.0 | 2.65% | 215.7 | 0.60% | motif file (matrix) | svg |
| 43 | A G T C T G C A T C G A C T G A A C T G C A T G A C G T A T G C G T C A T A C G | Erra(NR)/HepG2-Erra-ChIP-Seq(GSE31477)/Homer | 1e-98 | -2.265e+02 | 0.0000 | 4043.0 | 32.29% | 8585.2 | 23.96% | motif file (matrix) | svg |
| 44 | T C A G T C A G G C T A C G T A T A C G G A C T T C A G T C G A C T G A C G T A T A C G G A C T | IRF8(IRF)/BMDM-IRF8-ChIP-Seq(GSE77884)/Homer | 1e-97 | -2.248e+02 | 0.0000 | 813.0 | 6.49% | 1023.9 | 2.86% | motif file (matrix) | svg |
| 45 | G T C A G C A T A C T G G T A C G A C T A C T G G C T A A T C G C A G T G T A C C G T A A G C T | Nrf2(bZIP)/Lymphoblast-Nrf2-ChIP-Seq(GSE37589)/Homer | 1e-97 | -2.242e+02 | 0.0000 | 303.0 | 2.42% | 195.0 | 0.54% | motif file (matrix) | svg |
| 46 | A G C T A T G C G A C T G C A T C G T A A G C T G T A C C G A T A T C G A G T C | Gata6(Zf)/HUG1N-GATA6-ChIP-Seq(GSE51936)/Homer | 1e-84 | -1.956e+02 | 0.0000 | 1785.0 | 14.26% | 3183.9 | 8.89% | motif file (matrix) | svg |
| 47 | C T A G T C G A C T G A C G T A T A C G G A C T T C A G T C G A G T C A T G C A T A C G A G C T | IRF2(IRF)/Erythroblas-IRF2-ChIP-Seq(GSE36985)/Homer | 1e-84 | -1.954e+02 | 0.0000 | 367.0 | 2.93% | 310.2 | 0.87% | motif file (matrix) | svg |
| 48 | C T G A T A C G G C A T A G C T A G C T A G T C T C G A A C T G C A G T A G C T A G C T G A T C | IRF3(IRF)/BMDM-Irf3-ChIP-Seq(GSE67343)/Homer | 1e-84 | -1.953e+02 | 0.0000 | 759.0 | 6.06% | 987.8 | 2.76% | motif file (matrix) | svg |
| 49 | A G C T A G T C A T G C A G C T A C G T C G T A A C G T A G T C C G A T A T G C | Gata2(Zf)/K562-GATA2-ChIP-Seq(GSE18829)/Homer | 1e-83 | -1.918e+02 | 0.0000 | 1358.0 | 10.85% | 2237.6 | 6.25% | motif file (matrix) | svg |
| 50 | T G A C C T A G T C A G G T C A C G T A T C A G C G A T T C A G T C G A T G C A C T G A T A G C | PU.1-IRF(ETS:IRF)/Bcell-PU.1-ChIP-Seq(GSE21512)/Homer | 1e-81 | -1.880e+02 | 0.0000 | 2499.0 | 19.96% | 4917.5 | 13.73% | motif file (matrix) | svg |
| 51 | G A C T G C A T C T A G C G A T G A T C T C G A C A T G G A T C | Tgif1(Homeobox)/mES-Tgif1-ChIP-Seq(GSE55404)/Homer | 1e-79 | -1.840e+02 | 0.0000 | 5709.0 | 45.60% | 13361.2 | 37.29% | motif file (matrix) | svg |
| 52 | A G C T C A T G G C A T G A T C T G C A C T A G G A T C A C G T | Tgif2(Homeobox)/mES-Tgif2-ChIP-Seq(GSE55404)/Homer | 1e-79 | -1.831e+02 | 0.0000 | 5891.0 | 47.06% | 13873.1 | 38.72% | motif file (matrix) | svg |
| 53 | T A G C G C T A A C T G C G T A A C G T C G T A C G T A T A C G T C A G T C G A | Gata1(Zf)/K562-GATA1-ChIP-Seq(GSE18829)/Homer | 1e-78 | -1.800e+02 | 0.0000 | 1208.0 | 9.65% | 1952.5 | 5.45% | motif file (matrix) | svg |
| 54 | C G T A C T G A C G T A C T A G T C G A C T A G A C T G C G T A C G T A T A C G A G C T A T C G | SpiB(ETS)/OCILY3-SPIB-ChIP-Seq(GSE56857)/Homer | 1e-73 | -1.702e+02 | 0.0000 | 585.0 | 4.67% | 716.6 | 2.00% | motif file (matrix) | svg |
| 55 | G A C T C T A G G A T C C A G T A C T G C T G A A T G C G C A T A T G C C T G A | MafA(bZIP)/Islet-MafA-ChIP-Seq(GSE30298)/Homer | 1e-69 | -1.592e+02 | 0.0000 | 1663.0 | 13.28% | 3064.9 | 8.55% | motif file (matrix) | svg |
| 56 | T A C G A T G C G C T A A C T G C G T A A C G T C G T A C T G A T A C G T C G A | Gata4(Zf)/Heart-Gata4-ChIP-Seq(GSE35151)/Homer | 1e-68 | -1.587e+02 | 0.0000 | 1864.0 | 14.89% | 3540.9 | 9.88% | motif file (matrix) | svg |
| 57 | G C T A C G T A A G T C A C G T T C G A T A C G A C T G A G C T A G T C T C G A | RORgt(NR)/EL4-RORgt.Flag-ChIP-Seq(GSE56019)/Homer | 1e-67 | -1.549e+02 | 0.0000 | 404.0 | 3.23% | 425.1 | 1.19% | motif file (matrix) | svg |
| 58 | T C A G C T G A C G T A C G T A T A C G G C A T C T A G C T G A C G T A C G T A T A C G G A C T | IRF1(IRF)/PBMC-IRF1-ChIP-Seq(GSE43036)/Homer | 1e-66 | -1.524e+02 | 0.0000 | 411.0 | 3.28% | 442.0 | 1.23% | motif file (matrix) | svg |
| 59 | T A C G C T G A T C G A C G A T C T A G C T A G T C G A C T G A T C G A T C G A C G T A T C G A G C A T C A T G C G T A T A C G G C A T T G A C C G T A A G C T | NFAT:AP1(RHD,bZIP)/Jurkat-NFATC1-ChIP-Seq(Jolma\_et\_al.)/Homer | 1e-65 | -1.504e+02 | 0.0000 | 446.0 | 3.56% | 505.2 | 1.41% | motif file (matrix) | svg |
| 60 | G C T A A T C G G C T A G A C T G C T A T C G A T A G C T C G A | GATA3(Zf)/iTreg-Gata3-ChIP-Seq(GSE20898)/Homer | 1e-64 | -1.494e+02 | 0.0000 | 2610.0 | 20.85% | 5419.7 | 15.13% | motif file (matrix) | svg |
| 61 | T C G A T C A G T C G A A C T G C A T G A C G T A G T C C T G A | COUP-TFII(NR)/Artia-Nr2f2-ChIP-Seq(GSE46497)/Homer | 1e-59 | -1.376e+02 | 0.0000 | 3124.0 | 24.95% | 6809.6 | 19.01% | motif file (matrix) | svg |
| 62 | C A T G A G C T T A C G G T C A G T A C T A G C A G C T G A C T A T C G T C G A | Esrrb(NR)/mES-Esrrb-ChIP-Seq(GSE11431)/Homer | 1e-57 | -1.328e+02 | 0.0000 | 1468.0 | 11.73% | 2735.2 | 7.63% | motif file (matrix) | svg |
| 63 | C T A G C T A G C G T A C G T A T A C G C G A T C T A G C T G A C T G A C G T A T A C G G A C T | PU.1:IRF8(ETS:IRF)/pDC-Irf8-ChIP-Seq(GSE66899)/Homer | 1e-53 | -1.239e+02 | 0.0000 | 471.0 | 3.76% | 605.5 | 1.69% | motif file (matrix) | svg |
| 64 | G C T A T A G C A G C T A T C G G T C A C G T A G C T A A T G C G A T C C T G A | IRF4(IRF)/GM12878-IRF4-ChIP-Seq(GSE32465)/Homer | 1e-51 | -1.184e+02 | 0.0000 | 991.0 | 7.92% | 1708.1 | 4.77% | motif file (matrix) | svg |
| 65 | C G T A C T A G C A T G A G C T A C T G C G A T A T C G C G T A G T C A G T C A | Tbet(T-box)/CD8-Tbet-ChIP-Seq(GSE33802)/Homer | 1e-49 | -1.150e+02 | 0.0000 | 2147.0 | 17.15% | 4486.6 | 12.52% | motif file (matrix) | svg |
| 66 | A T G C C T G A G A C T A C G T A C G T G T A C G A T C C G A T C T A G C A T G C G T A C G T A C T G A G A C T | STAT1(Stat)/HelaS3-STAT1-ChIP-Seq(GSE12782)/Homer | 1e-49 | -1.143e+02 | 0.0000 | 630.0 | 5.03% | 945.5 | 2.64% | motif file (matrix) | svg |
| 67 | T A G C G T A C A G T C G T A C C G A T A G T C A G T C A G T C A G T C A G T C C G T A G A T C | Zfp281(Zf)/ES-Zfp281-ChIP-Seq(GSE81042)/Homer | 1e-46 | -1.066e+02 | 0.0000 | 437.0 | 3.49% | 581.2 | 1.62% | motif file (matrix) | svg |
| 68 | C T G A A T C G A G C T A G C T A C G T T A G C C T G A T A C G C G A T A C G T G A C T A G T C | ISRE(IRF)/ThioMac-LPS-Expression(GSE23622)/Homer | 1e-44 | -1.033e+02 | 0.0000 | 220.0 | 1.76% | 204.5 | 0.57% | motif file (matrix) | svg |
| 69 | T A G C C T A G T C G A G A C T A C T G C T G A A G T C T C A G G C A T T G A C C T G A A G C T | Atf7(bZIP)/3T3L1-Atf7-ChIP-Seq(GSE56872)/Homer | 1e-41 | -9.564e+01 | 0.0000 | 930.0 | 7.43% | 1669.3 | 4.66% | motif file (matrix) | svg |
| 70 | C G T A G A C T C G A T A T C G G T A C G C A T C A T G C G T A T A C G G C A T G T A C C G T A C A T G A T G C G C T A C T A G G C A T G C A T G C A T G A C T | MafB(bZIP)/BMM-Mafb-ChIP-Seq(GSE75722)/Homer | 1e-40 | -9.229e+01 | 0.0000 | 942.0 | 7.52% | 1712.1 | 4.78% | motif file (matrix) | svg |
| 71 | T C G A G C A T A C T G C T G A A G T C T C A G G A C T G T A C C G T A A G C T A G T C G A T C | c-Jun-CRE(bZIP)/K562-cJun-ChIP-Seq(GSE31477)/Homer | 1e-39 | -9.080e+01 | 0.0000 | 635.0 | 5.07% | 1034.1 | 2.89% | motif file (matrix) | svg |
| 72 | C T G A A C G T A C G T A C G T A G T C G A C T C G A T C T G A A C T G C G T A C G T A T C G A | STAT5(Stat)/mCD4+-Stat5-ChIP-Seq(GSE12346)/Homer | 1e-38 | -8.852e+01 | 0.0000 | 694.0 | 5.54% | 1171.6 | 3.27% | motif file (matrix) | svg |
| 73 | C T G A C T G A C T A G T C G A C G T A A T G C C G T A A C T G C G T A A C G T C T G A C G A T A G C T C G T A A C G T A G T C C G A T T A C G G T C A G C A T | GATA(Zf),IR3/iTreg-Gata3-ChIP-Seq(GSE20898)/Homer | 1e-37 | -8.669e+01 | 0.0000 | 372.0 | 2.97% | 505.7 | 1.41% | motif file (matrix) | svg |
| 74 | T C A G T A C G T A G C A C G T A C T G C G A T A G T C C G T A T A C G A G T C | Meis1(Homeobox)/MastCells-Meis1-ChIP-Seq(GSE48085)/Homer | 1e-35 | -8.166e+01 | 0.0000 | 3145.0 | 25.12% | 7341.1 | 20.49% | motif file (matrix) | svg |
| 75 | C T G A C T A G A T C G A G C T A C T G G A C T A G T C C T G A | Tbx5(T-box)/HL1-Tbx5.biotin-ChIP-Seq(GSE21529)/Homer | 1e-35 | -8.107e+01 | 0.0000 | 5196.0 | 41.50% | 12934.6 | 36.10% | motif file (matrix) | svg |
| 76 | A T G C T C G A A G T C A G C T A C G T G T A C A G T C G C T A C T A G C A T G G T C A C T G A T C A G A G T C | Stat3+il21(Stat)/CD4-Stat3-ChIP-Seq(GSE19198)/Homer | 1e-33 | -7.810e+01 | 0.0000 | 1189.0 | 9.50% | 2370.1 | 6.62% | motif file (matrix) | svg |
| 77 | A T G C T C A G T C G A G C A T A C T G C G T A A G T C T C A G G A C T T G A C C G T A A G C T | Atf2(bZIP)/3T3L1-Atf2-ChIP-Seq(GSE56872)/Homer | 1e-32 | -7.583e+01 | 0.0000 | 665.0 | 5.31% | 1159.1 | 3.24% | motif file (matrix) | svg |
| 78 | T A C G T C G A G A C T A C T G C T G A A G T C T C A G G A C T T G A C C T G A | Atf1(bZIP)/K562-ATF1-ChIP-Seq(GSE31477)/Homer | 1e-32 | -7.385e+01 | 0.0000 | 1162.0 | 9.28% | 2331.9 | 6.51% | motif file (matrix) | svg |
| 79 | A T G C C G T A A C T G C G T A A C G T G C T A T C G A A G C T C G A T C G T A A C G T A G T C C G A T A C T G G A T C | GATA(Zf),IR4/iTreg-Gata3-ChIP-Seq(GSE20898)/Homer | 1e-31 | -7.346e+01 | 0.0000 | 211.0 | 1.69% | 236.8 | 0.66% | motif file (matrix) | svg |
| 80 | T C G A T G A C G C A T A G C T C A G T G A T C G C T A G A T C G A C T A C G T G C A T A G T C | PRDM1(Zf)/Hela-PRDM1-ChIP-Seq(GSE31477)/Homer | 1e-30 | -6.975e+01 | 0.0000 | 1069.0 | 8.54% | 2132.1 | 5.95% | motif file (matrix) | svg |
| 81 | T C A G A G C T A C G T A C G T G T A C G A T C C G T A C T A G C A T G G T C A C G T A T C G A | STAT4(Stat)/CD4-Stat4-ChIP-Seq(GSE22104)/Homer | 1e-29 | -6.873e+01 | 0.0000 | 1495.0 | 11.94% | 3186.8 | 8.90% | motif file (matrix) | svg |
| 82 | C T A G A C T G C T A G T C A G T C A G T A C G C T A G A C T G | Maz(Zf)/HepG2-Maz-ChIP-Seq(GSE31477)/Homer | 1e-29 | -6.702e+01 | 0.0000 | 1491.0 | 11.91% | 3189.8 | 8.90% | motif file (matrix) | svg |
| 83 | C T G A T C A G C A G T C T A G A C T G C T A G G A T C A T C G A C T G C T G A T C A G G A T C | Sp5(Zf)/mES-Sp5.Flag-ChIP-Seq(GSE72989)/Homer | 1e-28 | -6.487e+01 | 0.0000 | 1093.0 | 8.73% | 2221.6 | 6.20% | motif file (matrix) | svg |
| 84 | A T C G A G C T C T G A C T A G A C T G A C G T G T A C G C T A A T G C A C G T C T A G C A T G T A C G C G A T A T G C C G T A | Reverb(NR),DR2/RAW-Reverba.biotin-ChIP-Seq(GSE45914)/Homer | 1e-28 | -6.456e+01 | 0.0000 | 330.0 | 2.64% | 482.8 | 1.35% | motif file (matrix) | svg |
| 85 | A G T C A C G T A C T G A G C T A C G T A C G T G T C A A G T C | Foxo1(Forkhead)/RAW-Foxo1-ChIP-Seq(Fan\_et\_al.)/Homer | 1e-27 | -6.373e+01 | 0.0000 | 2927.0 | 23.38% | 6950.3 | 19.40% | motif file (matrix) | svg |
| 86 | T A C G C T A G A T G C G A T C G T A C A G T C C T A G A G T C A G T C A G T C G T A C A G T C | Sp1(Zf)/Promoter/Homer | 1e-26 | -6.140e+01 | 0.0000 | 238.0 | 1.90% | 310.8 | 0.87% | motif file (matrix) | svg |
| 87 | T C A G C T A G C T A G C T A G T C A G T C G A C T G A C G A T A G T C G A T C A G T C T G A C | NFkB-p50,p52(RHD)/Monocyte-p50-ChIP-Chip(Schreiber\_et\_al.)/Homer | 1e-25 | -5.911e+01 | 0.0000 | 178.0 | 1.42% | 205.4 | 0.57% | motif file (matrix) | svg |
| 88 | T C A G A G C T A T G C C G T A A G C T T C A G C A G T A C T G C T G A A G T C | MITF(bHLH)/MastCells-MITF-ChIP-Seq(GSE48085)/Homer | 1e-25 | -5.848e+01 | 0.0000 | 1631.0 | 13.03% | 3613.5 | 10.09% | motif file (matrix) | svg |
| 89 | C T A G T C A G C A G T T C A G A C T G A C T G G A T C C T A G A C T G C T A G T C A G A T G C | KLF14(Zf)/HEK293-KLF14.GFP-ChIP-Seq(GSE58341)/Homer | 1e-24 | -5.681e+01 | 0.0000 | 2024.0 | 16.17% | 4642.9 | 12.96% | motif file (matrix) | svg |
| 90 | C G T A C A T G C A T G A C T G C T A G T C G A G C A T C G A T A G C T A G T C G A T C G T A C | NFkB-p65(RHD)/GM12787-p65-ChIP-Seq(GSE19485)/Homer | 1e-22 | -5.231e+01 | 0.0000 | 696.0 | 5.56% | 1346.9 | 3.76% | motif file (matrix) | svg |
| 91 | G A C T C A G T G A T C G A T C A C G T G A T C C T G A T A C G C G T A G T C A | STAT6(Stat)/Macrophage-Stat6-ChIP-Seq(GSE38377)/Homer | 1e-20 | -4.766e+01 | 0.0000 | 916.0 | 7.32% | 1906.4 | 5.32% | motif file (matrix) | svg |
| 92 | G C A T T C A G C T G A A T C G A C T G C G A T G A T C C T G A | THRb(NR)/Liver-NR1A2-ChIP-Seq(GSE52613)/Homer | 1e-20 | -4.724e+01 | 0.0000 | 6327.0 | 50.54% | 16604.6 | 46.35% | motif file (matrix) | svg |
| 93 | A G T C G A C T C A G T G T A C A G T C A T C G T C A G A C T G G T C A C G T A | Stat3(Stat)/mES-Stat3-ChIP-Seq(GSE11431)/Homer | 1e-20 | -4.667e+01 | 0.0000 | 738.0 | 5.90% | 1480.8 | 4.13% | motif file (matrix) | svg |
| 94 | C A G T T C A G G A T C A C T G A C G T C T A G A C T G A C T G G A C T C T A G | Egr1(Zf)/K562-Egr1-ChIP-Seq(GSE32465)/Homer | 1e-20 | -4.612e+01 | 0.0000 | 920.0 | 7.35% | 1927.0 | 5.38% | motif file (matrix) | svg |
| 95 | G A C T G T A C T G C A A C G T G A T C G C T A T C G A A C G T A G T C C G T A | Pdx1(Homeobox)/Islet-Pdx1-ChIP-Seq(SRA008281)/Homer | 1e-19 | -4.450e+01 | 0.0000 | 1461.0 | 11.67% | 3306.8 | 9.23% | motif file (matrix) | svg |
| 96 | C T G A A G C T A C G T A C G T A G T C G A C T G A C T C T G A C T G A C T A G C G T A C G T A | STAT6(Stat)/CD4-Stat6-ChIP-Seq(GSE22104)/Homer | 1e-18 | -4.297e+01 | 0.0000 | 870.0 | 6.95% | 1827.8 | 5.10% | motif file (matrix) | svg |
| 97 | A C T G C A T G G C T A T C G A G C T A A G C T A G C T G T A C A G T C T G A C | NFkB-p65-Rel(RHD)/ThioMac-LPS-Expression(GSE23622)/Homer | 1e-18 | -4.224e+01 | 0.0000 | 124.0 | 0.99% | 142.4 | 0.40% | motif file (matrix) | svg |
| 98 | C T G A A T G C G C T A C G A T A T G C C G T A C G T A C G T A C T A G T A C G | Tcf3(HMG)/mES-Tcf3-ChIP-Seq(GSE11724)/Homer | 1e-18 | -4.216e+01 | 0.0000 | 506.0 | 4.04% | 957.9 | 2.67% | motif file (matrix) | svg |
| 99 | T G C A A G C T C T G A A T C G G A C T C T A G G T A C G A T C G T C A A G T C G T A C G A C T C T A G A T C G G C A T C A T G C A T G G A T C G T A C C T G A | CTCF(Zf)/CD4+-CTCF-ChIP-Seq(Barski\_et\_al.)/Homer | 1e-17 | -4.008e+01 | 0.0000 | 255.0 | 2.04% | 405.6 | 1.13% | motif file (matrix) | svg |
| 100 | C T A G T C G A C G A T C T A G G C A T C A G T C T A G G A T C C G T A G T C A | CEBP:AP1(bZIP)/ThioMac-CEBPb-ChIP-Seq(GSE21512)/Homer | 1e-16 | -3.898e+01 | 0.0000 | 1118.0 | 8.93% | 2481.5 | 6.93% | motif file (matrix) | svg |
| 101 | G A C T T C A G C T A G A G T C A G T C G T A C A G T C C T G A A G T C A G T C A G T C G A C T A G T C A C T G A T G C | KLF3(Zf)/MEF-Klf3-ChIP-Seq(GSE44748)/Homer | 1e-16 | -3.867e+01 | 0.0000 | 586.0 | 4.68% | 1166.2 | 3.26% | motif file (matrix) | svg |
| 102 | C G T A C T A G A C T G A C T G G A C T C T A G C A G T C T A G C A T G G A T C | KLF5(Zf)/LoVo-KLF5-ChIP-Seq(GSE49402)/Homer | 1e-16 | -3.772e+01 | 0.0000 | 1514.0 | 12.09% | 3513.9 | 9.81% | motif file (matrix) | svg |
| 103 | T C G A A C G T A C T G C T G A A G T C T C A G A G C T G T A C C G T A A G C T G A T C T C G A | JunD(bZIP)/K562-JunD-ChIP-Seq/Homer | 1e-15 | -3.529e+01 | 0.0000 | 211.0 | 1.69% | 329.7 | 0.92% | motif file (matrix) | svg |
| 104 | C T G A C T G A C A T G A T C G A G C T A T C G G A C T C A T G C T G A G T C A | Tbr1(T-box)/Cortex-Tbr1-ChIP-Seq(GSE71384)/Homer | 1e-14 | -3.295e+01 | 0.0000 | 2160.0 | 17.25% | 5282.1 | 14.74% | motif file (matrix) | svg |
| 105 | C T G A A T G C G C T A G C A T A T G C C G T A T C G A C T G A C T A G T C A G T A C G G T C A | Tcf4(HMG)/Hct116-Tcf4-ChIP-Seq(SRA012054)/Homer | 1e-12 | -2.917e+01 | 0.0000 | 820.0 | 6.55% | 1815.9 | 5.07% | motif file (matrix) | svg |
| 106 | A T G C A T C G T A C G A G C T A T C G C T G A A G T C C T A G A G C T A T G C C T G A A T G C | CRE(bZIP)/Promoter/Homer | 1e-12 | -2.877e+01 | 0.0000 | 360.0 | 2.88% | 690.8 | 1.93% | motif file (matrix) | svg |
| 107 | T C G A A C T G A C T G C G T A C G T A T C G A A G T C C T G A A T C G G T A C G C A T C A T G | ETS:E-box(ETS,bHLH)/HPC7-Scl-ChIP-Seq(GSE22178)/Homer | 1e-12 | -2.812e+01 | 0.0000 | 151.0 | 1.21% | 228.9 | 0.64% | motif file (matrix) | svg |
| 108 | A T G C A G T C G C A T A G C T A C G T T C A G C G A T A G C T G A T C A T C G | Sox10(HMG)/SciaticNerve-Sox3-ChIP-Seq(GSE35132)/Homer | 1e-11 | -2.759e+01 | 0.0000 | 2460.0 | 19.65% | 6174.0 | 17.23% | motif file (matrix) | svg |
| 109 | C G T A T A G C A G T C C T A G C A G T C T A G C T G A G T A C G C A T T C G A C G T A G C A T A G C T C T A G T C G A | PAX3:FKHR-fusion(Paired,Homeobox)/Rh4-PAX3:FKHR-ChIP-Seq(GSE19063)/Homer | 1e-11 | -2.673e+01 | 0.0000 | 369.0 | 2.95% | 723.9 | 2.02% | motif file (matrix) | svg |
| 110 | T G A C G C T A T G A C C G T A T C A G G A T C C G T A C A T G C A T G C T A G C T A G C T A G | Unknown-ESC-element(?)/mES-Nanog-ChIP-Seq(GSE11724)/Homer | 1e-11 | -2.598e+01 | 0.0000 | 772.0 | 6.17% | 1723.4 | 4.81% | motif file (matrix) | svg |
| 111 | T G C A A G C T A C G T C T A G G A T C C T A G G A T C G T C A C T G A A G T C | CEBP(bZIP)/ThioMac-CEBPb-ChIP-Seq(GSE21512)/Homer | 1e-10 | -2.493e+01 | 0.0000 | 844.0 | 6.74% | 1917.2 | 5.35% | motif file (matrix) | svg |
| 112 | G C T A G A C T G A C T T G C A C G T A A G T C C G T A T A G C G A T C G A C T | Eomes(T-box)/H9-Eomes-ChIP-Seq(GSE26097)/Homer | 1e-10 | -2.386e+01 | 0.0000 | 3262.0 | 26.06% | 8442.6 | 23.56% | motif file (matrix) | svg |
| 113 | T G C A A T G C A C G T A C G T A C G T A T G C C T A G A C G T A C G T A G C T G A T C A G C T | T1ISRE(IRF)/ThioMac-Ifnb-Expression/Homer | 1e-10 | -2.336e+01 | 0.0000 | 34.0 | 0.27% | 25.6 | 0.07% | motif file (matrix) | svg |
| 114 | T A C G T A G C G C T A C G A T C T A G A C G T C A G T C A G T G C T A A G T C G T C A G C A T | FOXK2(Forkhead)/U2OS-FOXK2-ChIP-Seq(E-MTAB-2204)/Homer | 1e-8 | -2.012e+01 | 0.0000 | 926.0 | 7.40% | 2183.8 | 6.10% | motif file (matrix) | svg |
| 115 | C T A G G T A C A G T C T G C A A G T C C T G A A G T C A G T C A G T C G C T A | Klf4(Zf)/mES-Klf4-ChIP-Seq(GSE11431)/Homer | 1e-8 | -1.941e+01 | 0.0000 | 491.0 | 3.92% | 1074.7 | 3.00% | motif file (matrix) | svg |
| 116 | C T A G C G T A G T C A C G T A A G T C G A T C A G C T C T A G C G T A A C G T G T C A G A T C | Six2(Homeobox)/NephronProgenitor-Six2-ChIP-Seq(GSE39837)/Homer | 1e-8 | -1.896e+01 | 0.0000 | 1485.0 | 11.86% | 3682.3 | 10.28% | motif file (matrix) | svg |
| 117 | C G T A A T G C C G A T A C G T A G T C C G T A C G T A C G T A C T A G A T C G | TCFL2(HMG)/K562-TCF7L2-ChIP-Seq(GSE29196)/Homer | 1e-8 | -1.886e+01 | 0.0000 | 174.0 | 1.39% | 313.6 | 0.88% | motif file (matrix) | svg |
| 118 | G T A C C A G T A C T G A C T G A C T G G A T C A C T G A C G T A C T G A C T G A G T C G A T C | KLF6(Zf)/PDAC-KLF6-ChIP-Seq(GSE64557)/Homer | 1e-8 | -1.871e+01 | 0.0000 | 1117.0 | 8.92% | 2704.5 | 7.55% | motif file (matrix) | svg |
| 119 | C G T A A C T G G T C A A C G T A T C G C A G T C T A G T C A G C G T A A C T G C G T A A C G T C G T A C T G A T A C G | GATA3(Zf),DR4/iTreg-Gata3-ChIP-Seq(GSE20898)/Homer | 1e-7 | -1.817e+01 | 0.0000 | 157.0 | 1.25% | 278.6 | 0.78% | motif file (matrix) | svg |
| 120 | T C A G T G A C G T A C T G C A G T A C C T A G G T A C A T G C A G T C G T C A A G T C G A C T | Klf9(Zf)/GBM-Klf9-ChIP-Seq(GSE62211)/Homer | 1e-7 | -1.774e+01 | 0.0000 | 462.0 | 3.69% | 1016.6 | 2.84% | motif file (matrix) | svg |
| 121 | C G A T C T A G A C G T G T C A C G T A C G T A A G T C C G T A | Foxo3(Forkhead)/U2OS-Foxo3-ChIP-Seq(E-MTAB-2701)/Homer | 1e-7 | -1.660e+01 | 0.0000 | 1055.0 | 8.43% | 2570.2 | 7.17% | motif file (matrix) | svg |
| 122 | T C G A G C A T A C G T C T A G G T A C T C G A G C A T T G A C T C G A A C G T | Chop(bZIP)/MEF-Chop-ChIP-Seq(GSE35681)/Homer | 1e-7 | -1.637e+01 | 0.0000 | 315.0 | 2.52% | 662.2 | 1.85% | motif file (matrix) | svg |
| 123 | A C T G C G T A A C T G A T G C T G A C G A T C A T C G T G C A A C T G A G T C | ZNF519(Zf)/HEK293-ZNF519.GFP-ChIP-Seq(GSE58341)/Homer | 1e-7 | -1.637e+01 | 0.0000 | 195.0 | 1.56% | 374.5 | 1.05% | motif file (matrix) | svg |
| 124 | A T G C G A C T A C G T C T A G A C G T A C G T A C G T C T G A G A T C G C T A A G C T C G T A | Foxa2(Forkhead)/Liver-Foxa2-ChIP-Seq(GSE25694)/Homer | 1e-7 | -1.627e+01 | 0.0000 | 1208.0 | 9.65% | 2983.2 | 8.33% | motif file (matrix) | svg |
| 125 | A G C T T G A C C G A T C G A T C T A G A C G T C A G T C A G T G C T A A G T C | FOXK1(Forkhead)/HEK293-FOXK1-ChIP-Seq(GSE51673)/Homer | 1e-6 | -1.575e+01 | 0.0000 | 1471.0 | 11.75% | 3699.7 | 10.33% | motif file (matrix) | svg |
| 126 | T A G C A G T C T G A C A G T C C T A G A T C G A G T C C A T G T G A C A G T C G T A C A G T C A G T C G C A T C T A G A T C G G C A T A C T G A T C G G A T C | BORIS(Zf)/K562-CTCFL-ChIP-Seq(GSE32465)/Homer | 1e-6 | -1.502e+01 | 0.0000 | 270.0 | 2.16% | 562.4 | 1.57% | motif file (matrix) | svg |
| 127 | T G C A A G C T C A T G C G T A A G C T A C T G G A T C G T C A C G T A A G C T | Atf4(bZIP)/MEF-Atf4-ChIP-Seq(GSE35681)/Homer | 1e-6 | -1.439e+01 | 0.0000 | 370.0 | 2.96% | 815.5 | 2.28% | motif file (matrix) | svg |
| 128 | T A C G G A C T A C T G A C T G C T A G A T G C A G T C A G T C A G T C C T G A | ZNF692(Zf)/HEK293-ZNF692.GFP-ChIP-Seq(GSE58341)/Homer | 1e-5 | -1.366e+01 | 0.0000 | 133.0 | 1.06% | 245.7 | 0.69% | motif file (matrix) | svg |
| 129 | A G T C T A G C G A C T A C G T C T A G A C G T A C G T A C G T C T G A A G T C G C T A G A C T C G T A C T A G A C T G | Foxa3(Forkhead)/Liver-Foxa3-ChIP-Seq(GSE77670)/Homer | 1e-5 | -1.224e+01 | 0.0000 | 470.0 | 3.75% | 1092.3 | 3.05% | motif file (matrix) | svg |
| 130 | G T A C C G T A C G T A T A C G G C A T G T A C C G T A C A T G A G T C C G T A C G T A C G A T G C A T G C A T G A C T | MafF(bZIP)/HepG2-MafF-ChIP-Seq(GSE31477)/Homer | 1e-5 | -1.223e+01 | 0.0000 | 380.0 | 3.04% | 861.7 | 2.41% | motif file (matrix) | svg |
| 131 | A T G C G A T C C G A T A C G T A C G T A C T G C A G T A G C T | Sox3(HMG)/NPC-Sox3-ChIP-Seq(GSE33059)/Homer | 1e-5 | -1.166e+01 | 0.0000 | 2514.0 | 20.08% | 6653.2 | 18.57% | motif file (matrix) | svg |
| 132 | C T G A T C A G G T A C G C T A A C T G T G A C G C A T C A T G | SCL(bHLH)/HPC7-Scl-ChIP-Seq(GSE13511)/Homer | 1e-4 | -1.125e+01 | 0.0000 | 5969.0 | 47.68% | 16409.3 | 45.80% | motif file (matrix) | svg |
| 133 | T C A G A G C T A T G C C G T A A G T C T C A G A C G T A T C G T C G A A G T C G A T C T G A C | TFE3(bHLH)/MEF-TFE3-ChIP-Seq(GSE75757)/Homer | 1e-4 | -1.086e+01 | 0.0001 | 141.0 | 1.13% | 279.2 | 0.78% | motif file (matrix) | svg |
| 134 | C T G A G T A C G A C T A G T C C A G T T G C A C T G A A C G T A G C T G A T C C T A G C G A T A C T G A T G C G A C T C T G A G A T C G A C T A G C T G A T C | Mouse\_Recombination\_Hotspot(Zf)/Testis-DMC1-ChIP-Seq(GSE24438)/Homer | 1e-4 | -1.084e+01 | 0.0001 | 105.0 | 0.84% | 195.0 | 0.54% | motif file (matrix) | svg |
| 135 | T C A G A C G T A G T C T C G A A G T C T C A G G C A T C T A G C T A G A G C T | Usf2(bHLH)/C2C12-Usf2-ChIP-Seq(GSE36030)/Homer | 1e-4 | -1.083e+01 | 0.0001 | 462.0 | 3.69% | 1088.5 | 3.04% | motif file (matrix) | svg |
| 136 | C T G A T C A G A G T C C G T A A T C G A T G C C G A T A C T G A G T C G A C T A T C G A G T C | MyoD(bHLH)/Myotube-MyoD-ChIP-Seq(GSE21614)/Homer | 1e-4 | -9.617e+00 | 0.0002 | 869.0 | 6.94% | 2187.8 | 6.11% | motif file (matrix) | svg |
| 137 | T C A G G A C T C A G T C T G A A G C T C T A G G A C T T G C A C T G A A G T C | HLF(bZIP)/HSC-HLF.Flag-ChIP-Seq(GSE69817)/Homer | 1e-3 | -9.193e+00 | 0.0003 | 1033.0 | 8.25% | 2640.0 | 7.37% | motif file (matrix) | svg |
| 138 | C T G A T C A G C T G A C T A G C A T G A C G T A T G C C G T A A T G C G C A T T C A G C T G A A C T G A C G T C A G T A G T C C G T A C A G T C T A G C A T G | VDR(NR),DR3/GM10855-VDR+vitD-ChIP-Seq(GSE22484)/Homer | 1e-3 | -9.009e+00 | 0.0003 | 319.0 | 2.55% | 739.8 | 2.07% | motif file (matrix) | svg |
| 139 | A C T G G A C T A G T C C T G A G A T C T C A G A T G C G A C T A G T C A T G C T A G C A G C T A T C G T G C A | PAX5(Paired,Homeobox),condensed/GM12878-PAX5-ChIP-Seq(GSE32465)/Homer | 1e-3 | -8.948e+00 | 0.0003 | 169.0 | 1.35% | 360.8 | 1.01% | motif file (matrix) | svg |
| 140 | A C G T T C G A T C G A A G T C G T C A T A C G A T G C A C G T A C T G A G C T | Myf5(bHLH)/GM-Myf5-ChIP-Seq(GSE24852)/Homer | 1e-3 | -8.754e+00 | 0.0004 | 847.0 | 6.77% | 2145.0 | 5.99% | motif file (matrix) | svg |
| 141 | A G T C G C A T C G T A C G T A G T A C A C G T A C T G G A T C G A T C T C G A | BMYB(HTH)/Hela-BMYB-ChIP-Seq(GSE27030)/Homer | 1e-3 | -8.670e+00 | 0.0004 | 2189.0 | 17.49% | 5836.7 | 16.29% | motif file (matrix) | svg |
| 142 | T C G A G C A T A T G C C T G A A T G C T A G C A G T C G T A C T C G A A G C T | Srebp1a(bHLH)/HepG2-Srebp1a-ChIP-Seq(GSE31477)/Homer | 1e-3 | -8.628e+00 | 0.0005 | 289.0 | 2.31% | 667.0 | 1.86% | motif file (matrix) | svg |
| 143 | C G A T C T A G T C G A A G C T C G A T C T G A C G T A A G C T A C T G C T A G A T G C G A T C | Hoxb4(Homeobox)/ES-Hoxb4-ChIP-Seq(GSE34014)/Homer | 1e-3 | -8.264e+00 | 0.0007 | 282.0 | 2.25% | 652.6 | 1.82% | motif file (matrix) | svg |
| 144 | C T A G C A T G T G C A A C G T A G T C C G T A C A T G T C A G A C G T A C G T G C T A A G T C | Six1(Homeobox)/Myoblast-Six1-ChIP-Chip(GSE20150)/Homer | 1e-3 | -8.033e+00 | 0.0008 | 371.0 | 2.96% | 886.8 | 2.48% | motif file (matrix) | svg |
| 145 | G C T A A G C T T A C G G T C A C T G A C G A T C T G A G C A T C A G T A G T C | Brn2(POU,Homeobox)/NPC-Brn2-ChIP-Seq(GSE35496)/Homer | 1e-3 | -7.906e+00 | 0.0009 | 123.0 | 0.98% | 255.5 | 0.71% | motif file (matrix) | svg |
| 146 | T A G C T A G C G A C T C T A G A G C T A G T C G T C A T G C A A C G T A T G C G C T A T G C A | Pbx3(Homeobox)/GM12878-PBX3-ChIP-Seq(GSE32465)/Homer | 1e-3 | -7.883e+00 | 0.0009 | 414.0 | 3.31% | 1001.5 | 2.80% | motif file (matrix) | svg |
| 147 | C G T A A C G T A C T G G T A C C G T A A C G T C G T A C G T A A C G T A C T G A G T C C G T A A C G T C T G A G C A T | OCT:OCT-short(POU,Homeobox)/NPC-OCT6-ChIP-Seq(GSE43916)/Homer | 1e-3 | -7.692e+00 | 0.0011 | 817.0 | 6.53% | 2084.7 | 5.82% | motif file (matrix) | svg |
| 148 | T A C G T A C G G T A C A T C G T A C G T A C G G T C A C T G A C G T A G A C T | E2F4(E2F)/K562-E2F4-ChIP-Seq(GSE31477)/Homer | 1e-3 | -7.616e+00 | 0.0012 | 238.0 | 1.90% | 547.0 | 1.53% | motif file (matrix) | svg |
| 149 | C A T G C T A G A G C T G A C T C A T G A G T C G A T C G C T A C G A T C T A G T C A G G T A C C T G A T C G A | X-box(HTH)/NPC-H3K4me1-ChIP-Seq(GSE16256)/Homer | 1e-3 | -7.552e+00 | 0.0013 | 160.0 | 1.28% | 349.9 | 0.98% | motif file (matrix) | svg |
| 150 | A G C T A G T C A G T C A C G T C T A G A C G T A C G T A C G T C G T A A G T C G A T C C G T A | FOXP1(Forkhead)/H9-FOXP1-ChIP-Seq(GSE31006)/Homer | 1e-3 | -7.215e+00 | 0.0018 | 585.0 | 4.67% | 1467.6 | 4.10% | motif file (matrix) | svg |
| 151 | T C G A C A T G C A T G A C G T A T G C T C G A C T G A A G C T T A C G T G C A G T A C G A T C A G C T A G T C | FXR(NR),IR1/Liver-FXR-ChIP-Seq(Chong\_et\_al.)/Homer | 1e-3 | -7.177e+00 | 0.0018 | 482.0 | 3.85% | 1192.1 | 3.33% | motif file (matrix) | svg |
| 152 | T C A G A T C G G A C T A C T G G A C T C A G T C T A G C G T A G T A C C G T A C T A G A T C G | Tbx20(T-box)/Heart-Tbx20-ChIP-Seq(GSE29636)/Homer | 1e-3 | -7.076e+00 | 0.0020 | 336.0 | 2.68% | 807.0 | 2.25% | motif file (matrix) | svg |
| 153 | G T A C C T A G T C A G A G C T T A G C C G T A A T G C T A C G A G T C G T A C G T C A A G T C | Srebp2(bHLH)/HepG2-Srebp2-ChIP-Seq(GSE31477)/Homer | 1e-2 | -6.769e+00 | 0.0027 | 179.0 | 1.43% | 404.2 | 1.13% | motif file (matrix) | svg |
| 154 | C A T G T A C G T A G C G A T C G A T C A T G C G T A C G A C T T C A G A T G C C G A T A T C G C A G T A C T G G T A C | Zic3(Zf)/mES-Zic3-ChIP-Seq(GSE37889)/Homer | 1e-2 | -6.747e+00 | 0.0028 | 561.0 | 4.48% | 1411.3 | 3.94% | motif file (matrix) | svg |
| 155 | G A C T C T A G C T A G C T A G A C T G T C G A C T G A C T A G C T A G C T A G G T A C G T C A | ZNF467(Zf)/HEK293-ZNF467.GFP-ChIP-Seq(GSE58341)/Homer | 1e-2 | -6.638e+00 | 0.0031 | 942.0 | 7.52% | 2448.1 | 6.83% | motif file (matrix) | svg |
| 156 | A G T C G A C T C A G T A C T G C T A G T G A C G C T A A T G C G C A T A T C G C G A T A C T G G A T C G T A C G T C A C T G A | NF1(CTF)/LNCAP-NF1-ChIP-Seq(Unpublished)/Homer | 1e-2 | -6.582e+00 | 0.0032 | 355.0 | 2.84% | 864.8 | 2.41% | motif file (matrix) | svg |
| 157 | A C G T T A C G G A T C A C T G A C G T C T A G A C T G A C T G G A T C C T A G C A T G C T A G | Egr2(Zf)/Thymocytes-Egr2-ChIP-Seq(GSE34254)/Homer | 1e-2 | -6.323e+00 | 0.0042 | 180.0 | 1.44% | 411.4 | 1.15% | motif file (matrix) | svg |
| 158 | C G T A A C T G C G T A A C G T A T C G C A G T T A G C C G T A T C G A G T A C C T G A T A G C C G T A A C T G C G T A A C G T C G T A C T G A A T C G G C T A | GATA3(Zf),DR8/iTreg-Gata3-ChIP-Seq(GSE20898)/Homer | 1e-2 | -6.237e+00 | 0.0045 | 149.0 | 1.19% | 333.3 | 0.93% | motif file (matrix) | svg |
| 159 | T A C G C G T A T C A G G A C T C T A G A C T G C A G T T A G C T C G A A C G T G T A C C T A G A G T C A G T C G A T C | ZNF669(Zf)/HEK293-ZNF669.GFP-ChIP-Seq(GSE58341)/Homer | 1e-2 | -6.226e+00 | 0.0045 | 89.0 | 0.71% | 185.0 | 0.52% | motif file (matrix) | svg |
| 160 | T G A C C G A T A C T G A C T G A C T G G A C T A C T G A C G T A C T G A C T G G A T C G A T C | EKLF(Zf)/Erythrocyte-Klf1-ChIP-Seq(GSE20478)/Homer | 1e-2 | -6.213e+00 | 0.0046 | 287.0 | 2.29% | 690.6 | 1.93% | motif file (matrix) | svg |
| 161 | A T G C C T G A A T C G T A C G A G T C C G A T T C A G C G A T C T A G A G C T G T C A G T C A C G T A A G T C C G T A T A C G C T G A | Fox:Ebox(Forkhead,bHLH)/Panc1-Foxa2-ChIP-Seq(GSE47459)/Homer | 1e-2 | -5.897e+00 | 0.0062 | 1607.0 | 12.84% | 4306.9 | 12.02% | motif file (matrix) | svg |
| 162 | G C A T C G A T C T A G G C T A G C A T C G T A G A T C G C T A C G A T C G A T C T A G C G A T C G T A C G A T G A T C | DMRT1(DM)/Testis-DMRT1-ChIP-Seq(GSE64892)/Homer | 1e-2 | -5.470e+00 | 0.0095 | 377.0 | 3.01% | 940.3 | 2.62% | motif file (matrix) | svg |
| 163 | C T A G T A C G G A T C G T A C G C T A A G C T A G C T G T C A T C G A T A G C | Nanog(Homeobox)/mES-Nanog-ChIP-Seq(GSE11724)/Homer | 1e-2 | -5.423e+00 | 0.0099 | 5529.0 | 44.16% | 15406.8 | 43.00% | motif file (matrix) | svg |
| 164 | T C A G C G T A A G T C A G C T C G T A A G T C C T G A C G T A A G T C G C A T A G T C A G T C A G T C C T G A A C T G T G C A T C G A C A T G A T C G G A T C | Ronin(THAP)/ES-Thap11-ChIP-Seq(GSE51522)/Homer | 1e-2 | -5.398e+00 | 0.0100 | 27.0 | 0.22% | 45.0 | 0.13% | motif file (matrix) | svg |
| 165 | C A T G A G T C A G C T C G T A C G A T C G A T G C A T G C A T C G A T C T G A C A T G T G A C | Mef2d(MADS)/Retina-Mef2d-ChIP-Seq(GSE61391)/Homer | 1e-2 | -5.342e+00 | 0.0106 | 184.0 | 1.47% | 432.8 | 1.21% | motif file (matrix) | svg |
| 166 | T A C G T A C G G T A C A T C G A C T G T A C G T C G A C T G A T C G A A T C G | E2F6(E2F)/Hela-E2F6-ChIP-Seq(GSE31477)/Homer | 1e-2 | -5.325e+00 | 0.0107 | 306.0 | 2.44% | 753.8 | 2.10% | motif file (matrix) | svg |
| 167 | A T G C G T A C C G T A A G C T G C A T T A C G A G C T A G C T A G T C A G C T | Sox6(HMG)/Myotubes-Sox6-ChIP-Seq(GSE32627)/Homer | 1e-2 | -5.044e+00 | 0.0141 | 2147.0 | 17.15% | 5847.2 | 16.32% | motif file (matrix) | svg |
| 168 | T G A C C T A G A C T G T A G C C G A T A C T G A T G C C A T G A T C G A T C G A T C G T A G C C T G A T A G C G C T A A C T G C G T A A G C T C G T A C T G A | GATA:SCL(Zf,bHLH)/Ter119-SCL-ChIP-Seq(GSE18720)/Homer | 1e-2 | -4.990e+00 | 0.0148 | 167.0 | 1.33% | 392.4 | 1.10% | motif file (matrix) | svg |
| 169 | C A G T T C A G A G C T G A C T A C G T A G T C G A T C G A C T C T G A A C T G G A T C C G T A C T G A A G T C G T A C | Rfx6(HTH)/Min6b1-Rfx6.HA-ChIP-Seq(GSE62844)/Homer | 1e-2 | -4.976e+00 | 0.0149 | 1320.0 | 10.54% | 3539.6 | 9.88% | motif file (matrix) | svg |
| 170 | A G T C G T A C A G C T C T A G A G T C C G A T A C T G C G T A A C T G G T C A | Zic(Zf)/Cerebellum-ZIC1.2-ChIP-Seq(GSE60731)/Homer | 1e-2 | -4.910e+00 | 0.0158 | 1081.0 | 8.63% | 2878.8 | 8.04% | motif file (matrix) | svg |
| 171 | T A C G A T G C G A C T A C T G A G C T A G T C G T C A T G C A A C G T A G T C G C T A T G C A | Pknox1(Homeobox)/ES-Prep1-ChIP-Seq(GSE63282)/Homer | 1e-2 | -4.842e+00 | 0.0168 | 358.0 | 2.86% | 900.9 | 2.51% | motif file (matrix) | svg |
| 172 | C T A G A T G C A T G C C G A T A C T G G A C T A T G C G C T A T G A C A G C T T A G C G C T A | PBX1(Homeobox)/MCF7-PBX1-ChIP-Seq(GSE28007)/Homer | 1e-2 | -4.770e+00 | 0.0179 | 156.0 | 1.25% | 367.0 | 1.02% | motif file (matrix) | svg |
| 173 | C T A G G C A T G A T C C G T A A G T C T C A G G A C T C T A G | CLOCK(bHLH)/Liver-Clock-ChIP-Seq(GSE39860)/Homer | 1e-2 | -4.752e+00 | 0.0182 | 739.0 | 5.90% | 1939.2 | 5.41% | motif file (matrix) | svg |
