# Supplementary material for: Genome-wide CRISPR Screens in T Helper Cells Reveal Pervasive Crosstalk between Activation and Differentiation
Source: Cell. 2019 Feb 7;176(4):882–896.e18. doi: 10.1016/j.cell.2018.11.044 (PMC6370901; doi:10.1016/j.cell.2018.11.044)
Supplement: Data S2. Processed Data from All the Steps of the Analysis, Related to Figure 1 [file mmc2.zip › supplemental data/motif analysis/Bhlhe40_homer/homerResults.html]

Bhlhe40\_motifs/ - Homer de novo Motif Results


# Homer *de novo* Motif Results (Bhlhe40\_motifs/)

Known Motif Enrichment Results  
Gene Ontology Enrichment Results  
If Homer is having trouble matching a motif to a known motif, try copy/pasting the matrix file into
STAMP  
More information on motif finding results: HOMER
| Description of Results
| Tips
  
Total target sequences = 1200  
Total background sequences = 42471  
\* - possible false positive  

|  |  |  |  |  |  |  |  |  |
| --- | --- | --- | --- | --- | --- | --- | --- | --- |
| Rank | Motif | P-value | log P-pvalue | % of Targets | % of Background | STD(Bg STD) | Best Match/Details | Motif File |
| 1 | A T C G A T C G C A T G T A G C C T G A G A T C C A T G A C G T A C T G G T A C | 1e-502 | -1.157e+03 | 47.25% | 3.14% | 47.7bp (79.6bp) | bHLHE40(bHLH)/HepG2-BHLHE40-ChIP-Seq(GSE31477)/Homer(0.991) More Information | Similar Motifs Found | motif file (matrix) |
| 2 | T C G A A C G T A C T G C G T A T A G C C G A T T G A C G T C A | 1e-39 | -9.086e+01 | 10.17% | 2.37% | 50.9bp (68.3bp) | AP-1(bZIP)/ThioMac-PU.1-ChIP-Seq(GSE21512)/Homer(0.969) More Information | Similar Motifs Found | motif file (matrix) |
| 3 | C A G T G C T A T C A G C T A G A G T C G A T C T G A C A T G C C T A G G A T C T A G C A G T C | 1e-28 | -6.600e+01 | 23.58% | 11.91% | 53.9bp (63.4bp) | POL003.1\_GC-box/Jaspar(0.928) More Information | Similar Motifs Found | motif file (matrix) |
| 4 | C T G A G A T C G A C T A C G T A G T C G A T C C G A T A C T G A G C T C A G T | 1e-23 | -5.423e+01 | 13.17% | 5.41% | 54.2bp (62.0bp) | Ets1-distal(ETS)/CD4+-PolII-ChIP-Seq(Barski\_et\_al.)/Homer(0.956) More Information | Similar Motifs Found | motif file (matrix) |
| 5 | A C T G A T G C A G T C G A T C A G T C A G T C A C G T A C T G T A C G A G C T A C T G T C A G | 1e-14 | -3.446e+01 | 2.92% | 0.52% | 58.5bp (67.2bp) | BORIS(Zf)/K562-CTCFL-ChIP-Seq(GSE32465)/Homer(0.921) More Information | Similar Motifs Found | motif file (matrix) |
| 6 | C G T A A C T G C G T A G T C A C G T A A G T C C G A T C G T A A G T C C G T A C G T A A G T C | 1e-14 | -3.354e+01 | 0.58% | 0.00% | 58.6bp (47.6bp) | PB0122.1\_Foxk1\_2/Jaspar(0.676) More Information | Similar Motifs Found | motif file (matrix) |
| 7 | T C A G A G T C A G T C C G T A C G T A A G C T T G A C T C A G A C T G G T C A | 1e-13 | -3.214e+01 | 4.58% | 1.33% | 54.8bp (61.4bp) | NFY(CCAAT)/Promoter/Homer(0.895) More Information | Similar Motifs Found | motif file (matrix) |
| 8 | A C G T A C T G C G T A A G T C C G T A A C T G A G T C G T C A A G C T A T G C T A C G C G A T | 1e-13 | -3.046e+01 | 0.67% | 0.01% | 62.2bp (49.1bp) | PH0158.1\_Rhox11\_2/Jaspar(0.781) More Information | Similar Motifs Found | motif file (matrix) |
| 9 | A G T C T C A G A T C G G A T C A C T G T A C G C G T A G T C A C T G A G A C T | 1e-12 | -2.814e+01 | 4.58% | 1.48% | 58.9bp (68.2bp) | Fli1(ETS)/CD8-FLI-ChIP-Seq(GSE20898)/Homer(0.769) More Information | Similar Motifs Found | motif file (matrix) |
| 10 | G A C T G T C A C T G A C T A G A C T G A T G C A C T G A G T C A C T G C A T G T G C A C G A T | 1e-12 | -2.773e+01 | 1.83% | 0.25% | 51.3bp (64.3bp) | PB0110.1\_Bcl6b\_2/Jaspar(0.715) More Information | Similar Motifs Found | motif file (matrix) |
| 11 \* | A C G T A G T C C A G T A G T C A C T G G T A C A C G T C G T A | 1e-10 | -2.458e+01 | 4.17% | 1.39% | 53.6bp (68.7bp) | GFX(?)/Promoter/Homer(0.686) More Information | Similar Motifs Found | motif file (matrix) |
| 12 \* | A C G T A G T C C A G T A C T G T G A C C A T G C G T A C T G A T G A C A G T C | 1e-10 | -2.439e+01 | 1.83% | 0.29% | 50.5bp (60.8bp) | Stat5a::Stat5b/MA0519.1/Jaspar(0.651) More Information | Similar Motifs Found | motif file (matrix) |
| 13 \* | A T C G G T A C C T A G T G A C T A C G A G T C C A T G G T A C G C T A A G T C G T C A A G T C | 1e-9 | -2.168e+01 | 4.58% | 1.76% | 46.9bp (90.2bp) | PB0095.1\_Zfp161\_1/Jaspar(0.742) More Information | Similar Motifs Found | motif file (matrix) |
| 14 \* | C G T A T A C G T C G A T G C A C T G A C T G A A T C G C G A T A G T C C G T A | 1e-9 | -2.165e+01 | 2.92% | 0.83% | 56.7bp (71.8bp) | Nr2e1/MA0676.1/Jaspar(0.822) More Information | Similar Motifs Found | motif file (matrix) |
| 15 \* | T G C A C G T A G T C A T G C A C A G T C T A G C G T A C G T A C G T A T A G C | 1e-9 | -2.137e+01 | 3.17% | 0.97% | 50.7bp (67.8bp) | IRF3(IRF)/BMDM-Irf3-ChIP-Seq(GSE67343)/Homer(0.873) More Information | Similar Motifs Found | motif file (matrix) |
| 16 \* | A C T G A T G C C T A G A G T C C G A T A G C T A C T G T G A C C G T A C G T A | 1e-8 | -1.933e+01 | 1.75% | 0.35% | 59.9bp (70.5bp) | CEBP:AP1(bZIP)/ThioMac-CEBPb-ChIP-Seq(GSE21512)/Homer(0.631) More Information | Similar Motifs Found | motif file (matrix) |
| 17 \* | A G T C A C G T A G T C A T G C A G T C A C T G A C T G A G T C C G T A A C T G A C T G A G T C | 1e-8 | -1.922e+01 | 0.42% | 0.01% | 50.4bp (16.6bp) | PB0205.1\_Zic1\_2/Jaspar(0.667) More Information | Similar Motifs Found | motif file (matrix) |
| 18 \* | A C G T A G C T A G T C A C G T A C T G A C G T A T C G A G T C C G T A A C T G G T C A A C G T | 1e-6 | -1.539e+01 | 0.58% | 0.03% | 48.0bp (47.0bp) | ZBTB18/MA0698.1/Jaspar(0.588) More Information | Similar Motifs Found | motif file (matrix) |
| 19 \* | A C T G A G T C A C T G A C G T A C T G A G C T T A G C C G T A A C T G A G C T | 1e-6 | -1.534e+01 | 2.75% | 0.98% | 58.5bp (73.2bp) | Meis1(Homeobox)/MastCells-Meis1-ChIP-Seq(GSE48085)/Homer(0.723) More Information | Similar Motifs Found | motif file (matrix) |
| 20 \* | A C G T C G T A A C G T C G T A A C G T A G T C A C T G A C G T A C T G A C G T A C G T A C G T | 1e-6 | -1.473e+01 | 0.33% | 0.01% | 31.2bp (36.8bp) | PH0044.1\_Homez/Jaspar(0.642) More Information | Similar Motifs Found | motif file (matrix) |
| 21 \* | A G C T G T A C A T C G A T C G A G T C C G A T G T C A A T C G T C A G A G T C | 1e-5 | -1.214e+01 | 1.25% | 0.30% | 62.5bp (67.2bp) | SMAD3/MA0795.1/Jaspar(0.615) More Information | Similar Motifs Found | motif file (matrix) |
| 22 \* | A G C T A T G C C G T A A G T C A C T G C T G A A C T G A C T G A G T C A T C G A C G T T G C A | 1e-4 | -1.050e+01 | 0.50% | 0.05% | 39.0bp (60.4bp) | Mitf/MA0620.1/Jaspar(0.617) More Information | Similar Motifs Found | motif file (matrix) |
| 23 \* | C G T A A C T G A G T C A C G T A C G T C G T A A C G T A G T C C G T A A G T C | 1e-4 | -9.265e+00 | 0.25% | 0.01% | 68.0bp (44.6bp) | Gata4(Zf)/Heart-Gata4-ChIP-Seq(GSE35151)/Homer(0.754) More Information | Similar Motifs Found | motif file (matrix) |
| 24 \* | A C G T A G T C A C G T A G T C A C G T A C G T A C G T A G T C A C G T A C G T A G T C A C G T | 1e-1 | -2.903e+00 | 0.08% | 0.01% | 22.6bp (41.5bp) | Sox4(HMG)/proB-Sox4-ChIP-Seq(GSE50066)/Homer(0.633) More Information | Similar Motifs Found | motif file (matrix) |
