# Supplementary material for: Genome-wide CRISPR Screens in T Helper Cells Reveal Pervasive Crosstalk between Activation and Differentiation
Source: Cell. 2019 Feb 7;176(4):882–896.e18. doi: 10.1016/j.cell.2018.11.044 (PMC6370901; doi:10.1016/j.cell.2018.11.044)
Supplement: Data S2. Processed Data from All the Steps of the Analysis, Related to Figure 1 [file mmc2.zip › supplemental data/motif analysis/Bhlhe40_homer/knownResults.html]

Bhlhe40\_motifs - Homer Known Motif Enrichment Results


# Homer Known Motif Enrichment Results (Bhlhe40\_motifs)

Homer *de novo* Motif Results  
Gene Ontology Enrichment Results  
Known Motif Enrichment Results (txt file)  
Total Target Sequences = 1200, Total Background Sequences = 42553

|  |  |  |  |  |  |  |  |  |  |  |  |
| --- | --- | --- | --- | --- | --- | --- | --- | --- | --- | --- | --- |
| Rank | Motif | Name | P-value | log P-pvalue | q-value (Benjamini) | # Target Sequences with Motif | % of Targets Sequences with Motif | # Background Sequences with Motif | % of Background Sequences with Motif | Motif File | SVG |
| 1 | C A T G G T A C C G T A A G T C C T A G A C G T A C T G G T A C A G T C A G C T | bHLHE40(bHLH)/HepG2-BHLHE40-ChIP-Seq(GSE31477)/Homer | 1e-411 | -9.466e+02 | 0.0000 | 606.0 | 50.50% | 2408.5 | 5.66% | motif file (matrix) | svg |
| 2 | C T A G G C A T G A T C C G T A A G T C T C A G G A C T C T A G | CLOCK(bHLH)/Liver-Clock-ChIP-Seq(GSE39860)/Homer | 1e-346 | -7.971e+02 | 0.0000 | 633.0 | 52.75% | 3537.1 | 8.32% | motif file (matrix) | svg |
| 3 | T C G A T A G C T G A C C T G A A G T C A C T G G A C T C A T G | c-Myc(bHLH)/LNCAP-cMyc-ChIP-Seq(Unpublished)/Homer | 1e-286 | -6.604e+02 | 0.0000 | 623.0 | 51.92% | 4311.3 | 10.14% | motif file (matrix) | svg |
| 4 | T A C G T C G A T A G C A G T C C G T A A G T C C T A G G C A T A C T G A T C G | n-Myc(bHLH)/mES-nMyc-ChIP-Seq(GSE11431)/Homer | 1e-281 | -6.489e+02 | 0.0000 | 621.0 | 51.75% | 4366.5 | 10.27% | motif file (matrix) | svg |
| 5 | C G A T T G C A G T A C C G T A A G T C C T A G G A C T C A T G | NPAS(bHLH)/Liver-NPAS-ChIP-Seq(GSE39860)/Homer | 1e-281 | -6.476e+02 | 0.0000 | 793.0 | 66.08% | 7966.0 | 18.73% | motif file (matrix) | svg |
| 6 | T A C G T C A G A G C T A T G C C G T A A G T C T C A G A C G T A C T G T C G A | USF1(bHLH)/GM12878-Usf1-ChIP-Seq(GSE32465)/Homer | 1e-280 | -6.451e+02 | 0.0000 | 528.0 | 44.00% | 2905.5 | 6.83% | motif file (matrix) | svg |
| 7 | C T A G C A G T T G A C C G T A G A T C T C A G G A C T C A T G | BMAL1(bHLH)/Liver-Bmal1-ChIP-Seq(GSE39860)/Homer | 1e-242 | -5.578e+02 | 0.0000 | 780.0 | 65.00% | 8734.6 | 20.54% | motif file (matrix) | svg |
| 8 | T C G A T G A C A G T C C G T A A G T C C T A G A C G T A C T G A C T G A G C T A G T C G C A T | Max(bHLH)/K562-Max-ChIP-Seq(GSE31477)/Homer | 1e-214 | -4.934e+02 | 0.0000 | 514.0 | 42.83% | 3769.2 | 8.86% | motif file (matrix) | svg |
| 9 | C T G A C A G T C T G A A G T C C T A G G A C T A T C G G T A C | HIF-1b(HLH)/T47D-HIF1b-ChIP-Seq(GSE59937)/Homer | 1e-195 | -4.513e+02 | 0.0000 | 715.0 | 59.58% | 8529.7 | 20.06% | motif file (matrix) | svg |
| 10 | A C T G T G A C G T A C C G T A A G T C T A C G A C G T A C T G G T C A A G T C | NPAS2(bHLH)/Liver-NPAS2-ChIP-Seq(GSE39860)/Homer | 1e-187 | -4.310e+02 | 0.0000 | 585.0 | 48.75% | 5763.4 | 13.55% | motif file (matrix) | svg |
| 11 | T C A G T C A G T A G C A G T C C T G A A G T C C T A G A C G T A C T G A T C G | c-Myc(bHLH)/mES-cMyc-ChIP-Seq(GSE11431)/Homer | 1e-118 | -2.719e+02 | 0.0000 | 343.0 | 28.58% | 2850.5 | 6.70% | motif file (matrix) | svg |
| 12 | T C A G A C G T A G T C T C G A A G T C T C A G G C A T C T A G C T A G A G C T | Usf2(bHLH)/C2C12-Usf2-ChIP-Seq(GSE36030)/Homer | 1e-117 | -2.716e+02 | 0.0000 | 269.0 | 22.42% | 1680.7 | 3.95% | motif file (matrix) | svg |
| 13 | A T G C A T G C A T C G T A C G A G C T A G T C G C T A A G T C T C A G G A C T A C T G T C G A | E-box(bHLH)/Promoter/Homer | 1e-102 | -2.368e+02 | 0.0000 | 176.0 | 14.67% | 727.3 | 1.71% | motif file (matrix) | svg |
| 14 | T C A G A G C T A T G C C G T A A G T C T C A G A C G T A T C G T C G A A G T C G A T C T G A C | TFE3(bHLH)/MEF-TFE3-ChIP-Seq(GSE75757)/Homer | 1e-88 | -2.047e+02 | 0.0000 | 149.0 | 12.42% | 592.5 | 1.39% | motif file (matrix) | svg |
| 15 | T C A G A G C T A T G C C G T A A G C T T C A G C A G T A C T G C T G A A G T C | MITF(bHLH)/MastCells-MITF-ChIP-Seq(GSE48085)/Homer | 1e-68 | -1.575e+02 | 0.0000 | 324.0 | 27.00% | 3971.7 | 9.34% | motif file (matrix) | svg |
| 16 | A C G T C T G A G A T C A T C G G A C T T C A G G T A C T A G C | HIF-1a(bHLH)/MCF7-HIF1a-ChIP-Seq(GSE28352)/Homer | 1e-60 | -1.392e+02 | 0.0000 | 206.0 | 17.17% | 1895.3 | 4.46% | motif file (matrix) | svg |
| 17 | A T G C A T C G T A C G A G C T A T C G C T G A A G T C C T A G A G C T A T G C C T G A A T G C | CRE(bZIP)/Promoter/Homer | 1e-41 | -9.650e+01 | 0.0000 | 170.0 | 14.17% | 1788.3 | 4.21% | motif file (matrix) | svg |
| 18 | C A G T T G C A A C G T A C T G C G T A A T C G C G A T T G A C C G T A A C G T | BATF(bZIP)/Th17-BATF-ChIP-Seq(GSE39756)/Homer | 1e-36 | -8.421e+01 | 0.0000 | 146.0 | 12.17% | 1510.7 | 3.55% | motif file (matrix) | svg |
| 19 | C T A G T C G A A C G T A C T G C G T A A T G C A C G T G T A C C G T A A G C T G A T C G T A C | Atf3(bZIP)/GBM-ATF3-ChIP-Seq(GSE33912)/Homer | 1e-34 | -7.964e+01 | 0.0000 | 143.0 | 11.92% | 1519.7 | 3.57% | motif file (matrix) | svg |
| 20 | C T A G T C G A G C A T C A T G G C T A T A G C C G A T G T A C C T G A A G C T | JunB(bZIP)/DendriticCells-Junb-ChIP-Seq(GSE36099)/Homer | 1e-34 | -7.951e+01 | 0.0000 | 130.0 | 10.83% | 1280.6 | 3.01% | motif file (matrix) | svg |
| 21 | T C G A A C G T C A T G G C T A T A G C C G A T G T A C G C T A A C G T A T G C | AP-1(bZIP)/ThioMac-PU.1-ChIP-Seq(GSE21512)/Homer | 1e-33 | -7.805e+01 | 0.0000 | 153.0 | 12.75% | 1737.8 | 4.09% | motif file (matrix) | svg |
| 22 | A C T G C T A G T C G A C G A T C A T G G C T A A T C G C G A T G T A C G C T A A G C T G T A C | Fra1(bZIP)/BT549-Fra1-ChIP-Seq(GSE46166)/Homer | 1e-32 | -7.578e+01 | 0.0000 | 126.0 | 10.50% | 1257.1 | 2.96% | motif file (matrix) | svg |
| 23 | C A T G C T A G T C G A A C G T A C T G C G T A T A G C C G A T T G A C C G T A A G C T G A T C | Fra2(bZIP)/Striatum-Fra2-ChIP-Seq(GSE43429)/Homer | 1e-31 | -7.338e+01 | 0.0000 | 116.0 | 9.67% | 1109.5 | 2.61% | motif file (matrix) | svg |
| 24 | T C G A T A G C T G C A A C T G A C T G C G T A C G T A C T A G G A C T T A C G | ETS1(ETS)/Jurkat-ETS1-ChIP-Seq(GSE17954)/Homer | 1e-30 | -6.925e+01 | 0.0000 | 284.0 | 23.67% | 4990.2 | 11.73% | motif file (matrix) | svg |
| 25 | T C G A A G T C C G T A A T C G T A G C A C G T A C T G A G C T A C G T A G T C | Ptf1a(bHLH)/Panc1-Ptf1a-ChIP-Seq(GSE47459)/Homer | 1e-29 | -6.818e+01 | 0.0000 | 538.0 | 44.83% | 12436.2 | 29.24% | motif file (matrix) | svg |
| 26 | C T A G T C G A C G A T A C T G C G T A T A C G A G C T T G A C G C T A A C G T G A T C T A G C | Fosl2(bZIP)/3T3L1-Fosl2-ChIP-Seq(GSE56872)/Homer | 1e-27 | -6.271e+01 | 0.0000 | 92.0 | 7.67% | 824.9 | 1.94% | motif file (matrix) | svg |
| 27 | T C G A T A G C G T C A A C T G A C T G C G T A C G T A C T A G A G C T T C A G | ERG(ETS)/VCaP-ERG-ChIP-Seq(GSE14097)/Homer | 1e-25 | -5.891e+01 | 0.0000 | 327.0 | 27.25% | 6509.5 | 15.31% | motif file (matrix) | svg |
| 28 | C T A G T C G A A C G T A C T G C G T A T A G C C G A T G T A C C G T A A G C T G A T C G T A C | Jun-AP1(bZIP)/K562-cJun-ChIP-Seq(GSE31477)/Homer | 1e-25 | -5.831e+01 | 0.0000 | 75.0 | 6.25% | 595.0 | 1.40% | motif file (matrix) | svg |
| 29 | A G T C C T G A A G T C C G A T C A G T G A T C A T G C A C T G A T C G G A C T | Fli1(ETS)/CD8-FLI-ChIP-Seq(GSE20898)/Homer | 1e-22 | -5.175e+01 | 0.0000 | 303.0 | 25.25% | 6115.2 | 14.38% | motif file (matrix) | svg |
| 30 | A T G C A G T C C T G A A G T C C G A T A C G T A G T C A G T C A C G T A T C G G A C T A C G T | Etv2(ETS)/ES-ER71-ChIP-Seq(GSE59402)/Homer(0.967) | 1e-20 | -4.826e+01 | 0.0000 | 216.0 | 18.00% | 3889.9 | 9.15% | motif file (matrix) | svg |
| 31 | T A C G C T A G A T G C G A T C G T A C A G T C C T A G A G T C A G T C A G T C G T A C A G T C | Sp1(Zf)/Promoter/Homer | 1e-19 | -4.591e+01 | 0.0000 | 222.0 | 18.50% | 4126.0 | 9.70% | motif file (matrix) | svg |
| 32 | T C G A T C G A T A G C G T A C T C A G T A C G C G T A C G T A T C A G A G C T | GABPA(ETS)/Jurkat-GABPa-ChIP-Seq(GSE17954)/Homer | 1e-18 | -4.312e+01 | 0.0000 | 240.0 | 20.00% | 4712.6 | 11.08% | motif file (matrix) | svg |
| 33 | T C G A C T G A T A G C T G A C T C A G T C A G C G T A C G T A T C A G A G C T | ETV1(ETS)/GIST48-ETV1-ChIP-Seq(GSE22441)/Homer | 1e-18 | -4.193e+01 | 0.0000 | 305.0 | 25.42% | 6594.9 | 15.51% | motif file (matrix) | svg |
| 34 | G A T C C T G A A G T C C G A T C G A T G A T C A G T C A C T G A T C G A G C T | Elk4(ETS)/Hela-Elk4-ChIP-Seq(GSE31477)/Homer | 1e-17 | -4.124e+01 | 0.0000 | 222.0 | 18.50% | 4296.3 | 10.10% | motif file (matrix) | svg |
| 35 | C T G A T C A G C A G T C T A G A C T G C T A G G A T C A T C G A C T G C T G A T C A G G A T C | Sp5(Zf)/mES-Sp5.Flag-ChIP-Seq(GSE72989)/Homer | 1e-17 | -4.074e+01 | 0.0000 | 392.0 | 32.67% | 9258.7 | 21.77% | motif file (matrix) | svg |
| 36 | C A T G A G T C C T G A T G A C A T C G G A C T G T C A A G T C T A G C G A T C | HIF2a(bHLH)/785\_O-HIF2a-ChIP-Seq(GSE34871)/Homer | 1e-17 | -3.949e+01 | 0.0000 | 150.0 | 12.50% | 2496.7 | 5.87% | motif file (matrix) | svg |
| 37 | C G A T T A C G T G A C G A C T C A T G C G T A T A C G A C G T G T A C C T G A | Bach2(bZIP)/OCILy7-Bach2-ChIP-Seq(GSE44420)/Homer | 1e-16 | -3.886e+01 | 0.0000 | 56.0 | 4.67% | 498.9 | 1.17% | motif file (matrix) | svg |
| 38 | T C G A A G C T A C G T A C G T A G T C A G T C A C G T A T C G G A C T A T C G | EWS:ERG-fusion(ETS)/CADO\_ES1-EWS:ERG-ChIP-Seq(SRA014231)/Homer | 1e-16 | -3.788e+01 | 0.0000 | 123.0 | 10.25% | 1894.9 | 4.46% | motif file (matrix) | svg |
| 39 | G A T C T C G A A G T C C G A T C G A T A G T C A T G C A C T G A T C G G A C T | Elk1(ETS)/Hela-Elk1-ChIP-Seq(GSE31477)/Homer | 1e-15 | -3.490e+01 | 0.0000 | 212.0 | 17.67% | 4276.2 | 10.06% | motif file (matrix) | svg |
| 40 | C G T A C T A G A C T G A C T G G A C T C T A G C A G T C T A G C A T G G A T C | KLF5(Zf)/LoVo-KLF5-ChIP-Seq(GSE49402)/Homer | 1e-14 | -3.394e+01 | 0.0000 | 426.0 | 35.50% | 10727.2 | 25.23% | motif file (matrix) | svg |
| 41 | T G C A A G C T C T G A A T C G G A C T C T A G G T A C G A T C G T C A A G T C G T A C G A C T C T A G A T C G G C A T C A T G C A T G G A T C G T A C C T G A | CTCF(Zf)/CD4+-CTCF-ChIP-Seq(Barski\_et\_al.)/Homer | 1e-13 | -3.183e+01 | 0.0000 | 61.0 | 5.08% | 684.4 | 1.61% | motif file (matrix) | svg |
| 42 | T G C A C T G A A G T C G T C A A C T G A C T G C G T A C G T A C T G A A G C T | EWS:FLI1-fusion(ETS)/SK\_N\_MC-EWS:FLI1-ChIP-Seq(SRA014231)/Homer | 1e-13 | -2.997e+01 | 0.0000 | 144.0 | 12.00% | 2651.2 | 6.23% | motif file (matrix) | svg |
| 43 | G A C T T C A G C T A G A G T C A G T C G T A C A G T C C T G A A G T C A G T C A G T C G A C T A G T C A C T G A T G C | KLF3(Zf)/MEF-Klf3-ChIP-Seq(GSE44748)/Homer | 1e-12 | -2.813e+01 | 0.0000 | 225.0 | 18.75% | 4952.8 | 11.65% | motif file (matrix) | svg |
| 44 | G T A C C A G T A C T G A C T G A C T G G A T C A C T G A C G T A C T G A C T G A G T C G A T C | KLF6(Zf)/PDAC-KLF6-ChIP-Seq(GSE64557)/Homer | 1e-11 | -2.590e+01 | 0.0000 | 344.0 | 28.67% | 8671.5 | 20.39% | motif file (matrix) | svg |
| 45 | C T A G T C A G C A G T T C A G A C T G A C T G G A T C C T A G A C T G C T A G T C A G A T G C | KLF14(Zf)/HEK293-KLF14.GFP-ChIP-Seq(GSE58341)/Homer | 1e-10 | -2.482e+01 | 0.0000 | 512.0 | 42.67% | 14214.5 | 33.43% | motif file (matrix) | svg |
| 46 | T G C A T C G A T A G C G T A C T C A G C T A G G T C A G C T A T C A G G A C T | ETS(ETS)/Promoter/Homer | 1e-10 | -2.479e+01 | 0.0000 | 130.0 | 10.83% | 2477.4 | 5.83% | motif file (matrix) | svg |
| 47 | T G C A C T G A A T G C G T C A A C T G A C T G C G T A C G T A C T A G A G C T | Ets1-distal(ETS)/CD4+-PolII-ChIP-Seq(Barski\_et\_al.)/Homer | 1e-10 | -2.458e+01 | 0.0000 | 69.0 | 5.75% | 990.5 | 2.33% | motif file (matrix) | svg |
| 48 | C T G A T G C A T A G C T G A C T A C G T C A G C T G A G C T A T C A G G A C T | ELF1(ETS)/Jurkat-ELF1-ChIP-Seq(SRA014231)/Homer | 1e-10 | -2.320e+01 | 0.0000 | 178.0 | 14.83% | 3859.6 | 9.08% | motif file (matrix) | svg |
| 49 | C T A G A C T G C T A G T C A G T C A G T A C G C T A G A C T G | Maz(Zf)/HepG2-Maz-ChIP-Seq(GSE31477)/Homer | 1e-9 | -2.268e+01 | 0.0000 | 403.0 | 33.58% | 10792.2 | 25.38% | motif file (matrix) | svg |
| 50 | C G T A T A G C T A G C T G C A A C T G C T A G C G T A C G T A T C A G G A C T | EHF(ETS)/LoVo-EHF-ChIP-Seq(GSE49402)/Homer | 1e-8 | -1.902e+01 | 0.0000 | 205.0 | 17.08% | 4877.3 | 11.47% | motif file (matrix) | svg |
| 51 | T C A G T G A C G T A C T G C A G T A C C T A G G T A C A T G C A G T C G T C A A G T C G A C T | Klf9(Zf)/GBM-Klf9-ChIP-Seq(GSE62211)/Homer | 1e-8 | -1.899e+01 | 0.0000 | 176.0 | 14.67% | 4026.8 | 9.47% | motif file (matrix) | svg |
| 52 | T A G C A G T C T G A C A G T C C T A G A T C G A G T C C A T G T G A C A G T C G T A C A G T C A G T C G C A T C T A G A T C G G C A T A C T G A T C G G A T C | BORIS(Zf)/K562-CTCFL-ChIP-Seq(GSE32465)/Homer | 1e-8 | -1.881e+01 | 0.0000 | 80.0 | 6.67% | 1410.8 | 3.32% | motif file (matrix) | svg |
| 53 | T C G A T A G C G T C A A C T G C T A G C G T A C G A T A C T G A C G T A C T G A C T G A C G T | ETS:RUNX(ETS,Runt)/Jurkat-RUNX1-ChIP-Seq(GSE17954)/Homer | 1e-8 | -1.848e+01 | 0.0000 | 36.0 | 3.00% | 419.3 | 0.99% | motif file (matrix) | svg |
| 54 | C T A G G T A C A G T C T G C A A G T C C T G A A G T C A G T C A G T C G C T A | Klf4(Zf)/mES-Klf4-ChIP-Seq(GSE11431)/Homer | 1e-7 | -1.721e+01 | 0.0000 | 137.0 | 11.42% | 3005.2 | 7.07% | motif file (matrix) | svg |
| 55 | T C G A C T A G A G T C A G T C C G T A C G T A A C G T T A G C T C A G T A C G | NFY(CCAAT)/Promoter/Homer | 1e-7 | -1.719e+01 | 0.0000 | 149.0 | 12.42% | 3348.6 | 7.87% | motif file (matrix) | svg |
| 56 | T A G C G C T A T C G A C T G A A G T C A G T C C T G A A G T C C G T A C T A G | RUNX(Runt)/HPC7-Runx1-ChIP-Seq(GSE22178)/Homer | 1e-7 | -1.714e+01 | 0.0000 | 121.0 | 10.08% | 2561.3 | 6.02% | motif file (matrix) | svg |
| 57 | T C A G C G T A A G T C A G C T C G T A A G T C C T G A C G T A A G T C G C A T A G T C A G T C A G T C C T G A A C T G T G C A T C G A C A T G A T C G G A T C | Ronin(THAP)/ES-Thap11-ChIP-Seq(GSE51522)/Homer | 1e-7 | -1.664e+01 | 0.0000 | 25.0 | 2.08% | 242.4 | 0.57% | motif file (matrix) | svg |
| 58 | C G T A T G A C T A G C T G C A A C T G A C T G C G T A C G T A T C A G G A C T | ELF3(ETS)/PDAC-ELF3-ChIP-Seq(GSE64557)/Homer | 1e-7 | -1.648e+01 | 0.0000 | 115.0 | 9.58% | 2427.8 | 5.71% | motif file (matrix) | svg |
| 59 | T G A C G C T A T C G A T G C A A G T C A G T C C G T A A G T C C G T A C T G A G C T A G T A C | RUNX2(Runt)/PCa-RUNX2-ChIP-Seq(GSE33889)/Homer | 1e-7 | -1.619e+01 | 0.0000 | 133.0 | 11.08% | 2946.2 | 6.93% | motif file (matrix) | svg |
| 60 | A T G C T C G A A G T C A G C T A C G T G T A C A G T C G C T A C T A G C A T G G T C A C T G A T C A G A G T C | Stat3+il21(Stat)/CD4-Stat3-ChIP-Seq(GSE19198)/Homer | 1e-6 | -1.568e+01 | 0.0000 | 107.0 | 8.92% | 2245.1 | 5.28% | motif file (matrix) | svg |
| 61 | C T G A A T G C C G T A A C G T A G T C A G T C A C G T A C T G A T C G G C A T | SPDEF(ETS)/VCaP-SPDEF-ChIP-Seq(SRA014231)/Homer | 1e-6 | -1.480e+01 | 0.0000 | 168.0 | 14.00% | 4047.7 | 9.52% | motif file (matrix) | svg |
| 62 | A G C T A G T C A T G C A G C T A C G T C G T A A C G T A G T C C G A T A T G C | Gata2(Zf)/K562-GATA2-ChIP-Seq(GSE18829)/Homer | 1e-6 | -1.473e+01 | 0.0000 | 68.0 | 5.67% | 1249.7 | 2.94% | motif file (matrix) | svg |
| 63 | A T C G A G C T A C T G A G T C A C T G A G T C C G T A A C G T A C T G A G T C A C T G A G T C | NRF(NRF)/Promoter/Homer | 1e-6 | -1.446e+01 | 0.0000 | 124.0 | 10.33% | 2784.4 | 6.55% | motif file (matrix) | svg |
| 64 | G C T A A G T C T A C G T G C A A T C G T C A G G C T A T C G A T C A G A G C T | ELF5(ETS)/T47D-ELF5-ChIP-Seq(GSE30407)/Homer | 1e-6 | -1.427e+01 | 0.0000 | 128.0 | 10.67% | 2909.3 | 6.84% | motif file (matrix) | svg |
| 65 | A T G C A G C T T C A G T G A C T C A G A T G C T G C A A C G T A T C G G A T C A C T G A G T C | NRF1(NRF)/MCF7-NRF1-ChIP-Seq(Unpublished)/Homer | 1e-5 | -1.297e+01 | 0.0000 | 112.0 | 9.33% | 2525.2 | 5.94% | motif file (matrix) | svg |
| 66 | T G A C C T A G T C A G G T C A C G T A T C A G C G A T T C A G T C G A T G C A C T G A T A G C | PU.1-IRF(ETS:IRF)/Bcell-PU.1-ChIP-Seq(GSE21512)/Homer | 1e-5 | -1.261e+01 | 0.0000 | 174.0 | 14.50% | 4382.9 | 10.31% | motif file (matrix) | svg |
| 67 | G C T A C T G A T C G A A G T C A G T C C T G A A G T C G T C A C T G A T G C A | RUNX1(Runt)/Jurkat-RUNX1-ChIP-Seq(GSE29180)/Homer | 1e-5 | -1.249e+01 | 0.0000 | 140.0 | 11.67% | 3369.5 | 7.92% | motif file (matrix) | svg |
| 68 | A C T G G A T C G A C T A C T G A C G T C A T G A C T G A C G T A G C T C G A T | RUNX-AML(Runt)/CD4+-PolII-ChIP-Seq(Barski\_et\_al.)/Homer | 1e-5 | -1.229e+01 | 0.0000 | 106.0 | 8.83% | 2392.5 | 5.63% | motif file (matrix) | svg |
| 69 | T C A G A G C T A C G T A C G T G T A C G A T C C G T A C T A G C A T G G T C A C G T A T C G A | STAT4(Stat)/CD4-Stat4-ChIP-Seq(GSE22104)/Homer | 1e-5 | -1.224e+01 | 0.0000 | 108.0 | 9.00% | 2452.2 | 5.77% | motif file (matrix) | svg |
| 70 | G T C A G C A T A C T G G T A C G A C T A C T G G C T A A T C G C A G T G T A C C G T A A G C T | Nrf2(bZIP)/Lymphoblast-Nrf2-ChIP-Seq(GSE37589)/Homer | 1e-5 | -1.215e+01 | 0.0000 | 14.0 | 1.17% | 111.6 | 0.26% | motif file (matrix) | svg |
| 71 | T A C G T C G A C A G T A C T G G C T A A T G C C G A T G T A C C G T A A C T G T A G C C G T A | NF-E2(bZIP)/K562-NFE2-ChIP-Seq(GSE31477)/Homer | 1e-5 | -1.205e+01 | 0.0000 | 15.0 | 1.25% | 128.1 | 0.30% | motif file (matrix) | svg |
| 72 | C G T A C G T A C G T A G C A T G C A T T A C G G T A C G A C T A C T G C G T A A T C G A C G T G T A C C G T A A G C T | Bach1(bZIP)/K562-Bach1-ChIP-Seq(GSE31477)/Homer | 1e-5 | -1.196e+01 | 0.0000 | 14.0 | 1.17% | 113.5 | 0.27% | motif file (matrix) | svg |
| 73 | A T G C C T G A G A C T A C G T A C G T G T A C G A T C C G A T C T A G C A T G C G T A C G T A C T G A G A C T | STAT1(Stat)/HelaS3-STAT1-ChIP-Seq(GSE12782)/Homer | 1e-4 | -1.137e+01 | 0.0001 | 42.0 | 3.50% | 714.1 | 1.68% | motif file (matrix) | svg |
| 74 | A G C T C A T G G C A T G A T C T G C A C T A G G A T C A C G T | Tgif2(Homeobox)/mES-Tgif2-ChIP-Seq(GSE55404)/Homer | 1e-4 | -1.134e+01 | 0.0001 | 382.0 | 31.83% | 11191.2 | 26.32% | motif file (matrix) | svg |
| 75 | T A G C G T A C A G T C G T A C C G A T A G T C A G T C A G T C A G T C A G T C C G T A G A T C | Zfp281(Zf)/ES-Zfp281-ChIP-Seq(GSE81042)/Homer | 1e-4 | -1.131e+01 | 0.0001 | 78.0 | 6.50% | 1658.5 | 3.90% | motif file (matrix) | svg |
| 76 | T G C A T A G C G A C T T G C A T G A C T G C A C G T A A G C T A G C T A G T C A G T C G T A C | GFY(?)/Promoter/Homer | 1e-4 | -1.126e+01 | 0.0001 | 26.0 | 2.17% | 350.1 | 0.82% | motif file (matrix) | svg |
| 77 | T A G C G C T A A C T G C G T A A C G T C G T A C G T A T A C G T C A G T C G A | Gata1(Zf)/K562-GATA1-ChIP-Seq(GSE18829)/Homer | 1e-4 | -1.119e+01 | 0.0001 | 57.0 | 4.75% | 1100.0 | 2.59% | motif file (matrix) | svg |
| 78 | T C G A C G T A A G T C A G C T C G T A A G T C T C G A G C T A G A C T C G A T A G T C A G T C A G T C C T G A T C A G T G C A T C G A C A G T A T C G A G T C | GFY-Staf(?,Zf)/Promoter/Homer | 1e-4 | -1.091e+01 | 0.0001 | 25.0 | 2.08% | 336.0 | 0.79% | motif file (matrix) | svg |
| 79 | T A C G A T G C G C T A A C T G C G T A A C G T C G T A C T G A T A C G T C G A | Gata4(Zf)/Heart-Gata4-ChIP-Seq(GSE35151)/Homer | 1e-4 | -1.084e+01 | 0.0001 | 87.0 | 7.25% | 1933.7 | 4.55% | motif file (matrix) | svg |
| 80 | T C G A G A C T A G C T T G A C A G C T G T A C T C A G G A T C A T C G T G C A A C T G C T G A | GFX(?)/Promoter/Homer | 1e-4 | -1.046e+01 | 0.0001 | 16.0 | 1.33% | 165.6 | 0.39% | motif file (matrix) | svg |
| 81 | A G C T A T G C G A C T G C A T C G T A A G C T G T A C C G A T A T C G A G T C | Gata6(Zf)/HUG1N-GATA6-ChIP-Seq(GSE51936)/Homer | 1e-4 | -1.025e+01 | 0.0002 | 78.0 | 6.50% | 1712.3 | 4.03% | motif file (matrix) | svg |
| 82 | C G T A T A C G T C G A A C T G A C T G C G T A C G T A T A C G A G C T T A C G | PU.1(ETS)/ThioMac-PU.1-ChIP-Seq(GSE21512)/Homer | 1e-4 | -9.910e+00 | 0.0002 | 77.0 | 6.42% | 1702.6 | 4.00% | motif file (matrix) | svg |
| 83 | T C A G T C A G G C T A C G T A T A C G G A C T T C A G T C G A C T G A C G T A T A C G G A C T | IRF8(IRF)/BMDM-IRF8-ChIP-Seq(GSE77884)/Homer | 1e-4 | -9.624e+00 | 0.0003 | 50.0 | 4.17% | 979.9 | 2.30% | motif file (matrix) | svg |
| 84 | C T G A A C G T A C G T A C G T A G T C G A C T C G A T C T G A A C T G C G T A C G T A T C G A | STAT5(Stat)/mCD4+-Stat5-ChIP-Seq(GSE12346)/Homer | 1e-4 | -9.288e+00 | 0.0004 | 44.0 | 3.67% | 836.2 | 1.97% | motif file (matrix) | svg |
| 85 | C T A G T C G A C T G A C G T A T A C G G A C T T C A G T C G A G T C A T G C A T A C G A G C T | IRF2(IRF)/Erythroblas-IRF2-ChIP-Seq(GSE36985)/Homer | 1e-3 | -9.081e+00 | 0.0005 | 21.0 | 1.75% | 289.8 | 0.68% | motif file (matrix) | svg |
| 86 | A G T C C T G A A T C G A G C T A G C T G A C T A G T C G C T A A C G T C G A T G C A T C G A T A T C G C G T A T A G C G C A T A T G C C G T A | bZIP:IRF(bZIP,IRF)/Th17-BatF-ChIP-Seq(GSE39756)/Homer | 1e-3 | -8.497e+00 | 0.0009 | 47.0 | 3.92% | 947.0 | 2.23% | motif file (matrix) | svg |
| 87 | T A C G T C G A G A C T A C T G C T G A A G T C T C A G G A C T T G A C C T G A | Atf1(bZIP)/K562-ATF1-ChIP-Seq(GSE31477)/Homer | 1e-3 | -7.871e+00 | 0.0016 | 105.0 | 8.75% | 2653.6 | 6.24% | motif file (matrix) | svg |
| 88 | C T G A T A C G G C A T A G C T A G C T A G T C T C G A A C T G C A G T A G C T A G C T G A T C | IRF3(IRF)/BMDM-Irf3-ChIP-Seq(GSE67343)/Homer | 1e-3 | -7.797e+00 | 0.0017 | 40.0 | 3.33% | 791.9 | 1.86% | motif file (matrix) | svg |
| 89 | T C A G T A G C G A C T C A T G C T G A A T C G G C A T G T A C C G T A A C T G T A G C T G C A | MafK(bZIP)/C2C12-MafK-ChIP-Seq(GSE36030)/Homer | 1e-3 | -7.586e+00 | 0.0021 | 38.0 | 3.17% | 747.4 | 1.76% | motif file (matrix) | svg |
| 90 | T A G C C T A G T C G A G A C T A C T G C T G A A G T C T C A G G C A T T G A C C T G A A G C T | Atf7(bZIP)/3T3L1-Atf7-ChIP-Seq(GSE56872)/Homer | 1e-3 | -7.432e+00 | 0.0024 | 79.0 | 6.58% | 1909.9 | 4.49% | motif file (matrix) | svg |
| 91 | G C T A C G T A A G T C A C G T T C G A T A C G A C T G A G C T A G T C T C G A | RORgt(NR)/EL4-RORgt.Flag-ChIP-Seq(GSE56019)/Homer | 1e-3 | -7.305e+00 | 0.0027 | 18.0 | 1.50% | 264.0 | 0.62% | motif file (matrix) | svg |
| 92 | C T A G C T A G C G T A C G T A T A C G C G A T C T A G C T G A C T G A C G T A T A C G G A C T | PU.1:IRF8(ETS:IRF)/pDC-Irf8-ChIP-Seq(GSE66899)/Homer | 1e-3 | -7.240e+00 | 0.0028 | 27.0 | 2.25% | 478.1 | 1.12% | motif file (matrix) | svg |
| 93 | G C T A T A G C A G C T A T C G G T C A C G T A G C T A A T G C G A T C C T G A | IRF4(IRF)/GM12878-IRF4-ChIP-Seq(GSE32465)/Homer | 1e-3 | -7.238e+00 | 0.0028 | 51.0 | 4.25% | 1116.1 | 2.62% | motif file (matrix) | svg |
| 94 | T G C A A G C T C A T G C G T A A G C T A C T G G A T C G T C A C G T A A G C T | Atf4(bZIP)/MEF-Atf4-ChIP-Seq(GSE35681)/Homer | 1e-3 | -7.181e+00 | 0.0029 | 31.0 | 2.58% | 581.9 | 1.37% | motif file (matrix) | svg |
| 95 | C G T A C T G A C G T A C T A G T C G A C T A G A C T G C G T A C G T A T A C G A G C T A T C G | SpiB(ETS)/OCILY3-SPIB-ChIP-Seq(GSE56857)/Homer | 1e-3 | -7.180e+00 | 0.0029 | 36.0 | 3.00% | 711.9 | 1.67% | motif file (matrix) | svg |
| 96 | G A C T C A G T G A T C G A T C A C G T G A T C C T G A T A C G C G T A G T C A | STAT6(Stat)/Macrophage-Stat6-ChIP-Seq(GSE38377)/Homer | 1e-3 | -7.150e+00 | 0.0030 | 55.0 | 4.58% | 1233.8 | 2.90% | motif file (matrix) | svg |
| 97 | C T G A C T G A C T A G T C G A C G T A A T G C C G T A A C T G C G T A A C G T C T G A C G A T A G C T C G T A A C G T A G T C C G A T T A C G G T C A G C A T | GATA(Zf),IR3/iTreg-Gata3-ChIP-Seq(GSE20898)/Homer | 1e-3 | -7.053e+00 | 0.0032 | 15.0 | 1.25% | 203.1 | 0.48% | motif file (matrix) | svg |
| 98 | A T G C G A C T A G C T A G C T A G T C G C T A C A G T C G A T G C T A A C G T A C T G G C T A T A G C G C A T T G A C | IRF:BATF(IRF:bZIP)/pDC-Irf8-ChIP-Seq(GSE66899)/Homer | 1e-3 | -7.034e+00 | 0.0033 | 16.0 | 1.33% | 225.1 | 0.53% | motif file (matrix) | svg |
| 99 | A G T C A T C G C T A G A G C T G A C T C T A G A G T C A G T C G C T A C A G T T C A G T C A G G A T C C T G A T C G A G A T C | RFX(HTH)/K562-RFX3-ChIP-Seq(SRA012198)/Homer | 1e-3 | -7.031e+00 | 0.0033 | 27.0 | 2.25% | 485.3 | 1.14% | motif file (matrix) | svg |
| 100 | C G T A C A T G C A T G A C T G C T A G T C G A G C A T C G A T A G C T A G T C G A T C G T A C | NFkB-p65(RHD)/GM12787-p65-ChIP-Seq(GSE19485)/Homer | 1e-3 | -6.971e+00 | 0.0034 | 59.0 | 4.92% | 1357.0 | 3.19% | motif file (matrix) | svg |
| 101 | T C A G C T G A C G T A C G T A T A C G G C A T C T A G C T G A C G T A C G T A T A C G G A C T | IRF1(IRF)/PBMC-IRF1-ChIP-Seq(GSE43036)/Homer | 1e-2 | -6.815e+00 | 0.0040 | 21.0 | 1.75% | 345.2 | 0.81% | motif file (matrix) | svg |
| 102 | G C T A A T C G G C T A G A C T G C T A T C G A T A G C T C G A | GATA3(Zf)/iTreg-Gata3-ChIP-Seq(GSE20898)/Homer | 1e-2 | -6.808e+00 | 0.0040 | 115.0 | 9.58% | 3048.8 | 7.17% | motif file (matrix) | svg |
| 103 | C T A G C G T A G T C A C G T A A G T C G A T C A G C T C T A G C G T A A C G T G T C A G A T C | Six2(Homeobox)/NephronProgenitor-Six2-ChIP-Seq(GSE39837)/Homer | 1e-2 | -6.797e+00 | 0.0040 | 99.0 | 8.25% | 2557.2 | 6.01% | motif file (matrix) | svg |
| 104 | A G T C G C A T C G T A C G T A G T A C A C G T A C T G G A T C G A T C T C G A | BMYB(HTH)/Hela-BMYB-ChIP-Seq(GSE27030)/Homer | 1e-2 | -6.413e+00 | 0.0057 | 189.0 | 15.75% | 5442.8 | 12.80% | motif file (matrix) | svg |
| 105 | C T A G A G C T G A C T C A T G A G T C A G T C G T C A C A G T C T A G T C A G G T A C C T G A T C G A G A T C T G A C | Rfx2(HTH)/LoVo-RFX2-ChIP-Seq(GSE49402)/Homer | 1e-2 | -6.381e+00 | 0.0059 | 27.0 | 2.25% | 508.4 | 1.20% | motif file (matrix) | svg |
| 106 | T A C G T A C G G T A C A T C G T A C G T A C G G T C A C T G A C G T A G A C T | E2F4(E2F)/K562-E2F4-ChIP-Seq(GSE31477)/Homer | 1e-2 | -6.143e+00 | 0.0074 | 145.0 | 12.08% | 4058.9 | 9.54% | motif file (matrix) | svg |
| 107 | G C A T A T C G C A T G G T A C G C T A A G T C T C A G T G A C G T C A T G C A | Arnt:Ahr(bHLH)/MCF7-Arnt-ChIP-Seq(Lo\_et\_al.)/Homer | 1e-2 | -5.989e+00 | 0.0085 | 171.0 | 14.25% | 4911.9 | 11.55% | motif file (matrix) | svg |
| 108 | T C A G C A T G C A T G A C T G A C T G A G C T A C T G A C G T A C T G C A G T A T G C A G T C | KLF10(Zf)/HEK293-KLF10.GFP-ChIP-Seq(GSE58341)/Homer | 1e-2 | -5.799e+00 | 0.0102 | 213.0 | 17.75% | 6307.6 | 14.83% | motif file (matrix) | svg |
| 109 | T G A C C G A T A C T G A C T G A C T G G A C T A C T G A C G T A C T G A C T G G A T C G A T C | EKLF(Zf)/Erythrocyte-Klf1-ChIP-Seq(GSE20478)/Homer | 1e-2 | -5.597e+00 | 0.0124 | 46.0 | 3.83% | 1066.9 | 2.51% | motif file (matrix) | svg |
| 110 | A T G C T C A G T C G A G C A T A C T G C G T A A G T C T C A G G A C T T G A C C G T A A G C T | Atf2(bZIP)/3T3L1-Atf2-ChIP-Seq(GSE56872)/Homer | 1e-2 | -5.521e+00 | 0.0132 | 56.0 | 4.67% | 1362.3 | 3.20% | motif file (matrix) | svg |
| 111 | T C G A G C A T A C G T C T A G G T A C T C G A G C A T T G A C T C G A A C G T | Chop(bZIP)/MEF-Chop-ChIP-Seq(GSE35681)/Homer | 1e-2 | -5.127e+00 | 0.0195 | 22.0 | 1.83% | 426.3 | 1.00% | motif file (matrix) | svg |
| 112 | T C G A G C A T A C T G C T G A A G T C T C A G G A C T G T A C C G T A A G C T A G T C G A T C | c-Jun-CRE(bZIP)/K562-cJun-ChIP-Seq(GSE31477)/Homer | 1e-2 | -5.047e+00 | 0.0209 | 45.0 | 3.75% | 1071.8 | 2.52% | motif file (matrix) | svg |
| 113 | A G T C G A C T C A G T G T A C A G T C A T C G T C A G A C T G G T C A C G T A | Stat3(Stat)/mES-Stat3-ChIP-Seq(GSE11431)/Homer | 1e-2 | -5.039e+00 | 0.0209 | 70.0 | 5.83% | 1819.9 | 4.28% | motif file (matrix) | svg |
| 114 | C G T A T A G C A G T C C T A G C A G T C T A G C T G A G T A C G C A T T C G A C G T A G C A T A G C T C T A G T C G A | PAX3:FKHR-fusion(Paired,Homeobox)/Rh4-PAX3:FKHR-ChIP-Seq(GSE19063)/Homer | 1e-2 | -4.811e+00 | 0.0260 | 20.0 | 1.67% | 386.2 | 0.91% | motif file (matrix) | svg |
| 115 | T C G A A C G T A C T G C T G A A G T C T C A G A G C T G T A C C G T A A G C T G A T C T C G A | JunD(bZIP)/K562-JunD-ChIP-Seq/Homer | 1e-2 | -4.771e+00 | 0.0268 | 26.0 | 2.17% | 548.5 | 1.29% | motif file (matrix) | svg |
| 116 | T A C G T C A G T G C A A G C T T G A C A G C T A G T C A C T G G A T C A C T G T C G A A C T G C T G A C T G A A T G C | ZBTB33(Zf)/GM12878-ZBTB33-ChIP-Seq(GSE32465)/Homer | 1e-2 | -4.606e+00 | 0.0313 | 23.0 | 1.92% | 474.7 | 1.12% | motif file (matrix) | svg |
