# Supplementary material for: Genome-wide CRISPR Screens in T Helper Cells Reveal Pervasive Crosstalk between Activation and Differentiation
Source: Cell. 2019 Feb 7;176(4):882–896.e18. doi: 10.1016/j.cell.2018.11.044 (PMC6370901; doi:10.1016/j.cell.2018.11.044)
Supplement: Data S2. Processed Data from All the Steps of the Analysis, Related to Figure 1 [file mmc2.zip › supplemental data/motif analysis/Stat6_SRX021632_homer/homerResults/motif19.similar.html]

motif19

## Information for motif19

T
G
C
A
G
C
T
A
A
C
G
T
T
G
A
C
G
C
A
T
A
T
G
C
A
T
C
G
G
A
T
C
T
C
A
G
T
G
C
A
  
Reverse Opposite:  

A
C
G
T
A
G
T
C
C
T
A
G
T
A
G
C
T
A
C
G
C
G
T
A
A
C
T
G
T
G
C
A
C
G
A
T
A
C
G
T
  

|  |  |
| --- | --- |
| p-value: | 1e-38 |
| log p-value: | -8.929e+01 |
| Information Content per bp: | 1.452 |
| Number of Target Sequences with motif | 235.0 |
| Percentage of Target Sequences with motif | 0.93% |
| Number of Background Sequences with motif | 83.2 |
| Percentage of Background Sequences with motif | 0.34% |
| Average Position of motif in Targets | 100.4 +/- 52.5bp |
| Average Position of motif in Background | 84.6 +/- 64.5bp |
| Strand Bias (log2 ratio + to - strand density) | -0.1 |
| Multiplicity (# of sites on avg that occur together) | 1.08 |
| Motif File: | file (matrix) reverse opposite |

### Similar de novo motifs found

|  |  |  |  |  |  |  |  |
| --- | --- | --- | --- | --- | --- | --- | --- |
| Rank | Match Score | Redundant Motif | P-value | log P-value | % of Targets | % of Background | Motif file |
| 1 | 0.832 | T C A G G T A C G A C T T G A C G A C T A G T C A C T G G A T C A C T G T C G A A C T G T C G A | 1e-36 | -84.021642 | 0.72% | 0.24% | motif file (matrix) |
| 2 | 0.913 | A C G T G T A C A C G T A G T C A C T G A G T C A C T G C G T A | 1e-35 | -82.235290 | 1.48% | 0.72% | motif file (matrix) |
