# Supplementary figures and images for: Genome-wide CRISPR Screens in T Helper Cells Reveal Pervasive Crosstalk between Activation and Differentiation
Source: Cell. 2019 Feb 7;176(4):882–896.e18. doi: 10.1016/j.cell.2018.11.044 (PMC6370901; doi:10.1016/j.cell.2018.11.044)

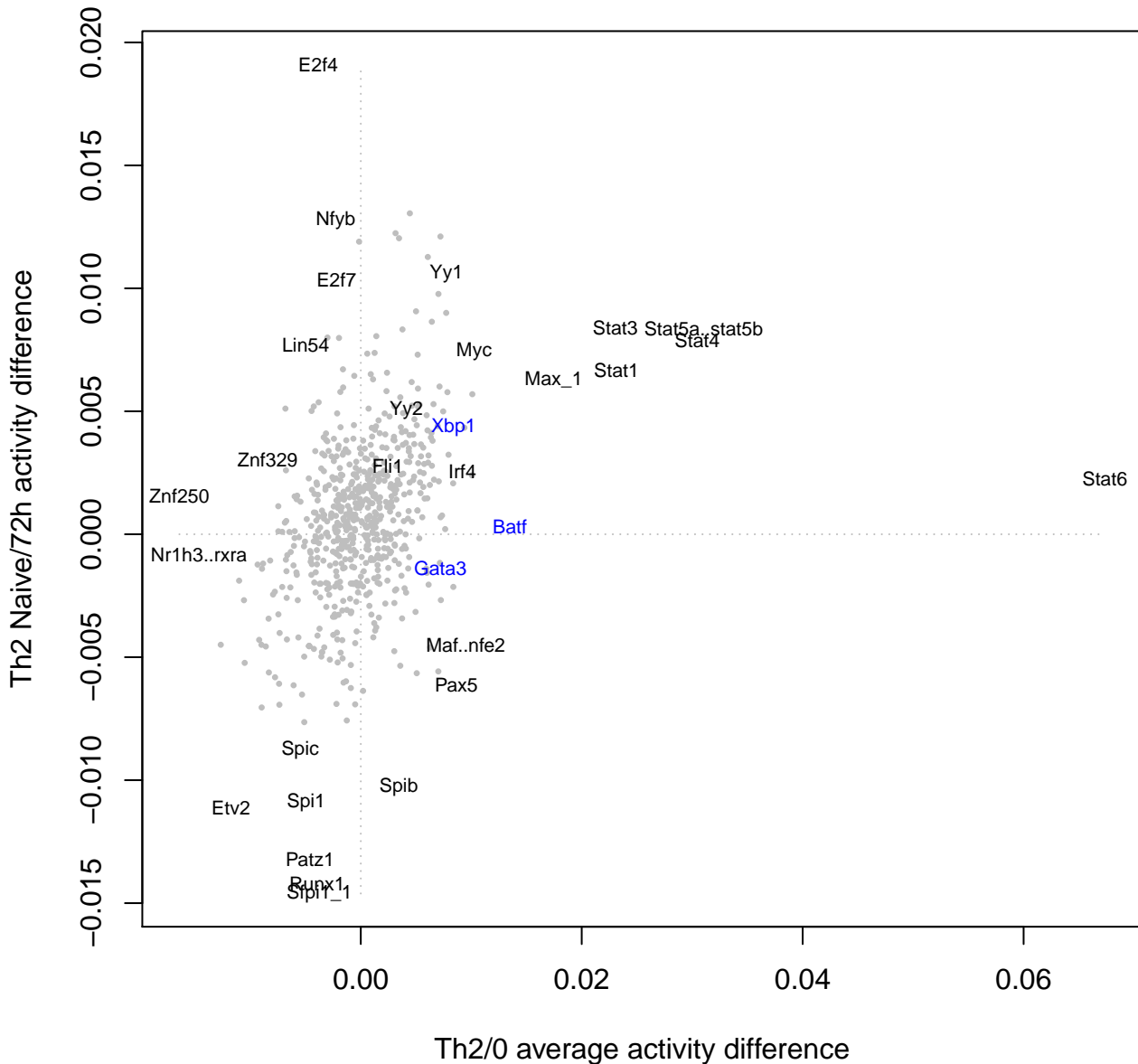

Supplement: Data S2. Processed Data from All the Steps of the Analysis, Related to Figure 1 [file mmc2.zip › supplemental data/mara/mouse_alltf_allgenes_alltime_th20_nonconsP/plot_diffvsdiff.pdf]

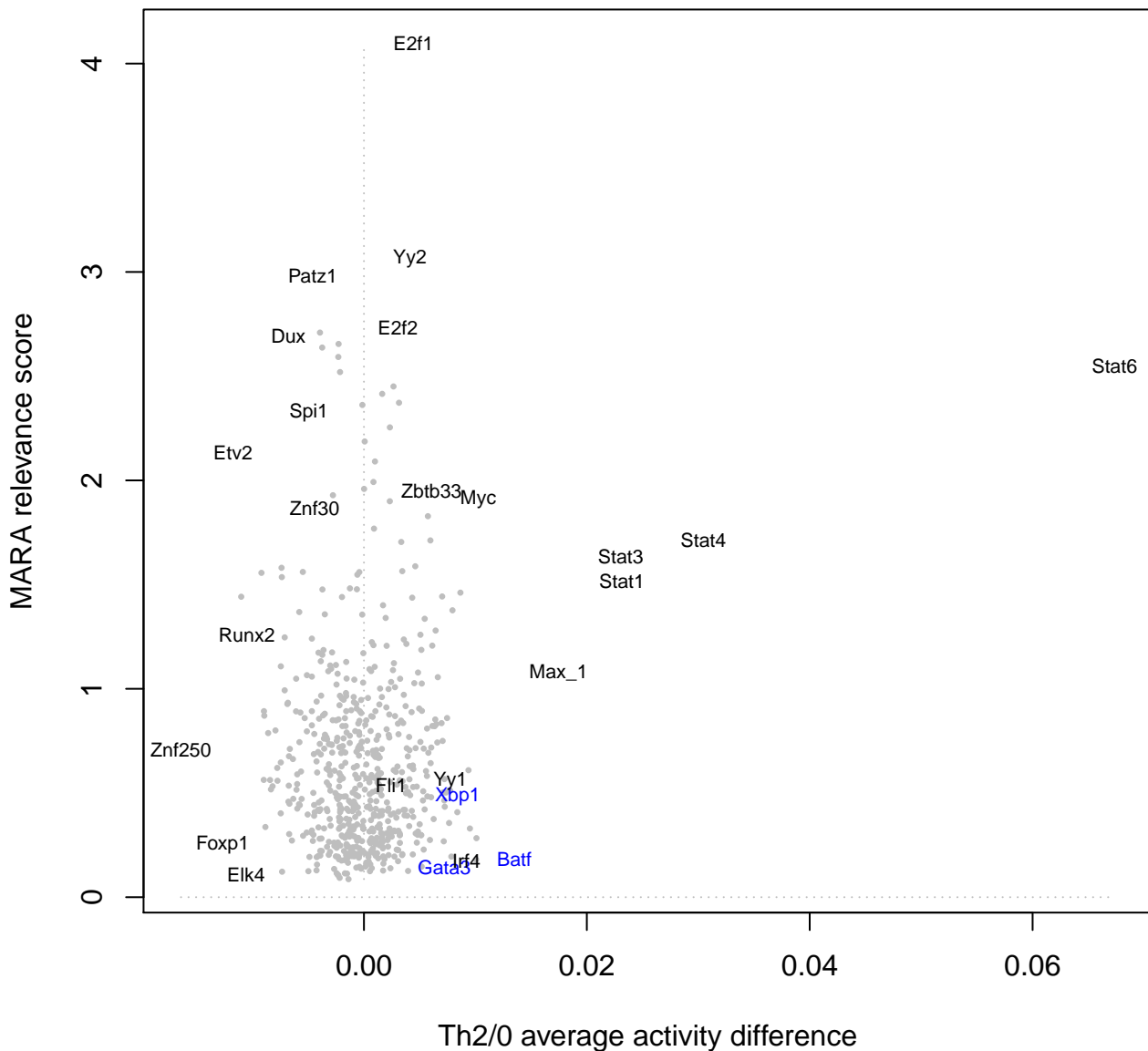

Supplement: Data S2. Processed Data from All the Steps of the Analysis, Related to Figure 1 [file mmc2.zip › supplemental data/mara/mouse_alltf_allgenes_alltime_th20_nonconsP/plot_diffvsZ.pdf]

Th2/0 activity difference

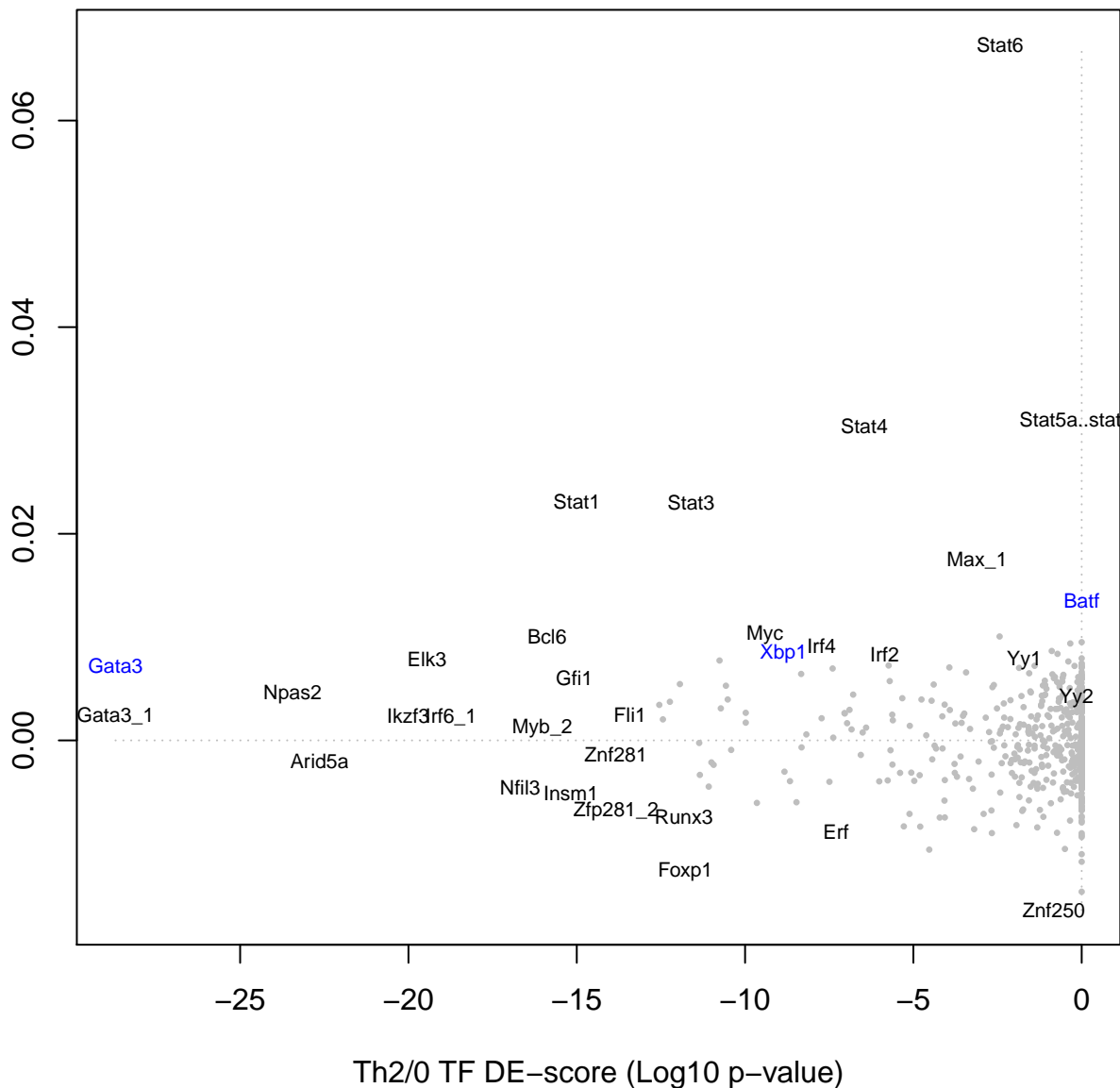

Supplement: Data S2. Processed Data from All the Steps of the Analysis, Related to Figure 1 [file mmc2.zip › supplemental data/mara/mouse_alltf_allgenes_alltime_th20_nonconsP/plot_diffvsDE.pdf]

Th2 Naive/72h activity difference

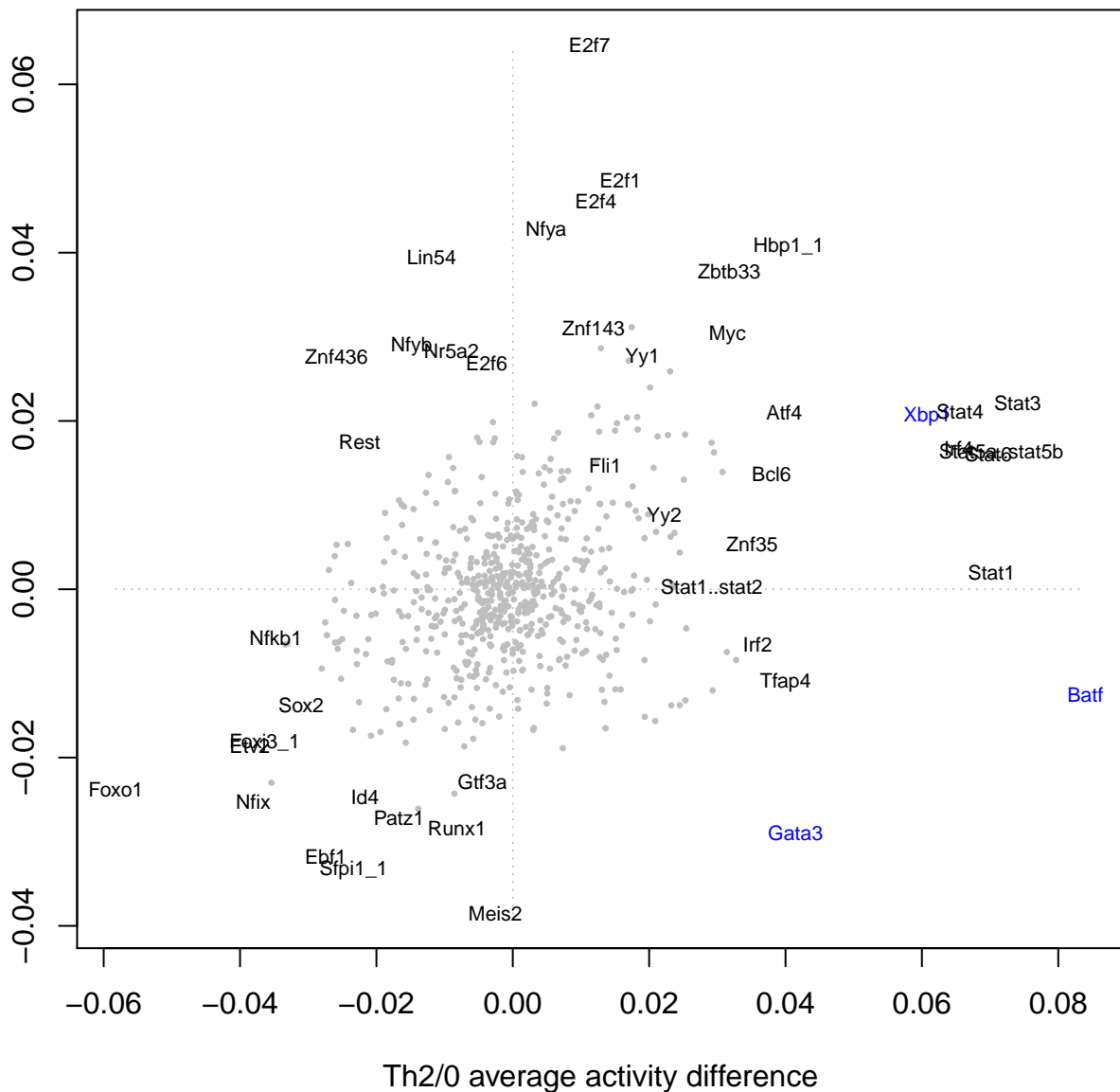

Supplement: Data S2. Processed Data from All the Steps of the Analysis, Related to Figure 1 [file mmc2.zip › supplemental data/mara/mouse_alltf_allgenes_alltime_th20/plot_diffvsdiff.pdf]

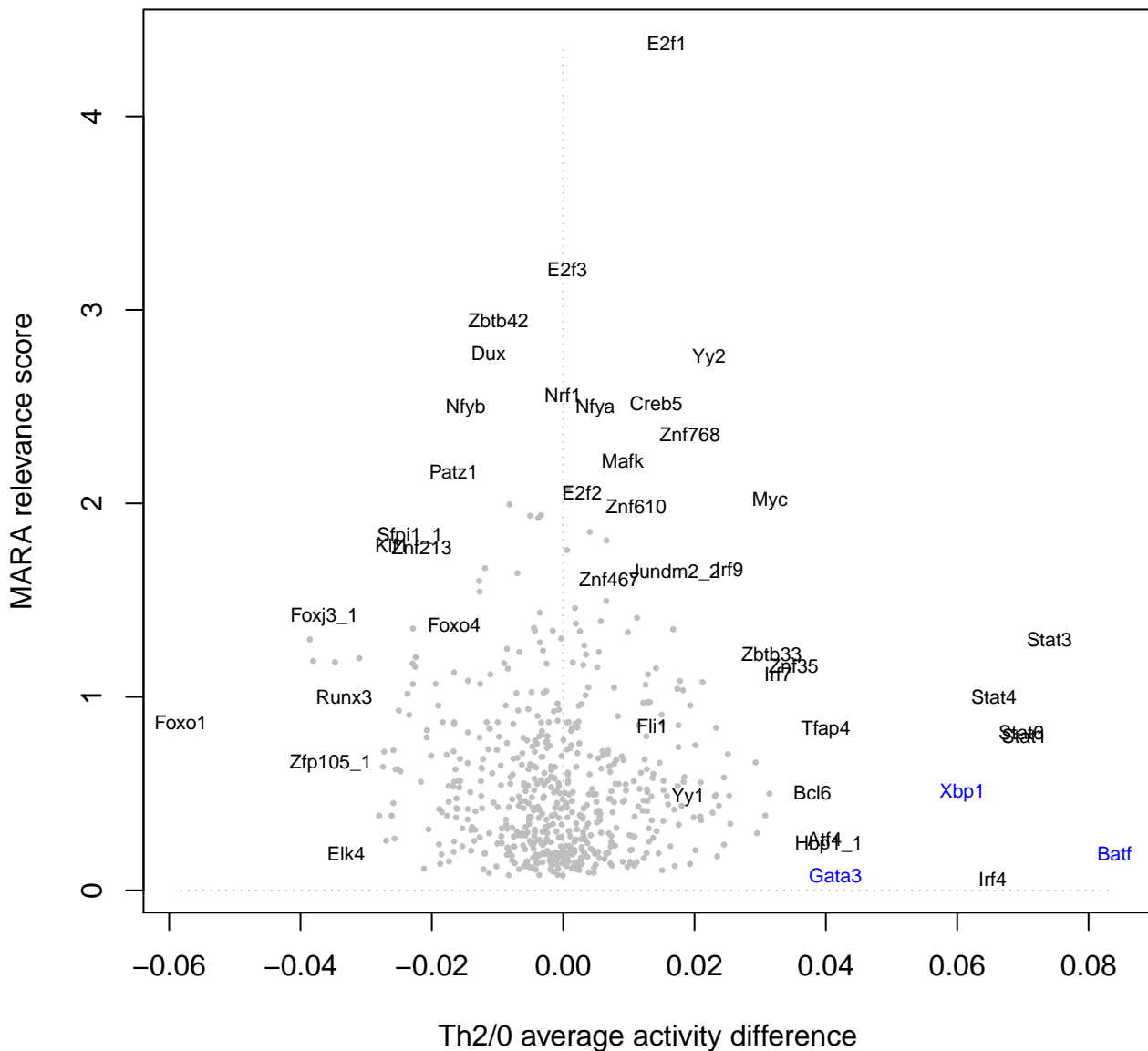

Supplement: Data S2. Processed Data from All the Steps of the Analysis, Related to Figure 1 [file mmc2.zip › supplemental data/mara/mouse_alltf_allgenes_alltime_th20/plot_diffvsZ.pdf]

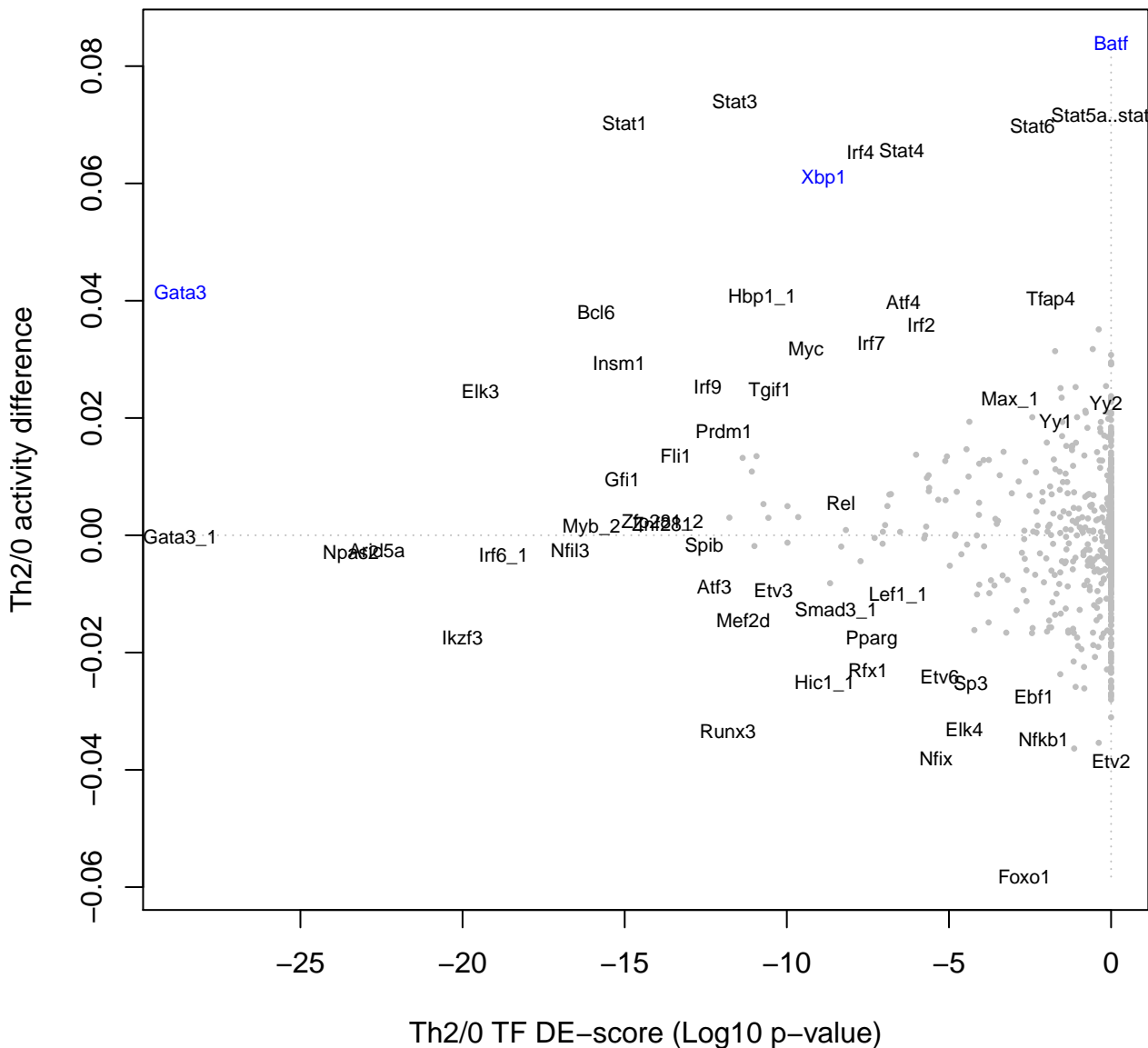

Supplement: Data S2. Processed Data from All the Steps of the Analysis, Related to Figure 1 [file mmc2.zip › supplemental data/mara/mouse_alltf_allgenes_alltime_th20/plot_diffvsDE.pdf]

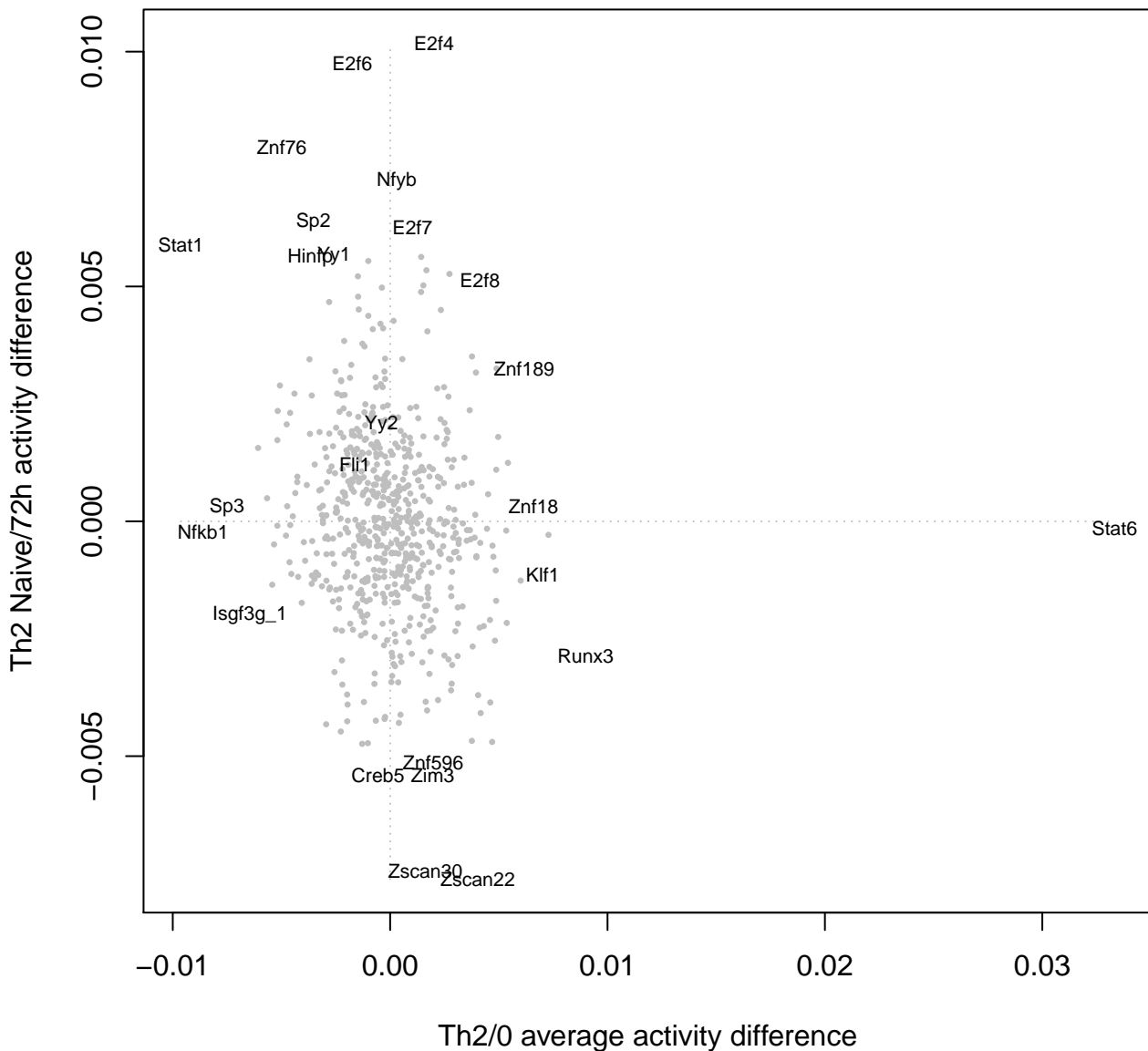

Supplement: Data S2. Processed Data from All the Steps of the Analysis, Related to Figure 1 [file mmc2.zip › supplemental data/mara/human_alltf_allgenes_alltime_th20_nonconsP/plot_diffvsdiff.pdf]

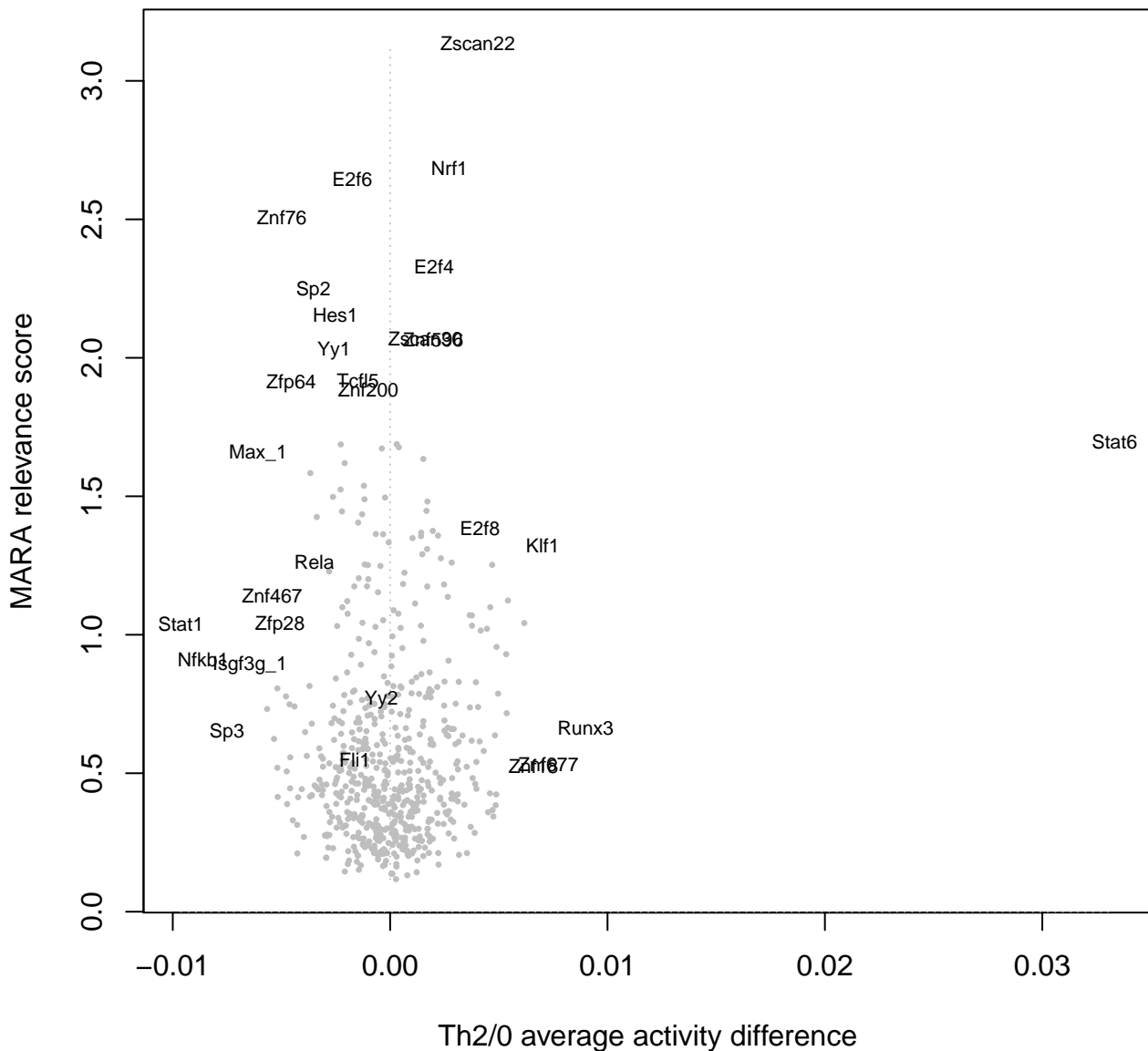

Supplement: Data S2. Processed Data from All the Steps of the Analysis, Related to Figure 1 [file mmc2.zip › supplemental data/mara/human_alltf_allgenes_alltime_th20_nonconsP/plot_diffvsZ.pdf]

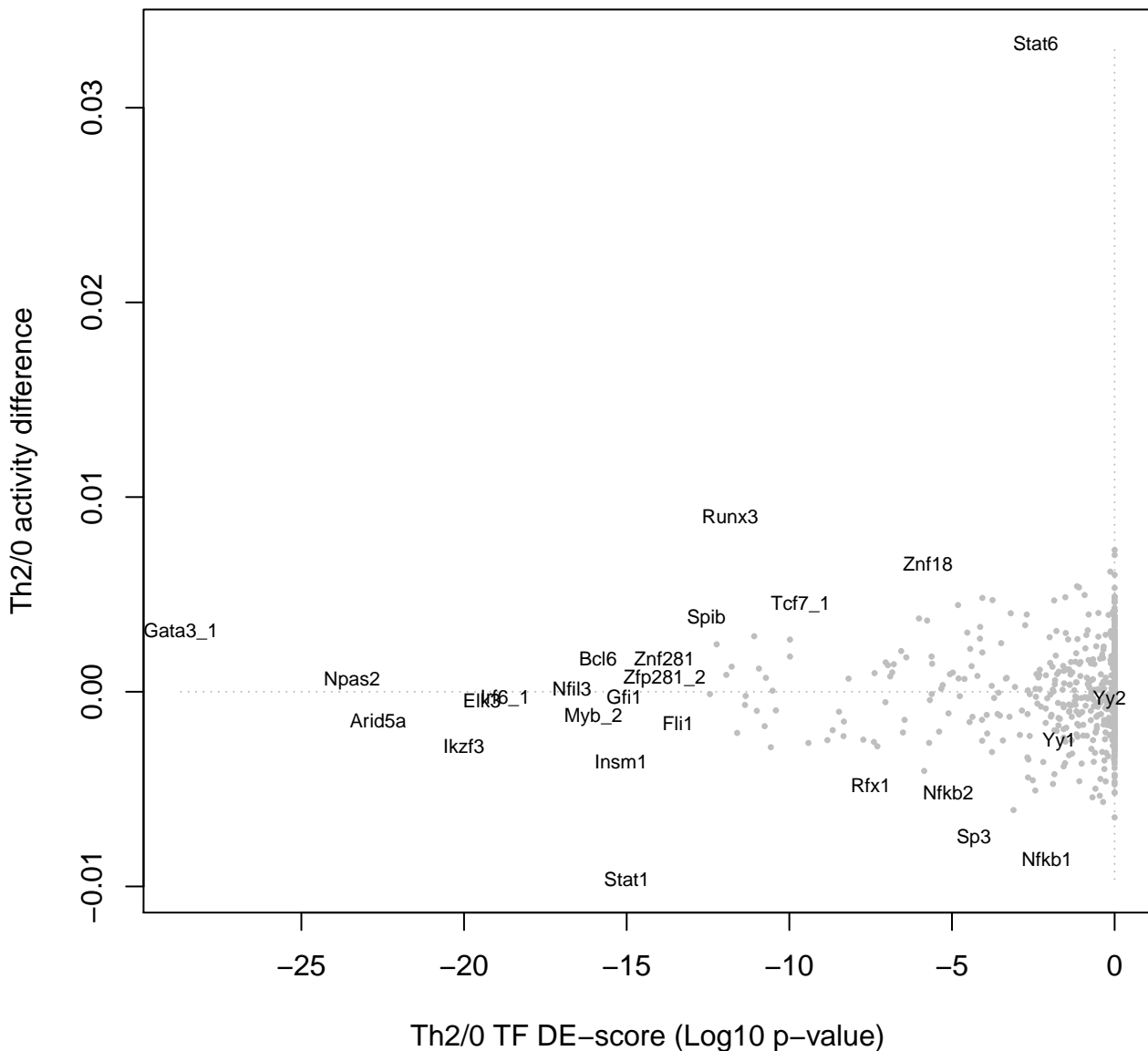

Supplement: Data S2. Processed Data from All the Steps of the Analysis, Related to Figure 1 [file mmc2.zip › supplemental data/mara/human_alltf_allgenes_alltime_th20_nonconsP/plot_diffvsDE.pdf]

Th2 Naive/72h activity difference

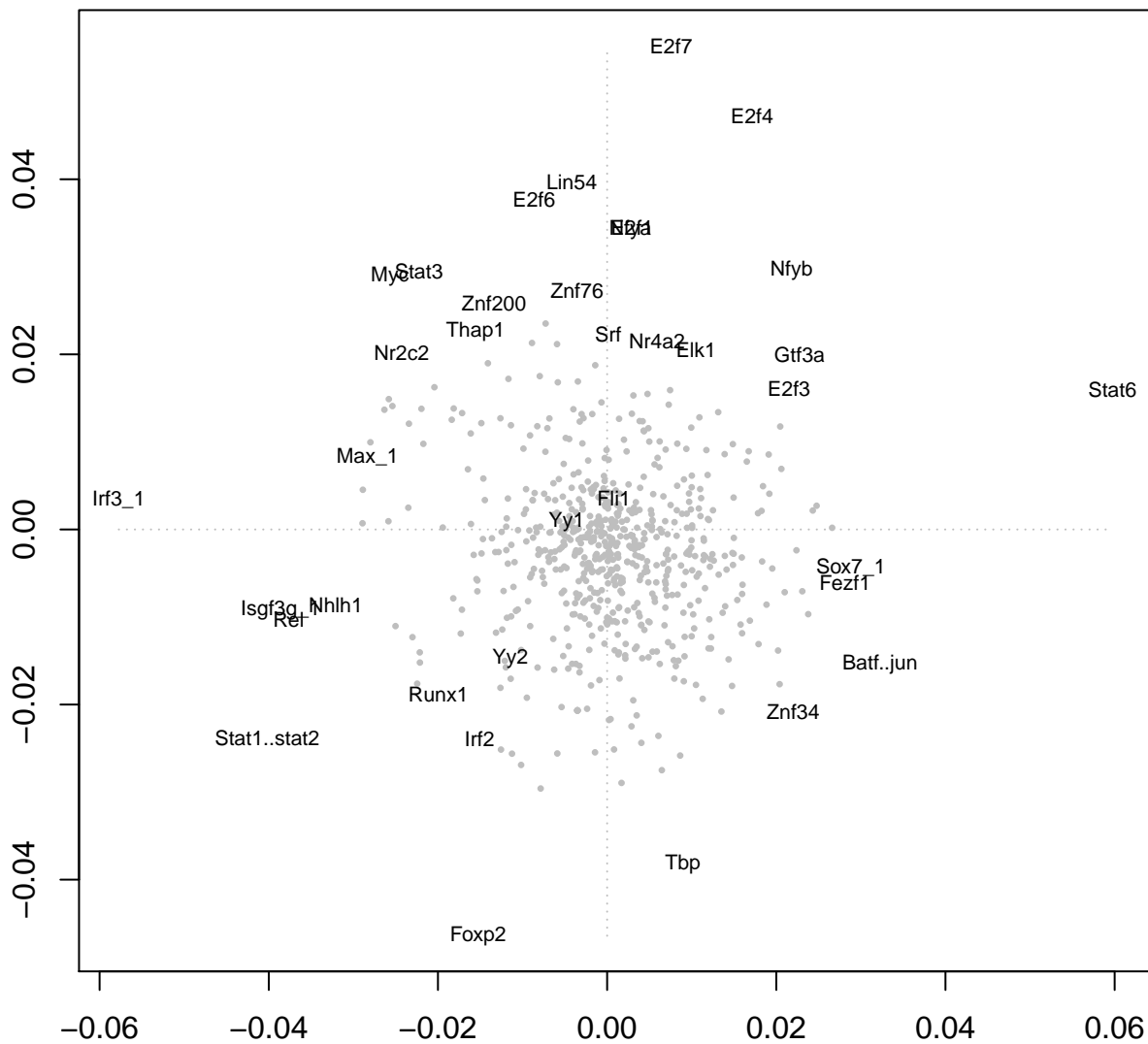

Th2/0 average activity difference

Supplement: Data S2. Processed Data from All the Steps of the Analysis, Related to Figure 1 [file mmc2.zip › supplemental data/mara/human_alltf_allgenes_alltime_th20/plot_diffvsdiff.pdf]

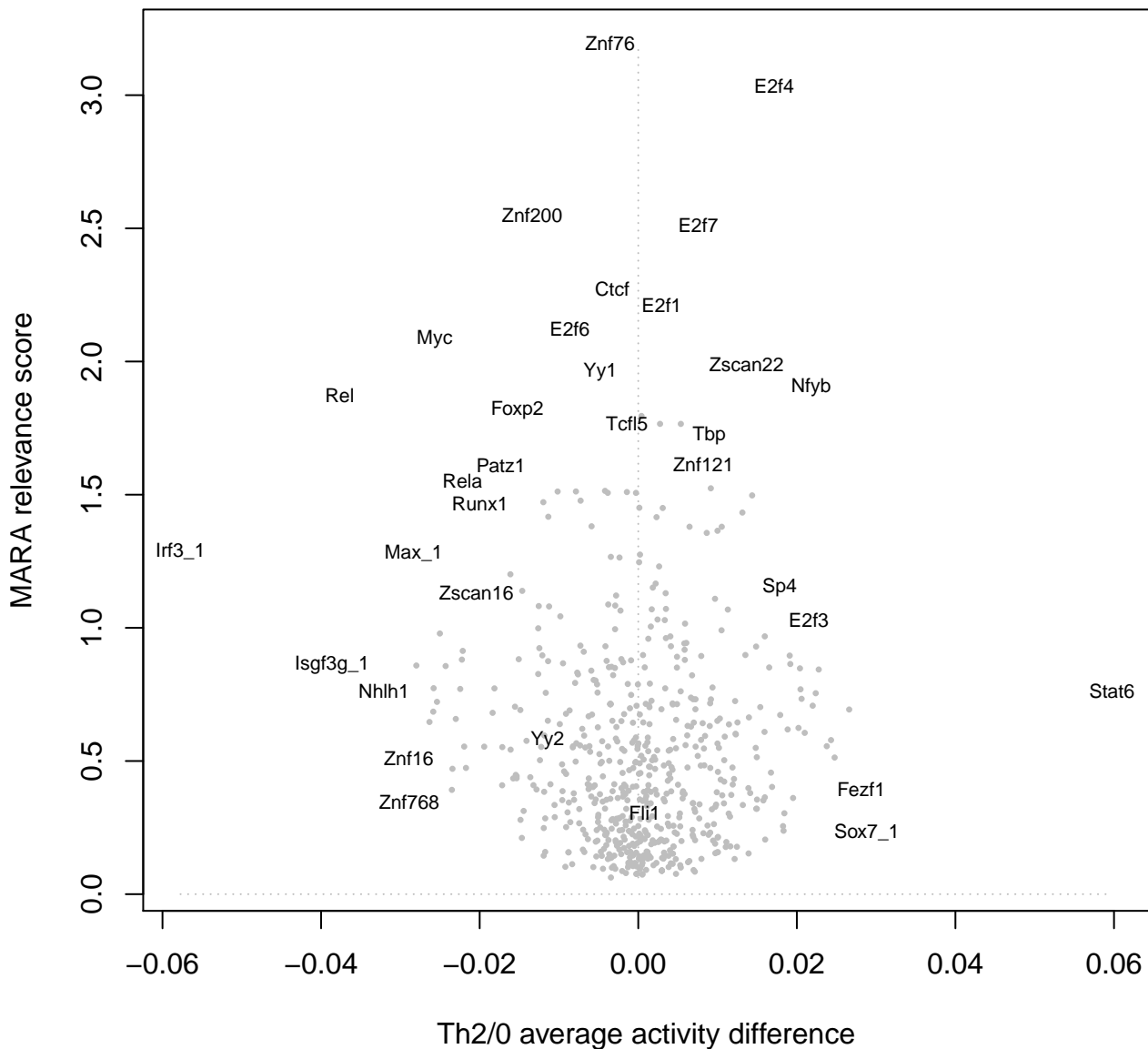

Supplement: Data S2. Processed Data from All the Steps of the Analysis, Related to Figure 1 [file mmc2.zip › supplemental data/mara/human_alltf_allgenes_alltime_th20/plot_diffvsZ.pdf]

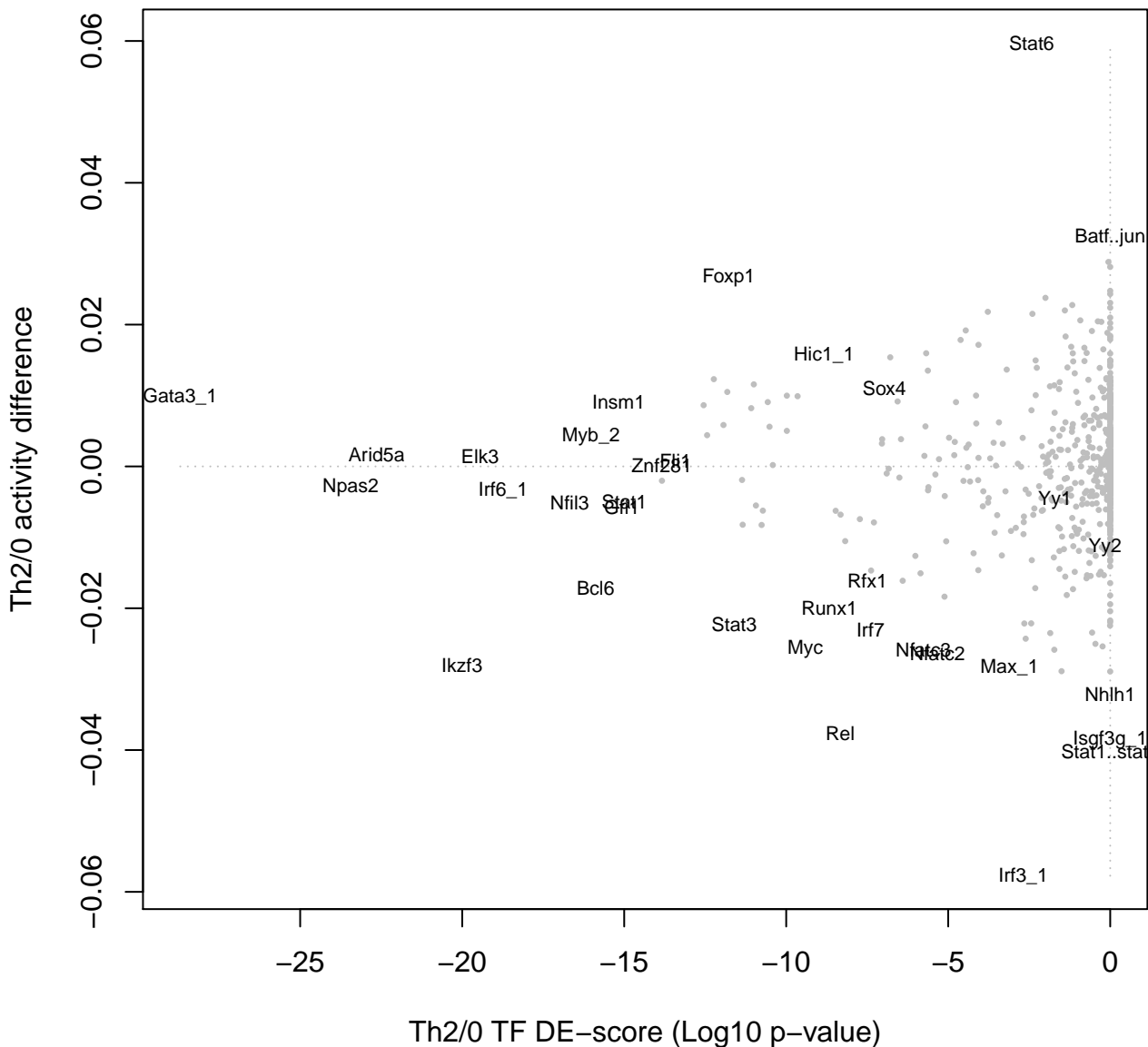

Supplement: Data S2. Processed Data from All the Steps of the Analysis, Related to Figure 1 [file mmc2.zip › supplemental data/mara/human_alltf_allgenes_alltime_th20/plot_diffvsDE.pdf]
